# Molecular targets of Chinese herbs: a clinical study of hepatoma based on network pharmacology

**DOI:** 10.1038/srep24944

**Published:** 2016-05-04

**Authors:** Li Gao, Xiao-dong Wang, Yang-yang Niu, Dan-dan Duan, Xue Yang, Jian Hao, Cui-hong Zhu, Dan Chen, Ke-xin Wang, Xue-mei Qin, Xiong-zhi Wu

**Affiliations:** 1 Modern Research Center for Traditional Chinese Medicine, Shanxi University, Taiyuan 030006, PR China; 2 Tianjin Medical University, Tianjin, 300070, China; 3 Tianjin Medical University Cancer Institute and Hospital, National Clinical Research Center for Cancer, Key Laboratory of Cancer Prevention and Therapy, Tianjin, 300060, China; 4 College of Chemistry and Chemical Engineering, Shanxi University, Taiyuan 030006, PR China; 5 Department of Pharmacology, Basic Medical College, Tianjin Medical University, Tianjin, 300070, China; 6 Tianjin People’s Hospital, NO.190 Jieyuan Road, Hongqiao, District, 300000, China

## Abstract

Traditional Chinese medicine (TCM) has been used to treat tumors for years and has been demonstrated to be effective. However, the underlying molecular mechanisms of herbs remain unclear. This study aims to ascertain molecular targets of herbs prolonging survival time of patients with advanced hepatocellular carcinoma (HCC) based on network pharmacology, and to establish a research method for accurate treatment of TCM. The survival benefit of TCM treatment with Chinese herbal medicine (CHM) was proved by Kaplan–Meier method and Cox regression analysis among 288 patients. The correlation between herbs and survival time was performed by bivariate correlation analysis. Network pharmacology method was utilized to construct the active ingredient-target networks of herbs that were responsible for the beneficial effects against HCC. Cox regression analysis showed CHM was an independent favorable prognostic factor. The median survival time was 13 months and the 5-year overall survival rates were 2.61% in the TCM group, while there were 6 months, 0 in the non-TCM group. Correlation analysis demonstrated that 8 herbs closely associated with prognosis. Network pharmacology analysis revealed that the 8 herbs regulated multiple HCC relative genes, among which the genes affected proliferation (*KRAS*, *AKT2*, *MAPK*), metastasis (*SRC*, *MMP*), angiogenesis (*PTGS2*) and apoptosis (*CASP3*) etc.

Hepatocellular carcinoma (HCC) is one of the most common malignant cancers worldwide^1^,^2^,^3^. More than 70% of new cases are diagnosed in Asia each year, and 50% are diagnosed in China alone^4^. The incidence of HCC is increasing and it is the second most common cause of cancer-related mortality^2^,^3^,^5^. The incidence and mortality rates are similar because most cases of HCC are diagnosed at an advanced stage^6^. Five-year survival rates had been improved but still less than 30%^7^. Previous studies reported that the median survival of patients with advanced HCC was less than 5 months^8^,^9^. But unfortunately, few therapies could be performed in advanced HCC (stage III–IV), only trans-arterial chemoembolization (TACE) and the drug Sorafenib have been shown to provide a survival benefit^10^,^11^.

Traditional Chinese medicine (TCM) has been widely used in China for thousands of years. Chinese herbal medicine (CHM) has become a treatment option in many cancer centers in Asia, Western countries and Africa^12^,^13^,^14^. Many clinical studies have shown that CHM is effective in the treatment of breast, gastric, lung and pancreatic cancers^15^,^16^,^17^. Our previous clinical and experimental studies indicated that CHM was effective on HCC^18^,^19^. However, whether patients with advanced HCC (stage III–IV) could obtain a benefit from CHM or not should be confirmed, and the mechanisms of CHM acting on HCC remain largely unsuspected.

Two famous Chinese herbal formulae, *Carapacis Trionycis Bolus* (*Synopsis of Golden Chamber*) and *Cang Niu Fangji Decoction* (*Synopsis of Golden Chamber*; Yao-zhong Fang, 1921–1995), are commonly used for cirrhosis, chronic liver disease and hepatocellular carcinoma. Chinese herbal formulae always contain many kinds of herbs and could have effect on many targets, so the anti-tumor mechanisms of herbal formulae are hard to understand. Encouragingly, the newly emerged network pharmacology approach, first proposed by Andrew L Hopkins^20^, brings the hope to study the effective constituents and targets of herbs in a traditional Chinese formula. As bioinformatics, systems biology and poly-pharmacology have rapidly progressed, network pharmacology method could clarify the mechanisms of drug action in a holistic way^21^,^22^,^23^,^24^. So, using the method, combined with the rich experience of TCM treatment, could hopefully shift the “one target, one drug” model to a “network targets, multi-component” strategy^25^. In recent years, there was an increasing concern about applying the network pharmacology to reveal the scientific basis and systematic features of CHM.

In the present work, the herbs are selected based on our clinical therapy; therefore it is much more reliable than those based on literature retrieval. The flowchart of the whole study design is illustrated in Fig. 1, and the following is a brief description: firstly, we proposed a method to screen out herbs that are effective for prolonging overall survival (OS) of patients with advanced HCC, and then predicted molecular mechanisms of these herbs by using the network pharmacology approach. This process would contribute to clarifying the molecular mechanisms of TCM and improving the effectiveness and specificity of TCM clinical treatment.

## Results

### Patient characteristics

In this study, a total of 288 patients with advanced HCC (stage III–IV) were included, and 227 (78.8%) patients were more than 50 years old when diagnosed. It has been demonstrated that age would not affect the survival time of patients with advanced HCC (*P* = 0.842) based on the univariate analysis. The baselines of patient demographics were summarized in Table 1. There were no significant differences in the patients’ age, gender, Child-Pugh score (A/B), etiology, serum AFP levels (<200 μg/L/>200 μg/L), palliative surgery (yes/no), TACE (yes/no), systemic chemotherapy (yes/no) and disease stage (III/IV) between the TCM and non-TCM groups.

### Univariate and multivariate analyses

The univariate analysis revealed that serum AFP (>200 μg/L) (*P* < 0.001) and clinical stage IV (*P* < 0.001) were significantly associated with the reduced median overall survival (Table 2). In contrast, TACE (*P* < 0.001), palliative surgery (*P* = 0.001) and TCM (*P* < 0.001) were protective factors.

Patient- and treatment-related variables were significantly associated with OS, and these results were then subsequently evaluated by Cox regression analysis to determine independent risk factors for the survival of patients with advanced HCC. Serum AFP > 200 μg/L and clinical stage IV were independent predictors of poor survival; TACE, palliative surgery and TCM were independent protective factors (Table 2).

### Survival analysis

**Figure 2:**
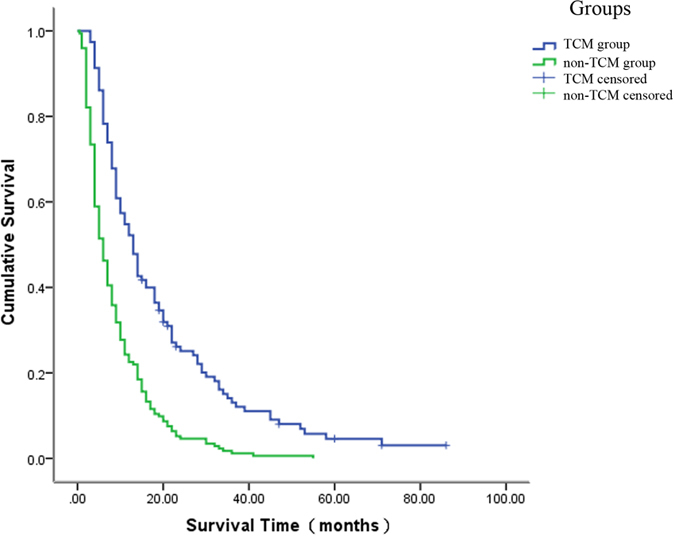
Survival analysis between the TCM and non-TCM group. The median OS in the TCM group was longer than that in the on-TCM group (13 vs 6 months, respectively; *P* < 0.001). OS = overall survival, TCM = traditional Chinese medicine.

The OS curves for TCM and non-TCM groups are shown in Fig. 2. The median survival time was 13 months vs 6 months and the 1-, 2-, and 5-year overall survival rates was 54.78%, 21.74%, 2.61% vs 24.3%, 4.62%, 0 in the TCM and non-TCM group, respectively. The Log-rank test revealed significant differences between the two groups in terms of OS (*P* < 0.001).

### Candidate genes associated with HCC

A total of 566 significant genes were obtained from the OncoDB. HCC^26^ and Liverome^27^ databases after removing redundant entries (supplementary file 1, Table S1). These genes were differentially expressed or harbored genetic variations in HCC tissues compared to normal tissues.

### Herbs, chemicals and targets information

All the Chinese herbal formulae used by 115 patients were collected. These formulae included a total of 240 types of herbs, and the highest using frequency was 425. There were 67 kinds of herbs, whose using frequency was more than 34 (425*8%) were selected to have a correlation analysis. Spearman bivariate correlate analysis showed 37 herbs had positive correlations with survival time, and the correlation coefficients of 20 herbs ≧0.25. Finally, 8 herbs (*Radix Stephaniae Tetrandrae* (RST), *Flos Campsis* (FC), *Carapax Trionycis* (CT), *Radix Scutellariae* (RS), *Radix Achyranthis Bidentatae* (RAB), *Radix Bupleuri* (RB), *Semen Lepidii* (SL), and *Rhizoma Atractylodis Macrocephalae* (RAM)) were found to have high correlation coefficients, and they were also components of the TCM formulae *Carapacis Trionycis Bolus* and *Cang Niu Fangji Decoction*.

For each of the 8 herbs, the ingredients with DL values ≥0.18 were listed in supplementary file 1, Table S2. As shown in Table 3, the 8 herbs were listed in descending order of their correlation coefficients. The detail target information for the ingredients of 8 herbs was shown in supplementary file 1, Table S3, and totally 9377 targets were found for the whole ingredients.

### Putative major ingredients and major targets of the 8 herbs

**Figure 3:**
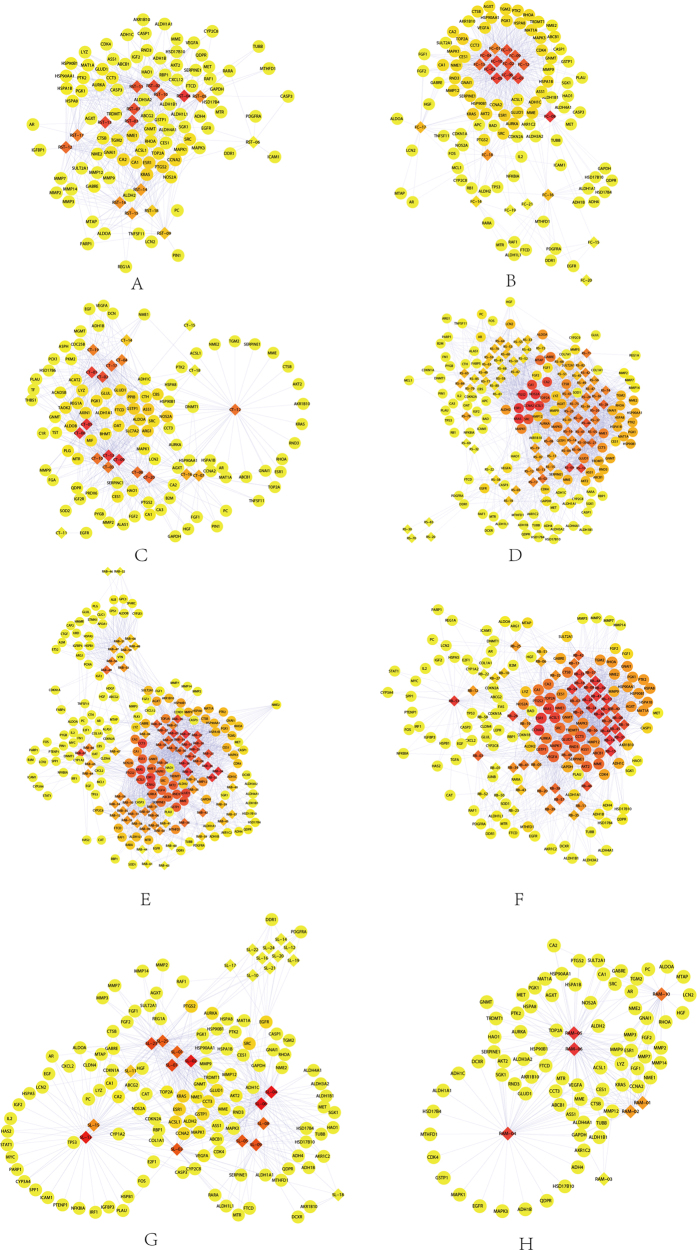
The ingredient-target networks of (**A**) RST, (**B**) FC, (**C**) CT, (**D**) RS, (**E**) RAB, (**F**) RB, (**G**) SL and (**H**) RAM. The diamond nodes represent ingredients, and the circular nodes represent targets. The colors of the nodes are illustrated from red to yellow in descending order of degree values.

Ingredient-target networks were constructed for the 8 herbs (Fig. 3). The results revealed that 2 major ingredients in RST, 1 in FC, 5 in CT, 5 in RS, 2 in RAB, 2 in RB, 3 in SL and 2 in RAM may play important roles in anti-HCC. The major putative ingredients and targets of each herb were listed in Table 3.

As shown in Fig. 3(A), the RST network was consisted of 111 nodes and contains 12 validated targets and 83 predicted targets. The analysis of the network revealed that beta-sitosterol (1 validated target, 59 predicted targets) was predicted as the major ingredient in RST. Genes such as *ESR1*, *KRAS* and *PTGS2* were predicted as the major targets of RST for the treatment of HCC. As shown in Fig. 3(B), the FC network was consisted of 116 nodes, including 22 validated targets and 73 predicted targets. Among the ingredients of FC, beta-sitosterol was also predicted as a major ingredient. Genes such as *ESR1*, *KRAS* and *AURKA* were predicted as major targets of FC for the treatment of HCC. The CT network consisted of 119 nodes and contained 1 validated target and 98 predicted targets (Fig. 3(C)). Galacturonic acid (0 validated targets, 52 predicted targets) was predicted as the major ingredient of CT for the treatment of HCC, and *ASS1*, *GSTP1* and *NOS2A* were predicted as the major targets of CT. The RS network consisted of 199 nodes, including 32 validated targets and 91 predicted targets (Fig. 3(D)). Beta-sitosterol was also the major ingredient, and genes such as *KRAS*, *CCNA2* and *ESR1* were predicted as major targets of RS for the treatment of HCC.

As shown in Fig. 3(E), the RAB network consisted of 224 nodes and contained 49 validated targets and 107 predicted targets. Beta-sitosterol was predicted as the major ingredient, and genes such as *ESR1*, *CCNA2* and *CCT3* were predicted as major targets of RAB for the treatment of HCC. As illustrated in Fig. 3(F), the RB network consisted of 186 nodes, including 49 validated targets and 81 predicted targets. Spinasterol was predicted as the major ingredient, and genes such as *CCNA2*, *ESR1* and *KRAS* were predicted as major targets of RB for the treatment of HCC. The SL network consisted of 144 nodes and contained 39 validated targets and 80 predicted targets (Fig. 3(G)). Beta-sitosterol was predicted as the major ingredient, and *ESR1*, *PTGS2* and *KRAS* were predicted as the major targets of SL for the treatment of HCC. The RAM network consisted of 88 nodes and contained 0 validated targets and 77 predicted targets (Fig. 3(H)). 3β-acetoxyatractylone was predicted as the major ingredient, and genes such as *MMP12*, *KRAS* and *GAPDH* were predicted as the major targets of RAM for the treatment of HCC.

### The major ingredients and underlying pharmacological mechanisms of TCM for the treatment of HCC

Although many ingredients may contribute to the HCC treatment effects of herbs, researching the major ingredients is an effective approach to elucidate the pharmacological mechanisms of action of herbs. In this study, network pharmacology was applied to explore the major ingredients. As shown in Table 3, beta-sitosterol was predicted as the major ingredient in 5 out of 8 herbs.

**Figure 4:**
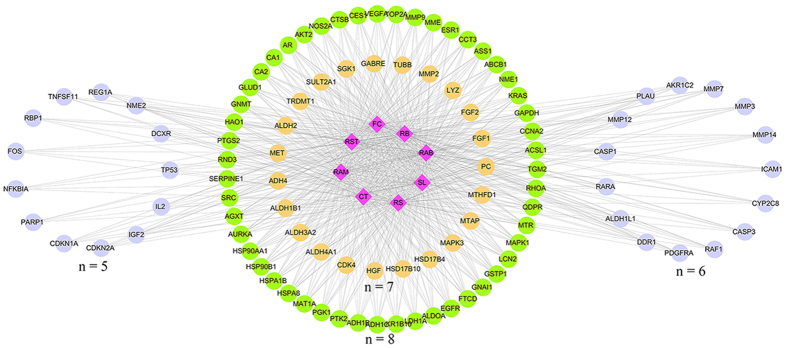
The herb-target networks of the 8 herbs. The diamond nodes represent herbs, and the circular nodes represent targets. The targets distributed in a circle represent they are acting by the same number of herbs, which illustrated as “n”.

**Figure 5:**
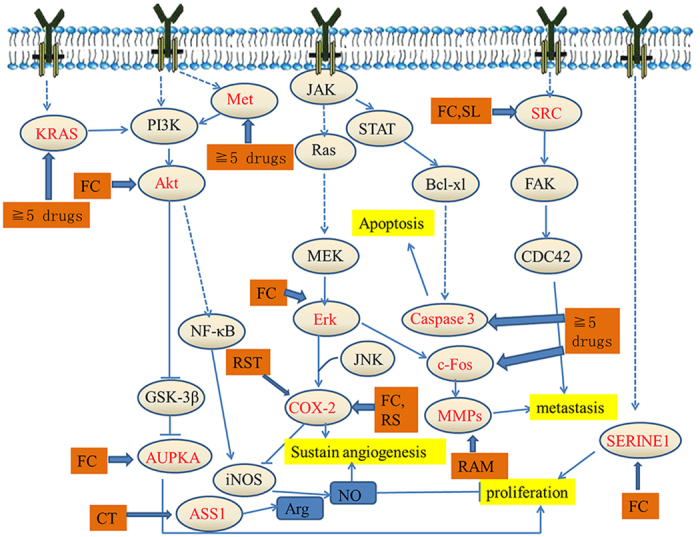
Simplified pathways in HCC. All of the targets are shown by the gene name.

The common targets for at least 5 herbs were illustrated in Fig. 4. Among these genes, 61 were common targets of 8 herbs, 23 were common targets of 7 herbs, 15 were common targets of 6 herbs, and 13 were common targets of 5 herbs. The representative genes included *ACSL1*, *ADH1C*, *ASS1*, *AURKA*, *CA1*, *CCNA2*, *CCT3*, *EGFR*, *ESR1*, *FTCD*, *GAPDH*, *GLUD1*, *GSTP1*, *KRAS*, *NME1*, *NOS2A*, *PTGS2*, *SRC*, *TOP2A* and *AKT2, MAPK*. Gene enrichment analysis found that the phosphatidylinositol 3′ kinase/Akt (PI3K/Akt) signaling pathway, nuclear SMAD2/3 signaling, and E-cadherin might be influenced by these herbs. The anti-HCC roles may mainly play in four fields: anti-angiogenesis, induction of apoptosis, inhibition of proliferation and metastasis (Fig. 5).

### Experimental validation

**Figure 6:**
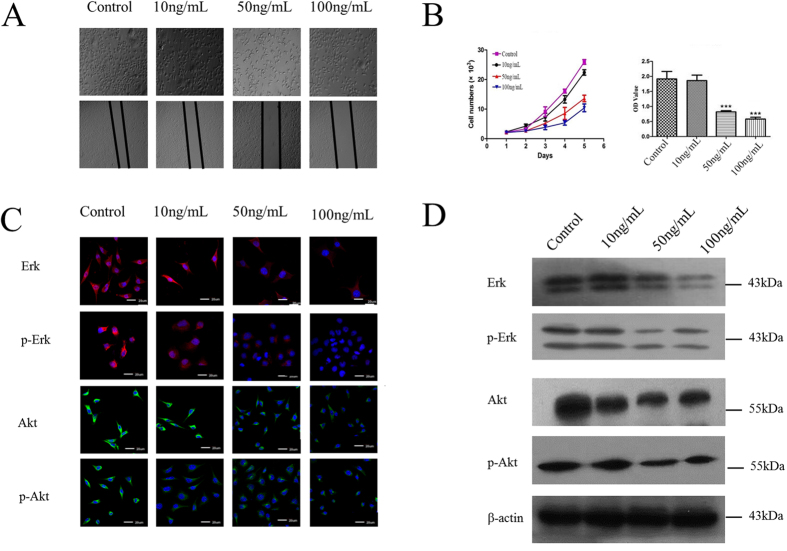
Effect of the aqueous extract of FC on cell proliferation and cell migration. Data are expressed as mean ± S.D. (**A**) The morphology of SMMC-7721 cells (upper) and the result of wound-scratch assay (lower). (**B**) The results of Typanblue staining assay (left) and CCK-8 assay (right), “***” represented for *p* < 0.001. (**C**) Immunocytochemical staining showed Erk, p-Erk, Akt and p-Akt of four groups. (**D**) Western blot assay analyzed Erk, p-Erk, Akt and p-Akt of four groups.

FC had high correlation coefficient with HCC (0.454), and multiple putative major targets (12) were selected for experimental validation. The effects of aqueous extract of FC on cell proliferation and wound healing assays were shown in Fig. 6(A); the statistical views of cell proliferation and cell migration after treating with FC were shown in Fig. 6(B). Our results revealed that aqueous of FC could obviously inhibit cell proliferation and cell migration (24 h) in dose of 50 ng/mL and 100 ng/mL *in vitro*.

Aqueous of FC affected protein expression and also affected phosphorylation progress of Erk and Akt. As shown in Fig. 6(C,D), FC significantly inhibited the expression of Erk and Akt, and decreased p-Erk and p-Akt in dose of 50 ng/mL and 100 ng/mL.

## Discussion

Many patients with HCC have a poor prognosis because most cases are diagnosed at the advanced stage^6^. In the field of western medicine (WM), many studies have focused on combination therapy for patients with unresectable HCC, but the prognostic improvement is limited. Studies on a variety of systemic cytotoxic chemotherapeutic agents have been reported in HCC^28^,^29^,^30^,^31^,^32^,^33^, however, most of these agents have not shown a survival benefit. TCM has been used for years to treat tumors worldwide especially in China and has been demonstrated effective in clinical practice. TCM seems appropriate for the treatment of cancer because the principle of TCM is to focus on systemic functional adjustments, which is in accordance with tumor treatment theories.

Our previous clinical study had proved TCM was effective in HCC patients from stage I to stage IV^18^. The present study included patients with advanced HCC (stage III–IV), of which the expected survival time was much shorter. Previous studies reported that the median survival of patients with advanced HCC without effective treatment was less than 5 months^8^,^9^. In the current study, the median survival times of the TCM group and the non-TCM group were 13 months and 6 months, and the survival time was significantly improved in the TCM group. These results indicated that TCM treatment was beneficial for improving survival time of patients with advanced HCC.

*Carapacis Trionycis Bolus* (*Synopsis of Golden Chamber*) and *Cang Niu Fangji Decoction* (*Synopsis of Golden Chamber*; Yao-zhong Fang, 1921–1995) are common used for cirrhosis, chronic liver disease and hepatocellular carcinoma in China. In the clinical treatment, herbs were used and adjusted according to different symptoms. At the end of the treatment, a total of 240 kinds of herbs were ever used. Correlation analysis showed 37 kinds of herbs had positive correlations with OS, and 20 kinds of herbs’ correlation coefficients were ≧0.25. We mainly concerned about 8 herbs, RST, FC, CT, RS, RAB, RB, SL and RAM, because FC, CT, RS, RB and SL were herbs of *Carapacis Trionycis Bolus*, RST, RAB, RAM were herbs of *Cang Niu Fangji Decoction*.

Network pharmacology approach could help us search for putative active ingredients and targets of herbs. 327 ingredients of these 8 herbs were found in total and 17 ingredients were screened out according to ingredients fishing rule for further research. Ingredients affected 95 targets in RST, 95 in FC, 99 in CT, 123 in RS, 156 in RAB, 130 in RB, 119 in SL and 77 in RAM. Furthermore, we found that 21 representative gene targets including *ACSL1*, *ADH1C*, *ASS1*, *AURKA*, *CA1*, *CCNA2*, *CCT3*, *EGFR*, *ESR1*, *FTCD*, *GAPDH*, *GLUD1*, *GSTP1*, *KRAS*, *NME1*, *NOS2A*, *PTGS2*, *SRC*, *TOP2A*, *AKT2* and *MAPK* were regulated by these herbs. Particularly, it was found that *AKT2* was the putative major target of FC. *AKT2* is related to tumor growth and metastasis in HCC, and is activated by PI3K pathway. Overexpression of *AKT2* is frequently observed in HCC and indicates poor prognosis^34^,^35^,^36^,^37^. Consistently, the experimental results showed that FC could inhibit the expression of Akt and phosphorylation of Akt, and could also obviously inhibit cell proliferation and cell migration *in vitro*. Furthermore, FC also had effects on *MAPK1*, whose activation promotes proliferation and metastasis in HCC^36^.

Repressing HCC progression and metastasis is important for prolonging the OS of patients with HCC. Other targets, such as *CCNA2,* involved in repressing the expression of cyclin E and tumor cell proliferation^38^. *SRC2*, *NME1*, *GSTP1*, *FTCD3*, *COX-2*, *AUPKA*, *ASS1* and *ADH1C* associated with HCC. Beta-sitosterol, frequently predicted as the major ingredient of herbs, was found as a potential therapeutic agent for the hepatofibrosis in activating human hepatic stellate cell (HSC) model and dimethyl nitrosamine (DMN)-induced mouse hepatic fibrosis model^39^. Baicalin, a component of RS, had anticancer effects such as inducing autophagy and anti-proliferative function of tumor cells of HCC^40^. These facts support our research and confirm that TCM could affect HCC.

We conclude, from this retrospective study, that TCM treatment was associated with a survival benefit in patients with HCC. In addition, the use of network pharmacology method is beneficial for exploring the underlying anti-tumor mechanisms of herbs through prediction of active ingredients and molecular targets of herbs. The combination of clinical study and network pharmacology is a promising research method for “precise treatment of TCM”.

There are several limitations for the use of network pharmacology approach to predict active ingredients and potential targets. (i) the ingredients of herbs screened by DL values may be inconsistent with the ingredients actually existed in blood of the patients; (ii) the predicted targets were influenced by different target prediction tools; (iii) it is difficult to distinguish inhibitory effects from activated effects of the targets.

## Materials and Methods

### Statement

This retrospective study was supported by Medical Ethics Committee and the methods were carried out in accordance with the approved guidelines (The certificate no. 2016.4). Our study did not pose risk to patients, and did not have negative effects on patients’ rights and health. The subjects’ privacy and personal identity information were protected, and the majority of patients had died or lost to follow-up and unable to get in touch. According to guidelines of exempt from inform consent of Tianjin Medical University Cancer Institute and Hospital, our study meet the requirement of informed consent exemption.

### Patient characteristics

From March 2009 to December 2014, 389 patients with HCC were followed up. Major inclusion criteria were as follows: aged 18 years or older; diagnosed by histology, cytology, or clinical and radiographic evidence. Moreover, patients in the TCM group should receive TCM treatment ≧2 months. The major exclusion criteria were as follows: serious complications (severe cardiac or pulmonary disease; acute upper gastrointestinal bleeding leading to death); Child-Pugh Class C; concurrent cancer; incomplete medical records; radical surgery or liver transplantation; or no accurate survival time. Diagnostic criteria included pathogenesis as well as clinical and radiographic evidence^11^. Baseline staging was performed based on age, gender, Child-Pugh score, serum alpha fetal protein (AFP) levels, palliative surgery, TACE, systemic chemotherapy and clinical stage. After screening, 288 patients were included in our study. Finally, 115 patients who received TCM together with WM were included in TCM group.

### Treatment

TCM treatment was used according to different syndromes. In general, a formula contains 20–30 types of herbs in our study for patients with advanced HCC. The formula was taken orally 3 times a day, 30 minutes after meals, for 2 months or more in the TCM group. Every 2 weeks, some herbs were changed based on the patient’s symptoms. During TCM treatment, patients in the TCM group also received WM treatment. The patients in the non-TCM group only received WM treatment. The WM treatment included palliative surgery, TACE and systemic chemotherapy. Local radiation therapy and biotherapy were rarely used in patients.

Patients’ formulae were gathered every 2 weeks counted from the date received TCM treatment to the date the patients dead or the time of data closure. We analyzed the TCM formulae of 115 patients, who received 62940 days of TCM treatment with CHM and 240 types of herbs in total. The frequency of single-herb treatment was balanced by dividing the average treatment days and patient numbers. The frequency of single herb over 8% was selected out, and correlation coefficients between these herbs and survival time were calculated separately. The average of the correlation coefficient, which was 0.25, was set as a boundary for further selection. Herbs were included for further analysis if they satisfy the following criteria: using frequency/highest frequency >8%; correlation coefficient ≧0.25 and *P*-value < 0.05.

### HCC significant genes collection

HCC significant genes were collected from 2 databases, OncoDB.HCC^26^ (http://oncodb.hcc.ibms.sinica.edu.tw) and Liverome^27^ (http://liverome.kobic.re.kr/index.php). A gene was selected if it satisfied at least one of the following criteria: (i) experimentally validated in OncoDB.HCC, or (ii) occurrence frequency >7 among the various collected gene signatures in Liverome. The repeated genes between the two databases were removed. To normalize the gene information, different ID types were converted to Swiss-Prot accession numbers.

### Herb formulation ingredient collection and target fishing

The chemical ingredients were collected form TCM databases, including the Traditional Chinese Medicine Systems Pharmacology (TCMSP) Database^41^ (http://lsp.nwsuaf.edu.cn/tcmsp.php), the Traditional Chinese Medicine Integrated Database^42^ (TCMID, http://www.megabionet.org/tcmid/) and the TCM Potential Target Database (TCM-PTD, http://tcm.zju.edu.cn/ptd/). The ingredients were screened according to drug-likeness (DL) values, and the ingredients were retained if DL ≥ 0.18, a suggested criterion by TCMSP database. Specifically, the ingredients of CT were amino acids with low DL values; therefore they were not screened by these criteria.

Target fishing was performed to search for or predict the potential targets of small molecules. The validated targets were extracted from the Herbal Ingredients’ Targets (HIT) Database^43^ (http://lifecenter.sgst.cn/hit/). The predicted targets were obtained using ChemMapper^44^ (http://lilab.ecust.edu.cn/chemmapper/), an online tool for predicting targets based on 3D similarity. In this study, if the 3D similarity of a molecule was above 0.85^45^ compared with drugs in the DrugBank database, the targets of known drugs were predicted as the targets of the investigated molecules. The targets were retained if they were common targets between HCC and small molecules, and the prediction score was >0 as well.

### Network construction and analysis

The ingredients-targets networks were constructed for these herbs using Cytoscape software^46^ (Version 3.2.1). The networks were analyzed using Cytoscape plugin CentiScaPe^47^ to calculate topological parameters, including the degree, betweenness, closeness and centroid. The significant nodes representing putative major ingredients and major targets of herbs were explored. Except for CT whose ingredients were amino acids, the putative major ingredients were remained if DL ≥ 0.18 and oral bioavailability values ≥30%.

### Pathways analysis

All pathways associated with HCC were searched through the internet (http://cgap.nci.nih.gov/Pathways/BioCarta_Pathways, http://www.cellsignal.com and http://www.genome.ad.jp). The mechanisms of herbs were mapped to the pathway network according to their targets information.

### Experimental validation

Hepatocarcinoma cell line SMMC-7721 was used to examine the effects of aqueous extract of FC.

### Cell culture and morphologic observation

SMMC-7721 cells were obtained from the Tianjin cancer institute & hospital. Cells were cultured as described in literature. When SMMC-7721 cells reached 60% confluency, the cells were then continuously exposed to 10 ng/mL, 50 ng/mL and 100 ng/mL aqueous extract of FC. Photos were taken by inverted phase contrast microscope (Olympus).

### Wound-scratch assay, Typanblue staining assay and CCK-8 assay

Wound-scratch assay was performed as previously described. CCK-8 assay was performed as manual. Typanblue staining was performed as described^19^.

### Antibodies

The following antibodies were used: Erk (Cell Signaling Technology 4695, Danvers, MA, USA), Phospho-Erk1/2 (Cell Signaling Technology 4370, Danvers, MA, USA), β-actin (Cell Signaling Technology 3700), Akt (Cell Signaling Technology 4691, Danvers, MA, USA), Phospho-Akt (Cell Signaling 4060, Danvers, MA, USA).

### Immunocytochemical staining

Immunocytochemical staining was performed according to the method published before^19^. The staining results were repeated at least three times. The representative pictures were shown in related figures captured by confocal microscope (Olympus Corporation, Beijing, China).

### Western blot assay

Western blot analyses were operated as previously described^48^. The results were repeated at least three times.

### Statistics

Baseline comparison was analyzed by the χ^2^ test. OS from the date of the definitive diagnosis of HCC was estimated by using the Kaplan–Meier method, and survival curves were established by the log-rank test. The continuous variables were divided into categories based on population quartiles, upper normal values, and published data to determine cutoff values. Multivariate Cox regression analysis was performed to determine survival trends adjusted for clinical and demographic factors. Spearman bivariate correlate analysis was used in searching for correlation between herbs and survival time. A P-value < 0.05 was considered to indicate statistical significance. Statistical analyses were performed by SPSS 19.0 for Windows.

## Additional Information

**How to cite this article**: Gao, L. *et al.* Molecular targets of Chinese herbs: a clinical study of hepatoma based on network pharmacology. *Sci. Rep.* **6**, 24944; doi: 10.1038/srep24944 (2016).

## Supplementary Material

Supplementary Information

Supplementary Information

Supplementary Information

## Figures and Tables

**Figure 1:**
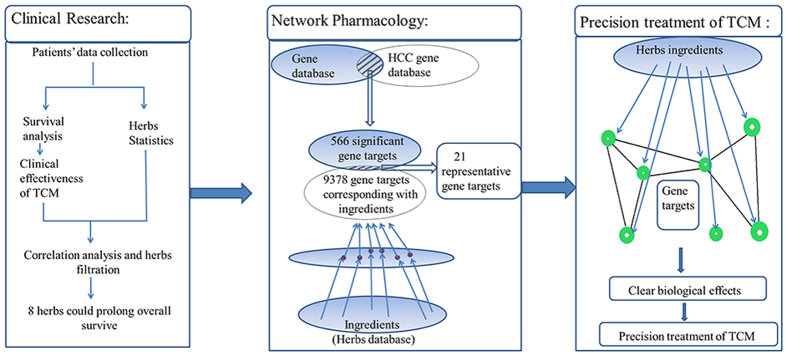
Process overview.

**Table 1:** Patients’ baseline characteristics and treatments between TCM group and non-TCM group.

		TCM	non-TCM	
t Variable		(n = 115)	(n = 173)	*P-* value
Age	<30	1	0	0.176
	30–40	9	5	
	40–50	17	29	
	50–60	40	89	
	60–70	31	36	
	>70	17	14	
Gender (male/female)		94/21	136/37	0.517
Child-Pugh stage (A/B)		102/13	140/33	0.053
Etiology	Alcohol	17	29	0.237
	HBV/HCV	26	45	
	HBV/HCV+Alcohol	6	18	
	Cryptogenic	66	81	
Serum AFP levels (<200μg/L/>200μg/L)		88/27	126/47	0.483
Palliative Surgery (yes/no)		29/86	28/145	0.060
TACE (yes/no)		46/69	67/106	0.829
Systemic Treatment (yes/no)		8/107	22/151	0.117
Clinical Stage Composition (IIIB- IIIC /IV)		96/19	142/31	0.759

Abbreviations: AFP: Alpha-fetoprotein; HBV: Hepatitis B Virus; HCV: Hepatitis C Virus; TACE: Transcatheter arterial chemoembolization.

**Table 2:** Univariate and Multivariate Analyses of Variables Influencing Survival of 288 Patients with HCC.

Characteristics	Univariate Analysis			Multivariate Analysis		
	N (%)	*P*-Value	β	Exp (β)	95% CI for Exp (β)	*P*-Value
Gender		0.908	–	–	–	–
Male	230 (79.9)					
Female	58 (20.1)					
Age, years		0.842	–	–	–	–
<50	61 (21.2)					
≥50	227 (78.8)					
Child-Pugh stage		0.126	–	–	–	–
A	242 (84.0)					
B	46 (16.0)					
Serum AFP	214 (74.3)	**<0.001**	−0.493	0.611		0.001
<200μg/L	74 (25.7)				0.457–0.817	
>200μg/L						
TACE		**<0.001**	0.597	1.816		<0.001
Yes	113 (39.2)				1.362–2.421	
No	175 (60.8)					
Palliative Surgery		**0.001**	0.460	1.584		0.006
Yes	57 (19.8)				1.142–2.198	
No	231 (80.2)					
Systemic Chemotherapy		0.064	–	–	–	–
Yes	28 (9.7)					
No	260 (90.3)					
Clinical Stage Composition		**<0.001**	−1.072	0.342		<0.001
III	197 (68.4)				0.247–0.474	
IV	91 (31.6)					
TCM		**<0.001**	0.845	2.328		<0.001
Yes	115 (39.9)				1.796–3.017	
No	173 (60.1)					

*P*-values in bold font are statistically significant. TACE: Transcatheter arterial chemoembolization; AFP: Alpha-fetoprotein; CI: confidence interval; TCM: Traditional Chinese medicine.

**Table 3:**
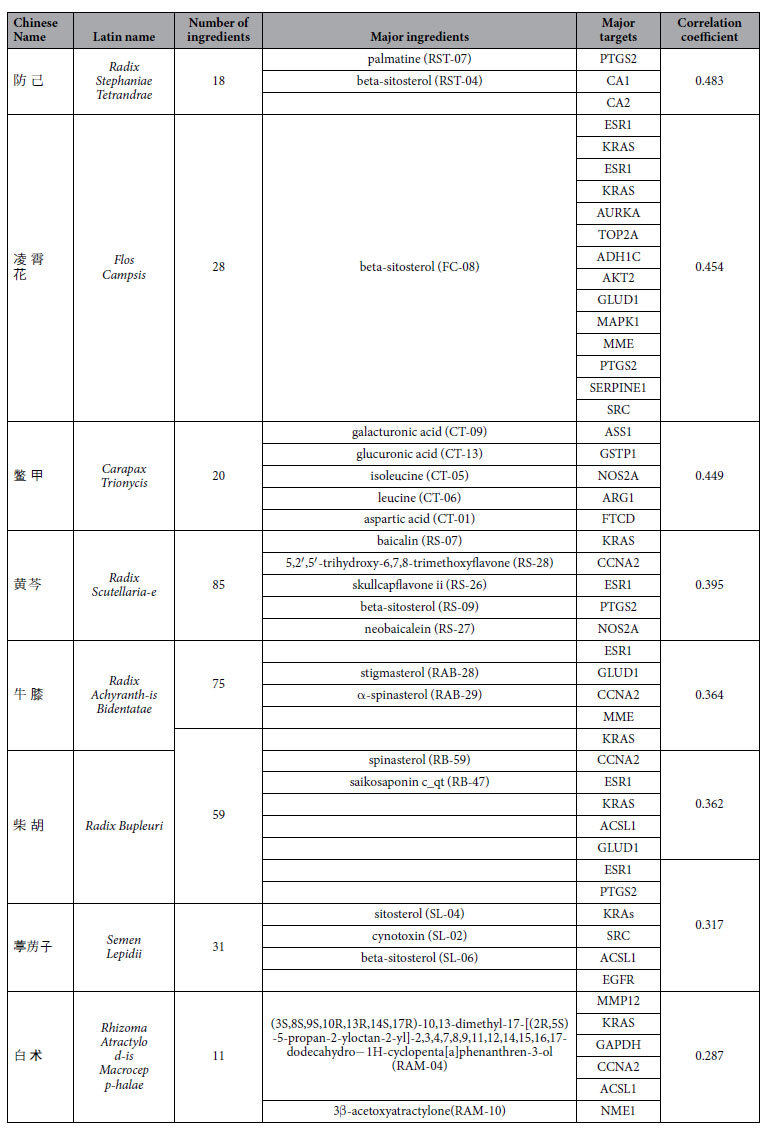
The putative major ingredients and major targets of 8 herbs.
