# Supplementary material for: Molecular targets of Chinese herbs: a clinical study of hepatoma based on network pharmacology
Source: Sci Rep. 2016 May 4;6:24944. doi: 10.1038/srep24944 (PMC4855233; doi:10.1038/srep24944)
Supplement: Supplementary Information [file srep24944-s1.doc]

| syndrome | herb | Correlation coefficient |
| --- | --- | --- |
| Stagnation of Liver-qi | *Carapax Trionycis*  *Eupolyphaga Seu Steleophaga*  *Radix Bupleuri*  *Radix Scutellariae*  *Rhizoma Pinelliae*  *Radix Changii*  *Ramulus Cinnamomi*  *Radix Paeoniae Alba*  *Cortex Moutan Radicis*  *Flos Campsis*  *Semen Lepidii*  *Radix Bupleuri*  *Radix Scutellariae*  *Gekko japonicus Dumeril et Bibron*  *Radix Notoginseng*  *Radix Curcumae*  *Fructus Akebiae* | 0.512  0.147  0.410  0.423  0.145  0.384  0.101  0.365  0.342  0.474  0.362  0.392  0.421  0.432  0.353  0.567  0.417 |
|  |  |  |
| Dampness-heat in  Liver channe | *Herba Artemisiae Scopariae*  *Radix Scutellariae*  *Actinidia valvata Dunn*  *Rhizoma Bolbostemmae* | 0.212  0.442  0.312  0.463 |
| Deficiency of Spleen  and Kidney | *Rhizoma Atractylodis*  *Radix Achyranthis Bidentatae*  *Herba Taxilli*  *Radix Stephaniae Tetrandrae*  *Radix Notoginseng*  *Gekko japonicus Dumeril et Bibron*  *Radix Pseudostellariae*  *Radix Astragali seu Hedysar*  *Rhizoma Atractylodis Macrocephalae* | 0.197  0.394  0.291  0.483  0.353  0.512  0.096  0.114  0.356 |

Table S5 TCM syndromes based effective herbs.

As shown in this table, the 8 kinds of herbs, *Radix Stephaniae Tetrandrae* (RST)*, Flos Campsis* (FC)*, Carapax Trionycis* (CT)*, Radix Scutellariae* (RS)*, Radix Achyranthis Bidentatae* (RAB)*, Radix Bupleuri* (RB)*, Semen Lepidii* (SL)*, and Rhizoma Atractylodis Macrocephalae* (RAM), have highly correlation with overall survival. *Radix Changii, Radix Paeoniae Alba, Cortex Moutan Radicis, Radix Notoginseng, Fructus Akebiae, Actinidia valvata Dunn, Rhizoma Bolbostemmae* and *Herba Taxilli* also have higuly correlation coefficient, but the using frequency of them are much lower. What attracts us most is *Gekko japonicus Dumeril et Bibron,* our previous study has shown it has many anti-tumor functions. It’s mainly ingredient is *Gekko-sulfated Glycopeptide*. But network pharmacology hasn’t concerned about this kind of herb yet.
