# Supplementary material for: Molecular targets of Chinese herbs: a clinical study of hepatoma based on network pharmacology
Source: Sci Rep. 2016 May 4;6:24944. doi: 10.1038/srep24944 (PMC4855233; doi:10.1038/srep24944)
Supplement: Supplementary Information [file srep24944-s2.doc]

**Molecular targets of Chinese herbs: a clinical study of hepatoma based on network pharmacology**

Li Gao^&^, Xiao-dong Wang ^&^, Yang-yang Niu ^&^, Dan-dan Duan, Xue Yang, Jian Hao, Cui-hong Zhu, Dan Chen,

Ke-xin Wang, Xue-mei Qin, Xiong-zhi Wu^*^

**Supplementary files**

**Table S1**: The 566 significant genes associated with HCC.

| **Gene symbol** | **Swiss-Prot ID** | **Gene name** | **Database** |
| --- | --- | --- | --- |
| A2M | P01023 | alpha-2-macroglobulin | Liverome/OncoDB.HCC |
| AGXT | P21549 | alanine-glyoxylate aminotransferase | Liverome/OncoDB.HCC |
| AKR1B10 | O60218 | aldo-keto reductase family 1, member B10 (aldose reductase) | Liverome/OncoDB.HCC |
| ALB | P02768 | albumin | Liverome/OncoDB.HCC |
| ALDOB | [P05062](http://www.uniprot.org/entry/P05062) | aldolase B, fructose-bisphosphate | Liverome/OncoDB.HCC |
| APOA1 | P02647 | apolipoprotein A-I | Liverome/OncoDB.HCC |
| ARG1 | P05089 | arginase, liver | Liverome/OncoDB.HCC |
| ASS1 | P00966 | argininosuccinate synthase 1 | Liverome/OncoDB.HCC |
| CA2 | P00918 | carbonic anhydrase II | Liverome/OncoDB.HCC |
| CAP2 | P40123 | CAP, adenylate cyclase-associated protein, 2 (yeast) | Liverome/OncoDB.HCC |
| CCT3 | P49368 | chaperonin containing TCP1, subunit 3 (gamma) | Liverome/OncoDB.HCC |
| CDKN1A | P38936 | cyclin-dependent kinase inhibitor 1A (p21, Cip1) | Liverome/OncoDB.HCC |
| CLIC1 | O00299 | chloride intracellular channel 1 | Liverome/OncoDB.HCC |
| CPS1 | P31327 | carbamoyl-phosphate synthase 1, mitochondrial | Liverome/OncoDB.HCC |
| CTGF | P29279 | connective tissue growth factor | Liverome/OncoDB.HCC |
| CXCL12 | P48061 | chemokine (C-X-C motif) ligand 12 | Liverome/OncoDB.HCC |
| CYP2E1 | P05181 | cytochrome P450, family 2, subfamily E, polypeptide 1 | Liverome/OncoDB.HCC |
| DLGAP5 | Q15398 | discs, large (Drosophila) homolog-associated protein 5 | Liverome/OncoDB.HCC |
| ETS2 | P15036 | v-ets erythroblastosis virus E26 oncogene homolog 2 (avian) | Liverome/OncoDB.HCC |
| FABP5 | Q01469 | fatty acid binding protein 5 (psoriasis-associated) | Liverome/OncoDB.HCC |
| GLUL | P15104 | glutamate-ammonia ligase | Liverome/OncoDB.HCC |
| GNMT | Q14749 | glycine N-methyltransferase | Liverome/OncoDB.HCC |
| GPC3 | P51654 | glypican 3 | Liverome/OncoDB.HCC |
| HDGF | P51858 | hepatoma-derived growth factor | Liverome/OncoDB.HCC |
| HMGA1 | P17096 | high mobility group AT-hook 1 | Liverome/OncoDB.HCC |
| HMMR | O75330 | hyaluronan-mediated motility receptor (RHAMM) | Liverome/OncoDB.HCC |
| HSP90B1 | P14625 | heat shock protein 90kDa beta (Grp94), member 1 | Liverome/OncoDB.HCC |
| HSPA5 | P11021 | heat shock 70kDa protein 5 (glucose-regulated protein, 78kDa) | Liverome/OncoDB.HCC |
| HSPA8 | P11142 | heat shock 70kDa protein 8 | Liverome/OncoDB.HCC |
| HSPB1 | P04792 | heat shock 27kDa protein 1 | Liverome/OncoDB.HCC |
| IGF2 | P01344 | insulin-like growth factor 2 (somatomedin A) | Liverome/OncoDB.HCC |
| IGF2R | P11717 | insulin-like growth factor 2 receptor | Liverome/OncoDB.HCC |
| IGFBP3 | P17936 | insulin-like growth factor binding protein 3 | Liverome/OncoDB.HCC |
| MAD2L1 | [Q13257](http://www.uniprot.org/entry/Q13257) | MAD2 mitotic arrest deficient-like 1 (yeast) | Liverome/OncoDB.HCC |
| MAT1A | Q00266 | methionine adenosyltransferase I, alpha | Liverome/OncoDB.HCC |
| MDK | P21741 | midkine (neurite growth-promoting factor 2) | Liverome/OncoDB.HCC |
| MMP9 | P14780 | matrix metallopeptidase 9 (gelatinase B, 92kDa gelatinase, 92kDa type IV collagenase) | Liverome/OncoDB.HCC |
| PCNA | P12004 | proliferating cell nuclear antigen | Liverome/OncoDB.HCC |
| PEG10 | Q86TG7 | paternally expressed 10 | Liverome/OncoDB.HCC |
| PLG | P00747 | plasminogen | Liverome/OncoDB.HCC |
| PTTG1 | O95997 | pituitary tumor-transforming 1 | Liverome/OncoDB.HCC |
| RBP4 | P02753 | retinol binding protein 4, plasma | Liverome/OncoDB.HCC |
| RPLP0 | P05388 | ribosomal protein, large, P0 | Liverome/OncoDB.HCC |
| SERPINF2 | P08697 | serpin peptidase inhibitor, clade F (alpha-2 antiplasmin, pigment epithelium derived factor), member 2 | Liverome/OncoDB.HCC |
| SPARC | P09486 | secreted protein, acidic, cysteine-rich (osteonectin) | Liverome/OncoDB.HCC |
| SPARCL1 | Q14515 | SPARC-like 1 (hevin) | Liverome/OncoDB.HCC |
| STMN1 | P16949 | stathmin 1 | Liverome/OncoDB.HCC |
| SULT2A1 | Q06520 | sulfotransferase family, cytosolic, 2A, dehydroepiandrosterone (DHEA)-preferring, member 1 | Liverome/OncoDB.HCC |
| THBS1 | P07996 | thrombospondin 1 | Liverome/OncoDB.HCC |
| TOP2A | P11388 | topoisomerase (DNA) II alpha 170kDa | Liverome/OncoDB.HCC |
| UBD | O15205 | ubiquitin D | Liverome/OncoDB.HCC |
| VTN | P04004 | vitronectin | Liverome/OncoDB.HCC |
| ACAA1 | P09110 | acetyl-CoA acyltransferase 1 | Liverome |
| ACAA2 | P42765 | acetyl-CoA acyltransferase 2 | Liverome |
| ACADSB | P45954 | acyl-CoA dehydrogenase, short/branched chain | Liverome |
| ACADVL | P49748 | acyl-CoA dehydrogenase, very long chain | Liverome |
| ACAT1 | P24752 | acetyl-CoA acetyltransferase 1 | Liverome |
| ACSL1 | P33121 | acyl-CoA synthetase long-chain family member 1 | Liverome |
| ACTB | P60709 | actin, beta | Liverome |
| ACY1 | Q03154 | aminoacylase 1 | Liverome |
| ADH1B | [P00325](http://www.uniprot.org/entry/P00325) | alcohol dehydrogenase 1B (class I), beta polypeptide | Liverome |
| ADH1C | P00326 | alcohol dehydrogenase 1C (class I), gamma polypeptide | Liverome |
| ADH4 | P08319 | alcohol dehydrogenase 4 (class II), pi polypeptide | Liverome |
| ADH6 | P28332 | alcohol dehydrogenase 6 (class V) | Liverome |
| AFP | P02771 | alpha-fetoprotein | Liverome |
| AKR1C2 | P52895 | aldo-keto reductase family 1, member C2 (dihydrodiol dehydrogenase 2; bile acid binding protein; 3-alpha hydroxysteroid dehydrogenase, type III) | Liverome |
| ALAS1 | P13196 | aminolevulinate, delta-, synthase 1 | Liverome |
| ALDH1A1 | P00352 | aldehyde dehydrogenase 1 family, member A1 | Liverome |
| ALDH1B1 | P30837 | aldehyde dehydrogenase 1 family, member B1 | Liverome |
| ALDH1L1 | O75891 | aldehyde dehydrogenase 1 family, member L1 | Liverome |
| ALDH2 | P05091 | aldehyde dehydrogenase 2 family (mitochondrial) | Liverome |
| ALDH3A2 | [P51648](http://www.uniprot.org/entry/P51648) | aldehyde dehydrogenase 3 family, member A2 | Liverome |
| ALDH4A1 | P30038 | aldehyde dehydrogenase 4 family, member A1 | Liverome |
| ALDOA | P04075 | aldolase A, fructose-bisphosphate | Liverome |
| ANXA2 | P07355 | annexin A2 | Liverome |
| AOX1 | Q06278 | aldehyde oxidase 1 | Liverome |
| APOC3 | P02656 | apolipoprotein C-III | Liverome |
| ARID3A | Q99856 | AT rich interactive domain 3A (BRIGHT-like) | Liverome |
| B2M | P61769 | beta-2-microglobulin | Liverome |
| BHMT | Q93088 | betaine--homocysteine S-methyltransferase | Liverome |
| BNIP3 | Q12983 | BCL2/adenovirus E1B 19kDa interacting protein 3 | Liverome |
| BUB1 | O43683 | budding uninhibited by benzimidazoles 1 homolog (yeast) | Liverome |
| C1R | P00736 | complement component 1, r subcomponent | Liverome |
| C5orf13 | Q16612 | chromosome 5 open reading frame 13 | Liverome |
| C6 | P13671 | complement component 6 | Liverome |
| C8A | P07357 | complement component 8, alpha polypeptide | Liverome |
| C9 | P02748 | complement component 9 | Liverome |
| CAPG | P40121 | capping protein (actin filament), gelsolin-like | Liverome |
| CAT | P04040 | catalase | Liverome |
| CBX1 | P83916 | chromobox homolog 1 (HP1 beta homolog Drosophila ) | Liverome |
| CCT5 | P48643 | chaperonin containing TCP1, subunit 5 (epsilon) | Liverome |
| CD14 | P08571 | CD14 molecule | Liverome |
| CD24 | P25063 | CD24 molecule | Liverome |
| CDK4 | P11802 | cyclin-dependent kinase 4 | Liverome |
| CES1 | P23141 | carboxylesterase 1 (monocyte/macrophage serine esterase 1) | Liverome |
| CES2 | O00748 | carboxylesterase 2 (intestine, liver) | Liverome |
| CFH | P08603 | complement factor H | Liverome |
| CLU | P10909 | clusterin | Liverome |
| COL1A1 | P02452 | collagen, type I, alpha 1 | Liverome |
| COL1A2 | P08123 | collagen, type I, alpha 2 | Liverome |
| COMT | P21964 | catechol-O-methyltransferase | Liverome |
| CP | P00450 | ceruloplasmin (ferroxidase) | Liverome |
| CPB2 | Q96IY4 | carboxypeptidase B2 (plasma) | Liverome |
| CRHBP | P24387 | corticotropin releasing hormone binding protein | Liverome |
| CSTB | P04080 | cystatin B (stefin B) | Liverome |
| CTH | P32929 | cystathionase (cystathionine gamma-lyase) | Liverome |
| CTSB | P07858 | cathepsin B | Liverome |
| CYB5A | P00167 | cytochrome b5 type A (microsomal) | Liverome |
| CYP1A2 | P05177 | cytochrome P450, family 1, subfamily A, polypeptide 2 | Liverome |
| CYP27A1 | Q02318 | cytochrome P450, family 27, subfamily A, polypeptide 1 | Liverome |
| CYP2A6 | P11509 | cytochrome P450, family 2, subfamily A, polypeptide 6 | Liverome |
| CYP2A7 | P20853 | cytochrome P450, family 2, subfamily A, polypeptide 7 | Liverome |
| CYP2B6 | [P20813](http://www.uniprot.org/entry/P20813) | cytochrome P450, family 2, subfamily B, polypeptide 6 | Liverome |
| CYP2C8 | P10632 | cytochrome P450, family 2, subfamily C, polypeptide 8 | Liverome |
| CYP2C9 | P11712 | cytochrome P450, family 2, subfamily C, polypeptide 9 | Liverome |
| CYP2J2 | P51589 | cytochrome P450, family 2, subfamily J, polypeptide 2 | Liverome |
| CYP3A4 | P08684 | cytochrome P450, family 3, subfamily A, polypeptide 4 | Liverome |
| CYR61 | O00622 | cysteine-rich, angiogenic inducer, 61 | Liverome |
| DCXR | Q7Z4W1 | dicarbonyl/L-xylulose reductase | Liverome |
| DDR1 | Q08345 | discoidin domain receptor tyrosine kinase 1 | Liverome |
| DNASE1L3 | Q13609 | deoxyribonuclease I-like 3 | Liverome |
| EFNA1 | P20827 | ephrin-A1 | Liverome |
| ENO1 | P06733 | enolase 1, (alpha) | Liverome |
| EPHX1 | P07099 | epoxide hydrolase 1, microsomal (xenobiotic) | Liverome |
| FABP1 | P07148 | fatty acid binding protein 1, liver | Liverome |
| FAH | P16930 | fumarylacetoacetate hydrolase (fumarylacetoacetase) | Liverome |
| FCGRT | P55899 | Fc fragment of IgG, receptor, transporter, alpha | Liverome |
| FCN3 | O75636 | ficolin (collagen/fibrinogen domain containing) 3 (Hakata antigen) | Liverome |
| FEN1 | P39748 | flap structure-specific endonuclease 1 | Liverome |
| FGA | P02671 | fibrinogen alpha chain | Liverome |
| FGB | P02675 | fibrinogen beta chain | Liverome |
| FGG | P02679 | fibrinogen gamma chain | Liverome |
| FGL1 | Q08830 | fibrinogen-like 1 | Liverome |
| FH | P07954 | fumarate hydratase | Liverome |
| FN1 | P02751 | fibronectin 1 | Liverome |
| FTCD | O95954 | formiminotransferase cyclodeaminase | Liverome |
| GAPDH | P04406 | glyceraldehyde-3-phosphate dehydrogenase | Liverome |
| GC | P02774 | group-specific component (vitamin D binding protein) | Liverome |
| GHR | P10912 | growth hormone receptor | Liverome |
| GLUD1 | [P00367](http://www.uniprot.org/entry/P00367) | glutamate dehydrogenase 1 | Liverome |
| GRHPR | Q9UBQ7 | glyoxylate reductase/hydroxypyruvate reductase | Liverome |
| GYS2 | P54840 | glycogen synthase 2 (liver) | Liverome |
| HAAO | P46952 | 3-hydroxyanthranilate 3,4-dioxygenase | Liverome |
| HAMP | P81172 | hepcidin antimicrobial peptide | Liverome |
| HAO1 | Q9UJM8 | hydroxyacid oxidase (glycolate oxidase) 1 | Liverome |
| HGD | Q93099 | homogentisate 1,2-dioxygenase | Liverome |
| HGFAC | Q04756 | HGF activator | Liverome |
| HMGCS2 | P54868 | 3-hydroxy-3-methylglutaryl-CoA synthase 2 (mitochondrial) | Liverome |
| HNRNPA2B1 | P22626 | heterogeneous nuclear ribonucleoprotein A2/B1 | Liverome |
| HNRNPC | P07910 | heterogeneous nuclear ribonucleoprotein C (C1/C2) | Liverome |
| HPD | P32754 | 4-hydroxyphenylpyruvate dioxygenase | Liverome |
| HPX | [P02790](http://www.uniprot.org/entry/P02790) | hemopexin | Liverome |
| HRSP12 | P52758 | heat-responsive protein 12 | Liverome |
| HSD17B10 | Q99714 | hydroxysteroid (17-beta) dehydrogenase 10 | Liverome |
| HSD17B4 | P51659 | hydroxysteroid (17-beta) dehydrogenase 4 | Liverome |
| HSD17B6 | [O14756](http://www.uniprot.org/entry/O14756) | hydroxysteroid (17-beta) dehydrogenase 6 homolog (mouse) | Liverome |
| HSPA1B | P0DMV8 | heat shock 70kDa protein 1B | Liverome |
| IFI27 | P40305 | interferon, alpha-inducible protein 27 | Liverome |
| IFIT1 | P09914 | interferon-induced protein with tetratricopeptide repeats 1 | Liverome |
| IGKC | P01601 | immunoglobulin kappa constant | Liverome |
| ILF2 | Q12905 | interleukin enhancer binding factor 2, 45kDa | Liverome |
| ISG15 | P05161 | ISG15 ubiquitin-like modifier | Liverome |
| ITIH2 | P19823 | inter-alpha (globulin) inhibitor H2 | Liverome |
| ITIH4 | Q14624 | inter-alpha (globulin) inhibitor H4 (plasma Kallikrein-sensitive glycoprotein) | Liverome |
| ITPR2 | Q14571 | inositol 1,4,5-triphosphate receptor, type 2 | Liverome |
| KHK | P50053 | ketohexokinase (fructokinase) | Liverome |
| KIF23 | Q02241 | kinesin family member 23 | Liverome |
| KNG1 | P01042 | kininogen 1 | Liverome |
| KRT8 | P05787 | keratin 8 | Liverome |
| LAMP2 | P13473 | lysosomal-associated membrane protein 2 | Liverome |
| LPGAT1 | Q92604 | lysophosphatidylglycerol acyltransferase 1 | Liverome |
| LYZ | P61626 | lysozyme | Liverome |
| MCM2 | P49736 | minichromosome maintenance complex component 2 | Liverome |
| MCM6 | Q14566 | minichromosome maintenance complex component 6 | Liverome |
| MT1F | P04733 | metallothionein 1F | Liverome |
| MT2A | P02795 | metallothionein 2A | Liverome |
| MTHFD1 | P11586 | methylenetetrahydrofolate dehydrogenase (NADP+ dependent) 1, methenyltetrahydrofolate cyclohydrolase, formyltetrahydrofolate synthetase | Liverome |
| NAMPT | P43490 | nicotinamide phosphoribosyltransferase | Liverome |
| NDRG1 | Q92597 | N-myc downstream regulated 1 | Liverome |
| NFKBIA | P25963 | nuclear factor of kappa light polypeptide gene enhancer in B-cells inhibitor, alpha | Liverome |
| NME1 | P15531 | non-metastatic cells 1, protein (NM23A) expressed in | Liverome |
| NNMT | P40261 | nicotinamide N-methyltransferase | Liverome |
| NPM1 | P06748 | nucleophosmin (nucleolar phosphoprotein B23, numatrin) | Liverome |
| PC | P11498 | pyruvate carboxylase | Liverome |
| PCK1 | P35558 | phosphoenolpyruvate carboxykinase 1 (soluble) | Liverome |
| PCK2 | Q16822 | phosphoenolpyruvate carboxykinase 2 (mitochondrial) | Liverome |
| PCSK6 | P29122 | proprotein convertase subtilisin/kexin type 6 | Liverome |
| PDIA3 | P30101 | protein disulfide isomerase family A, member 3 | Liverome |
| PEMT | Q9UBM1 | phosphatidylethanolamine N-methyltransferase | Liverome |
| PGK1 | P00558 | phosphoglycerate kinase 1 | Liverome |
| PGM1 | P36871 | phosphoglucomutase 1 | Liverome |
| PGRMC1 | O00264 | progesterone receptor membrane component 1 | Liverome |
| PKM2 | P14618 | pyruvate kinase, muscle | Liverome |
| PON3 | Q15166 | paraoxonase 3 | Liverome |
| PPIB | P23284 | peptidylprolyl isomerase B (cyclophilin B) | Liverome |
| PRDX1 | Q06830 | peroxiredoxin 1 | Liverome |
| PRDX6 | P30041 | peroxiredoxin 6 | Liverome |
| PROZ | P22891 | protein Z, vitamin K-dependent plasma glycoprotein | Liverome |
| PUF60 | Q9UHX1 | poly-U binding splicing factor 60KDa | Liverome |
| PYGB | P11216 | phosphorylase, glycogen; brain | Liverome |
| QDPR | P09417 | quinoid dihydropteridine reductase | Liverome |
| RBP1 | P09455 | retinol binding protein 1, cellular | Liverome |
| RCAN1 | P53805 | regulator of calcineurin 1 | Liverome |
| RDBP | P18615 | RD RNA binding protein | Liverome |
| RFC4 | P35249 | replication factor C (activator 1) 4, 37kDa | Liverome |
| RGN | Q15493 | regucalcin (senescence marker protein-30) | Liverome |
| RHOA | P61586 | ras homolog gene family, member A | Liverome |
| RHOB | P62745 | ras homolog gene family, member B | Liverome |
| RND3 | P61587 | Rho family GTPase 3 | Liverome |
| RPS5 | P46782 | ribosomal protein S5 | Liverome |
| RPSA | P08865 | ribosomal protein SA | Liverome |
| S100A10 | P60903 | S100 calcium binding protein A10 | Liverome |
| S100A6 | P06703 | S100 calcium binding protein A6 | Liverome |
| SAA2 | P0DJI9 | serum amyloid A2 | Liverome |
| SCAMP3 | O14828 | secretory carrier membrane protein 3 | Liverome |
| SCP2 | P22307 | sterol carrier protein 2 | Liverome |
| SELENBP1 | Q13228 | selenium binding protein 1 | Liverome |
| SERPINA1 | P01009 | serpin peptidase inhibitor, clade A (alpha-1 antiproteinase, antitrypsin), member 1 | Liverome |
| SERPINC1 | P01008 | serpin peptidase inhibitor, clade C (antithrombin), member 1 | Liverome |
| SERPING1 | P05155 | serpin peptidase inhibitor, clade G (C1 inhibitor), member 1 | Liverome |
| SGK1 | O00141 | serum/glucocorticoid regulated kinase 1 | Liverome |
| SLC16A2 | P36021 | solute carrier family 16, member 2 (monocarboxylic acid transporter 8) | Liverome |
| SLC22A1 | O15245 | solute carrier family 22 (organic cation transporter), member 1 | Liverome |
| SLC2A2 | P11168 | solute carrier family 2 (facilitated glucose transporter), member 2 | Liverome |
| SLC7A2 | P52569 | solute carrier family 7 (cationic amino acid transporter, y+ system), member 2 | Liverome |
| SNRPE | P62304 | small nuclear ribonucleoprotein polypeptide E | Liverome |
| SOD1 | P00441 | superoxide dismutase 1, soluble | Liverome |
| SOD2 | P04179 | superoxide dismutase 2, mitochondrial | Liverome |
| SRGN | P10124 | serglycin | Liverome |
| STAT1 | P42224 | signal transducer and activator of transcription 1, 91kDa | Liverome |
| TDO2 | P48775 | tryptophan 2,3-dioxygenase | Liverome |
| TF | P02787 | transferrin | Liverome |
| TGM2 | P21980 | transglutaminase 2 (C polypeptide, protein-glutamine-gamma-glutamyltransferase) | Liverome |
| TIMP1 | P01033 | TIMP metallopeptidase inhibitor 1 | Liverome |
| TMED2 | Q15363 | transmembrane emp24 domain trafficking protein 2 | Liverome |
| TPM2 | P07951 | tropomyosin 2 (beta) | Liverome |
| TST | Q16762 | thiosulfate sulfurtransferase (rhodanese) | Liverome |
| TTR | P02766 | transthyretin | Liverome |
| TUBB | P07437 | tubulin, beta | Liverome |
| UGT2B7 | P16662 | UDP glucuronosyltransferase 2 family, polypeptide B7 | Liverome |
| VIM | P08670 | vimentin | Liverome |
| ABCB1 | P08183 | Multidrug resistance protein 1 | OncoDB.HCC |
| ABCG2 | Q9UNQ0 | ATP-binding cassette sub-family G member 2 | OncoDB.HCC |
| ACAT2 | Q9BWD1 | Acetyl-CoA acetyltransferase, cytosolic | OncoDB.HCC |
| ACP5 | P13686 | Tartrate-resistant acid phosphatase type 5 precursor | OncoDB.HCC |
| ADAM17 | P78536 | ADAM 17 precursor | OncoDB.HCC |
| AFM | P43652 | Afamin precursor | OncoDB.HCC |
| AKAP13 | Q12802 | LBC oncogene | OncoDB.HCC |
| AKT2 | P31751 | RAC-beta serine/threonine-protein kinase | OncoDB.HCC |
| ANGPT1 | Q15389 | Eukaryotic translation initiation factor 3 subunit 6 | OncoDB.HCC |
| ANGPT2 | O15123 | Angiopoietin-2 precursor | OncoDB.HCC |
| ANXA10 | Q9UJ72 | Annexin A10 | OncoDB.HCC |
| APC | P25054 | Adenomatous polyposis coli protein | OncoDB.HCC |
| APOE | P02649 | Apolipoprotein E precursor | OncoDB.HCC |
| AR | P10275 | Androgen receptor | OncoDB.HCC |
| ARRDC4 | Q8NCT1 | Arrestin domain-containing protein 4. | OncoDB.HCC |
| ASPH | Q12797 | Aspartyl/asparaginyl beta-hydroxylase | OncoDB.HCC |
| [ATF6](http://oncodb.hcc.ibms.sinica.edu.tw/hcc/display_by_gene.cgi?stable_id=ENSG00000118217&cut_off=1&option=simple) | P18850 | Cyclic AMP-dependent transcription factor ATF-6 alpha | OncoDB.HCC |
| AURKA | O14965 | Serine/threonine-protein kinase 6 | OncoDB.HCC |
| AXIN1 | O15169 | Axin-1 | OncoDB.HCC |
| AXIN2 | Q9Y2T1 | Axin-2 | OncoDB.HCC |
| BAD | Q92934 | Bcl2 antagonist of cell death | OncoDB.HCC |
| BAGE5 | Q86Y29 | B melanoma antigen 3 precursor | OncoDB.HCC |
| BAX | Q07814 | Apoptosis regulator BAX, cytoplasmic isoform beta. | OncoDB.HCC |
| BCL10 | O95999 | B-cell lymphoma/leukemia 10 | OncoDB.HCC |
| BIRC5 | O15392 | Baculoviral IAP repeat-containing protein 5 | OncoDB.HCC |
| CA1 | P00915 | Carbonic anhydrase 1 | OncoDB.HCC |
| CA3 | P07451 | Carbonic anhydrase 3 | OncoDB.HCC |
| CASP1 | P29466 | Caspase-1 precursor | OncoDB.HCC |
| CASP3 | P42574 | Caspase-3 precursor | OncoDB.HCC |
| CBS | P35520 | Cystathionine beta-synthase | OncoDB.HCC |
| CCNA2 | P20248 | Cyclin-A2 | OncoDB.HCC |
| CCND1 | P24385 | G1/S-specific cyclin-D1 | OncoDB.HCC |
| CCNE1 | P24864 | G1/S-specific cyclin-E1 | OncoDB.HCC |
| CD163 | Q86VB7 | Scavenger receptor cysteine-rich type 1 protein M130 precursor | OncoDB.HCC |
| CD302 | Q8IX05 | CD302 antigen precursor | OncoDB.HCC |
| CD40 | P25942 | Tumor necrosis factor receptor superfamily member 5 precursor | OncoDB.HCC |
| CD44 | P16070 | CD44 antigen precursor | OncoDB.HCC |
| CD81 | P60033 | CD81 antigen | OncoDB.HCC |
| CD82 | P27701 | CD82 antigen | OncoDB.HCC |
| CD9 | P21926 | CD9 antigen | OncoDB.HCC |
| CD99 | P14209 | CD99 antigen precursor | OncoDB.HCC |
| CDC25A | P30304 | M-phase inducer phosphatase 1 | OncoDB.HCC |
| CDC25B | P30305 | M-phase inducer phosphatase 2 | OncoDB.HCC |
| CDC34 | P49427 | Ubiquitin-conjugating enzyme E2-32 kDa complementing | OncoDB.HCC |
| CDH1 | P12830 | Epithelial-cadherin precursor | OncoDB.HCC |
| CDH17 | Q12864 | Cadherin-17 precursor | OncoDB.HCC |
| CDKN1B | P46527 | Cyclin-dependent kinase inhibitor 1B | OncoDB.HCC |
| CDKN1C | P49918 | Cyclin-dependent kinase inhibitor 1C | OncoDB.HCC |
| CDKN2A | P42771 | Cyclin-dependent kinase inhibitor 2A | OncoDB.HCC |
| CDKN2B | P42772 | Cyclin-dependent kinase 4 inhibitor B | OncoDB.HCC |
| CDKN2C | P42773 | Cyclin-dependent kinase 6 inhibitor | OncoDB.HCC |
| CENTB1 | Q15027 | Centaurin-beta 1 | OncoDB.HCC |
| CIB2 | O75838 | Calcium and integrin-binding protein 2 | OncoDB.HCC |
| CLDN4 | O14493 | Claudin-4 | OncoDB.HCC |
| COL7A1 | Q02388 | Collagen alpha-1(VII) chain precursor | OncoDB.HCC |
| COPA | P53621 | Coatomer subunit alpha | OncoDB.HCC |
| COPS5 | Q92905 | COP9 signalosome complex subunit 5 | OncoDB.HCC |
| CREB3L3 | Q68CJ9 | cAMP responsive element binding protein 3-like 3 | OncoDB.HCC |
| CRYL1 | Q9Y2S2 | Lambda-crystallin homolog. | OncoDB.HCC |
| CSDA | P16989 | DNA-binding protein A | OncoDB.HCC |
| CTAG1A | P78358 | Cancer/testis antigen 1B | OncoDB.HCC |
| CTNNB1 | P35222 | Catenin beta-1 | OncoDB.HCC |
| CXCL2 | P19875 | Macrophage inflammatory protein 2-alpha precursor | OncoDB.HCC |
| DACT1 | Q9NYF0 | Dapper homolog 1 | OncoDB.HCC |
| DAD1 | P61803 | Dolichyl-diphosphooligosaccharide--protein glycosyltransferase subunit DAD1 | OncoDB.HCC |
| DCC | P43146 | Netrin receptor DCC precursor | OncoDB.HCC |
| DCN | P07585 | Decorin precursor | OncoDB.HCC |
| DDX3X | O00571 | ATP-dependent RNA helicase DDX3X | OncoDB.HCC |
| [DEK](http://oncodb.hcc.ibms.sinica.edu.tw/hcc/display_by_gene.cgi?stable_id=ENSG00000124795&cut_off=1&option=simple) | P35659 | Protein DEK | OncoDB.HCC |
| DERL2 | Q9GZP9 | Derlin-2 | OncoDB.HCC |
| DLC1 | Q96QB1 | Rho-GTPase-activating protein 7 | OncoDB.HCC |
| DNMT1 | P26358 | DNA (cytosine-5)-methyltransferase 1 | OncoDB.HCC |
| [DNMT3A](http://oncodb.hcc.ibms.sinica.edu.tw/hcc/display_by_gene.cgi?stable_id=ENSG00000119772&cut_off=1&option=simple) | Q9Y6K1 | DNA (cytosine-5)-methyltransferase 3A | OncoDB.HCC |
| DNMT3B | Q9UBC3 | DNA (cytosine-5)-methyltransferase 3B | OncoDB.HCC |
| DOCK4 | Q8N1I0 | Dedicator of cytokinesis protein 4 | OncoDB.HCC |
| DUSP1 | P28562 | Dual specificity protein phosphatase 1 | OncoDB.HCC |
| DYNLL2 | Q96FJ2 | Dynein light chain 2, cytoplasmic | OncoDB.HCC |
| E2F1 | Q01094 | Transcription factor E2F1 | OncoDB.HCC |
| EBAG9 | O00559 | Receptor-binding cancer antigen expressed on SiSo cells | OncoDB.HCC |
| ECGF1 | P19971 | Thymidine phosphorylase precursor | OncoDB.HCC |
| EDN1 | P05305 | Endothelin-1 precursor | OncoDB.HCC |
| EDNRB | P24530 | Endothelin B receptor precursor | OncoDB.HCC |
| EFNA1 | P20827 | Ephrin-A1 precursor | OncoDB.HCC |
| EFNB1 | P98172 | Ephrin-B1 precursor | OncoDB.HCC |
| EGF | P01133 | Pro-epidermal growth factor precursor | OncoDB.HCC |
| EGFR | P00533 | Epidermal growth factor receptor precursor | OncoDB.HCC |
| EIF3S2 | Q13347 | Eukaryotic translation initiation factor 3 subunit 2 | OncoDB.HCC |
| EIF3S3 | O15372 | Eukaryotic translation initiation factor 3 subunit 3 | OncoDB.HCC |
| ENG | P17813 | Endoglin precursor | OncoDB.HCC |
| ENPP2 | Q13822 | Ectonucleotide pyrophosphatase/phosphodiesterase 2 | OncoDB.HCC |
| ERBB2 | P04626 | Receptor tyrosine-protein kinase erbB-2 precursor | OncoDB.HCC |
| ERBB3 | P21860 | Receptor tyrosine-protein kinase erbB-3 precursor | OncoDB.HCC |
| ERBB4 | Q15303 | Receptor tyrosine-protein kinase erbB-4 precursor | OncoDB.HCC |
| ERCC1 | P07992 | DNA excision repair protein ERCC-1. | OncoDB.HCC |
| ESR1 | P03372 | Estrogen receptor | OncoDB.HCC |
| ETHE1 | O95571 | ETHE1 protein, mitochondrial precursor | OncoDB.HCC |
| ETV4 | P43268 | ETS translocation variant 4 | OncoDB.HCC |
| EZH2 | Q15910 | Enhancer of zeste homolog 2 | OncoDB.HCC |
| F9 | P00740 | Coagulation factor IX precursor | OncoDB.HCC |
| FADD | Q13158 | FAS-associating death domain-containing protein | OncoDB.HCC |
| FAM10A6 | P50502 | Hsc70-interacting protein | OncoDB.HCC |
| FAS | P25445 | Tumor necrosis factor receptor superfamily member 6 precursor | OncoDB.HCC |
| FATE1 | Q969F0 | Fetal and adult testis-expressed transcript protein | OncoDB.HCC |
| FGF1 | P05230 | Heparin-binding growth factor 1 precursor | OncoDB.HCC |
| FGF2 | P09038 | Heparin-binding growth factor 2 precursor | OncoDB.HCC |
| FGFR3 | P22607 | Fibroblast growth factor receptor 3 precursor | OncoDB.HCC |
| FHIT | P49789 | Bis(5'-adenosyl)-triphosphatase | OncoDB.HCC |
| FKBP4 | Q02790 | FK506-binding protein 4 | OncoDB.HCC |
| FOS | P01100 | Proto-oncogene protein c-fos | OncoDB.HCC |
| FTL | P02792 | Ferritin light chain | OncoDB.HCC |
| FUT4 | P22083 | Alpha-(1,3)-fucosyltransferase | OncoDB.HCC |
| GABRE | P78334 | Gamma-aminobutyric-acid receptor subunit epsilon precursor | OncoDB.HCC |
| GADD45B | O75293 | Growth arrest and DNA-damage-inducible protein GADD45 beta | OncoDB.HCC |
| GADD45G | O95257 | Growth arrest and DNA-damage-inducible protein GADD45 gamma | OncoDB.HCC |
| GAGE8 | Q13065 | G antigen 1 | OncoDB.HCC |
| GFER | P55789 | Augmenter of liver regeneration | OncoDB.HCC |
| GJB1 | P08034 | Gap junction beta-1 protein | OncoDB.HCC |
| [GJB2](http://oncodb.hcc.ibms.sinica.edu.tw/hcc/display_by_gene.cgi?stable_id=ENSG00000165474&cut_off=1&option=simple) | P29033 | Gap junction beta-2 protein | OncoDB.HCC |
| GLI1 | P08151 | Zinc finger protein GLI1 | OncoDB.HCC |
| GNAI1 | P63096 | Guanine nucleotide-binding protein G(i), alpha-1 subunit | OncoDB.HCC |
| GNAI3 | P08754 | Guanine nucleotide-binding protein G(k) subunit alpha (G(i) alpha-3) | OncoDB.HCC |
| GNAS | Q5JWF2 | Guanine nucleotide-binding protein G(s) subunit alpha isoforms XLas | OncoDB.HCC |
| GPAA1 | O43292 | Glycosylphosphatidylinositol anchor attachment 1 protein | OncoDB.HCC |
| GPR75 | O95800 | Probable G-protein coupled receptor 75 | OncoDB.HCC |
| GPR78 | Q96P69 | Probable G-protein coupled receptor 78 | OncoDB.HCC |
| GRN | P28799 | Granulins precursor | OncoDB.HCC |
| GSK3A | P49840 | Glycogen synthase kinase-3 alpha | OncoDB.HCC |
| GSTP1 | P09211 | Glutathione S-transferase P | OncoDB.HCC |
| H19 | NR_002196 | H19, imprinted maternally expressed untranslated mRNA | OncoDB.HCC |
| HAS2 | Q92819 | Hyaluronan synthase 2 | OncoDB.HCC |
| HBEGF | Q99075 | Heparin-binding EGF-like growth factor precursor | OncoDB.HCC |
| HFE | Q30201 | Hereditary hemochromatosis protein precursor | OncoDB.HCC |
| HGF | P14210 | Hepatocyte growth factor precursor (Scatter factor) | OncoDB.HCC |
| HOXA13 | P31271 | Homeobox protein Hox-A13 | OncoDB.HCC |
| HP | P00738 | Haptoglobin precursor | OncoDB.HCC |
| HPSE | Q9Y251 | Heparanase precursor | OncoDB.HCC |
| HSP90AA1 | P07900 | Heat shock protein HSP 90-alpha | OncoDB.HCC |
| HSPA1B | P08107 | Heat shock 70 kDa protein 1 | OncoDB.HCC |
| HSPA4 | P34932 | Heat shock 70 kDa protein 4 | OncoDB.HCC |
| HSPH1 | Q92598 | Heat-shock protein 105 kDa | OncoDB.HCC |
| ICAM1 | P05362 | Intercellular adhesion molecule 1 precursor | OncoDB.HCC |
| ID1 | P41134 | DNA-binding protein inhibitor ID-1 | OncoDB.HCC |
| IGF1 | P01343 | Insulin-like growth factor IA precursor | OncoDB.HCC |
| IGFBP1 | P08833 | Insulin-like growth factor-binding protein 3 precursor | OncoDB.HCC |
| IGLL1 | P15814 | Immunoglobulin lambda-like polypeptide 1 precursor | OncoDB.HCC |
| IGLV4-3 | P01842 | Ig lambda chain C regions. | OncoDB.HCC |
| IL12RB2 | Q99665 | Interleukin-12 receptor beta-2 chain precursor | OncoDB.HCC |
| IL18RAP | O95256 | Interleukin-18 receptor accessory protein precursor | OncoDB.HCC |
| IL2 | P60568 | Interleukin-2 precursor | OncoDB.HCC |
| IRF1 | P10914 | Interferon regulatory factor 1 | OncoDB.HCC |
| ITGA6 | P23229 | Integrin alpha-6 precursor | OncoDB.HCC |
| [ITGB1](http://oncodb.hcc.ibms.sinica.edu.tw/hcc/display_by_gene.cgi?stable_id=ENSG00000150093&cut_off=1&option=simple) | P05556 | Integrin beta-1 precursor | OncoDB.HCC |
| JTB | O76095 | Protein JTB precursor | OncoDB.HCC |
| JUNB | P17275 | Transcription factor jun-B. | OncoDB.HCC |
| KHDRBS1 | Q07666 | KH domain-containing, RNA-binding, signal transduction-associated protein 1 | OncoDB.HCC |
| KIAA0101 | Q15004 | PCNA-associated factor | OncoDB.HCC |
| [KISS1](http://oncodb.hcc.ibms.sinica.edu.tw/hcc/display_by_gene.cgi?stable_id=ENSG00000170498&cut_off=1&option=simple) | Q15726 | Metastasis-suppressor KiSS-1 precursor | OncoDB.HCC |
| KISS1R | Q969F8 | KiSS-1 receptor | OncoDB.HCC |
| KLF6 | Q99612 | Krueppel-like factor 6 | OncoDB.HCC |
| KLKB1 | P03952 | Plasma kallikrein precursor | OncoDB.HCC |
| KRAS | P01116 | GTPase KRas | OncoDB.HCC |
| KRT19 | P08727 | Keratin, type I cytoskeletal 19 | OncoDB.HCC |
| LAPTM4B | Q86VI4 | Lysosomal-associated transmembrane protein 4B | OncoDB.HCC |
| LCN2 | P80188 | Neutrophil gelatinase-associated lipocalin precursor | OncoDB.HCC |
| LEP | P41159 | Leptin precursor | OncoDB.HCC |
| LEPR | P48357 | Leptin receptor precursor | OncoDB.HCC |
| LETMD1 | Q6P1Q0 | LETM1 domain containing 1 isoform 1 | OncoDB.HCC |
| LGALS3 | P17931 | Galectin-3 | OncoDB.HCC |
| LGALS4 | P56470 | Galectin-4 | OncoDB.HCC |
| LGR5 | O75473 | Leucine-rich repeat-containing G-protein coupled receptor 5 precursor | OncoDB.HCC |
| LUZP4 | Q9P127 | leucine zipper protein 4 | OncoDB.HCC |
| LY6E | Q16553 | Lymphocyte antigen Ly-6E precursor | OncoDB.HCC |
| MAGEA1 | P43355 | Melanoma-associated antigen 1 | OncoDB.HCC |
| MAGEA10 | P43363 | Melanoma-associated antigen 10 | OncoDB.HCC |
| MAGEA11 | P43364 | Melanoma-associated antigen 11 | OncoDB.HCC |
| MAGEA2B | P43356 | Melanoma-associated antigen 2 | OncoDB.HCC |
| MAGEA3 | P43357 | Melanoma-associated antigen 3 | OncoDB.HCC |
| MAGEA4 | P43358 | Melanoma-associated antigen 4 | OncoDB.HCC |
| MAGEA8 | P43361 | Melanoma-associated antigen 8 | OncoDB.HCC |
| MAP2K1 | Q02750 | Dual specificity mitogen-activated protein kinase kinase 1 | OncoDB.HCC |
| MAP2K2 | P36507 | Dual specificity mitogen-activated protein kinase kinase 2 | OncoDB.HCC |
| MAPK1 | P28482 | Mitogen-activated protein kinase 1 | OncoDB.HCC |
| MAPK3 | P27361 | Mitogen-activated protein kinase 3 | OncoDB.HCC |
| MAPRE1 | Q15691 | Microtubule-associated protein RP/EB family member 1 | OncoDB.HCC |
| MAT2A | P31153 | S-adenosylmethionine synthetase isoform type-2 | OncoDB.HCC |
| MCL1 | Q07820 | Induced myeloid leukemia cell differentiation protein Mcl-1 | OncoDB.HCC |
| MCM7 | P33993 | DNA replication licensing factor MCM7 | OncoDB.HCC |
| MET | P08581 | Hepatocyte growth factor receptor precursor | OncoDB.HCC |
| MGMT | P16455 | Methylated-DNA--protein-cysteine methyltransferase | OncoDB.HCC |
| MIF | P14174 | Macrophage migration inhibitory factor | OncoDB.HCC |
| MKI67 | P46013 | Antigen KI-67 | OncoDB.HCC |
| MLC1 | Q15049 | Membrane protein MLC1 | OncoDB.HCC |
| MME | P08473 | Neprilysin | OncoDB.HCC |
| MMP12 | P39900 | Macrophage metalloelastase precursor | OncoDB.HCC |
| MMP14 | P50281 | Matrix metalloproteinase-14 precursor | OncoDB.HCC |
| MMP2 | P08253 | 72 kDa type IV collagenase precursor | OncoDB.HCC |
| MMP3 | P08254 | Stromelysin-1 precursor | OncoDB.HCC |
| MMP7 | P09237 | Matrilysin precursor | OncoDB.HCC |
| MTA1 | Q13330 | Metastasis-associated protein MTA1. | OncoDB.HCC |
| MTAP | Q13126 | S-methyl-5-thioadenosine phosphorylase | OncoDB.HCC |
| MTR | Q99707 | Methionine synthase | OncoDB.HCC |
| MTSS1 | O43312 | Metastasis suppressor protein 1 | OncoDB.HCC |
| MTUS1 | NP_001001927 | mitochondrial tumor suppressor 1 isoform 3 | OncoDB.HCC |
| MUC1 | P15941 | Mucin-1 precursor | OncoDB.HCC |
| MUTED | Q8NBS9 | Thioredoxin domain-containing protein 5 precursor | OncoDB.HCC |
| MYBL2 | P10244 | Myb-related protein B | OncoDB.HCC |
| MYC | P01106 | Myc proto-oncogene protein | OncoDB.HCC |
| NCSTN | Q92542 | Nicastrin precursor | OncoDB.HCC |
| NEK6 | Q9HC98 | Serine/threonine-protein kinase Nek6 | OncoDB.HCC |
| NME2 | P22392 | Nucleoside diphosphate kinase B | OncoDB.HCC |
| NOS2A | P35228 | Nitric oxide synthase, inducible | OncoDB.HCC |
| NP_001032647.1 | NP_001032647 | associated with liver cancer | OncoDB.HCC |
| NP_114111.2 | Q8N2F6 | SVH protein | OncoDB.HCC |
| NP_689935.1 | NP_689935 | hepatocyte cell adhesion molecule | OncoDB.HCC |
| NRG1 | Q15491 | Neuregulin-1, sensory and motor neuron-derived factor isoform | OncoDB.HCC |
| NRG2 | O14511 | Pro-neuregulin-2, membrane-bound isoform precursor | OncoDB.HCC |
| NRG3 | P56975 | Pro-neuregulin-3, membrane-bound isoform precursor | OncoDB.HCC |
| OAT | P04181 | Ornithine aminotransferase, mitochondrial precursor | OncoDB.HCC |
| [PARK2](http://oncodb.hcc.ibms.sinica.edu.tw/hcc/display_by_gene.cgi?stable_id=ENSG00000185345&cut_off=1&option=simple) | O60260 | Parkin | OncoDB.HCC |
| PARP1 | P09874 | Poly [ADP-ribose] polymerase 1 | OncoDB.HCC |
| PDGFRA | P16234 | Alpha platelet-derived growth factor receptor precursor | OncoDB.HCC |
| PGC | P20142 | Gastricsin precursor | OncoDB.HCC |
| PIK3CA | P42336 | Phosphatidylinositol-4,5-bisphosphate 3-kinase catalytic subunit alpha isoform | OncoDB.HCC |
| PIN1 | Q13526 | Peptidyl-prolyl cis-trans isomerase NIMA-interacting 1 | OncoDB.HCC |
| PLAU | P00749 | Urokinase-type plasminogen activator precursor | OncoDB.HCC |
| PLK1 | P53350 | Serine/threonine-protein kinase PLK1 | OncoDB.HCC |
| PPIA_HUMAN | P62937 | Peptidyl-prolyl cis-trans isomerase A | OncoDB.HCC |
| PRDX2 | P32119 | Peroxiredoxin-2 | OncoDB.HCC |
| PSMD10 | O75832 | 26S proteasome non-ATPase regulatory subunit 10 | OncoDB.HCC |
| PSMD4 | P55036 | 26S proteasome non-ATPase regulatory subunit 4 | OncoDB.HCC |
| PTCH1 | Q13635 | Protein patched homolog 1 | OncoDB.HCC |
| PTENP1 | P60484 | Phosphatidylinositol-3,4,5-trisphosphate 3-phosphatase and dual- specificity protein phosphatase PTEN | OncoDB.HCC |
| PTGS2 | P35354 | Prostaglandin G/H synthase 2 precursor | OncoDB.HCC |
| PTK2 | Q05397 | Focal adhesion kinase 1 | OncoDB.HCC |
| PTPN13 | Q12923 | Tyrosine-protein phosphatase non-receptor type 13 | OncoDB.HCC |
| PYDC1 | Q8WXC3 | pyrin domain containing 1 | OncoDB.HCC |
| RAF1 | P04049 | RAF proto-oncogene serine/threonine-protein kinase | OncoDB.HCC |
| [RARA](http://oncodb.hcc.ibms.sinica.edu.tw/hcc/display_by_gene.cgi?stable_id=ENSG00000131759&cut_off=1&option=simple) | P10276 | Retinoic acid receptor alpha | OncoDB.HCC |
| RASSF1 | Q9NS23 | Ras association domain-containing protein 1 | OncoDB.HCC |
| RB1 | P06400 | Retinoblastoma-associated protein | OncoDB.HCC |
| RBL2 | Q08999 | Retinoblastoma-like protein 2 | OncoDB.HCC |
| RBMY1B | Q15414 | RNA-binding motif protein, Y chromosome, family 1 member A1 | OncoDB.HCC |
| REG1A | P05451 | Lithostathine 1 alpha precursor | OncoDB.HCC |
| REG3A | Q06141 | Regenerating islet-derived protein 3 alpha precursor | OncoDB.HCC |
| RET | P07949 | Proto-oncogene tyrosine-protein kinase receptor ret precursor | OncoDB.HCC |
| [RGS1](http://oncodb.hcc.ibms.sinica.edu.tw/hcc/display_by_gene.cgi?stable_id=ENSG00000090104&cut_off=1&option=simple) | Q08116 | Regulator of G-protein signaling 1 | OncoDB.HCC |
| RHOC | P08134 | Rho-related GTP-binding protein RhoC precursor (H9) | OncoDB.HCC |
| RLN1 | P04808 | Prorelaxin H1 precursor | OncoDB.HCC |
| ROBO1 | Q9Y6N7 | Roundabout homolog 1 precursor | OncoDB.HCC |
| RPL36AL | Q969Q0 | 60S ribosomal protein L36a-like. | OncoDB.HCC |
| RTN4 | Q9NQC3 | Reticulon-4 (Neurite outgrowth inhibitor) | OncoDB.HCC |
| RUNX3 | Q13761 | Runt-related transcription factor 3 | OncoDB.HCC |
| S100A9 | P06702 | Protein S100-A9 (S100 calcium-binding protein A9) | OncoDB.HCC |
| SAFB | Q15424 | Scaffold attachment factor B | OncoDB.HCC |
| SEMA3B | Q13214 | Semaphorin-3B precursor | OncoDB.HCC |
| SERPINB2 | P05120 | Plasminogen activator inhibitor 2 precursor | OncoDB.HCC |
| SERPINE1 | P05121 | Plasminogen activator inhibitor 1 precursor | OncoDB.HCC |
| SFN | P31947 | 14-3-3 protein sigma | OncoDB.HCC |
| SGPP1 | Q9BX95 | Sphingosine-1-phosphate phosphatase 1 | OncoDB.HCC |
| [SHC1](http://oncodb.hcc.ibms.sinica.edu.tw/hcc/display_by_gene.cgi?stable_id=ENSG00000160691&cut_off=1&option=simple) | P29353 | SHC-transforming protein 1 | OncoDB.HCC |
| SHH | Q15465 | Sonic hedgehog protein precursor | OncoDB.HCC |
| SIAH1 | Q8IUQ4 | Ubiquitin ligase SIAH1 | OncoDB.HCC |
| SLC10A1 | Q14973 | Sodium/bile acid cotransporter | OncoDB.HCC |
| SLC22A18 | Q96BI1 | Organic cation transporter-like protein 2 | OncoDB.HCC |
| SLC9A3R1 | O14745 | Ezrin-radixin-moesin-binding phosphoprotein 50 | OncoDB.HCC |
| SLCO1B1 | Q9Y6L6 | Solute carrier organic anion transporter family member 1B1 | OncoDB.HCC |
| SMAD2 | Q13485 | Mothers against decapentaplegic homolog 4 | OncoDB.HCC |
| SMO | Q99835 | Smoothened homolog precursor | OncoDB.HCC |
| SNAI1 | O95863 | Zinc finger protein SNAI1 | OncoDB.HCC |
| SNAI2 | O43623 | Zinc finger protein SLUG | OncoDB.HCC |
| SNCG | O76070 | Gamma-synuclein | OncoDB.HCC |
| SOCS1 | O15524 | Suppressor of cytokine signaling 1 | OncoDB.HCC |
| SPANXC | Q9UBF1 | Melanoma-associated antigen C2 | OncoDB.HCC |
| SPINT1 | O43278 | Kunitz-type protease inhibitor 1 precursor | OncoDB.HCC |
| SPINT2 | O43291 | Kunitz-type protease inhibitor 2 precursor | OncoDB.HCC |
| SPP1 | P10451 | Osteopontin precursor | OncoDB.HCC |
| SPRED1 | Q7Z699 | Sprouty-related, EVH1 domain-containing protein 1 | OncoDB.HCC |
| SPRY2 | O43597 | Sprouty homolog 2 | OncoDB.HCC |
| SRC | P12931 | Proto-oncogene tyrosine-protein kinase Src | OncoDB.HCC |
| SSTR1 | P30872 | Somatostatin receptor type 1 | OncoDB.HCC |
| SSTR2 | P30874 | Somatostatin receptor type 2 | OncoDB.HCC |
| [SSTR3](http://oncodb.hcc.ibms.sinica.edu.tw/hcc/display_by_gene.cgi?stable_id=ENSG00000183473&cut_off=1&option=simple) | P32745 | Somatostatin receptor type 3 | OncoDB.HCC |
| SSTR5 | P35346 | Somatostatin receptor type 5 | OncoDB.HCC |
| SSX2_HUMAN | Q16385 | Protein SSX2 | OncoDB.HCC |
| SSX4_HUMAN | O60224 | Protein SSX4 | OncoDB.HCC |
| SSX5 | Q16384 | Protein SSX1 | OncoDB.HCC |
| STC1 | P52823 | Stanniocalcin-1 precursor | OncoDB.HCC |
| STC2 | O76061 | Stanniocalcin-2 precursor | OncoDB.HCC |
| STEAP3 | Q658P3 | dudulin 2 isoform b | OncoDB.HCC |
| STMN2 | Q93045 | Stathmin-2 | OncoDB.HCC |
| SYCP1 | Q15431 | Synaptonemal complex protein 1 | OncoDB.HCC |
| TAOK2 | Q9UL54 | Serine/threonine-protein kinase TAO2 | OncoDB.HCC |
| TCF7L2 | Q9NQB0 | Transcription factor 7-like 2 | OncoDB.HCC |
| TEK | Q02763 | Angiopoietin-1 receptor precursor | OncoDB.HCC |
| TERT | O14746 | Telomerase reverse transcriptase | OncoDB.HCC |
| TFF3 | Q07654 | Trefoil factor 3 precursor | OncoDB.HCC |
| TFRC | P02786 | Transferrin receptor protein 1 | OncoDB.HCC |
| TGFA | P01135 | Transforming growth factor alpha precursor | OncoDB.HCC |
| TGFBR2 | P37173 | TGF-beta receptor type-2 precursor | OncoDB.HCC |
| TIAM1 | Q13009 | T-lymphoma invasion and metastasis-inducing protein 1 | OncoDB.HCC |
| TNFSF10 | P50591 | Tumor necrosis factor ligand superfamily member 10 | OncoDB.HCC |
| TNFSF11 | O14788 | Tumor necrosis factor ligand superfamily member 11 | OncoDB.HCC |
| TNFSF5IP1 | Q969U7 | tumor necrosis factor superfamily, member 5-induced protein 1 | OncoDB.HCC |
| TP53 | P04637 | Cellular tumor antigen p53 | OncoDB.HCC |
| TP73 | O15350 | Tumor protein p73 | OncoDB.HCC |
| TPTE | P56180 | Putative tyrosine-protein phosphatase TPTE | OncoDB.HCC |
| TRDMT1 | O14717 | tRNA (cytosine-5-)-methyltransferase | OncoDB.HCC |
| TSPAN8 | P19075 | Tetraspanin-8 | OncoDB.HCC |
| TSPY1_HUMAN | Q01534 | Testis-specific Y-encoded protein 1. | OncoDB.HCC |
| UBE2L3 | P68036 | Ubiquitin-conjugating enzyme E2 L3 | OncoDB.HCC |
| [UGT2B4](http://oncodb.hcc.ibms.sinica.edu.tw/hcc/display_by_gene.cgi?stable_id=ENSG00000156096&cut_off=1&option=simple) | P06133 | UDP-glucuronosyltransferase 2B4 precursor | OncoDB.HCC |
| VEGFA | P15692 | Vascular endothelial growth factor A precursor | OncoDB.HCC |
| VEGFC | P49767 | Vascular endothelial growth factor C precursor | OncoDB.HCC |
| VPS37A | Q8NEZ2 | hepatocellular carcinoma related protein 1 | OncoDB.HCC |
| VPS53 | Q5VIR6 | Vacuolar protein sorting-associated protein 53. | OncoDB.HCC |
| WWOX | NP_570859 | WW domain-containing oxidoreductase isoform 3 | OncoDB.HCC |
| XLKD1 | Q9Y5Y7 | Lymphatic vessel endothelial hyaluronic acid receptor 1 precursor | OncoDB.HCC |
| XPC | Q01831 | DNA-repair protein complementing XP-C cells | OncoDB.HCC |
| YBX1 | P67809 | Nuclease sensitive element-binding protein 1 | OncoDB.HCC |
| YY1AP1 | Q9H869 | YY1-associated protein 1 | OncoDB.HCC |
| ZHX2 | Q9Y6X8 | Zinc fingers and homeoboxes protein 2 | OncoDB.HCC |
| ZMYND10 | O75800 | Zinc finger MYND domain-containing protein 10 | OncoDB.HCC |
| ZNF342 | Q8WUU4 | Zinc finger protein 342. | OncoDB.HCC |
| ZNF689 | Q96CS4 | Zinc finger protein 689 | OncoDB.HCC |
| ZYX | Q15942 | Zyxin | OncoDB.HCC |

**Table S2**: The detailed information of ingredients and number of targets of herbs.

**1. *Radix Stephaniae Tetrandrae*** 18 ingredients

| **Serial number** | **Ingredients** | **Drug-likeness** | **Number of validated targets** | | **Number of predicted targets** |
| --- | --- | --- | --- | --- | --- |
| RST-01 | javanicin | 0.78 | | 0 | 0 |
| RST-02 | berberine | 0.78 | | 8 | 40 |
| RST-03 | faradiol | 0.77 | | 0 | 48 |
| RST-04 | beta-sitosterol | 0.75 | | 1 | 59 |
| RST-05 | jatrorrhizine | 0.75 | | 0 | 32 |
| RST-06 | hesperidin | 0.67 | | 3 | 9 |
| RST-07 | palmatine | 0.65 | | 0 | 46 |
| RST-08 | menisperine | 0.59 | | 0 | 0 |
| RST-09 | Aristololactum | 0.58 | | 0 | 19 |
| RST-10 | cyclanoline | 0.57 | | 0 | 42 |
| RST-11 | dichotomitin | 0.55 | | 0 | 43 |
| RST-12 | Betaine | 0.55 | | 2 | 37 |
| RST-13 | magnoflorine | 0.55 | | 0 | 48 |
| RST-14 | feralolide | 0.39 | | 0 | 36 |
| RST-15 | STOCK1N-53032 | 0.33 | | 0 | 28 |
| RST-16 | stepharine | 0.33 | | 0 | 30 |
| RST-17 | tetraneurin a | 0.31 | | 0 | 38 |
| RST-18 | Hesperetin | 0.27 | | 0 | 28 |

**2.** ***Flos Campsis*** **28 ingredients**

| **Serial number** | **Ingredients** | **Drug-likeness** | **Number of validated targets** | | **Number of predicted targets** |
| --- | --- | --- | --- | --- | --- |
| FC-01 | 2α,3α-dihydroxyurs-12-en-28-oic acid | 0.77 | | 0 | 35 |
| FC-02 | 3β-Hydroxyurs-12-en-28-al(Ursolic aldehyde) | 0.77 | | 0 | 47 |
| FC-03 | ursolic aldehyde | 0.77 | | 0 | 51 |
| FC-04 | 3-Epiursolic Acid | 0.76 | | 0 | 46 |
| FC-05 | alpha-amyrin | 0.76 | | 2 | 49 |
| FC-06 | Amyrin | 0.76 | | 0 | 50 |
| FC-07 | Oleanolic acid | 0.76 | | 2 | 48 |
| FC-08 | beta-sitosterol | 0.75 | | 1 | 59 |
| FC-09 | 3β,23-Dihydroxyurs-12-en-28-oic acid (23-Hydroxyursolic acid) | 0.74 | | 0 | 45 |
| FC-10 | 3-epicorosolic,acid | 0.74 | | 0 | 50 |
| FC-11 | Maslinic acid | 0.74 | | 0 | 47 |
| FC-12 | 2α,3β,23-Trihydroxy-olean-12-en-28-oic acid (Arjunolic acid) | 0.72 | | 0 | 43 |
| FC-13 | Tormentic acid | 0.71 | | 0 | 41 |
| FC-14 | Calceolarioside A | 0.68 | | 0 | 13 |
| FC-15 | acteoside | 0.62 | | 1 | 3 |
| FC-16 | Sitogluside | 0.62 | | 0 | 23 |
| FC-17 | cyanidol | 0.24 | | 0 | 22 |
| FC-18 | apigenin | 0.21 | | 22 | 7 |
| FC-19 | Linolenic acid ethyl ester | 0.20 | | 0 | 4 |
| FC-20 | Ethyl stearate | 0.19 | | 0 | 1 |
| FC-21 | Mandenol | 0.19 | | 0 | 6 |
| FC-22 | cyanidin-3-rutinoside | 0.72 | | 0 | 0 |
| FC-23 | Capsanthin | 0.51 | | 0 | 0 |
| FC-24 | Myrizylalkohol | 0.46 | | 0 | 0 |
| FC-25 | HEXACOSANE | 0.31 | | 0 | 0 |
| FC-26 | PENTACOSANE | 0.27 | | 0 | 0 |
| FC-27 | Tetracosane | 0.24 | | 0 | 0 |
| FC-28 | Tricosane | 0.21 | | 0 | 0 |

**3.** ***Carapax Trionycis*** **20 ingredients**

| **Serial number** | **Ingredients** | **Drug-likeness** | **Number of validated targets** | | **Number of predicted targets** |
| --- | --- | --- | --- | --- | --- |
| CT-01 | Aspartic acid |  | | 0 | 47 |
| CT-02 | Valine |  | | 0 | 43 |
| CT-03 | Methionine |  | | 0 | 34 |
| CT-04 | Cysteine |  | | 0 | 31 |
| CT-05 | Isoleucine |  | | 0 | 50 |
| CT-06 | Leucine |  | | 0 | 48 |
| CT-07 | Phenylanine |  | | 0 | 23 |
| CT-08 | Mannose |  | | 0 | 36 |
| CT-09 | Galacturonic acid |  | | 0 | 52 |
| CT-10 | Glutamine |  | | 0 | 36 |
| CT-11 | Galactose |  | | 0 | 6 |
| CT-12 | Glucose |  | | 0 | 36 |
| CT-13 | Glucuronic acid |  | | 1 | 52 |
| CT-14 | Serine |  | | 0 | 18 |
| CT-15 | Glycine |  | | 0 | 2 |
| CT-16 | Arginine |  | | 0 | 29 |
| CT-17 | Threonine |  | | 0 | 36 |
| CT-18 | Alanine |  | | 0 | 6 |
| CT-19 | Proline |  | | 0 | 32 |
| CT-20 | Tyrosine |  | | 0 | 38 |

**4.** ***Radix Scutellariae*** **85 ingredients**

| **Serial number** | **Ingredients** | **Drug-likeness** | **Number of validated targets** | | **Number of predicted targets** |
| --- | --- | --- | --- | --- | --- |
| RS-01 | coptisine | 0.86 | | 0 | 39 |
| RS-02 | stigmasterol-beta-d-glucoside | 0.85 | | 0 | 43 |
| RS-03 | isoschaftoside | 0.83 | | 0 | 31 |
| RS-04 | scutellarin | 0.79 | | 0 | 34 |
| RS-05 | epiberberine | 0.78 | | 0 | 44 |
| RS-06 | Oroxindin/wogonoside | 0.77 | | 0 | 38 |
| RS-07 | Baicalin | 0.75 | | 0 | 30 |
| RS-08 | stigmasterol | 0.76 | | 0 | 40 |
| RS-09 | beta-sitosterol | 0.75 | | 1 | 59 |
| RS-10 | sitosterol | 0.75 | | 0 | 59 |
| RS-11 | dihydrobaicalin | 0.75 | | 0 | 29 |
| RS-12 | gamma-sitosterol | 0.75 | | 0 | 30 |
| RS-13 | Cosmetin | 0.74 | | 0 | 34 |
| RS-14 | (+)-Syringaresinol | 0.72 | | 0 | 37 |
| RS-15 | campesterol | 0.72 | | 0 | 30 |
| RS-16 | 3,4',5,7-tetrahydroxyflavone-3-L-rhamnoside | 0.70 | | 0 | 41 |
| RS-17 | (2S,3R,4R,5R,6S)-2-[(2R,3R,4S,5R,6R)-3,5-dihydroxy-2-[2-(3-hydroxy-4-methoxy-phenyl)ethoxy]-6-methylol-tetrahydropyran-4-yl]oxy-6-methyl-tetrahydropyran-3,4,5-triol | 0.67 | | 0 | 39 |
| RS-18 | Sitogluside | 0.62 | | 0 | 23 |
| RS-19 | darendoside B | 0.59 | | 0 | 43 |
| RS-20 | campesteryl ferulate | 0.59 | | 0 | 1 |
| RS-21 | jatrorrizine | 0.59 | | 0 | 45 |
| RS-22 | Isomartynoside | 0.56 | | 0 | 7 |
| RS-23 | Methyl montanate | 0.48 | | 0 | 0 |
| RS-24 | 5,7,2,5-tetrahydroxy-8,6-dimethoxyflavone | 0.45 | | 0 | 45 |
| RS-25 | catalpol | 0.44 | | 2 | 42 |
| RS-26 | skullcapflavone ii | 0.44 | | 0 | 46 |
| RS-27 | NEOBAICALEIN | 0.44 | | 0 | 43 |
| RS-28 | 5,2',5'-trihydroxy-6,7,8-trimethoxyflavone | 0.43 | | 0 | 47 |
| RS-29 | METHYL HEXACOSANOATE | 0.43 | | 0 | 0 |
| RS-30 | Supraene | 0.42 | | 0 | 1 |
| RS-31 | HEXATRIACONTANE | 0.41 | | 0 | 0 |
| RS-32 | Diop | 0.39 | | 0 | 16 |
| RS-33 | Ganhuangenin | 0.37 | | 0 | 26 |
| RS-34 | Viscidulin III | 0.37 | | 0 | 42 |
| RS-35 | Methyl lignocerate | 0.37 | | 0 | 0 |
| RS-36 | rivularin | 0.37 | | 0 | 38 |
| RS-37 | 5,2'-dihydroxy-6,7,8-trimethoxyflavone | 0.35 | | 0 | 39 |
| RS-38 | bis[(2S)-2-ethylhexyl] benzene-1,2-dicarboxylate | 0.35 | | 0 | 28 |
| RS-39 | Salvigenin | 0.33 | | 0 | 42 |
| RS-40 | Methyl tricosanoate | 0.33 | | 0 | 0 |
| RS-41 | 5-o-caffeoylquinic acid | 0.33 | | 0 | 43 |
| RS-42 | 5,2',6'-trihydroxy-7,8-dimethoxyflavone | 0.33 | | 0 | 38 |
| RS-43 | 5,6,3',4'-tetrahydroxy-7-methoxyflavone | 0.31 | | 0 | 35 |
| RS-44 | paniculatin | 0.30 | | 0 | 51 |
| RS-45 | 5,8-Dihydroxy-6,7-dimethoxyflavone | 0.29 | | 0 | 32 |
| RS-46 | Methyl behenate | 0.29 | | 0 | 0 |
| RS-47 | Panicolin | 0.29 | | 0 | 39 |
| RS-48 | scutevulin | 0.27 | | 0 | 21 |
| RS-49 | 5,7,4'-trihydroxy-6-methoxyflavanone | 0.27 | | 0 | 33 |
| RS-50 | chrysoeriol | 0.27 | | 0 | 0 |
| RS-51 | 5,8,2'-trihydroxy-6,7-dimethoxyflavone | 0.27 | | 0 | 24 |
| RS-52 | 5,7,4'-trihydroxy-8-methoxyflavone | 0.27 | | 0 | 15 |
| RS-53 | 2-(2,6-dihydroxyphenyl)-3,5,7-trihydroxy-chromone | 0.27 | | 0 | 24 |
| RS-54 | methyl henicosanoate | 0.26 | | 0 | 0 |
| RS-55 | 5,7,4'-trihydroxy-8-methoxyflavanone | 0.26 | | 0 | 21 |
| RS-56 | Moslosooflavone | 0.25 | | 0 | 32 |
| RS-57 | TETRATETRACONTANE | 0.25 | | 0 | 0 |
| RS-58 | acacetin | 0.24 | | 2 | 17 |
| RS-59 | Eriodyctiol (flavanone) | 0.24 | | 0 | 24 |
| RS-60 | ent-Epicatechin | 0.24 | | 0 | 23 |
| RS-61 | scutellarein | 0.24 | | 0 | 14 |
| RS-62 | Carthamidin | 0.24 | | 0 | 16 |
| RS-63 | 2',3',5,7-tetrahydroxyflavone | 0.24 | | 0 | 21 |
| RS-64 | 5,7,2',6'-tetrahydroxyflavone | 0.24 | | 0 | 26 |
| RS-65 | wogonin | 0.23 | | 4 | 22 |
| RS-66 | oroxylin a | 0.23 | | 2 | 24 |
| RS-67 | DIHYDROOROXYLIN | 0.23 | | 0 | 20 |
| RS-68 | 7-methoxybaicalein/Negletein | 0.23 | | 0 | 15 |
| RS-69 | methyl icos-11-enoate | 0.23 | | 0 | 2 |
| RS-70 | 11,13-Eicosadienoic acid, methyl ester | 0.23 | | 0 | 1 |
| RS-71 | sucrose | 0.23 | | 5 | 41 |
| RS-72 | dihydrooroxylin a | 0.23 | | 0 | 24 |
| RS-73 | Methyl icosanoate | 0.22 | | 0 | 0 |
| RS-74 | 2,6,2',4'-tetrahydroxy-6'-methoxychaleone | 0.22 | | 0 | 40 |
| RS-75 | darendoside B_qt | 0.22 | | 0 | 41 |
| RS-76 | Dihydrobaicalin_qt | 0.21 | | 0 | 24 |
| RS-77 | norwogonin | 0.21 | | 0 | 20 |
| RS-78 | apigenin | 0.21 | | 22 | 7 |
| RS-79 | baicalein | 0.21 | | 6 | 15 |
| RS-80 | 7,3',4'-trihydroxyflavone | 0.21 | | 0 | 14 |
| RS-81 | (2R)-7-hydroxy-5-methoxy-2-phenylchroman-4-one | 0.20 | | 0 | 17 |
| RS-82 | salidroside | 0.20 | | 1 | 35 |
| RS-83 | METHYL NONADECANOATE | 0.19 | | 0 | 1 |
| RS-84 | 1,3,5-trihydroxyxanthone | 0.19 | | 0 | 28 |
| RS-85 | chrysin | 0.18 | | 1 | 14 |

**5.** ***Radix Achyranthis Bidentatae*** **75 ingredients**

| **Serial number** | **Ingredients** | **Drug-likeness** | **Number of validated targets** | | **Number of predicted targets** | |
| --- | --- | --- | --- | --- | --- | --- |
| RAB-01 | coptisine | 0.86 | 1 | 39 | |  |
| RAB-02 | (20r,22r)-2beta,3beta,20,22,26-pentahydroxy-cholestan-7,12-dien-6-one | 0.85 | 0 | 28 | |  |
| RAB-03 | (20R,22R)-2β,3β,20,22,26-pentahydroxy-cholestan-7,12-dien-6-one | 0.85 | 0 | 28 | |  |
| RAB-04 | Rubschisantherin | 0.85 | 0 | 28 | |  |
| RAB-05 | Inophyllum E | 0.85 | 0 | 49 | |  |
| RAB-06 | inophyllolide | 0.85 | 0 | 31 | |  |
| RAB-07 | 2β,3β,20α,22α,25-pentahydroxy cholesta-8,14-dien-6-one | 0.84 | 0 | 31 | |  |
| RAB-08 | stachysterone D | 0.84 | 0 | 40 | |  |
| RAB-09 | Inokosterone | 0.83 | 0 | 27 | |  |
| RAB-10 | achyranthesterone A | 0.83 | 0 | 31 | |  |
| RAB-11 | Ecdysterone-3-O-beta-D-glucopyranoside_qt | 0.83 | 0 | 31 | |  |
| RAB-12 | ginsenoside-Rg2_qt | 0.82 | 0 | 26 | |  |
| RAB-13 | rhapontisterone B | 0.82 | 0 | 31 | |  |
| RAB-14 | β-ecdysterone | 0.82 | 0 | 27 | |  |
| RAB-15 | ecdysterone | 0.82 | 1 | 27 | |  |
| RAB-16 | Polypodine B | 0.81 | 0 | 28 | |  |
| RAB-17 | 28-norolean-17-en-3-ol | 0.78 | 0 | 49 | |  |
| RAB-18 | berberine | 0.78 | 8 | 40 | |  |
| RAB-19 | epiberberine | 0.78 | 0 | 45 | |  |
| RAB-20 | (3S,4aR,6aR,6bS,8aS,12aS,14aR,14bR)-4,4,6a,6b,11,11,14b-heptamethyl-1,2,3,4a,5,6,7,8,9,10,12,12a,14,14a-tetradecahydropicene-3,8a-diol | 0.77 | 0 | 48 | |  |
| RAB-21 | Hyperin | 0.77 | 0 | 41 | |  |
| RAB-22 | 3-epioleanolic acid | 0.76 | 0 | 48 | |  |
| RAB-23 | 18-(β-D-Oxy glucose)-28-Oxo-12-oleanolic acid-3β-3-O-(β-D-glucose)-β-D-glucuronic acid methylester_qt | 0.76 | 0 | 47 | |  |
| RAB-24 | achybidensaponin,ii_qt | 0.76 | 0 | 57 | |  |
| RAB-25 | deglucose chikusetsusaponin Iva_qt | 0.76 | 0 | 49 | |  |
| RAB-26 | zingibroside r1_qt | 0.76 | 0 | 56 | |  |
| RAB-27 | Spinasterol | 0.76 | 0 | 53 | |  |
| RAB-28 | Stigmasterol | 0.76 | 0 | 40 | |  |
| RAB-29 | α-spinasterol | 0.76 | 0 | 36 | |  |
| RAB-30 | delta 7-stigmastenol | 0.75 | 0 | 40 | |  |
| RAB-31 | Baicalin | 0.75 | 0 | 30 | |  |
| RAB-32 | beta-sitosterol | 0.75 | 1 | 59 | |  |
| RAB-33 | beta-daucosterol_qt | 0.75 | 0 | 30 | |  |
| RAB-34 | hederagenin-28-O-β-D-glucopyranosyl ester_qt | 0.74 | 0 | 59 | |  |
| RAB-35 | Astragalin | 0.74 | 0 | 27 | |  |
| RAB-36 | N-trans-feruloyl-3-methoxytyramine-4-O-β-D-glucopyranoside | 0.73 | 0 | 61 | |  |
| RAB-37 | N-trans-feruloyl-3-methoxytyramine-4'-O-β-D-glucopyranoside | 0.72 | 0 | 43 | |  |
| RAB-38 | stachysterone A | 0.69 | 0 | 40 | |  |
| RAB-39 | Chrysophanol-8-O-beta-D-(6'-O-galloyl)-glucopyranoside | 0.69 | 0 | 22 | |  |
| RAB-40 | quercetin-3-O-rutinoside | 0.68 | 0 | 19 | |  |
| RAB-41 | rutin | 0.68 | 6 | 25 | |  |
| RAB-42 | α-spinasterol-β-d-glucoside | 0.67 | 0 | 41 | |  |
| RAB-43 | palmatine | 0.65 | 0 | 46 | |  |
| RAB-44 | 3-O-β-D-glucopyranosyl-α-spinalsterol | 0.63 | 0 | 6 | |  |
| RAB-45 | (2R,3R,4S,5S,6R)-2-[[(3S,5S,9R,10S,13R,14R,17R)-17-[(E,2R,5S)-5-ethyl-6-methylhept-3-en-2-yl]-10,13-dimethyl-2,3,4,5,6,9,11,12,14,15,16,17-dodecahydro-1H-cyclopenta[a]phenanthren-3-yl]oxy]-6-(hydroxymethyl)oxane-3,4,5-triol | 0.63 | 0 | 23 | |  |
| RAB-46 | beta-daucosterol | 0.63 | 0 | 2 | |  |
| RAB-47 | Sitogluside | 0.62 | 0 | 23 | |  |
| RAB-48 | bidentatoside,ii | 0.26 | 0 | 17 | |  |
| RAB-49 | bidentatoside,ii_qt | 0.59 | 0 | 39 | |  |
| RAB-50 | niuxixinsterone B | 0.59 | 0 | 24 | |  |
| RAB-51 | niuxixinsterone C | 0.56 | 0 | 21 | |  |
| RAB-52 | Ecdysterone-3-O-beta-D-glucopyranoside | 0.55 | 0 | 5 | |  |
| RAB-53 | niuxixinsterone A | 0.54 | 0 | 28 | |  |
| RAB-54 | rubrosterone | 0.47 | 0 | 42 | |  |
| RAB-55 | geniposide | 0.44 | 0 | 38 | |  |
| RAB-56 | pjs-1 | 0.41 | 0 | 31 | |  |
| RAB-57 | hederagenin-28-O-β-D-glucopyranosyl ester | 0.39 | 0 | 23 | |  |
| RAB-58 | Hmp-hmpep | 0.34 | 0 | 30 | |  |
| RAB-59 | 3-O-(β-D-glucopyranosiduronic acid) oleanolic acid | 0.32 | 0 | 21 | |  |
| RAB-60 | 3-O-β-D-glucuronopyranoside-6-O-methyl ester | 0.32 | 0 | 8 | |  |
| RAB-61 | achybidensaponin,i_qt | 0.32 | 0 | 10 | |  |
| RAB-62 | quercetin | 0.28 | 37 | 11 | |  |
| RAB-63 | deglucose chikusetsusaponin Iva | 0.34 | 0 | 8 | |  |
| RAB-64 | kaempferol | 0.24 | 10 | 16 | |  |
| RAB-65 | wogonin | 0.23 | 4 | 22 | |  |
| RAB-66 | Crysophanol | 0.21 | 0 | 33 | |  |
| RAB-67 | baicalein | 0.21 | 6 | 15 | |  |
| RAB-68 | chrysophanol | 0.21 | 0 | 18 | |  |
| RAB-69 | poriferasta-7,22E-dien-3beta-ol | 42.98 | 0 | 0 | |  |
| RAB-70 | Spinoside A | 41.75 | 0 | 0 | |  |
| RAB-71 | 3-O-β-D-glucuronopyranoside-6-O-butyl ester | 0.26 | 0 | 0 | |  |
| RAB-72 | chikusetsusaponin I | 0.25 | 0 | 0 | |  |
| RAB-73 | Tetracosane | 0.24 | 0 | 0 | |  |
| RAB-74 | Tricosane | 0.21 | 0 | 0 | |  |
| RAB-75 | achyranthoside E_qt | 0.20 | 0 | 0 | |  |

**6.** ***Radix Bupleuri* 59 ingredients**

| **Serial number** | **Ingredients** | **Drug-likeness** | **Number of validated targets** | **Number of predicted targets** |
| --- | --- | --- | --- | --- |
| RB-01 | Linoleyl acetate | 0.20 | 0 | 5 |
| RB-02 | 20-Hexadecanoylingenol | 0.68 | 0 | 0 |
| RB-03 | capsaicin | 0.20 | 10 | 28 |
| RB-04 | Nonacosanol | 0.43 | 0 | 0 |
| RB-05 | Baicalin | 0.75 | 0 | 30 |
| RB-06 | 5,8-Dihydroxy-6,7-dimethoxyflavone | 0.29 | 0 | 32 |
| RB-07 | Stigmasterol | 0.76 | 0 | 40 |
| RB-08 | Myricadiol | 0.77 | 0 | 48 |
| RB-09 | Isorhamnetin-3-mono-beta-D-glucoside | 0.80 | 0 | 45 |
| RB-10 | [isorhamnetin](http://lsp.nwsuaf.edu.cn/molecule.php?qn=354) | 0.31 | 1 | 16 |
| RB-11 | Narcissoside | 0.65 | 0 | 30 |
| RB-12 | kaempferol | 0.24 | 10 | 16 |
| RB-13 | 11α-methoxysaikosaponin f_qt | 0.75 | 0 | 50 |
| RB-14 | 3'-O-Acetylsaikosaponin D_qt | 0.63 | 0 | 54 |
| RB-15 | 3',6'-O,O-diacetylsaikosaponin b2_qt | 0.74 | 0 | 60 |
| RB-16 | 3,5,6,7-tetramethoxy-2-(3,4,5-trimethoxyphenyl)chromone | 0.59 | 0 | 42 |
| RB-17 | FLAVONE,3,5,7-TRIHYDROXY-3,4-DIMETHOXY | 0.33 | 0 | 38 |
| RB-18 | Areapillin | 0.41 | 0 | 47 |
| RB-19 | 6,7,3',8'-diligustilide | 0.70 | 0 | 30 |
| RB-20 | 6-O-Vanilloylajugol | 0.86 | 0 | 30 |
| RB-21 | Cubebin | 0.64 | 0 | 40 |
| RB-22 | Longikaurin A | 0.53 | 0 | 11 |
| RB-23 | Longispinogenin 3-O-beta-D-glucuronopyranoside | 0.34 | 0 | 10 |
| RB-24 | Longispinogenin | 0.75 | 0 | 48 |
| RB-25 | Octalupine | 0.28 | 0 | 27 |
| RB-26 | puerarin | 0.69 | 12 | 35 |
| RB-27 | 7,8,4'-Trihydroxyisoflavone | 0.22 | 0 | 15 |
| RB-28 | Pulsatillic acid | 0.77 | 0 | 53 |
| RB-29 | Saikogenin F | 0.63 | 0 | 41 |
| RB-30 | (3S,4aS,6aR,6bS,8S,8aS,12aS,14aR,14bR)-4,4,6a,6b,11,11,14b-heptamethyl-8a-methylol-1,2,3,4a,5,6,7,8,9,10,12,12a,14,14a-tetradecahydropicene-3,8-diol | 0.75 | 0 | 48 |
| RB-31 | Saikosaponin t_qt | 0.71 | 0 | 56 |
| RB-32 | Sainfuran | 0.23 | 0 | 37 |
| RB-33 | Thymonin | 0.41 | 0 | 46 |
| RB-34 | Troxerutin | 0.28 | 0 | 29 |
| RB-35 | (2R,3R,4S,5S,6R)-2-[[(3S,5S,9R,10S,13R,14R,17R)-17-[(E,2R,5S)-5-ethyl-6-methylhept-3-en-2-yl]-10,13-dimethyl-2,3,4,5,6,9,11,12,14,15,16,17-dodecahydro-1H-cyclopenta[a]phenanthren-3-yl]oxy]-6-(hydroxymethyl)oxane-3,4,5-triol | 0.63 | 0 | 23 |
| RB-36 | (+)-Anomalin | 0.66 | 0 | 50 |
| RB-37 | chikusaikoside II_qt | 0.63 | 0 | 41 |
| RB-38 | kaempferitrin | 0.79 | 0 | 42 |
| RB-39 | kaempferol-3,7-α-L-dirhamnoside | 0.79 | 0 | 34 |
| RB-40 | kaempferol-7-O-rhamnoside | 0.72 | 0 | 41 |
| RB-41 | kaempferol-7-O-α-L-rhamnoside | 0.72 | 0 | 33 |
| RB-42 | PLO | 0.43 | 0 | 39 |
| RB-43 | rutin | 0.68 | 6 | 25 |
| RB-44 | saikosaponin b1_qt | 0.74 | 0 | 47 |
| RB-45 | saikosaponin b2_qt | 0.74 | 0 | 46 |
| RB-46 | saikosaponin b3_qt | 0.69 | 0 | 48 |
| RB-47 | saikosaponin c_qt | 0.63 | 0 | 53 |
| RB-48 | saikosaponin e_qt | 0.66 | 0 | 52 |
| RB-49 | α-spinasterol | 0.76 | 0 | 36 |
| RB-50 | α-spinasteryl glucoside | 0.63 | 0 | 6 |
| RB-51 | petunidin | 0.31 | 0 | 24 |
| RB-52 | darutoside | 0.63 | 0 | 10 |
| RB-53 | quercetin | 0.28 | 37 | 11 |
| RB-54 | oleanolic acid | 0.76 | 2 | 48 |
| RB-55 | Octacosane | 0.37 | 0 | 0 |
| RB-56 | Oroxindin | 0.77 | 0 | 38 |
| RB-57 | alpha-spinasterol-beta-d-glucoside | 0.67 | 0 | 41 |
| RB-58 | stigmasterol-beta-d-glucoside | 0.63 | 0 | 3 |
| RB-59 | spinasterol | 0.76 | 0 | 53 |

**7.** ***Semen Lepidii* 31 ingredients**

| **Serial number** | **Ingredients** | **Drug-likeness** | **Number of validated targets** | **Number of predicted targets** |
| --- | --- | --- | --- | --- |
| SL-01 | K-STROPHANTHOSIDE_qt | 0.78 | 0 | 38 |
| SL-02 | Cynotoxin | 0.78 | 0 | 51 |
| SL-03 | 3,5-dihydroxy-2-(4-hydroxyphenyl)-7-[(2S,3R,4S,5S,6R)-3,4,5-trihydroxy-6-(hydroxymethyl)oxan-2-yl]oxychromen-4-one | 0.76 | 0 | 36 |
| SL-04 | Sitosterol | 0.75 | 0 | 59 |
| SL-05 | hederagenin | 0.75 | 0 | 36 |
| SL-06 | beta-sitosterol | 0.75 | 1 | 59 |
| SL-07 | evobioside_qt | 0.71 | 0 | 44 |
| SL-08 | Helveticoside | 0.69 | 0 | 37 |
| SL-09 | evomonoside | 0.69 | 0 | 39 |
| SL-10 | alexandrin | 0.63 | 0 | 7 |
| SL-11 | isorhamnetin | 0.31 | 1 | 16 |
| SL-12 | (E)-tricos-11-enoic acid | 0.30 | 0 | 1 |
| SL-13 | quercetin | 0.28 | 37 | 11 |
| SL-14 | Erucic acid | 0.26 | 0 | 1 |
| SL-15 | kaempferol | 0.24 | 10 | 18 |
| SL-16 | evobioside | 0.24 | 0 | 15 |
| SL-17 | methyl (Z)-icos-11-enoate | 0.23 | 0 | 2 |
| SL-18 | erysimoside | 0.23 | 0 | 14 |
| SL-19 | K-STROPHANTHOSIDE | 0.22 | 0 | 3 |
| SL-20 | Eicosenoic acid | 0.20 | 0 | 1 |
| SL-21 | Dihomolinolenic acid | 0.20 | 0 | 5 |
| SL-22 | 11,14-eicosadienoic acid | 0.20 | 0 | 6 |
| SL-23 | Myronate | 0.19 | 0 | 44 |
| SL-24 | Arachic acid | 0.19 | 0 | 1 |
| SL-25 | Sinigrin | 0.18 | 0 | 39 |
| SL-26 | Erucic acid ethyl ester | 0.33 | 0 | 0 |
| SL-27 | Docosanoate | 0.26 | 0 | 0 |
| SL-28 | Methyl icosanoate | 0.22 | 0 | 0 |
| SL-29 | Arachidic acid | 0.19 | 0 | 0 |
| SL-30 | STOCK1N-70051 | 0.78 | 0 | 0 |
| SL-31 | Strophanthidine | 0.78 | 0 | 0 |

8. ***Rhizoma Atractylodis Macrocephalae* 11 ingredients**

| **Serial number** | **Ingredients** | **Drug-likeness** | **Number of validated targets** | **Number of predicted targets** |
| --- | --- | --- | --- | --- |
| RAM-01 | ATRACTYLODES MACROCEPHALA | 0.81 | 0 | 26 |
| RAM-02 | biatractylolide | 0.81 | 0 | 26 |
| RAM-03 | 2alpha-(alpha-methylbutyrul)-oxy-5alpha,7belta,9alpha,10belta-tetraacetoxy-4(20),11-taxadiene | 0.80 | 0 | 3 |
| RAM-04 | (3S,8S,9S,10R,13R,14S,17R)-10,13-dimethyl-17-[(2R,5S)-5-propan-2-yloctan-2-yl]-2,3,4,7,8,9,11,12,14,15,16,17-dodecahydro-1H-cyclopenta[a]phenanthren-3-ol | 0.78 | 0 | 44 |
| RAM-05 | α-Amyrin | 0.76 | 0 | 50 |
| RAM-06 | stigmast-22E-en-3beta-ol | 0.75 | 0 | 50 |
| RAM-07 | 14-acetyl-12-senecioyl-2E,8E,10E-atractylentriol | 0.31 | 0 | 0 |
| RAM-08 | 14-acetyl-12-senecioyl-2E,8Z,10E-atractylentriol | 0.30 | 0 | 0 |
| RAM-09 | 12-senecioyl-2E,8E,10E-atractylentriol | 0.22 | 0 | 0 |
| RAM-10 | 3β-acetoxyatractylone | 0.22 | 0 | 33 |
| RAM-11 | 8β-ethoxy atractylenolide Ⅲ | 0.21 | 0 | 0 |

**Table S3**: The detailed target information of the ingredients of herbs.

***Radix Stephaniae Tetrandrae* (RST) *Flos Campsis* (FC) *Carapax Trionycis*** **(CT)  *Radix Scutellariae* (RS)**

***Radix Achyranthis Bidentatae* （RAB）*Radix Bupleuri* (RB) *Semen Lepidii* (SL) *Rhizoma Atractylodis Macrocephalae* (RAM)**

| **Ingredients** | **Targets** | **Validated or Predicted** | **Score** | **Ingredients** | **Targets** | **Validated or Predicted** | **Score** |
| --- | --- | --- | --- | --- | --- | --- | --- |
| RST-02 | ABCG2 | Validated | 1.000 | FC-01 | ABCB1 | Predicted | 0.022 |
| RST-02 | AGXT | Predicted | 0.006 | FC-01 | ACSL1 | Predicted | 0.022 |
| RST-02 | AKR1B10 | Predicted | 0.084 | FC-01 | ADH1C | Predicted | 0.018 |
| RST-02 | AKT2 | Predicted | 0.047 | FC-01 | AGXT | Predicted | 0.029 |
| RST-02 | AURKA | Predicted | 0.004 | FC-01 | AKT2 | Predicted | 0.357 |
| RST-02 | CA1 | Predicted | 0.039 | FC-01 | ASS1 | Predicted | 0.022 |
| RST-02 | CA2 | Predicted | 0.039 | FC-01 | AURKA | Predicted | 0.018 |
| RST-02 | CASP3 | Validated | 1.000 | FC-01 | CA1 | Predicted | 0.185 |
| RST-02 | CCNA2 | Predicted | 1.000 | FC-01 | CA2 | Predicted | 0.185 |
| RST-02 | CCT3 | Predicted | 0.004 | FC-01 | CCNA2 | Predicted | 0.373 |
| RST-02 | CDK4 | Validated | 1.000 | FC-01 | CCT3 | Predicted | 0.018 |
| RST-02 | CTSB | Predicted | 0.082 | FC-01 | CES1 | Predicted | 0.018 |
| RST-02 | CXCL12 | Validated | 1.000 | FC-01 | CTSB | Predicted | 0.381 |
| RST-02 | ESR1 | Predicted | 0.161 | FC-01 | ESR1 | Predicted | 0.399 |
| RST-02 | GABRE | Predicted | 0.003 | FC-01 | GLUD1 | Predicted | 0.052 |
| RST-02 | GLUD1 | Predicted | 0.013 | FC-01 | GNAI1 | Predicted | 0.022 |
| RST-02 | GNAI1 | Predicted | 0.005 | FC-01 | HAO1 | Predicted | 0.000 |
| RST-02 | GNMT | Predicted | 0.006 | FC-01 | HSP90AA1 | Predicted | 0.018 |
| RST-02 | GSTP1 | Predicted | 0.007 | FC-01 | HSP90B1 | Predicted | 0.018 |
| RST-02 | HAO1 | Predicted | 0.000 | FC-01 | HSPA1B | Predicted | 0.018 |
| RST-02 | HSP90AA1 | Predicted | 0.004 | FC-01 | HSPA8 | Predicted | 0.018 |
| RST-02 | HSP90B1 | Predicted | 0.004 | FC-01 | KRAS | Predicted | 0.111 |
| RST-02 | HSPA1B | Predicted | 0.004 | FC-01 | MAT1A | Predicted | 0.018 |
| RST-02 | HSPA8 | Predicted | 0.004 | FC-01 | MME | Predicted | 0.254 |
| RST-02 | IGF2 | Validated | 1.000 | FC-01 | MMP12 | Predicted | 0.121 |
| RST-02 | KRAS | Predicted | 0.020 | FC-01 | NME1 | Predicted | 0.080 |
| RST-02 | LYZ | Predicted | 0.081 | FC-01 | NME2 | Predicted | 0.022 |
| RST-02 | MAPK1 | Predicted | 0.079 | FC-01 | PGK1 | Predicted | 0.018 |
| RST-02 | MAPK1 | Validated | 1.000 | FC-01 | PTK2 | Predicted | 0.018 |
| RST-02 | MAPK3 | Predicted | 0.025 | FC-01 | RHOA | Predicted | 0.022 |
| RST-02 | MAT1A | Predicted | 0.004 | FC-01 | RND3 | Predicted | 0.023 |
| RST-02 | MME | Predicted | 0.052 | FC-01 | SERPINE1 | Predicted | 0.033 |
| RST-02 | NME1 | Predicted | 0.018 | FC-01 | SGK1 | Predicted | 0.000 |
| RST-02 | NME2 | Predicted | 0.005 | FC-01 | TGM2 | Predicted | 0.022 |
| RST-02 | NOS2A | Validated | 1.000 | FC-01 | TOP2A | Predicted | 0.092 |
| RST-02 | PGK1 | Predicted | 0.004 | FC-02 | ABCB1 | Predicted | 0.006 |
| RST-02 | PTGS2 | Predicted | 0.109 | FC-02 | ACSL1 | Predicted | 0.006 |
| RST-02 | PTK2 | Predicted | 0.004 | FC-02 | ADH1C | Predicted | 0.006 |
| RST-02 | RHOA | Predicted | 0.005 | FC-02 | AGXT | Predicted | 0.006 |
| RST-02 | RND3 | Predicted | 0.006 | FC-02 | AKR1B10 | Predicted | 0.096 |
| RST-02 | SERPINE1 | Predicted | 0.028 | FC-02 | AKT2 | Predicted | 0.147 |
| RST-02 | SGK1 | Predicted | 0.000 | FC-02 | ASS1 | Predicted | 0.006 |
| RST-02 | SRC | Predicted | 0.071 | FC-02 | AURKA | Predicted | 0.005 |
| RST-02 | TGM2 | Predicted | 0.005 | FC-02 | CA1 | Predicted | 0.072 |
| RST-02 | TOP2A | Predicted | 0.047 | FC-02 | CA2 | Predicted | 0.072 |
| RST-02 | TRDMT1 | Predicted | 0.006 | FC-02 | CASP1 | Predicted | 0.022 |
| RST-02 | VEGFA | Validated | 1.000 | FC-02 | CCNA2 | Predicted | 1.000 |
| RST-03 | ABCB1 | Predicted | 0.029 | FC-02 | CCT3 | Predicted | 0.005 |
| RST-03 | ACSL1 | Predicted | 0.087 | FC-02 | CDK4 | Predicted | 0.038 |
| RST-03 | ADH1C | Predicted | 0.015 | FC-02 | CES1 | Predicted | 0.006 |
| RST-03 | AGXT | Predicted | 0.018 | FC-02 | CTSB | Predicted | 0.096 |
| RST-03 | AKT2 | Predicted | 0.374 | FC-02 | ESR1 | Predicted | 0.181 |
| RST-03 | ASS1 | Predicted | 0.029 | FC-02 | GABRE | Predicted | 0.001 |
| RST-03 | AURKA | Predicted | 0.017 | FC-02 | GLUD1 | Predicted | 0.017 |
| RST-03 | CA1 | Predicted | 0.183 | FC-02 | GNAI1 | Predicted | 0.005 |
| RST-03 | CA2 | Predicted | 0.183 | FC-02 | GNMT | Predicted | 0.003 |
| RST-03 | CASP1 | Predicted | 0.052 | FC-02 | GSTP1 | Predicted | 0.030 |
| RST-03 | CCNA2 | Predicted | 1.000 | FC-02 | HAO1 | Predicted | 0.000 |
| RST-03 | CCT3 | Predicted | 0.017 | FC-02 | HSP90AA1 | Predicted | 0.005 |
| RST-03 | CDK4 | Predicted | 0.078 | FC-02 | HSP90B1 | Predicted | 0.005 |
| RST-03 | CES1 | Predicted | 0.015 | FC-02 | HSPA1B | Predicted | 0.005 |
| RST-03 | CTSB | Predicted | 0.251 | FC-02 | HSPA8 | Predicted | 0.005 |
| RST-03 | ESR1 | Predicted | 0.758 | FC-02 | KRAS | Predicted | 0.030 |
| RST-03 | GABRE | Predicted | 0.011 | FC-02 | MAPK1 | Predicted | 0.080 |
| RST-03 | GLUD1 | Predicted | 0.051 | FC-02 | MAPK3 | Predicted | 0.038 |
| RST-03 | GNAI1 | Predicted | 0.019 | FC-02 | MAT1A | Predicted | 0.005 |
| RST-03 | GNMT | Predicted | 0.011 | FC-02 | MME | Predicted | 0.150 |
| RST-03 | GSTP1 | Predicted | 0.070 | FC-02 | MMP12 | Predicted | 0.030 |
| RST-03 | HAO1 | Predicted | 0.000 | FC-02 | MMP9 | Predicted | 0.022 |
| RST-03 | HSP90AA1 | Predicted | 0.017 | FC-02 | NME1 | Predicted | 0.025 |
| RST-03 | HSP90B1 | Predicted | 0.017 | FC-02 | NME2 | Predicted | 0.005 |
| RST-03 | HSPA1B | Predicted | 0.017 | FC-02 | PGK1 | Predicted | 0.005 |
| RST-03 | HSPA8 | Predicted | 0.017 | FC-02 | PTK2 | Predicted | 0.005 |
| RST-03 | KRAS | Predicted | 0.066 | FC-02 | RHOA | Predicted | 0.005 |
| RST-03 | MAPK1 | Predicted | 0.078 | FC-02 | RND3 | Predicted | 0.008 |
| RST-03 | MAPK3 | Predicted | 0.157 | FC-02 | SERPINE1 | Predicted | 0.015 |
| RST-03 | MAT1A | Predicted | 0.017 | FC-02 | SGK1 | Predicted | 0.000 |
| RST-03 | MME | Predicted | 0.171 | FC-02 | SRC | Predicted | 0.095 |
| RST-03 | MMP12 | Predicted | 0.079 | FC-02 | TGM2 | Predicted | 0.005 |
| RST-03 | MMP9 | Predicted | 0.052 | FC-02 | TOP2A | Predicted | 0.052 |
| RST-03 | NME1 | Predicted | 0.069 | FC-02 | TRDMT1 | Predicted | 0.003 |
| RST-03 | NME2 | Predicted | 0.019 | FC-02 | VEGFA | Predicted | 0.055 |
| RST-03 | PGK1 | Predicted | 0.017 | FC-03 | ABCB1 | Predicted | 0.025 |
| RST-03 | PTGS2 | Predicted | 0.214 | FC-03 | ACSL1 | Predicted | 0.077 |
| RST-03 | PTK2 | Predicted | 0.017 | FC-03 | ADH1C | Predicted | 0.011 |
| RST-03 | RHOA | Predicted | 0.019 | FC-03 | AGXT | Predicted | 0.016 |
| RST-03 | RND3 | Predicted | 0.026 | FC-03 | AKR1B10 | Predicted | 0.253 |
| RST-03 | SERPINE1 | Predicted | 0.036 | FC-03 | AKT2 | Predicted | 0.365 |
| RST-03 | SGK1 | Predicted | 0.000 | FC-03 | ASS1 | Predicted | 0.025 |
| RST-03 | SRC | Predicted | 0.325 | FC-03 | AURKA | Predicted | 0.012 |
| RST-03 | SULT2A1 | Predicted | 0.027 | FC-03 | CA1 | Predicted | 0.192 |
| RST-03 | TGM2 | Predicted | 0.019 | FC-03 | CA2 | Predicted | 0.192 |
| RST-03 | TOP2A | Predicted | 0.132 | FC-03 | CASP1 | Predicted | 0.051 |
| RST-03 | TRDMT1 | Predicted | 0.011 | FC-03 | CCNA2 | Predicted | 0.783 |
| RST-03 | VEGFA | Predicted | 0.127 | FC-03 | CCT3 | Predicted | 0.012 |
| RST-04 | CASP3 | Validated | 1.000 | FC-03 | CDK4 | Predicted | 0.097 |
| RST-04 | ABCB1 | Predicted | 0.014 | FC-03 | CES1 | Predicted | 0.011 |
| RST-04 | ACSL1 | Predicted | 0.014 | FC-03 | CTSB | Predicted | 0.263 |
| RST-04 | ADH1B | Predicted | 0.009 | FC-03 | ESR1 | Predicted | 0.798 |
| RST-04 | ADH1C | Predicted | 0.019 | FC-03 | FGF1 | Predicted | 0.121 |
| RST-04 | ADH4 | Predicted | 0.009 | FC-03 | FGF2 | Predicted | 0.121 |
| RST-04 | AKR1C2 | Predicted | 0.001 | FC-03 | GABRE | Predicted | 0.008 |
| RST-04 | AKT2 | Predicted | 0.105 | FC-03 | GLUD1 | Predicted | 0.041 |
| RST-04 | ALDH1A1 | Predicted | 0.009 | FC-03 | GNAI1 | Predicted | 0.014 |
| RST-04 | ALDH1B1 | Predicted | 0.001 | FC-03 | GNMT | Predicted | 0.009 |
| RST-04 | ALDH1L1 | Predicted | 0.000 | FC-03 | GSTP1 | Predicted | 0.078 |
| RST-04 | ALDH2 | Predicted | 0.009 | FC-03 | HAO1 | Predicted | 0.000 |
| RST-04 | ALDH3A2 | Predicted | 0.001 | FC-03 | HSP90AA1 | Predicted | 0.012 |
| RST-04 | ALDH4A1 | Predicted | 0.001 | FC-03 | HSP90B1 | Predicted | 0.012 |
| RST-04 | ASS1 | Predicted | 0.014 | FC-03 | HSPA1B | Predicted | 0.012 |
| RST-04 | AURKA | Predicted | 0.006 | FC-03 | HSPA8 | Predicted | 0.012 |
| RST-04 | CASP1 | Predicted | 0.034 | FC-03 | KRAS | Predicted | 0.079 |
| RST-04 | CCNA2 | Predicted | 0.283 | FC-03 | MAPK1 | Predicted | 0.097 |
| RST-04 | CCT3 | Predicted | 0.021 | FC-03 | MAPK3 | Predicted | 0.203 |
| RST-04 | CDK4 | Predicted | 0.043 | FC-03 | MAT1A | Predicted | 0.012 |
| RST-04 | CES1 | Predicted | 0.003 | FC-03 | MME | Predicted | 0.174 |
| RST-04 | ESR1 | Predicted | 0.136 | FC-03 | MMP12 | Predicted | 0.080 |
| RST-04 | FTCD | Predicted | 0.000 | FC-03 | MMP9 | Predicted | 0.051 |
| RST-04 | GAPDH | Predicted | 0.029 | FC-03 | NME1 | Predicted | 0.062 |
| RST-04 | GLUD1 | Predicted | 0.034 | FC-03 | NME2 | Predicted | 0.014 |
| RST-04 | GNAI1 | Predicted | 0.004 | FC-03 | NOS2A | Predicted | 0.253 |
| RST-04 | GNMT | Predicted | 0.004 | FC-03 | PGK1 | Predicted | 0.012 |
| RST-04 | GSTP1 | Predicted | 0.115 | FC-03 | PTGS2 | Predicted | 0.201 |
| RST-04 | HAO1 | Predicted | 0.003 | FC-03 | PTK2 | Predicted | 0.012 |
| RST-04 | HSD17B10 | Predicted | 0.009 | FC-03 | RHOA | Predicted | 0.014 |
| RST-04 | HSD17B4 | Predicted | 0.009 | FC-03 | RND3 | Predicted | 0.017 |
| RST-04 | HSP90AA1 | Predicted | 0.006 | FC-03 | SERPINE1 | Predicted | 0.020 |
| RST-04 | HSP90B1 | Predicted | 0.006 | FC-03 | SGK1 | Predicted | 0.000 |
| RST-04 | HSPA1B | Predicted | 0.006 | FC-03 | SULT2A1 | Predicted | 0.024 |
| RST-04 | HSPA8 | Predicted | 0.006 | FC-03 | TGM2 | Predicted | 0.014 |
| RST-04 | KRAS | Predicted | 0.083 | FC-03 | TOP2A | Predicted | 0.128 |
| RST-04 | MAPK1 | Predicted | 0.120 | FC-03 | TRDMT1 | Predicted | 0.009 |
| RST-04 | MAPK3 | Predicted | 0.043 | FC-03 | VEGFA | Predicted | 0.132 |
| RST-04 | MAT1A | Predicted | 0.006 | FC-04 | ADH1C | Predicted | 0.018 |
| RST-04 | MET | Predicted | 0.134 | FC-04 | AGXT | Predicted | 0.024 |
| RST-04 | MME | Predicted | 0.209 | FC-04 | AKR1B10 | Predicted | 0.318 |
| RST-04 | MMP12 | Predicted | 0.054 | FC-04 | AKT2 | Predicted | 0.379 |
| RST-04 | MMP9 | Predicted | 0.034 | FC-04 | AURKA | Predicted | 0.017 |
| RST-04 | MTHFD1 | Predicted | 0.008 | FC-04 | CA1 | Predicted | 0.239 |
| RST-04 | MTR | Predicted | 0.000 | FC-04 | CA2 | Predicted | 0.239 |
| RST-04 | NME1 | Predicted | 0.028 | FC-04 | CASP1 | Predicted | 0.063 |
| RST-04 | NME2 | Predicted | 0.004 | FC-04 | CCNA2 | Predicted | 0.970 |
| RST-04 | PGK1 | Predicted | 0.006 | FC-04 | CCT3 | Predicted | 0.017 |
| RST-04 | PTK2 | Predicted | 0.006 | FC-04 | CDK4 | Predicted | 0.126 |
| RST-04 | QDPR | Predicted | 0.009 | FC-04 | CES1 | Predicted | 0.018 |
| RST-04 | RHOA | Predicted | 0.004 | FC-04 | CTSB | Predicted | 0.326 |
| RST-04 | RND3 | Predicted | 0.005 | FC-04 | ESR1 | Predicted | 0.659 |
| RST-04 | SERPINE1 | Predicted | 0.009 | FC-04 | FGF1 | Predicted | 0.154 |
| RST-04 | SGK1 | Predicted | 0.003 | FC-04 | FGF2 | Predicted | 0.154 |
| RST-04 | SRC | Predicted | 0.065 | FC-04 | GABRE | Predicted | 0.009 |
| RST-04 | TGM2 | Predicted | 0.004 | FC-04 | GLUD1 | Predicted | 0.047 |
| RST-04 | TOP2A | Predicted | 0.201 | FC-04 | GNAI1 | Predicted | 0.019 |
| RST-04 | TRDMT1 | Predicted | 0.004 | FC-04 | GNMT | Predicted | 0.008 |
| RST-04 | TUBB | Predicted | 0.006 | FC-04 | HAO1 | Predicted | 0.000 |
| RST-04 | VEGFA | Predicted | 0.065 | FC-04 | HSP90AA1 | Predicted | 0.017 |
| RST-05 | ABCB1 | Predicted | 0.014 | FC-04 | HSP90B1 | Predicted | 0.017 |
| RST-05 | ACSL1 | Predicted | 0.014 | FC-04 | HSPA1B | Predicted | 0.017 |
| RST-05 | AKT2 | Predicted | 0.114 | FC-04 | HSPA8 | Predicted | 0.017 |
| RST-05 | ALDH1L1 | Predicted | 0.012 | FC-04 | KRAS | Predicted | 0.101 |
| RST-05 | ASS1 | Predicted | 0.014 | FC-04 | MAPK1 | Predicted | 0.126 |
| RST-05 | AURKA | Predicted | 0.117 | FC-04 | MAPK3 | Predicted | 0.259 |
| RST-05 | CA1 | Predicted | 0.019 | FC-04 | MAT1A | Predicted | 0.017 |
| RST-05 | CA2 | Predicted | 0.019 | FC-04 | MME | Predicted | 0.218 |
| RST-05 | CCNA2 | Predicted | 0.861 | FC-04 | MMP12 | Predicted | 0.103 |
| RST-05 | CCT3 | Predicted | 0.008 | FC-04 | MMP9 | Predicted | 0.063 |
| RST-05 | CYP2C8 | Predicted | 0.012 | FC-04 | NME1 | Predicted | 0.073 |
| RST-05 | EGFR | Predicted | 0.121 | FC-04 | NME2 | Predicted | 0.019 |
| RST-05 | ESR1 | Predicted | 0.223 | FC-04 | PGK1 | Predicted | 0.017 |
| RST-05 | FTCD | Predicted | 0.012 | FC-04 | PTGS2 | Predicted | 0.123 |
| RST-05 | GLUD1 | Predicted | 0.009 | FC-04 | PTK2 | Predicted | 0.017 |
| RST-05 | GNMT | Predicted | 0.005 | FC-04 | RHOA | Predicted | 0.019 |
| RST-05 | GSTP1 | Predicted | 0.104 | FC-04 | RND3 | Predicted | 0.020 |
| RST-05 | KRAS | Predicted | 0.058 | FC-04 | SERPINE1 | Predicted | 0.029 |
| RST-05 | MAPK1 | Predicted | 0.011 | FC-04 | SGK1 | Predicted | 0.000 |
| RST-05 | MME | Predicted | 0.292 | FC-04 | SULT2A1 | Predicted | 0.033 |
| RST-05 | MTHFD1 | Predicted | 0.012 | FC-04 | TGM2 | Predicted | 0.019 |
| RST-05 | MTR | Predicted | 0.012 | FC-04 | TOP2A | Predicted | 0.174 |
| RST-05 | NME1 | Predicted | 0.118 | FC-04 | TRDMT1 | Predicted | 0.008 |
| RST-05 | NOS2A | Predicted | 0.120 | FC-04 | VEGFA | Predicted | 0.147 |
| RST-05 | PTGS2 | Predicted | 0.059 | FC-05 | ABCB1 | Predicted | 0.155 |
| RST-05 | RAF1 | Predicted | 0.015 | FC-05 | ACSL1 | Predicted | 0.000 |
| RST-05 | RARA | Predicted | 0.016 | FC-05 | ADH1C | Predicted | 0.010 |
| RST-05 | RND3 | Predicted | 0.009 | FC-05 | AGXT | Predicted | 0.002 |
| RST-05 | SERPINE1 | Predicted | 0.029 | FC-05 | AKR1B10 | Predicted | 0.193 |
| RST-05 | TRDMT1 | Predicted | 0.005 | FC-05 | AKT2 | Predicted | 0.010 |
| RST-05 | TUBB | Predicted | 0.006 | FC-05 | ASS1 | Predicted | 0.012 |
| RST-05 | VEGFA | Predicted | 0.026 | FC-05 | AURKA | Predicted | 0.011 |
| RST-06 | ALDH1L1 | Predicted | 0.000 | FC-05 | CA1 | Predicted | 0.031 |
| RST-06 | CASP3 | Validated | 1.000 | FC-05 | CA2 | Predicted | 0.010 |
| RST-06 | DDR1 | Predicted | 0.038 | FC-05 | CASP1 | Predicted | 0.147 |
| RST-06 | EGFR | Predicted | 0.316 | FC-05 | CCNA2 | Predicted | 0.609 |
| RST-06 | FTCD | Predicted | 0.000 | FC-05 | CCT3 | Predicted | 0.060 |
| RST-06 | ICAM1 | Validated | 1.000 | FC-05 | CDK4 | Predicted | 0.031 |
| RST-06 | MTHFD1 | Predicted | 0.000 | FC-05 | CES1 | Predicted | 0.147 |
| RST-06 | MTR | Predicted | 0.000 | FC-05 | CTSB | Predicted | 0.020 |
| RST-06 | PDGFRA | Predicted | 0.038 | FC-05 | ESR1 | Predicted | 0.093 |
| RST-06 | PTGS2 | Validated | 1.000 | FC-05 | FGF1 | Predicted | 0.039 |
| RST-06 | RARA | Predicted | 0.047 | FC-05 | FGF2 | Predicted | 0.048 |
| RST-06 | SRC | Predicted | 0.037 | FC-05 | GABRE | Predicted | 0.010 |
| RST-07 | ABCB1 | Predicted | 0.005 | FC-05 | GLUD1 | Predicted | 0.010 |
| RST-07 | ACSL1 | Predicted | 0.005 | FC-05 | GNAI1 | Predicted | 0.060 |
| RST-07 | ADH1C | Predicted | 0.005 | FC-05 | GNMT | Predicted | 0.133 |
| RST-07 | AGXT | Predicted | 0.006 | FC-05 | GSTP1 | Predicted | 0.020 |
| RST-07 | AKR1B10 | Predicted | 0.089 | FC-05 | HAO1 | Predicted | 0.103 |
| RST-07 | AKT2 | Predicted | 0.045 | FC-05 | HSP90AA1 | Predicted | 0.010 |
| RST-07 | ALDH1A1 | Predicted | 0.017 | FC-05 | HSP90B1 | Predicted | 0.074 |
| RST-07 | ASS1 | Predicted | 0.005 | FC-05 | HSPA1B | Predicted | 0.010 |
| RST-07 | AURKA | Predicted | 0.004 | FC-05 | HSPA8 | Predicted | 0.097 |
| RST-07 | CA1 | Predicted | 0.043 | FC-05 | KRAS | Predicted | 0.093 |
| RST-07 | CA2 | Predicted | 0.043 | FC-05 | MAPK1 | Validated | 1.000 |
| RST-07 | CCNA2 | Predicted | 1.000 | FC-05 | MAPK3 | Predicted | 0.012 |
| RST-07 | CCT3 | Predicted | 0.012 | FC-05 | MAT1A | Predicted | 0.011 |
| RST-07 | CDK4 | Predicted | 0.043 | FC-05 | MME | Predicted | 0.074 |
| RST-07 | CES1 | Predicted | 0.005 | FC-05 | MMP12 | Predicted | 1.000 |
| RST-07 | CTSB | Predicted | 0.088 | FC-05 | MMP9 | Predicted | 0.012 |
| RST-07 | ESR1 | Predicted | 0.178 | FC-05 | NME1 | Predicted | 0.279 |
| RST-07 | GLUD1 | Predicted | 0.014 | FC-05 | NME2 | Predicted | 0.063 |
| RST-07 | GNAI1 | Predicted | 0.005 | FC-05 | NOS2A | Predicted | 0.039 |
| RST-07 | GNMT | Predicted | 0.004 | FC-05 | PGK1 | Predicted | 0.194 |
| RST-07 | GSTP1 | Predicted | 0.027 | FC-05 | PTGS2 | Validated | 1.000 |
| RST-07 | HAO1 | Predicted | 0.000 | FC-05 | PTK2 | Predicted | 0.202 |
| RST-07 | HSP90AA1 | Predicted | 0.004 | FC-05 | RHOA | Predicted | 0.011 |
| RST-07 | HSP90B1 | Predicted | 0.004 | FC-05 | RND3 | Predicted | 0.013 |
| RST-07 | HSPA1B | Predicted | 0.004 | FC-05 | SERPINE1 | Predicted | 0.153 |
| RST-07 | HSPA8 | Predicted | 0.004 | FC-05 | SGK1 | Predicted | 0.003 |
| RST-07 | KRAS | Predicted | 0.052 | FC-05 | SULT2A1 | Predicted | 0.010 |
| RST-07 | LYZ | Predicted | 0.087 | FC-05 | TGM2 | Predicted | 0.018 |
| RST-07 | MAPK1 | Predicted | 0.089 | FC-05 | TOP2A | Predicted | 0.010 |
| RST-07 | MAPK3 | Predicted | 0.043 | FC-05 | TRDMT1 | Predicted | 0.002 |
| RST-07 | MAT1A | Predicted | 0.004 | FC-05 | VEGFA | Predicted | 0.000 |
| RST-07 | MME | Predicted | 0.135 | FC-06 | ABCB1 | Predicted | 0.010 |
| RST-07 | NME1 | Predicted | 0.020 | FC-06 | ACSL1 | Predicted | 0.010 |
| RST-07 | NME2 | Predicted | 0.005 | FC-06 | ADH1C | Predicted | 0.010 |
| RST-07 | PGK1 | Predicted | 0.004 | FC-06 | AGXT | Predicted | 0.010 |
| RST-07 | PTGS2 | Predicted | 0.022 | FC-06 | AKR1B10 | Predicted | 0.163 |
| RST-07 | PTK2 | Predicted | 0.004 | FC-06 | AKT2 | Predicted | 0.231 |
| RST-07 | RHOA | Predicted | 0.005 | FC-06 | ASS1 | Predicted | 0.010 |
| RST-07 | RND3 | Predicted | 0.007 | FC-06 | AURKA | Predicted | 0.008 |
| RST-07 | SERPINE1 | Predicted | 0.016 | FC-06 | CA1 | Predicted | 0.121 |
| RST-07 | SGK1 | Predicted | 0.000 | FC-06 | CA2 | Predicted | 0.121 |
| RST-07 | SRC | Predicted | 0.119 | FC-06 | CASP1 | Predicted | 0.033 |
| RST-07 | TGM2 | Predicted | 0.005 | FC-06 | CCNA2 | Predicted | 1.000 |
| RST-07 | TOP2A | Predicted | 0.023 | FC-06 | CCT3 | Predicted | 0.008 |
| RST-07 | TRDMT1 | Predicted | 0.004 | FC-06 | CDK4 | Predicted | 0.055 |
| RST-07 | VEGFA | Predicted | 0.026 | FC-06 | CES1 | Predicted | 0.010 |
| RST-09 | ACSL1 | Predicted | 0.024 | FC-06 | CTSB | Predicted | 0.163 |
| RST-09 | ALDH2 | Predicted | 0.084 | FC-06 | ESR1 | Predicted | 0.342 |
| RST-09 | ALDOA | Predicted | 0.084 | FC-06 | GABRE | Predicted | 0.002 |
| RST-09 | CA1 | Predicted | 0.166 | FC-06 | GLUD1 | Predicted | 0.029 |
| RST-09 | CA2 | Predicted | 0.150 | FC-06 | GNAI1 | Predicted | 0.011 |
| RST-09 | CCNA2 | Predicted | 0.085 | FC-06 | GNMT | Predicted | 0.005 |
| RST-09 | ESR1 | Predicted | 0.013 | FC-06 | GSTP1 | Predicted | 0.050 |
| RST-09 | GABRE | Predicted | 0.022 | FC-06 | HAO1 | Predicted | 0.000 |
| RST-09 | GSTP1 | Predicted | 0.000 | FC-06 | HSP90AA1 | Predicted | 0.008 |
| RST-09 | KRAS | Predicted | 0.016 | FC-06 | HSP90B1 | Predicted | 0.008 |
| RST-09 | MAPK1 | Predicted | 0.027 | FC-06 | HSPA1B | Predicted | 0.008 |
| RST-09 | MTAP | Predicted | 0.181 | FC-06 | HSPA8 | Predicted | 0.008 |
| RST-09 | NOS2A | Predicted | 0.117 | FC-06 | KRAS | Predicted | 0.050 |
| RST-09 | PARP1 | Predicted | 0.089 | FC-06 | MAPK1 | Predicted | 0.055 |
| RST-09 | PC | Predicted | 0.032 | FC-06 | MAPK3 | Predicted | 0.120 |
| RST-09 | PIN1 | Predicted | 0.040 | FC-06 | MAT1A | Predicted | 0.008 |
| RST-09 | PTGS2 | Predicted | 1.000 | FC-06 | MME | Predicted | 0.109 |
| RST-09 | SRC | Predicted | 0.039 | FC-06 | MMP12 | Predicted | 0.051 |
| RST-09 | TNFSF11 | Predicted | 0.026 | FC-06 | MMP9 | Predicted | 0.033 |
| RST-10 | ADH1C | Predicted | 0.020 | FC-06 | NME1 | Predicted | 0.043 |
| RST-10 | AGXT | Predicted | 0.012 | FC-06 | NME2 | Predicted | 0.011 |
| RST-10 | AKR1B10 | Predicted | 0.170 | FC-06 | NOS2A | Predicted | 0.159 |
| RST-10 | AKT2 | Predicted | 0.104 | FC-06 | PGK1 | Predicted | 0.008 |
| RST-10 | ALDH1A1 | Predicted | 0.039 | FC-06 | PTGS2 | Predicted | 0.122 |
| RST-10 | AURKA | Predicted | 0.009 | FC-06 | PTK2 | Predicted | 0.008 |
| RST-10 | CA1 | Predicted | 0.080 | FC-06 | RHOA | Predicted | 0.011 |
| RST-10 | CA2 | Predicted | 0.080 | FC-06 | RND3 | Predicted | 0.014 |
| RST-10 | CCNA2 | Predicted | 1.000 | FC-06 | SERPINE1 | Predicted | 0.026 |
| RST-10 | CCT3 | Predicted | 0.009 | FC-06 | SGK1 | Predicted | 0.000 |
| RST-10 | CES1 | Predicted | 0.020 | FC-06 | SRC | Predicted | 0.159 |
| RST-10 | CTSB | Predicted | 0.170 | FC-06 | SULT2A1 | Predicted | 0.015 |
| RST-10 | ESR1 | Predicted | 0.334 | FC-06 | TGM2 | Predicted | 0.011 |
| RST-10 | GLUD1 | Predicted | 0.025 | FC-06 | TOP2A | Predicted | 0.088 |
| RST-10 | GNAI1 | Predicted | 0.009 | FC-06 | TRDMT1 | Predicted | 0.005 |
| RST-10 | GNMT | Predicted | 0.007 | FC-06 | VEGFA | Predicted | 0.089 |
| RST-10 | GSTP1 | Predicted | 0.051 | FC-07 | ABCB1 | Predicted | 0.013 |
| RST-10 | HAO1 | Predicted | 0.000 | FC-07 | ACSL1 | Predicted | 0.013 |
| RST-10 | HSP90AA1 | Predicted | 0.009 | FC-07 | ADH1C | Predicted | 0.012 |
| RST-10 | HSP90B1 | Predicted | 0.009 | FC-07 | AGXT | Predicted | 0.013 |
| RST-10 | HSPA1B | Predicted | 0.009 | FC-07 | AKR1B10 | Predicted | 0.164 |
| RST-10 | HSPA8 | Predicted | 0.009 | FC-07 | AKT2 | Predicted | 0.234 |
| RST-10 | KRAS | Predicted | 0.096 | FC-07 | ASS1 | Predicted | 0.013 |
| RST-10 | LYZ | Predicted | 0.163 | FC-07 | AURKA | Predicted | 0.011 |
| RST-10 | MAPK1 | Predicted | 0.161 | FC-07 | CA1 | Predicted | 0.081 |
| RST-10 | MAT1A | Predicted | 0.009 | FC-07 | CA2 | Predicted | 0.081 |
| RST-10 | MME | Predicted | 0.110 | FC-07 | CASP1 | Predicted | 0.037 |
| RST-10 | NME1 | Predicted | 0.037 | FC-07 | CASP3 | Validated | 1.000 |
| RST-10 | NME2 | Predicted | 0.009 | FC-07 | CCNA2 | Predicted | 1.000 |
| RST-10 | NOS2A | Predicted | 0.162 | FC-07 | CCT3 | Predicted | 0.011 |
| RST-10 | PGK1 | Predicted | 0.009 | FC-07 | CDK4 | Predicted | 0.059 |
| RST-10 | PTK2 | Predicted | 0.009 | FC-07 | CES1 | Predicted | 0.012 |
| RST-10 | RARA | Predicted | 0.041 | FC-07 | CTSB | Predicted | 0.167 |
| RST-10 | RHOA | Predicted | 0.009 | FC-07 | ESR1 | Predicted | 0.176 |
| RST-10 | RND3 | Predicted | 0.011 | FC-07 | GABRE | Predicted | 0.004 |
| RST-10 | SERPINE1 | Predicted | 0.031 | FC-07 | GLUD1 | Predicted | 0.029 |
| RST-10 | SGK1 | Predicted | 0.000 | FC-07 | GNAI1 | Predicted | 0.011 |
| RST-10 | SRC | Predicted | 0.166 | FC-07 | GNMT | Predicted | 0.007 |
| RST-10 | TGM2 | Predicted | 0.009 | FC-07 | HAO1 | Predicted | 0.000 |
| RST-10 | TOP2A | Predicted | 0.037 | FC-07 | HSP90AA1 | Predicted | 0.011 |
| RST-10 | TRDMT1 | Predicted | 0.007 | FC-07 | HSP90B1 | Predicted | 0.011 |
| RST-10 | VEGFA | Predicted | 0.048 | FC-07 | HSPA1B | Predicted | 0.011 |
| RST-11 | ABCB1 | Predicted | 0.003 | FC-07 | HSPA8 | Predicted | 0.011 |
| RST-11 | ACSL1 | Predicted | 0.003 | FC-07 | ICAM1 | Validated | 1.000 |
| RST-11 | AGXT | Predicted | 0.002 | FC-07 | KRAS | Predicted | 0.052 |
| RST-11 | AKR1B10 | Predicted | 0.073 | FC-07 | MAPK1 | Predicted | 0.059 |
| RST-11 | AKT2 | Predicted | 0.031 | FC-07 | MAPK3 | Predicted | 0.120 |
| RST-11 | ALDH1A1 | Predicted | 0.018 | FC-07 | MAT1A | Predicted | 0.011 |
| RST-11 | ASS1 | Predicted | 0.003 | FC-07 | MME | Predicted | 0.113 |
| RST-11 | AURKA | Predicted | 0.001 | FC-07 | MMP12 | Predicted | 0.053 |
| RST-11 | CA1 | Predicted | 0.033 | FC-07 | MMP9 | Predicted | 0.037 |
| RST-11 | CA2 | Predicted | 0.033 | FC-07 | NME1 | Predicted | 0.043 |
| RST-11 | CCNA2 | Predicted | 1.000 | FC-07 | NME2 | Predicted | 0.011 |
| RST-11 | CCT3 | Predicted | 0.001 | FC-07 | PGK1 | Predicted | 0.011 |
| RST-11 | CTSB | Predicted | 0.070 | FC-07 | PTGS2 | Predicted | 0.055 |
| RST-11 | CYP2C8 | Predicted | 0.003 | FC-07 | PTK2 | Predicted | 0.011 |
| RST-11 | ESR1 | Predicted | 0.072 | FC-07 | RHOA | Predicted | 0.011 |
| RST-11 | GLUD1 | Predicted | 0.008 | FC-07 | RND3 | Predicted | 0.013 |
| RST-11 | GNAI1 | Predicted | 0.001 | FC-07 | SERPINE1 | Predicted | 0.028 |
| RST-11 | GNMT | Predicted | 0.002 | FC-07 | SGK1 | Predicted | 0.000 |
| RST-11 | GSTP1 | Predicted | 0.002 | FC-07 | SRC | Predicted | 0.162 |
| RST-11 | HSP90AA1 | Predicted | 0.001 | FC-07 | SULT2A1 | Predicted | 0.018 |
| RST-11 | HSP90B1 | Predicted | 0.001 | FC-07 | TGM2 | Predicted | 0.011 |
| RST-11 | HSPA1B | Predicted | 0.001 | FC-07 | TOP2A | Predicted | 0.089 |
| RST-11 | HSPA8 | Predicted | 0.001 | FC-07 | TRDMT1 | Predicted | 0.007 |
| RST-11 | KRAS | Predicted | 0.028 | FC-07 | VEGFA | Predicted | 0.088 |
| RST-11 | LYZ | Predicted | 0.074 | FC-08 | CASP3 | Validated | 1.000 |
| RST-11 | MAPK1 | Predicted | 0.070 | FC-08 | ABCB1 | Predicted | 0.014 |
| RST-11 | MAT1A | Predicted | 0.001 | FC-08 | ACSL1 | Predicted | 0.014 |
| RST-11 | MME | Predicted | 0.070 | FC-08 | ADH1B | Predicted | 0.009 |
| RST-11 | NME1 | Predicted | 0.014 | FC-08 | ADH1C | Predicted | 0.019 |
| RST-11 | NME2 | Predicted | 0.001 | FC-08 | ADH4 | Predicted | 0.009 |
| RST-11 | NOS2A | Predicted | 0.146 | FC-08 | AKR1C2 | Predicted | 0.001 |
| RST-11 | PGK1 | Predicted | 0.001 | FC-08 | AKT2 | Predicted | 0.105 |
| RST-11 | PTGS2 | Predicted | 0.063 | FC-08 | ALDH1A1 | Predicted | 0.009 |
| RST-11 | PTK2 | Predicted | 0.001 | FC-08 | ALDH1B1 | Predicted | 0.001 |
| RST-11 | RARA | Predicted | 0.012 | FC-08 | ALDH1L1 | Predicted | 0.000 |
| RST-11 | RBP1 | Predicted | 0.004 | FC-08 | ALDH2 | Predicted | 0.009 |
| RST-11 | RHOA | Predicted | 0.001 | FC-08 | ALDH3A2 | Predicted | 0.001 |
| RST-11 | RND3 | Predicted | 0.002 | FC-08 | ALDH4A1 | Predicted | 0.001 |
| RST-11 | SERPINE1 | Predicted | 0.016 | FC-08 | ASS1 | Predicted | 0.014 |
| RST-11 | SRC | Predicted | 0.072 | FC-08 | AURKA | Predicted | 0.006 |
| RST-11 | TGM2 | Predicted | 0.001 | FC-08 | CASP1 | Predicted | 0.034 |
| RST-11 | TOP2A | Predicted | 0.018 | FC-08 | CCNA2 | Predicted | 0.283 |
| RST-11 | TRDMT1 | Predicted | 0.002 | FC-08 | CCT3 | Predicted | 0.021 |
| RST-12 | ACSL1 | Predicted | 0.033 | FC-08 | CDK4 | Predicted | 0.043 |
| RST-12 | AURKA | Predicted | 0.001 | FC-08 | CES1 | Predicted | 0.003 |
| RST-12 | CA1 | Predicted | 0.203 | FC-08 | ESR1 | Predicted | 0.136 |
| RST-12 | CA2 | Predicted | 0.276 | FC-08 | FTCD | Predicted | 0.000 |
| RST-12 | CCNA2 | Predicted | 0.495 | FC-08 | GAPDH | Predicted | 0.029 |
| RST-12 | CCT3 | Predicted | 0.001 | FC-08 | GLUD1 | Predicted | 0.034 |
| RST-12 | CTSB | Predicted | 0.121 | FC-08 | GNAI1 | Predicted | 0.004 |
| RST-12 | ESR1 | Predicted | 0.055 | FC-08 | GNMT | Predicted | 0.004 |
| RST-12 | GABRE | Predicted | 0.032 | FC-08 | GSTP1 | Predicted | 0.115 |
| RST-12 | GLUD1 | Predicted | 0.001 | FC-08 | HAO1 | Predicted | 0.003 |
| RST-12 | GNAI1 | Predicted | 0.002 | FC-08 | HSD17B10 | Predicted | 0.009 |
| RST-12 | GNMT | Predicted | 0.009 | FC-08 | HSD17B4 | Predicted | 0.009 |
| RST-12 | GSTP1 | Predicted | 0.000 | FC-08 | HSP90AA1 | Predicted | 0.006 |
| RST-12 | HSP90AA1 | Predicted | 0.001 | FC-08 | HSP90B1 | Predicted | 0.006 |
| RST-12 | HSP90B1 | Predicted | 0.001 | FC-08 | HSPA1B | Predicted | 0.006 |
| RST-12 | HSPA1B | Predicted | 0.001 | FC-08 | HSPA8 | Predicted | 0.006 |
| RST-12 | HSPA8 | Predicted | 0.001 | FC-08 | KRAS | Predicted | 0.083 |
| RST-12 | IGFBP1 | Validated | 1.000 | FC-08 | MAPK1 | Predicted | 0.120 |
| RST-12 | KRAS | Predicted | 0.033 | FC-08 | MAPK3 | Predicted | 0.043 |
| RST-12 | MAT1A | Predicted | 0.001 | FC-08 | MAT1A | Predicted | 0.006 |
| RST-12 | MMP14 | Predicted | 0.005 | FC-08 | MET | Predicted | 0.134 |
| RST-12 | MMP2 | Predicted | 0.005 | FC-08 | MME | Predicted | 0.209 |
| RST-12 | MMP3 | Predicted | 0.005 | FC-08 | MMP12 | Predicted | 0.054 |
| RST-12 | MMP7 | Predicted | 0.005 | FC-08 | MMP9 | Predicted | 0.034 |
| RST-12 | MMP9 | Predicted | 0.005 | FC-08 | MTHFD1 | Predicted | 0.008 |
| RST-12 | MTAP | Predicted | 0.254 | FC-08 | MTR | Predicted | 0.000 |
| RST-12 | NME1 | Predicted | 0.009 | FC-08 | NME1 | Predicted | 0.028 |
| RST-12 | NME2 | Predicted | 0.002 | FC-08 | NME2 | Predicted | 0.004 |
| RST-12 | NOS2A | Predicted | 0.118 | FC-08 | PGK1 | Predicted | 0.006 |
| RST-12 | PARP1 | Predicted | 0.120 | FC-08 | PTK2 | Predicted | 0.006 |
| RST-12 | PGK1 | Predicted | 0.001 | FC-08 | QDPR | Predicted | 0.009 |
| RST-12 | PTGS2 | Predicted | 0.998 | FC-08 | RHOA | Predicted | 0.004 |
| RST-12 | PTGS2 | Validated | 1.000 | FC-08 | RND3 | Predicted | 0.005 |
| RST-12 | PTK2 | Predicted | 0.001 | FC-08 | SERPINE1 | Predicted | 0.009 |
| RST-12 | RHOA | Predicted | 0.002 | FC-08 | SGK1 | Predicted | 0.003 |
| RST-12 | SRC | Predicted | 0.119 | FC-08 | SRC | Predicted | 0.065 |
| RST-12 | TGM2 | Predicted | 0.002 | FC-08 | TGM2 | Predicted | 0.004 |
| RST-12 | TOP2A | Predicted | 0.035 | FC-08 | TOP2A | Predicted | 0.201 |
| RST-12 | TRDMT1 | Predicted | 0.009 | FC-08 | TRDMT1 | Predicted | 0.004 |
| RST-13 | ABCB1 | Predicted | 0.012 | FC-08 | TUBB | Predicted | 0.006 |
| RST-13 | ACSL1 | Predicted | 0.045 | FC-08 | VEGFA | Predicted | 0.065 |
| RST-13 | AGXT | Predicted | 0.011 | FC-09 | ABCB1 | Predicted | 0.012 |
| RST-13 | AKT2 | Predicted | 0.016 | FC-09 | ACSL1 | Predicted | 0.012 |
| RST-13 | AR | Predicted | 0.108 | FC-09 | ADH1C | Predicted | 0.008 |
| RST-13 | ASS1 | Predicted | 0.012 | FC-09 | AGXT | Predicted | 0.011 |
| RST-13 | AURKA | Predicted | 0.008 | FC-09 | AKR1B10 | Predicted | 0.193 |
| RST-13 | CA1 | Predicted | 0.060 | FC-09 | AKT2 | Predicted | 0.283 |
| RST-13 | CA2 | Predicted | 0.060 | FC-09 | ASS1 | Predicted | 0.012 |
| RST-13 | CCNA2 | Predicted | 1.000 | FC-09 | AURKA | Predicted | 0.009 |
| RST-13 | CCT3 | Predicted | 0.025 | FC-09 | CA1 | Predicted | 0.092 |
| RST-13 | CDK4 | Predicted | 0.050 | FC-09 | CA2 | Predicted | 0.092 |
| RST-13 | CES1 | Predicted | 0.059 | FC-09 | CCNA2 | Predicted | 1.000 |
| RST-13 | CTSB | Predicted | 0.194 | FC-09 | CCT3 | Predicted | 0.028 |
| RST-13 | EGFR | Predicted | 0.050 | FC-09 | CDK4 | Predicted | 0.077 |
| RST-13 | ESR1 | Predicted | 0.583 | FC-09 | CES1 | Predicted | 0.008 |
| RST-13 | GABRE | Predicted | 0.031 | FC-09 | CTSB | Predicted | 0.200 |
| RST-13 | GLUD1 | Predicted | 0.024 | FC-09 | ESR1 | Predicted | 0.198 |
| RST-13 | GNAI1 | Predicted | 0.006 | FC-09 | GLUD1 | Predicted | 0.030 |
| RST-13 | GNMT | Predicted | 0.011 | FC-09 | GNAI1 | Predicted | 0.009 |
| RST-13 | HAO1 | Predicted | 0.000 | FC-09 | GNMT | Predicted | 0.006 |
| RST-13 | HSP90AA1 | Predicted | 0.008 | FC-09 | GSTP1 | Predicted | 0.061 |
| RST-13 | HSP90B1 | Predicted | 0.008 | FC-09 | HAO1 | Predicted | 0.000 |
| RST-13 | HSPA1B | Predicted | 0.008 | FC-09 | HSP90AA1 | Predicted | 0.009 |
| RST-13 | HSPA8 | Predicted | 0.008 | FC-09 | HSP90B1 | Predicted | 0.009 |
| RST-13 | KRAS | Predicted | 0.121 | FC-09 | HSPA1B | Predicted | 0.009 |
| RST-13 | LYZ | Predicted | 0.249 | FC-09 | HSPA8 | Predicted | 0.009 |
| RST-13 | MAT1A | Predicted | 0.008 | FC-09 | KRAS | Predicted | 0.134 |
| RST-13 | MMP12 | Predicted | 0.062 | FC-09 | MAPK1 | Predicted | 0.164 |
| RST-13 | MMP14 | Predicted | 0.020 | FC-09 | MAPK3 | Predicted | 0.077 |
| RST-13 | MMP2 | Predicted | 0.020 | FC-09 | MAT1A | Predicted | 0.009 |
| RST-13 | MMP3 | Predicted | 0.054 | FC-09 | MME | Predicted | 0.301 |
| RST-13 | MMP7 | Predicted | 0.020 | FC-09 | MMP12 | Predicted | 0.078 |
| RST-13 | MMP9 | Predicted | 0.020 | FC-09 | NME1 | Predicted | 0.046 |
| RST-13 | NME1 | Predicted | 0.038 | FC-09 | NME2 | Predicted | 0.009 |
| RST-13 | NME2 | Predicted | 0.006 | FC-09 | PGK1 | Predicted | 0.009 |
| RST-13 | NOS2A | Predicted | 0.054 | FC-09 | PLAU | Predicted | 0.125 |
| RST-13 | PGK1 | Predicted | 0.008 | FC-09 | PTK2 | Predicted | 0.009 |
| RST-13 | PTGS2 | Predicted | 0.294 | FC-09 | RHOA | Predicted | 0.009 |
| RST-13 | PTK2 | Predicted | 0.008 | FC-09 | RND3 | Predicted | 0.012 |
| RST-13 | RHOA | Predicted | 0.006 | FC-09 | SERPINE1 | Predicted | 0.015 |
| RST-13 | RND3 | Predicted | 0.007 | FC-09 | SGK1 | Predicted | 0.000 |
| RST-13 | SGK1 | Predicted | 0.000 | FC-09 | SRC | Predicted | 0.193 |
| RST-13 | SRC | Predicted | 0.189 | FC-09 | TGM2 | Predicted | 0.009 |
| RST-13 | SULT2A1 | Predicted | 0.018 | FC-09 | TOP2A | Predicted | 0.105 |
| RST-13 | TGM2 | Predicted | 0.006 | FC-09 | TRDMT1 | Predicted | 0.006 |
| RST-13 | TOP2A | Predicted | 0.042 | FC-09 | VEGFA | Predicted | 0.040 |
| RST-13 | TRDMT1 | Predicted | 0.011 | FC-10 | ABCB1 | Predicted | 0.020 |
| RST-14 | ACSL1 | Predicted | 0.024 | FC-10 | ACSL1 | Predicted | 0.020 |
| RST-14 | AGXT | Predicted | 0.004 | FC-10 | ADH1C | Predicted | 0.018 |
| RST-14 | ALDOA | Predicted | 0.133 | FC-10 | AGXT | Predicted | 0.019 |
| RST-14 | AURKA | Predicted | 0.000 | FC-10 | AKR1B10 | Predicted | 0.245 |
| RST-14 | CA1 | Predicted | 0.040 | FC-10 | AKT2 | Predicted | 0.353 |
| RST-14 | CA2 | Predicted | 0.040 | FC-10 | ASS1 | Predicted | 0.020 |
| RST-14 | CCNA2 | Predicted | 0.900 | FC-10 | AURKA | Predicted | 0.015 |
| RST-14 | CCT3 | Predicted | 0.000 | FC-10 | CA1 | Predicted | 0.184 |
| RST-14 | CES1 | Predicted | 0.038 | FC-10 | CA2 | Predicted | 0.184 |
| RST-14 | CTSB | Predicted | 0.136 | FC-10 | CASP1 | Predicted | 0.042 |
| RST-14 | ESR1 | Predicted | 0.193 | FC-10 | CCT3 | Predicted | 0.015 |
| RST-14 | GABRE | Predicted | 0.016 | FC-10 | CDK4 | Predicted | 0.074 |
| RST-14 | GLUD1 | Predicted | 0.000 | FC-10 | CES1 | Predicted | 0.018 |
| RST-14 | GNAI1 | Predicted | 0.002 | FC-10 | CTSB | Predicted | 0.255 |
| RST-14 | GSTP1 | Predicted | 0.001 | FC-10 | ESR1 | Predicted | 0.518 |
| RST-14 | HSP90AA1 | Predicted | 0.000 | FC-10 | FGF1 | Predicted | 0.119 |
| RST-14 | HSP90B1 | Predicted | 0.000 | FC-10 | FGF2 | Predicted | 0.119 |
| RST-14 | HSPA1B | Predicted | 0.000 | FC-10 | GABRE | Predicted | 0.007 |
| RST-14 | HSPA8 | Predicted | 0.000 | FC-10 | GLUD1 | Predicted | 0.042 |
| RST-14 | KRAS | Predicted | 0.037 | FC-10 | GNAI1 | Predicted | 0.017 |
| RST-14 | MAPK3 | Predicted | 0.079 | FC-10 | GNMT | Predicted | 0.006 |
| RST-14 | MAT1A | Predicted | 0.000 | FC-10 | GSTP1 | Predicted | 0.078 |
| RST-14 | MMP12 | Predicted | 0.038 | FC-10 | HAO1 | Predicted | 0.000 |
| RST-14 | MMP3 | Predicted | 0.038 | FC-10 | HSP90AA1 | Predicted | 0.015 |
| RST-14 | MTAP | Predicted | 0.284 | FC-10 | HSP90B1 | Predicted | 0.015 |
| RST-14 | NME1 | Predicted | 0.012 | FC-10 | HSPA1B | Predicted | 0.015 |
| RST-14 | NME2 | Predicted | 0.002 | FC-10 | HSPA8 | Predicted | 0.015 |
| RST-14 | NOS2A | Predicted | 0.136 | FC-10 | KRAS | Predicted | 0.079 |
| RST-14 | PGK1 | Predicted | 0.000 | FC-10 | MAPK1 | Predicted | 0.074 |
| RST-14 | PTGS2 | Predicted | 0.449 | FC-10 | MAPK3 | Predicted | 0.157 |
| RST-14 | PTK2 | Predicted | 0.000 | FC-10 | MAT1A | Predicted | 0.015 |
| RST-14 | RHOA | Predicted | 0.002 | FC-10 | MME | Predicted | 0.169 |
| RST-14 | SRC | Predicted | 0.133 | FC-10 | MMP12 | Predicted | 0.080 |
| RST-14 | SULT2A1 | Predicted | 0.008 | FC-10 | MMP9 | Predicted | 0.042 |
| RST-14 | TGM2 | Predicted | 0.002 | FC-10 | NME1 | Predicted | 0.063 |
| RST-14 | TOP2A | Predicted | 0.027 | FC-10 | NME2 | Predicted | 0.017 |
| RST-15 | ALDH2 | Predicted | 0.095 | FC-10 | PGK1 | Predicted | 0.015 |
| RST-15 | ALDOA | Predicted | 0.097 | FC-10 | PTGS2 | Predicted | 0.076 |
| RST-15 | CA1 | Predicted | 0.155 | FC-10 | PTK2 | Predicted | 0.015 |
| RST-15 | CA2 | Predicted | 0.137 | FC-10 | RHOA | Predicted | 0.017 |
| RST-15 | CCNA2 | Predicted | 0.093 | FC-10 | RND3 | Predicted | 0.019 |
| RST-15 | CES1 | Predicted | 0.010 | FC-10 | SERPINE1 | Predicted | 0.042 |
| RST-15 | ESR1 | Predicted | 0.043 | FC-10 | SGK1 | Predicted | 0.000 |
| RST-15 | GABRE | Predicted | 0.097 | FC-10 | SRC | Predicted | 0.247 |
| RST-15 | GNAI1 | Predicted | 0.001 | FC-10 | SULT2A1 | Predicted | 0.027 |
| RST-15 | GSTP1 | Predicted | 0.000 | FC-10 | TGM2 | Predicted | 0.017 |
| RST-15 | KRAS | Predicted | 0.031 | FC-10 | TOP2A | Predicted | 0.150 |
| RST-15 | MAPK1 | Predicted | 0.032 | FC-10 | TRDMT1 | Predicted | 0.006 |
| RST-15 | MMP12 | Predicted | 0.003 | FC-10 | VEGFA | Predicted | 0.042 |
| RST-15 | MMP14 | Predicted | 0.003 | FC-11 | ABCB1 | Predicted | 0.016 |
| RST-15 | MMP2 | Predicted | 0.003 | FC-11 | ACSL1 | Predicted | 0.016 |
| RST-15 | MMP3 | Predicted | 0.011 | FC-11 | ADH1C | Predicted | 0.014 |
| RST-15 | MMP7 | Predicted | 0.003 | FC-11 | AGXT | Predicted | 0.015 |
| RST-15 | MMP9 | Predicted | 0.003 | FC-11 | AKR1B10 | Predicted | 0.195 |
| RST-15 | MTAP | Predicted | 0.190 | FC-11 | AKT2 | Predicted | 0.298 |
| RST-15 | NME1 | Predicted | 0.001 | FC-11 | ASS1 | Predicted | 0.016 |
| RST-15 | NME2 | Predicted | 0.001 | FC-11 | AURKA | Predicted | 0.013 |
| RST-15 | NOS2A | Predicted | 0.189 | FC-11 | CA1 | Predicted | 0.096 |
| RST-15 | PARP1 | Predicted | 0.093 | FC-11 | CA2 | Predicted | 0.096 |
| RST-15 | PTGS2 | Predicted | 0.505 | FC-11 | CASP1 | Predicted | 0.041 |
| RST-15 | REG1A | Predicted | 0.010 | FC-11 | CCNA2 | Predicted | 1.000 |
| RST-15 | RHOA | Predicted | 0.001 | FC-11 | CCT3 | Predicted | 0.013 |
| RST-15 | TGM2 | Predicted | 0.001 | FC-11 | CDK4 | Predicted | 0.081 |
| RST-15 | TOP2A | Predicted | 0.092 | FC-11 | CES1 | Predicted | 0.014 |
| RST-16 | ALDH2 | Predicted | 0.096 | FC-11 | CTSB | Predicted | 0.199 |
| RST-16 | ALDOA | Predicted | 0.097 | FC-11 | ESR1 | Predicted | 0.203 |
| RST-16 | CA1 | Predicted | 0.146 | FC-11 | GABRE | Predicted | 0.005 |
| RST-16 | CA2 | Predicted | 0.120 | FC-11 | GLUD1 | Predicted | 0.038 |
| RST-16 | CCNA2 | Predicted | 0.093 | FC-11 | GNAI1 | Predicted | 0.014 |
| RST-16 | CES1 | Predicted | 0.010 | FC-11 | GNMT | Predicted | 0.013 |
| RST-16 | ESR1 | Predicted | 0.043 | FC-11 | HAO1 | Predicted | 0.000 |
| RST-16 | GABRE | Predicted | 0.105 | FC-11 | HSP90AA1 | Predicted | 0.013 |
| RST-16 | GNAI1 | Predicted | 0.001 | FC-11 | HSP90B1 | Predicted | 0.013 |
| RST-16 | GNMT | Predicted | 0.001 | FC-11 | HSPA1B | Predicted | 0.013 |
| RST-16 | GSTP1 | Predicted | 0.000 | FC-11 | HSPA8 | Predicted | 0.013 |
| RST-16 | KRAS | Predicted | 0.031 | FC-11 | KRAS | Predicted | 0.062 |
| RST-16 | MAPK1 | Predicted | 0.039 | FC-11 | MAPK1 | Predicted | 0.081 |
| RST-16 | MMP12 | Predicted | 0.003 | FC-11 | MAPK3 | Predicted | 0.081 |
| RST-16 | MMP14 | Predicted | 0.003 | FC-11 | MAT1A | Predicted | 0.013 |
| RST-16 | MMP2 | Predicted | 0.003 | FC-11 | MME | Predicted | 0.135 |
| RST-16 | MMP3 | Predicted | 0.011 | FC-11 | MMP12 | Predicted | 0.064 |
| RST-16 | MMP7 | Predicted | 0.003 | FC-11 | MMP9 | Predicted | 0.041 |
| RST-16 | MMP9 | Predicted | 0.003 | FC-11 | NME1 | Predicted | 0.054 |
| RST-16 | MTAP | Predicted | 0.189 | FC-11 | NME2 | Predicted | 0.014 |
| RST-16 | NME1 | Predicted | 0.001 | FC-11 | PGK1 | Predicted | 0.013 |
| RST-16 | NME2 | Predicted | 0.001 | FC-11 | PTK2 | Predicted | 0.013 |
| RST-16 | NOS2A | Predicted | 0.188 | FC-11 | RHOA | Predicted | 0.014 |
| RST-16 | PARP1 | Predicted | 0.093 | FC-11 | RND3 | Predicted | 0.019 |
| RST-16 | PTGS2 | Predicted | 0.503 | FC-11 | SERPINE1 | Predicted | 0.034 |
| RST-16 | REG1A | Predicted | 0.010 | FC-11 | SGK1 | Predicted | 0.000 |
| RST-16 | RHOA | Predicted | 0.001 | FC-11 | SRC | Predicted | 0.196 |
| RST-16 | TGM2 | Predicted | 0.001 | FC-11 | SULT2A1 | Predicted | 0.021 |
| RST-16 | TOP2A | Predicted | 0.093 | FC-11 | TGM2 | Predicted | 0.014 |
| RST-16 | TRDMT1 | Predicted | 0.001 | FC-11 | TOP2A | Predicted | 0.108 |
| RST-17 | ABCB1 | Predicted | 0.011 | FC-11 | TRDMT1 | Predicted | 0.013 |
| RST-17 | ACSL1 | Predicted | 0.041 | FC-11 | VEGFA | Predicted | 0.097 |
| RST-17 | ALDH2 | Predicted | 0.133 | FC-12 | ABCB1 | Predicted | 0.008 |
| RST-17 | ALDOA | Predicted | 0.131 | FC-12 | ACSL1 | Predicted | 0.008 |
| RST-17 | AR | Predicted | 0.247 | FC-12 | ADH1C | Predicted | 0.006 |
| RST-17 | ASS1 | Predicted | 0.011 | FC-12 | AGXT | Predicted | 0.011 |
| RST-17 | AURKA | Predicted | 0.004 | FC-12 | AKR1B10 | Predicted | 0.186 |
| RST-17 | CA1 | Predicted | 0.120 | FC-12 | AKT2 | Predicted | 0.260 |
| RST-17 | CA2 | Predicted | 0.189 | FC-12 | ASS1 | Predicted | 0.008 |
| RST-17 | CCT3 | Predicted | 0.004 | FC-12 | AURKA | Predicted | 0.009 |
| RST-17 | CTSB | Predicted | 0.138 | FC-12 | CCNA2 | Predicted | 0.982 |
| RST-17 | ESR1 | Predicted | 1.000 | FC-12 | CCT3 | Predicted | 0.028 |
| RST-17 | GABRE | Predicted | 0.090 | FC-12 | CDK4 | Predicted | 0.089 |
| RST-17 | GLUD1 | Predicted | 0.004 | FC-12 | CES1 | Predicted | 0.006 |
| RST-17 | GNAI1 | Predicted | 0.000 | FC-12 | CTSB | Predicted | 0.186 |
| RST-17 | GSTP1 | Predicted | 0.003 | FC-12 | ESR1 | Predicted | 0.195 |
| RST-17 | HSP90AA1 | Predicted | 0.004 | FC-12 | GLUD1 | Predicted | 0.034 |
| RST-17 | HSP90B1 | Predicted | 0.004 | FC-12 | GNAI1 | Predicted | 0.012 |
| RST-17 | HSPA1B | Predicted | 0.004 | FC-12 | GNMT | Predicted | 0.006 |
| RST-17 | HSPA8 | Predicted | 0.004 | FC-12 | GSTP1 | Predicted | 0.059 |
| RST-17 | KRAS | Predicted | 0.046 | FC-12 | HAO1 | Predicted | 0.000 |
| RST-17 | LYZ | Predicted | 0.011 | FC-12 | HSP90AA1 | Predicted | 0.009 |
| RST-17 | MAT1A | Predicted | 0.004 | FC-12 | HSP90B1 | Predicted | 0.009 |
| RST-17 | MMP12 | Predicted | 0.008 | FC-12 | HSPA1B | Predicted | 0.009 |
| RST-17 | MMP14 | Predicted | 0.008 | FC-12 | HSPA8 | Predicted | 0.009 |
| RST-17 | MMP2 | Predicted | 0.008 | FC-12 | KRAS | Predicted | 0.112 |
| RST-17 | MMP3 | Predicted | 0.025 | FC-12 | MAPK1 | Predicted | 0.187 |
| RST-17 | MMP7 | Predicted | 0.008 | FC-12 | MAPK3 | Predicted | 0.089 |
| RST-17 | MMP9 | Predicted | 0.008 | FC-12 | MAT1A | Predicted | 0.009 |
| RST-17 | MTAP | Predicted | 0.130 | FC-12 | MME | Predicted | 0.292 |
| RST-17 | NME1 | Predicted | 0.021 | FC-12 | MMP12 | Predicted | 0.076 |
| RST-17 | NME2 | Predicted | 0.000 | FC-12 | NME1 | Predicted | 0.049 |
| RST-17 | PGK1 | Predicted | 0.004 | FC-12 | NME2 | Predicted | 0.012 |
| RST-17 | PTGS2 | Predicted | 0.228 | FC-12 | PGK1 | Predicted | 0.009 |
| RST-17 | PTK2 | Predicted | 0.004 | FC-12 | PLAU | Predicted | 0.134 |
| RST-17 | RHOA | Predicted | 0.000 | FC-12 | PTK2 | Predicted | 0.009 |
| RST-17 | SULT2A1 | Predicted | 0.108 | FC-12 | RHOA | Predicted | 0.012 |
| RST-17 | TGM2 | Predicted | 0.000 | FC-12 | RND3 | Predicted | 0.016 |
| RST-18 | ACSL1 | Predicted | 0.018 | FC-12 | SERPINE1 | Predicted | 0.014 |
| RST-18 | ALDOA | Predicted | 0.085 | FC-12 | SGK1 | Predicted | 0.000 |
| RST-18 | AURKA | Predicted | 0.000 | FC-12 | SRC | Predicted | 0.192 |
| RST-18 | CA1 | Predicted | 0.174 | FC-12 | TGM2 | Predicted | 0.012 |
| RST-18 | CA2 | Predicted | 0.084 | FC-12 | TOP2A | Predicted | 0.049 |
| RST-18 | CCNA2 | Predicted | 0.528 | FC-12 | TRDMT1 | Predicted | 0.006 |
| RST-18 | CCT3 | Predicted | 0.000 | FC-12 | VEGFA | Predicted | 0.040 |
| RST-18 | CTSB | Predicted | 0.082 | FC-13 | ADH1C | Predicted | 0.022 |
| RST-18 | ESR1 | Predicted | 0.120 | FC-13 | AGXT | Predicted | 0.023 |
| RST-18 | GLUD1 | Predicted | 0.000 | FC-13 | AKT2 | Predicted | 0.362 |
| RST-18 | GSTP1 | Predicted | 0.000 | FC-13 | AURKA | Predicted | 0.018 |
| RST-18 | HSP90AA1 | Predicted | 0.000 | FC-13 | CA1 | Predicted | 0.147 |
| RST-18 | HSP90B1 | Predicted | 0.000 | FC-13 | CA2 | Predicted | 0.147 |
| RST-18 | HSPA1B | Predicted | 0.000 | FC-13 | CCT3 | Predicted | 0.018 |
| RST-18 | HSPA8 | Predicted | 0.000 | FC-13 | CDK4 | Predicted | 0.079 |
| RST-18 | KRAS | Predicted | 0.005 | FC-13 | CES1 | Predicted | 0.022 |
| RST-18 | LCN2 | Predicted | 0.082 | FC-13 | CTSB | Predicted | 0.312 |
| RST-18 | MAPK3 | Predicted | 0.041 | FC-13 | ESR1 | Predicted | 0.627 |
| RST-18 | MAT1A | Predicted | 0.000 | FC-13 | FGF1 | Predicted | 0.147 |
| RST-18 | MTAP | Predicted | 0.177 | FC-13 | FGF2 | Predicted | 0.147 |
| RST-18 | NME1 | Predicted | 0.000 | FC-13 | GABRE | Predicted | 0.008 |
| RST-18 | NOS2A | Predicted | 0.179 | FC-13 | GLUD1 | Predicted | 0.046 |
| RST-18 | PGK1 | Predicted | 0.000 | FC-13 | GNAI1 | Predicted | 0.018 |
| RST-18 | PTGS2 | Predicted | 0.244 | FC-13 | HAO1 | Predicted | 0.000 |
| RST-18 | PTK2 | Predicted | 0.000 | FC-13 | HSP90AA1 | Predicted | 0.018 |
| RST-18 | SRC | Predicted | 0.080 | FC-13 | HSP90B1 | Predicted | 0.018 |
| RST-18 | SULT2A1 | Predicted | 0.005 | FC-13 | HSPA1B | Predicted | 0.018 |
| RST-18 | TOP2A | Predicted | 0.026 | FC-13 | HSPA8 | Predicted | 0.018 |
| CT-01 | ACADSB | Predicted | 0.027 | FC-13 | KRAS | Predicted | 0.090 |
| CT-01 | ACAT2 | Predicted | 0.079 | FC-13 | MAPK1 | Predicted | 0.079 |
| CT-01 | ADH1C | Predicted | 0.328 | FC-13 | MAPK3 | Predicted | 0.159 |
| CT-01 | ALDH1A1 | Predicted | 0.025 | FC-13 | MAT1A | Predicted | 0.018 |
| CT-01 | ALDOA | Predicted | 0.052 | FC-13 | MME | Predicted | 0.207 |
| CT-01 | ALDOB | Predicted | 0.136 | FC-13 | MMP12 | Predicted | 0.097 |
| CT-01 | ARG1 | Predicted | 0.062 | FC-13 | NME1 | Predicted | 0.072 |
| CT-01 | ASPH | Predicted | 0.047 | FC-13 | NME2 | Predicted | 0.018 |
| CT-01 | ASS1 | Predicted | 0.022 | FC-13 | PGK1 | Predicted | 0.018 |
| CT-01 | AXIN1 | Predicted | 0.025 | FC-13 | PTGS2 | Predicted | 0.071 |
| CT-01 | B2M | Predicted | 0.000 | FC-13 | PTK2 | Predicted | 0.018 |
| CT-01 | BHMT | Predicted | 0.080 | FC-13 | RHOA | Predicted | 0.018 |
| CT-01 | C1R | Predicted | 0.019 | FC-13 | RND3 | Predicted | 0.019 |
| CT-01 | CBS | Predicted | 0.018 | FC-13 | SERPINE1 | Predicted | 0.052 |
| CT-01 | CDC25B | Predicted | 0.026 | FC-13 | SGK1 | Predicted | 0.000 |
| CT-01 | CTH | Predicted | 0.018 | FC-13 | SRC | Predicted | 0.304 |
| CT-01 | DCN | Predicted | 0.013 | FC-13 | SULT2A1 | Predicted | 0.032 |
| CT-01 | EGF | Predicted | 0.074 | FC-13 | TGM2 | Predicted | 0.018 |
| CT-01 | FTCD | Predicted | 0.016 | FC-13 | TOP2A | Predicted | 0.184 |
| CT-01 | GLUD1 | Predicted | 0.016 | FC-13 | VEGFA | Predicted | 0.082 |
| CT-01 | GLUL | Predicted | 0.016 | FC-14 | AKT2 | Predicted | 0.171 |
| CT-01 | GNMT | Predicted | 0.013 | FC-14 | ALDH1L1 | Predicted | 0.003 |
| CT-01 | GSTP1 | Predicted | 0.079 | FC-14 | AURKA | Predicted | 0.151 |
| CT-01 | HSD17B6 | Predicted | 0.010 | FC-14 | CYP2C8 | Predicted | 0.008 |
| CT-01 | LYZ | Predicted | 0.022 | FC-14 | FTCD | Predicted | 0.003 |
| CT-01 | MAPK1 | Predicted | 0.010 | FC-14 | KRAS | Predicted | 0.047 |
| CT-01 | MGMT | Predicted | 0.018 | FC-14 | MTHFD1 | Predicted | 0.003 |
| CT-01 | MIF | Predicted | 0.013 | FC-14 | MTR | Predicted | 0.003 |
| CT-01 | MTR | Predicted | 0.051 | FC-14 | NOS2A | Predicted | 0.152 |
| CT-01 | NME1 | Predicted | 0.007 | FC-14 | PTGS2 | Predicted | 0.035 |
| CT-01 | NOS2A | Predicted | 0.154 | FC-14 | RAF1 | Predicted | 0.016 |
| CT-01 | OAT | Predicted | 0.062 | FC-14 | RARA | Predicted | 0.021 |
| CT-01 | PCK1 | Predicted | 0.061 | FC-14 | SRC | Predicted | 0.018 |
| CT-01 | PGK1 | Predicted | 0.058 | FC-15 | DDR1 | Predicted | 0.077 |
| CT-01 | PKM2 | Predicted | 0.046 | FC-15 | EGFR | Predicted | 0.471 |
| CT-01 | PLAU | Predicted | 0.019 | FC-15 | ICAM1 | Validated | 1.000 |
| CT-01 | PLG | Predicted | 0.328 | FC-15 | PDGFRA | Predicted | 0.077 |
| CT-01 | PPIB | Predicted | 0.050 | FC-16 | ADH1B | Predicted | 0.010 |
| CT-01 | REG1A | Predicted | 0.008 | FC-16 | ADH1C | Predicted | 0.001 |
| CT-01 | SERPINC1 | Predicted | 0.000 | FC-16 | ADH4 | Predicted | 0.001 |
| CT-01 | SLC7A2 | Predicted | 0.139 | FC-16 | AKR1B10 | Predicted | 0.000 |
| CT-01 | SRC | Predicted | 0.013 | FC-16 | AKR1C2 | Predicted | 0.000 |
| CT-01 | TAOK2 | Predicted | 0.008 | FC-16 | ALDH1A1 | Predicted | 0.001 |
| CT-01 | TF | Predicted | 0.019 | FC-16 | ALDH1L1 | Predicted | 0.004 |
| CT-01 | THBS1 | Predicted | 0.019 | FC-16 | ALDH2 | Predicted | 0.001 |
| CT-01 | TST | Predicted | 0.025 | FC-16 | DCXR | Predicted | 0.000 |
| CT-01 | VEGFA | Predicted | 0.049 | FC-16 | DDR1 | Predicted | 0.049 |
| CT-02 | ACADSB | Predicted | 0.041 | FC-16 | EGFR | Predicted | 0.246 |
| CT-02 | ACAT2 | Predicted | 0.160 | FC-16 | ESR1 | Predicted | 0.248 |
| CT-02 | ALDH1A1 | Predicted | 0.039 | FC-16 | FTCD | Predicted | 0.004 |
| CT-02 | ALDOA | Predicted | 0.095 | FC-16 | GAPDH | Predicted | 0.001 |
| CT-02 | ALDOB | Predicted | 0.262 | FC-16 | GLUD1 | Predicted | 0.001 |
| CT-02 | ARG1 | Predicted | 0.107 | FC-16 | HSD17B10 | Predicted | 0.001 |
| CT-02 | ASPH | Predicted | 0.083 | FC-16 | HSD17B4 | Predicted | 0.001 |
| CT-02 | ASS1 | Predicted | 0.032 | FC-16 | MME | Predicted | 0.255 |
| CT-02 | AXIN1 | Predicted | 0.039 | FC-16 | MTHFD1 | Predicted | 0.013 |
| CT-02 | BHMT | Predicted | 0.129 | FC-16 | MTR | Predicted | 0.004 |
| CT-02 | C1R | Predicted | 0.022 | FC-16 | PDGFRA | Predicted | 0.049 |
| CT-02 | CBS | Predicted | 0.095 | FC-16 | QDPR | Predicted | 0.001 |
| CT-02 | CDC25B | Predicted | 0.008 | FC-16 | RAF1 | Predicted | 0.033 |
| CT-02 | CTH | Predicted | 0.017 | FC-17 | ACSL1 | Predicted | 0.024 |
| CT-02 | DCN | Predicted | 0.011 | FC-17 | ALDH2 | Predicted | 0.089 |
| CT-02 | DNMT1 | Predicted | 0.057 | FC-17 | ALDOA | Predicted | 0.087 |
| CT-02 | EGF | Predicted | 0.129 | FC-17 | AR | Predicted | 0.129 |
| CT-02 | FTCD | Predicted | 0.018 | FC-17 | CA1 | Predicted | 0.165 |
| CT-02 | GLUD1 | Predicted | 0.018 | FC-17 | CA2 | Predicted | 0.052 |
| CT-02 | GLUL | Predicted | 0.018 | FC-17 | CCNA2 | Predicted | 0.265 |
| CT-02 | GNMT | Predicted | 0.008 | FC-17 | ESR1 | Predicted | 0.610 |
| CT-02 | GSTP1 | Predicted | 0.160 | FC-17 | FGF1 | Predicted | 0.014 |
| CT-02 | HSD17B6 | Predicted | 0.009 | FC-17 | FGF2 | Predicted | 0.014 |
| CT-02 | LYZ | Predicted | 0.106 | FC-17 | GABRE | Predicted | 0.022 |
| CT-02 | MGMT | Predicted | 0.017 | FC-17 | GSTP1 | Predicted | 0.000 |
| CT-02 | MIF | Predicted | 0.008 | FC-17 | HGF | Predicted | 0.014 |
| CT-02 | MTR | Predicted | 0.079 | FC-17 | KRAS | Predicted | 0.007 |
| CT-02 | NME1 | Predicted | 0.000 | FC-17 | LCN2 | Predicted | 0.088 |
| CT-02 | NOS2A | Predicted | 0.290 | FC-17 | MAPK1 | Predicted | 0.034 |
| CT-02 | OAT | Predicted | 0.107 | FC-17 | MTAP | Predicted | 0.178 |
| CT-02 | PCK1 | Predicted | 0.119 | FC-17 | NOS2A | Predicted | 0.179 |
| CT-02 | PGK1 | Predicted | 0.122 | FC-17 | PTGS2 | Predicted | 1.000 |
| CT-02 | PKM2 | Predicted | 0.091 | FC-17 | SRC | Predicted | 0.024 |
| CT-02 | PLAU | Predicted | 0.022 | FC-17 | TNFSF11 | Predicted | 0.025 |
| CT-02 | PLG | Predicted | 0.290 | FC-17 | TOP2A | Predicted | 0.055 |
| CT-02 | PPIB | Predicted | 0.088 | FC-18 | ALDH2 | Predicted | 0.131 |
| CT-02 | REG1A | Predicted | 0.005 | FC-18 | APC | Validated | 1.000 |
| CT-02 | SLC7A2 | Predicted | 0.107 | FC-18 | AR | Validated | 1.000 |
| CT-02 | SRC | Predicted | 0.008 | FC-18 | BAD | Validated | 1.000 |
| CT-02 | TAOK2 | Predicted | 0.005 | FC-18 | CA1 | Predicted | 0.125 |
| CT-02 | TF | Predicted | 0.022 | FC-18 | CA2 | Predicted | 0.058 |
| CT-02 | THBS1 | Predicted | 0.022 | FC-18 | CASP3 | Validated | 1.000 |
| CT-02 | VEGFA | Predicted | 0.078 | FC-18 | CCNA2 | Validated | 1.000 |
| CT-03 | ALDH1A1 | Predicted | 0.056 | FC-18 | CDK4 | Validated | 1.000 |
| CT-03 | ALDOA | Predicted | 0.084 | FC-18 | CDKN1A | Validated | 1.000 |
| CT-03 | ALDOB | Predicted | 0.204 | FC-18 | CDKN2A | Validated | 1.000 |
| CT-03 | ARG1 | Predicted | 0.158 | FC-18 | ESR1 | Validated | 1.000 |
| CT-03 | ASS1 | Predicted | 0.039 | FC-18 | FOS | Validated | 1.000 |
| CT-03 | AXIN1 | Predicted | 0.056 | FC-18 | ICAM1 | Validated | 1.000 |
| CT-03 | B2M | Predicted | 0.000 | FC-18 | IL2 | Validated | 1.000 |
| CT-03 | BHMT | Predicted | 0.178 | FC-18 | KRAS | Predicted | 0.007 |
| CT-03 | CA1 | Predicted | 0.030 | FC-18 | MCL1 | Validated | 1.000 |
| CT-03 | CA2 | Predicted | 0.030 | FC-18 | MMP9 | Validated | 1.000 |
| CT-03 | CA3 | Predicted | 0.030 | FC-18 | MTAP | Predicted | 0.264 |
| CT-03 | CES1 | Predicted | 0.440 | FC-18 | NFKBIA | Validated | 1.000 |
| CT-03 | FGF2 | Predicted | 0.568 | FC-18 | NOS2A | Validated | 1.000 |
| CT-03 | FTCD | Predicted | 0.016 | FC-18 | PLAU | Validated | 1.000 |
| CT-03 | GLUD1 | Predicted | 0.016 | FC-18 | PTGS2 | Validated | 1.000 |
| CT-03 | GLUL | Predicted | 0.016 | FC-18 | RB1 | Validated | 1.000 |
| CT-03 | GSTP1 | Predicted | 0.040 | FC-18 | SERPINE1 | Validated | 1.000 |
| CT-03 | HAO1 | Predicted | 0.195 | FC-18 | SRC | Predicted | 0.124 |
| CT-03 | MAPK1 | Predicted | 0.026 | FC-18 | TOP2A | Predicted | 0.079 |
| CT-03 | MIF | Predicted | 0.161 | FC-18 | TP53 | Validated | 1.000 |
| CT-03 | MTR | Predicted | 0.178 | FC-18 | VEGFA | Validated | 1.000 |
| CT-03 | NOS2A | Predicted | 0.160 | FC-19 | DDR1 | Predicted | 0.063 |
| CT-03 | OAT | Predicted | 0.045 | FC-19 | PDGFRA | Predicted | 0.063 |
| CT-03 | PGK1 | Predicted | 0.071 | FC-19 | PTGS2 | Predicted | 0.118 |
| CT-03 | PLG | Predicted | 0.437 | FC-19 | SRC | Predicted | 0.062 |
| CT-03 | PPIB | Predicted | 0.056 | FC-20 | EGFR | Predicted | 0.920 |
| CT-03 | PRDX6 | Predicted | 0.040 | FC-21 | AURKA | Predicted | 0.604 |
| CT-03 | PTGS2 | Predicted | 0.569 | FC-21 | DDR1 | Predicted | 0.080 |
| CT-03 | QDPR | Predicted | 0.040 | FC-21 | EGFR | Predicted | 0.580 |
| CT-03 | REG1A | Predicted | 0.028 | FC-21 | PDGFRA | Predicted | 0.080 |
| CT-03 | SERPINC1 | Predicted | 0.000 | FC-21 | PTGS2 | Predicted | 0.143 |
| CT-03 | SLC7A2 | Predicted | 0.108 | FC-21 | SRC | Predicted | 0.079 |
| CT-03 | TAOK2 | Predicted | 0.028 | RS-01 | ACSL1 | Predicted | 0.020 |
| CT-03 | TST | Predicted | 0.030 | RS-01 | AGXT | Predicted | 0.003 |
| CT-04 | ACADSB | Predicted | 0.040 | RS-01 | AKR1B10 | Predicted | 0.107 |
| CT-04 | ACAT2 | Predicted | 0.115 | RS-01 | AKT2 | Predicted | 0.059 |
| CT-04 | ALDH1A1 | Predicted | 0.031 | RS-01 | AURKA | Predicted | 0.000 |
| CT-04 | ALDOA | Predicted | 0.087 | RS-01 | CA1 | Predicted | 0.077 |
| CT-04 | ALDOB | Predicted | 0.242 | RS-01 | CA2 | Predicted | 0.077 |
| CT-04 | ARG1 | Predicted | 0.111 | RS-01 | CCNA2 | Predicted | 1.000 |
| CT-04 | ASPH | Predicted | 0.070 | RS-01 | CCT3 | Predicted | 0.000 |
| CT-04 | ASS1 | Predicted | 0.034 | RS-01 | CTSB | Predicted | 0.108 |
| CT-04 | AXIN1 | Predicted | 0.031 | RS-01 | ESR1 | Predicted | 0.104 |
| CT-04 | CBS | Predicted | 0.102 | RS-01 | GABRE | Predicted | 0.011 |
| CT-04 | CDC25B | Predicted | 0.013 | RS-01 | GLUD1 | Predicted | 0.011 |
| CT-04 | CTH | Predicted | 0.021 | RS-01 | GNAI1 | Predicted | 0.002 |
| CT-04 | EGF | Predicted | 0.134 | RS-01 | GNMT | Predicted | 0.005 |
| CT-04 | FTCD | Predicted | 0.018 | RS-01 | GSTP1 | Predicted | 0.006 |
| CT-04 | GLUD1 | Predicted | 0.018 | RS-01 | HSP90AA1 | Predicted | 0.000 |
| CT-04 | GLUL | Predicted | 0.018 | RS-01 | HSP90B1 | Predicted | 0.000 |
| CT-04 | GSTP1 | Predicted | 0.115 | RS-01 | HSPA1B | Predicted | 0.000 |
| CT-04 | HSD17B6 | Predicted | 0.000 | RS-01 | HSPA8 | Predicted | 0.000 |
| CT-04 | LYZ | Predicted | 0.105 | RS-01 | KRAS | Predicted | 0.042 |
| CT-04 | MGMT | Predicted | 0.021 | RS-01 | LYZ | Predicted | 0.105 |
| CT-04 | NME1 | Predicted | 0.005 | RS-01 | MAPK3 | Predicted | 0.038 |
| CT-04 | NOS2A | Predicted | 0.295 | RS-01 | MAT1A | Predicted | 0.000 |
| CT-04 | OAT | Predicted | 0.111 | RS-01 | MTAP | Predicted | 0.216 |
| CT-04 | PCK1 | Predicted | 0.088 | RS-01 | NME1 | Predicted | 0.019 |
| CT-04 | PGK1 | Predicted | 0.090 | RS-01 | NME2 | Predicted | 0.002 |
| CT-04 | PKM2 | Predicted | 0.073 | RS-01 | NOS2A | Predicted | 0.111 |
| CT-04 | PPIB | Predicted | 0.068 | RS-01 | PGK1 | Predicted | 0.000 |
| CT-04 | REG1A | Predicted | 0.010 | RS-01 | PTGS2 | Predicted | 0.208 |
| CT-04 | SLC7A2 | Predicted | 0.111 | RS-01 | PTK2 | Predicted | 0.000 |
| CT-04 | TAOK2 | Predicted | 0.010 | RS-01 | RHOA | Predicted | 0.002 |
| CT-04 | VEGFA | Predicted | 0.003 | RS-01 | RND3 | Predicted | 0.003 |
| CT-05 | ACADSB | Predicted | 0.029 | RS-01 | SERPINE1 | Predicted | 0.028 |
| CT-05 | ACAT2 | Predicted | 0.076 | RS-01 | SRC | Predicted | 0.105 |
| CT-05 | ADH1C | Predicted | 0.501 | RS-01 | SULT2A1 | Predicted | 0.004 |
| CT-05 | ALDH1A1 | Predicted | 0.036 | RS-01 | TGM2 | Predicted | 0.002 |
| CT-05 | ALDOA | Predicted | 0.077 | RS-01 | TOP2A | Predicted | 0.021 |
| CT-05 | ALDOB | Predicted | 0.199 | RS-01 | TRDMT1 | Predicted | 0.005 |
| CT-05 | ARG1 | Predicted | 0.086 | RS-02 | ABCB1 | Predicted | 0.013 |
| CT-05 | ASPH | Predicted | 0.049 | RS-02 | ACSL1 | Predicted | 0.013 |
| CT-05 | ASS1 | Predicted | 0.014 | RS-02 | ADH1B | Predicted | 0.019 |
| CT-05 | AXIN1 | Predicted | 0.036 | RS-02 | ADH1C | Predicted | 0.021 |
| CT-05 | B2M | Predicted | 0.000 | RS-02 | ADH4 | Predicted | 0.011 |
| CT-05 | BHMT | Predicted | 0.104 | RS-02 | AGXT | Predicted | 0.009 |
| CT-05 | C1R | Predicted | 0.051 | RS-02 | AKR1B10 | Predicted | 0.003 |
| CT-05 | CBS | Predicted | 0.012 | RS-02 | AKR1C2 | Predicted | 0.011 |
| CT-05 | CDC25B | Predicted | 0.036 | RS-02 | AKT2 | Predicted | 0.055 |
| CT-05 | CES1 | Predicted | 0.015 | RS-02 | ALDH1A1 | Predicted | 0.011 |
| CT-05 | CTH | Predicted | 0.012 | RS-02 | ALDH1B1 | Predicted | 0.003 |
| CT-05 | FGA | Predicted | 0.015 | RS-02 | ALDH2 | Predicted | 0.011 |
| CT-05 | FTCD | Predicted | 0.017 | RS-02 | ALDH3A2 | Predicted | 0.003 |
| CT-05 | GLUD1 | Predicted | 0.017 | RS-02 | ALDH4A1 | Predicted | 0.003 |
| CT-05 | GLUL | Predicted | 0.017 | RS-02 | ASS1 | Predicted | 0.013 |
| CT-05 | GNMT | Predicted | 0.009 | RS-02 | CCNA2 | Predicted | 0.280 |
| CT-05 | GSTP1 | Predicted | 0.108 | RS-02 | CCT3 | Predicted | 0.011 |
| CT-05 | HAO1 | Predicted | 0.470 | RS-02 | CDK4 | Predicted | 0.047 |
| CT-05 | HSD17B6 | Predicted | 0.013 | RS-02 | CES1 | Predicted | 0.004 |
| CT-05 | IGF2R | Predicted | 0.015 | RS-02 | DCXR | Predicted | 0.003 |
| CT-05 | LCN2 | Predicted | 0.015 | RS-02 | ESR1 | Predicted | 0.277 |
| CT-05 | LYZ | Predicted | 0.051 | RS-02 | GAPDH | Predicted | 0.025 |
| CT-05 | MAPK1 | Predicted | 0.022 | RS-02 | GLUD1 | Predicted | 0.011 |
| CT-05 | MGMT | Predicted | 0.012 | RS-02 | GSTP1 | Predicted | 0.116 |
| CT-05 | MIF | Predicted | 0.009 | RS-02 | HAO1 | Predicted | 0.002 |
| CT-05 | MMP9 | Predicted | 0.104 | RS-02 | HSD17B10 | Predicted | 0.011 |
| CT-05 | MTR | Predicted | 0.072 | RS-02 | HSD17B4 | Predicted | 0.011 |
| CT-05 | OAT | Predicted | 0.086 | RS-02 | KRAS | Predicted | 0.055 |
| CT-05 | PCK1 | Predicted | 0.088 | RS-02 | MAPK1 | Predicted | 0.083 |
| CT-05 | PGK1 | Predicted | 0.084 | RS-02 | MAPK3 | Predicted | 0.047 |
| CT-05 | PKM2 | Predicted | 0.066 | RS-02 | MME | Predicted | 0.209 |
| CT-05 | PLAU | Predicted | 0.014 | RS-02 | MMP12 | Predicted | 0.042 |
| CT-05 | PLG | Predicted | 0.500 | RS-02 | MTHFD1 | Predicted | 0.011 |
| CT-05 | PPIB | Predicted | 0.036 | RS-02 | NOS2A | Predicted | 0.135 |
| CT-05 | PRDX6 | Predicted | 0.009 | RS-02 | QDPR | Predicted | 0.011 |
| CT-05 | QDPR | Predicted | 0.009 | RS-02 | RARA | Predicted | 0.022 |
| CT-05 | REG1A | Predicted | 0.026 | RS-02 | SERPINE1 | Predicted | 0.010 |
| CT-05 | SERPINC1 | Predicted | 0.037 | RS-02 | SGK1 | Predicted | 0.002 |
| CT-05 | SLC7A2 | Predicted | 0.195 | RS-02 | SRC | Predicted | 0.184 |
| CT-05 | SRC | Predicted | 0.009 | RS-02 | TOP2A | Predicted | 0.169 |
| CT-05 | TAOK2 | Predicted | 0.026 | RS-02 | TUBB | Predicted | 0.007 |
| CT-05 | TF | Predicted | 0.014 | RS-02 | VEGFA | Predicted | 0.021 |
| CT-05 | THBS1 | Predicted | 0.014 | RS-03 | ADH1B | Predicted | 0.027 |
| CT-05 | TST | Predicted | 0.036 | RS-03 | ADH1C | Predicted | 0.015 |
| CT-06 | ACADSB | Predicted | 0.018 | RS-03 | ADH4 | Predicted | 0.015 |
| CT-06 | ACAT2 | Predicted | 0.039 | RS-03 | AKR1B10 | Predicted | 0.000 |
| CT-06 | ADH1C | Predicted | 0.329 | RS-03 | AKR1C2 | Predicted | 0.013 |
| CT-06 | ALDH1A1 | Predicted | 0.024 | RS-03 | ALDH1A1 | Predicted | 0.015 |
| CT-06 | ALDOA | Predicted | 0.050 | RS-03 | ALDH1B1 | Predicted | 0.001 |
| CT-06 | ALDOB | Predicted | 0.131 | RS-03 | ALDH1L1 | Predicted | 0.000 |
| CT-06 | ARG1 | Predicted | 0.046 | RS-03 | ALDH2 | Predicted | 0.015 |
| CT-06 | ASPH | Predicted | 0.036 | RS-03 | ALDH3A2 | Predicted | 0.001 |
| CT-06 | ASS1 | Predicted | 0.054 | RS-03 | ALDH4A1 | Predicted | 0.001 |
| CT-06 | AXIN1 | Predicted | 0.024 | RS-03 | CCNA2 | Predicted | 0.243 |
| CT-06 | B2M | Predicted | 0.000 | RS-03 | CDK4 | Predicted | 0.097 |
| CT-06 | BHMT | Predicted | 0.070 | RS-03 | DCXR | Predicted | 0.000 |
| CT-06 | C1R | Predicted | 0.041 | RS-03 | EGFR | Predicted | 0.252 |
| CT-06 | CDC25B | Predicted | 0.023 | RS-03 | ESR1 | Predicted | 0.240 |
| CT-06 | CES1 | Predicted | 0.098 | RS-03 | FTCD | Predicted | 0.000 |
| CT-06 | FGA | Predicted | 0.014 | RS-03 | GAPDH | Predicted | 0.039 |
| CT-06 | FTCD | Predicted | 0.011 | RS-03 | GLUD1 | Predicted | 0.015 |
| CT-06 | GLUD1 | Predicted | 0.011 | RS-03 | GSTP1 | Predicted | 0.210 |
| CT-06 | GLUL | Predicted | 0.011 | RS-03 | HSD17B10 | Predicted | 0.015 |
| CT-06 | GNMT | Predicted | 0.006 | RS-03 | HSD17B4 | Predicted | 0.015 |
| CT-06 | GSTP1 | Predicted | 0.061 | RS-03 | MAPK1 | Predicted | 0.097 |
| CT-06 | HAO1 | Predicted | 0.312 | RS-03 | MAPK3 | Predicted | 0.097 |
| CT-06 | HSD17B6 | Predicted | 0.008 | RS-03 | MME | Predicted | 0.511 |
| CT-06 | IGF2R | Predicted | 0.014 | RS-03 | MMP12 | Predicted | 0.052 |
| CT-06 | LCN2 | Predicted | 0.014 | RS-03 | MTHFD1 | Predicted | 0.025 |
| CT-06 | LYZ | Predicted | 0.042 | RS-03 | MTR | Predicted | 0.000 |
| CT-06 | MAPK1 | Predicted | 0.015 | RS-03 | QDPR | Predicted | 0.015 |

| CT-06 | MIF | Predicted | 0.006 | RS-03 | TUBB | Predicted | 0.035 |
| --- | --- | --- | --- | --- | --- | --- | --- |
| CT-06 | MMP9 | Predicted | 0.067 | RS-03 | VEGFA | Predicted | 0.032 |
| CT-06 | MTR | Predicted | 0.049 | RS-04 | ABCB1 | Predicted | 0.003 |
| CT-06 | NOS2A | Predicted | 0.076 | RS-04 | ACSL1 | Predicted | 0.003 |
| CT-06 | OAT | Predicted | 0.046 | RS-04 | AKT2 | Predicted | 0.124 |
| CT-06 | PCK1 | Predicted | 0.058 | RS-04 | ASS1 | Predicted | 0.003 |
| CT-06 | PGK1 | Predicted | 0.055 | RS-04 | AURKA | Predicted | 0.001 |
| CT-06 | PKM2 | Predicted | 0.043 | RS-04 | CA1 | Predicted | 0.011 |
| CT-06 | PLAU | Predicted | 0.012 | RS-04 | CA2 | Predicted | 0.011 |
| CT-06 | PLG | Predicted | 0.330 | RS-04 | CCNA2 | Validated | 1.000 |
| CT-06 | PPIB | Predicted | 0.024 | RS-04 | CCT3 | Predicted | 0.009 |
| CT-06 | PRDX6 | Predicted | 0.006 | RS-04 | CDK4 | Predicted | 0.035 |
| CT-06 | QDPR | Predicted | 0.006 | RS-04 | EGFR | Predicted | 0.085 |
| CT-06 | REG1A | Predicted | 0.017 | RS-04 | GAPDH | Predicted | 0.010 |
| CT-06 | SERPINC1 | Predicted | 0.029 | RS-04 | GLUD1 | Predicted | 0.014 |
| CT-06 | SLC7A2 | Predicted | 0.108 | RS-04 | GNMT | Predicted | 0.002 |
| CT-06 | SRC | Predicted | 0.006 | RS-04 | HSP90AA1 | Predicted | 0.001 |
| CT-06 | TAOK2 | Predicted | 0.017 | RS-04 | HSP90B1 | Predicted | 0.001 |
| CT-06 | TF | Predicted | 0.012 | RS-04 | HSPA1B | Predicted | 0.001 |
| CT-06 | THBS1 | Predicted | 0.012 | RS-04 | HSPA8 | Predicted | 0.001 |
| CT-06 | TST | Predicted | 0.023 | RS-04 | KRAS | Predicted | 0.050 |
| CT-07 | ALDOA | Predicted | 0.076 | RS-04 | MAPK1 | Predicted | 0.096 |
| CT-07 | AR | Predicted | 0.079 | RS-04 | MAPK3 | Predicted | 0.035 |
| CT-07 | ARG1 | Predicted | 0.023 | RS-04 | MAT1A | Predicted | 0.001 |
| CT-07 | B2M | Predicted | 0.014 | RS-04 | MME | Predicted | 0.132 |

| CT-07 | CA1 | Predicted | 0.210 | RS-04 | NME1 | Predicted | 0.001 |
| --- | --- | --- | --- | --- | --- | --- | --- |
| CT-07 | CA2 | Predicted | 0.100 | RS-04 | NOS2A | Predicted | 0.086 |
| CT-07 | CA3 | Predicted | 0.026 | RS-04 | PGK1 | Predicted | 0.001 |
| CT-07 | DNMT1 | Predicted | 0.033 | RS-04 | PTK2 | Predicted | 0.001 |
| CT-07 | ESR1 | Predicted | 0.251 | RS-04 | RND3 | Predicted | 0.006 |
| CT-07 | FGF1 | Predicted | 0.007 | RS-04 | SERPINE1 | Predicted | 0.024 |
| CT-07 | FGF2 | Predicted | 0.007 | RS-04 | SRC | Predicted | 0.116 |
| CT-07 | HAO1 | Predicted | 0.077 | RS-04 | TOP2A | Predicted | 0.115 |
| CT-07 | HGF | Predicted | 0.007 | RS-04 | TRDMT1 | Predicted | 0.002 |
| CT-07 | KRAS | Predicted | 0.000 | RS-04 | VEGFA | Predicted | 0.016 |
| CT-07 | LCN2 | Predicted | 0.083 | RS-04 | VEGFA | Validated | 1.000 |
| CT-07 | MAPK1 | Predicted | 0.020 | RS-05 | ABCB1 | Predicted | 0.005 |
| CT-07 | NOS2A | Predicted | 0.191 | RS-05 | ACSL1 | Predicted | 0.005 |
| CT-07 | PC | Predicted | 0.026 | RS-05 | AGXT | Predicted | 0.004 |
| CT-07 | PIN1 | Predicted | 0.035 | RS-05 | AKR1B10 | Predicted | 0.070 |
| CT-07 | PTGS2 | Predicted | 1.000 | RS-05 | AKT2 | Predicted | 0.039 |
| CT-07 | SRC | Predicted | 0.035 | RS-05 | ALDH1A1 | Predicted | 0.019 |
| CT-07 | TNFSF11 | Predicted | 0.020 | RS-05 | ASS1 | Predicted | 0.005 |
| CT-07 | TOP2A | Predicted | 0.079 | RS-05 | AURKA | Predicted | 0.003 |
| CT-08 | AGXT | Predicted | 0.014 | RS-05 | CA1 | Predicted | 0.051 |
| CT-08 | ALAS1 | Predicted | 0.014 | RS-05 | CA2 | Predicted | 0.051 |
| CT-08 | ALDH1A1 | Predicted | 0.028 | RS-05 | CCNA2 | Predicted | 1.000 |
| CT-08 | ARG1 | Predicted | 0.105 | RS-05 | CCT3 | Predicted | 0.003 |
| CT-08 | ASS1 | Predicted | 0.039 | RS-05 | CTSB | Predicted | 0.069 |
| CT-08 | AXIN1 | Predicted | 0.028 | RS-05 | ESR1 | Predicted | 0.068 |

| CT-08 | B2M | Predicted | 0.000 | RS-05 | GABRE | Predicted | 0.001 |
| --- | --- | --- | --- | --- | --- | --- | --- |
| CT-08 | BHMT | Predicted | 0.088 | RS-05 | GLUD1 | Predicted | 0.010 |
| CT-08 | CA1 | Predicted | 0.123 | RS-05 | GNAI1 | Predicted | 0.004 |
| CT-08 | CA2 | Predicted | 0.078 | RS-05 | GNMT | Predicted | 0.004 |
| CT-08 | CA3 | Predicted | 0.034 | RS-05 | HAO1 | Predicted | 0.000 |
| CT-08 | CBS | Predicted | 0.014 | RS-05 | HSP90AA1 | Predicted | 0.003 |
| CT-08 | CES1 | Predicted | 0.251 | RS-05 | HSP90B1 | Predicted | 0.003 |
| CT-08 | CTH | Predicted | 0.014 | RS-05 | HSPA1B | Predicted | 0.003 |
| CT-08 | FGF1 | Predicted | 0.075 | RS-05 | HSPA8 | Predicted | 0.003 |
| CT-08 | FGF2 | Predicted | 0.342 | RS-05 | KRAS | Predicted | 0.018 |
| CT-08 | FTCD | Predicted | 0.014 | RS-05 | LYZ | Predicted | 0.069 |
| CT-08 | GAPDH | Predicted | 0.232 | RS-05 | MAPK3 | Predicted | 0.016 |
| CT-08 | GSTP1 | Predicted | 0.032 | RS-05 | MAT1A | Predicted | 0.003 |
| CT-08 | HAO1 | Predicted | 0.227 | RS-05 | MME | Predicted | 0.067 |
| CT-08 | HGF | Predicted | 0.075 | RS-05 | NME1 | Predicted | 0.016 |
| CT-08 | IGF2R | Predicted | 0.061 | RS-05 | NME2 | Predicted | 0.004 |
| CT-08 | LCN2 | Predicted | 0.235 | RS-05 | NOS2A | Predicted | 0.068 |
| CT-08 | MIF | Predicted | 0.158 | RS-05 | PGK1 | Predicted | 0.003 |
| CT-08 | MMP2 | Predicted | 0.051 | RS-05 | PTGS2 | Predicted | 0.075 |
| CT-08 | MTR | Predicted | 0.088 | RS-05 | PTK2 | Predicted | 0.003 |
| CT-08 | NOS2A | Predicted | 0.189 | RS-05 | RARA | Predicted | 0.013 |
| CT-08 | OAT | Predicted | 0.014 | RS-05 | RBP1 | Predicted | 0.006 |
| CT-08 | PLG | Predicted | 0.240 | RS-05 | RHOA | Predicted | 0.004 |
| CT-08 | PPIB | Predicted | 0.028 | RS-05 | RND3 | Predicted | 0.004 |
| CT-08 | PRDX6 | Predicted | 0.032 | RS-05 | SERPINE1 | Predicted | 0.016 |

| CT-08 | PTGS2 | Predicted | 0.491 | RS-05 | SGK1 | Predicted | 0.000 |
| --- | --- | --- | --- | --- | --- | --- | --- |
| CT-08 | PYGB | Predicted | 0.014 | RS-05 | SRC | Predicted | 0.060 |
| CT-08 | QDPR | Predicted | 0.032 | RS-05 | TGM2 | Predicted | 0.004 |
| CT-08 | SERPINC1 | Predicted | 0.000 | RS-05 | TOP2A | Predicted | 0.043 |
| CT-08 | SLC7A2 | Predicted | 0.036 | RS-05 | TRDMT1 | Predicted | 0.004 |
| CT-09 | ACADSB | Predicted | 0.004 | RS-06 | ABCB1 | Predicted | 0.007 |
| CT-09 | ACAT2 | Predicted | 0.059 | RS-06 | ACSL1 | Predicted | 0.007 |
| CT-09 | ADH1C | Predicted | 0.358 | RS-06 | AGXT | Predicted | 0.006 |
| CT-09 | AGXT | Predicted | 0.008 | RS-06 | AKR1B10 | Predicted | 0.089 |
| CT-09 | ALAS1 | Predicted | 0.008 | RS-06 | AKT2 | Predicted | 0.061 |
| CT-09 | ALDH1A1 | Predicted | 0.028 | RS-06 | ALDH1A1 | Predicted | 0.028 |
| CT-09 | ALDOA | Predicted | 0.052 | RS-06 | ASS1 | Predicted | 0.007 |
| CT-09 | ALDOB | Predicted | 0.147 | RS-06 | AURKA | Predicted | 0.004 |
| CT-09 | ARG1 | Predicted | 0.152 | RS-06 | CCNA2 | Predicted | 1.000 |
| CT-09 | ASS1 | Predicted | 0.030 | RS-06 | CCT3 | Predicted | 0.012 |
| CT-09 | AXIN1 | Predicted | 0.028 | RS-06 | CDK4 | Predicted | 0.034 |
| CT-09 | B2M | Predicted | 0.095 | RS-06 | CTSB | Predicted | 0.087 |
| CT-09 | BHMT | Predicted | 0.076 | RS-06 | ESR1 | Predicted | 0.176 |
| CT-09 | C1R | Predicted | 0.019 | RS-06 | GLUD1 | Predicted | 0.016 |
| CT-09 | CA1 | Predicted | 0.063 | RS-06 | GNMT | Predicted | 0.005 |
| CT-09 | CA2 | Predicted | 0.018 | RS-06 | GSTP1 | Predicted | 0.026 |
| CT-09 | CA3 | Predicted | 0.018 | RS-06 | HSP90AA1 | Predicted | 0.004 |
| CT-09 | CBS | Predicted | 0.044 | RS-06 | HSP90B1 | Predicted | 0.004 |
| CT-09 | CES1 | Predicted | 0.140 | RS-06 | HSPA1B | Predicted | 0.004 |
| CT-09 | CTH | Predicted | 0.008 | RS-06 | HSPA8 | Predicted | 0.004 |

| CT-09 | FGA | Predicted | 0.019 | RS-06 | KRAS | Predicted | 0.047 |
| --- | --- | --- | --- | --- | --- | --- | --- |
| CT-09 | FGF2 | Predicted | 0.486 | RS-06 | LYZ | Predicted | 0.088 |
| CT-09 | FTCD | Predicted | 0.042 | RS-06 | MAPK1 | Predicted | 0.090 |
| CT-09 | GLUD1 | Predicted | 0.005 | RS-06 | MAPK3 | Predicted | 0.034 |
| CT-09 | GLUL | Predicted | 0.005 | RS-06 | MAT1A | Predicted | 0.004 |
| CT-09 | GNMT | Predicted | 0.002 | RS-06 | MME | Predicted | 0.136 |
| CT-09 | GSTP1 | Predicted | 0.122 | RS-06 | NME1 | Predicted | 0.004 |
| CT-09 | HAO1 | Predicted | 0.324 | RS-06 | NOS2A | Predicted | 0.087 |
| CT-09 | IGF2R | Predicted | 0.073 | RS-06 | PGK1 | Predicted | 0.004 |
| CT-09 | LCN2 | Predicted | 0.019 | RS-06 | PTGS2 | Predicted | 0.023 |
| CT-09 | LYZ | Predicted | 0.019 | RS-06 | PTK2 | Predicted | 0.004 |
| CT-09 | MAPK1 | Predicted | 0.022 | RS-06 | RARA | Predicted | 0.049 |
| CT-09 | MIF | Predicted | 0.066 | RS-06 | RBP1 | Predicted | 0.009 |
| CT-09 | MMP2 | Predicted | 0.082 | RS-06 | RND3 | Predicted | 0.008 |
| CT-09 | MMP9 | Predicted | 0.070 | RS-06 | SERPINE1 | Predicted | 0.022 |
| CT-09 | MTR | Predicted | 0.046 | RS-06 | SRC | Predicted | 0.087 |
| CT-09 | NOS2A | Predicted | 0.149 | RS-06 | TOP2A | Predicted | 0.051 |
| CT-09 | OAT | Predicted | 0.067 | RS-06 | TRDMT1 | Predicted | 0.005 |
| CT-09 | PGK1 | Predicted | 0.048 | RS-07 | ABCB1 | Predicted | 0.004 |
| CT-09 | PLG | Predicted | 0.378 | RS-07 | ACSL1 | Predicted | 0.004 |
| CT-09 | PPIB | Predicted | 0.028 | RS-07 | AKT2 | Predicted | 0.156 |
| CT-09 | PRDX6 | Predicted | 0.035 | RS-07 | ASS1 | Predicted | 0.004 |
| CT-09 | PTGS2 | Predicted | 0.018 | RS-07 | CCNA2 | Predicted | 1.000 |
| CT-09 | PYGB | Predicted | 0.008 | RS-07 | CCT3 | Predicted | 0.007 |
| CT-09 | QDPR | Predicted | 0.035 | RS-07 | CDK4 | Predicted | 0.036 |

| CT-09 | REG1A | Predicted | 0.027 | RS-07 | CTSB | Predicted | 0.095 |
| --- | --- | --- | --- | --- | --- | --- | --- |
| CT-09 | SERPINC1 | Predicted | 0.047 | RS-07 | EGFR | Predicted | 0.093 |
| CT-09 | SLC7A2 | Predicted | 0.090 | RS-07 | ESR1 | Predicted | 0.093 |
| CT-09 | SOD2 | Predicted | 0.056 | RS-07 | GABRE | Predicted | 0.000 |
| CT-09 | SRC | Predicted | 0.002 | RS-07 | GAPDH | Predicted | 0.005 |
| CT-09 | TAOK2 | Predicted | 0.027 | RS-07 | GLUD1 | Predicted | 0.010 |
| CT-09 | TST | Predicted | 0.015 | RS-07 | GNMT | Predicted | 0.004 |
| CT-10 | ALDH1A1 | Predicted | 0.053 | RS-07 | GSTP1 | Predicted | 0.028 |
| CT-10 | ALDOA | Predicted | 0.087 | RS-07 | HAO1 | Predicted | 0.000 |
| CT-10 | ALDOB | Predicted | 0.215 | RS-07 | KRAS | Predicted | 0.057 |
| CT-10 | ARG1 | Predicted | 0.113 | RS-07 | MAPK1 | Predicted | 0.101 |
| CT-10 | ASS1 | Predicted | 0.058 | RS-07 | MAPK3 | Predicted | 0.036 |
| CT-10 | AXIN1 | Predicted | 0.053 | RS-07 | MME | Predicted | 0.147 |
| CT-10 | B2M | Predicted | 0.000 | RS-07 | MMP12 | Predicted | 0.036 |
| CT-10 | BHMT | Predicted | 0.106 | RS-07 | NOS2A | Predicted | 0.096 |
| CT-10 | CA1 | Predicted | 0.448 | RS-07 | PTGS2 | Predicted | 0.026 |
| CT-10 | CES1 | Predicted | 0.618 | RS-07 | RND3 | Predicted | 0.010 |
| CT-10 | FGF2 | Predicted | 0.452 | RS-07 | SERPINE1 | Predicted | 0.024 |
| CT-10 | FTCD | Predicted | 0.020 | RS-07 | SGK1 | Predicted | 0.000 |
| CT-10 | GLUD1 | Predicted | 0.020 | RS-07 | SRC | Predicted | 0.128 |
| CT-10 | GLUL | Predicted | 0.020 | RS-07 | TOP2A | Predicted | 0.061 |
| CT-10 | GNMT | Predicted | 0.013 | RS-07 | TRDMT1 | Predicted | 0.004 |
| CT-10 | GSTP1 | Predicted | 0.042 | RS-07 | VEGFA | Predicted | 0.016 |
| CT-10 | HAO1 | Predicted | 0.205 | RS-08 | ABCB1 | Predicted | 0.018 |
| CT-10 | LYZ | Predicted | 0.453 | RS-08 | ACSL1 | Predicted | 0.018 |

| CT-10 | MAPK1 | Predicted | 0.028 | RS-08 | ADH1C | Predicted | 0.012 |
| --- | --- | --- | --- | --- | --- | --- | --- |
| CT-10 | MIF | Predicted | 0.090 | RS-08 | AGXT | Predicted | 0.013 |
| CT-10 | MTR | Predicted | 0.073 | RS-08 | AKT2 | Predicted | 0.387 |
| CT-10 | NOS2A | Predicted | 0.212 | RS-08 | ASS1 | Predicted | 0.018 |
| CT-10 | OAT | Predicted | 0.058 | RS-08 | CA1 | Predicted | 0.058 |
| CT-10 | PGK1 | Predicted | 0.082 | RS-08 | CA2 | Predicted | 0.058 |
| CT-10 | PLG | Predicted | 0.473 | RS-08 | CASP1 | Predicted | 0.037 |
| CT-10 | PPIB | Predicted | 0.053 | RS-08 | CCNA2 | Predicted | 0.777 |
| CT-10 | PRDX6 | Predicted | 0.042 | RS-08 | CDK4 | Predicted | 0.047 |
| CT-10 | PTGS2 | Predicted | 0.218 | RS-08 | CES1 | Predicted | 0.036 |
| CT-10 | QDPR | Predicted | 0.042 | RS-08 | CTSB | Predicted | 0.194 |
| CT-10 | REG1A | Predicted | 0.030 | RS-08 | ESR1 | Predicted | 0.395 |
| CT-10 | SERPINC1 | Predicted | 0.000 | RS-08 | FGF1 | Predicted | 0.095 |
| CT-10 | SLC7A2 | Predicted | 0.136 | RS-08 | FGF2 | Predicted | 0.095 |
| CT-10 | SOD2 | Predicted | 0.075 | RS-08 | GABRE | Predicted | 0.005 |
| CT-10 | SRC | Predicted | 0.013 | RS-08 | GAPDH | Predicted | 0.040 |
| CT-10 | TAOK2 | Predicted | 0.030 | RS-08 | GLUD1 | Predicted | 0.038 |
| CT-10 | TST | Predicted | 0.035 | RS-08 | GNMT | Predicted | 0.008 |
| CT-11 | AKR1B10 | Predicted | 0.000 | RS-08 | GSTP1 | Predicted | 0.044 |
| CT-11 | ADH1B | Predicted | 0.000 | RS-08 | HAO1 | Predicted | 0.000 |
| CT-11 | EGFR | Predicted | 0.937 | RS-08 | KRAS | Predicted | 0.126 |
| CT-11 | MTHFD1 | Predicted | 0.000 | RS-08 | LYZ | Predicted | 0.090 |
| CT-11 | AKR1C2 | Predicted | 0.000 | RS-08 | MAPK1 | Predicted | 0.088 |
| CT-11 | DCXR | Predicted | 0.000 | RS-08 | MAPK3 | Predicted | 0.047 |
| CT-12 | ABCB1 | P08183 | 0.014 | RS-08 | MET | Predicted | 0.188 |

| CT-12 | ACSL1 | P33121 | 0.014 | RS-08 | MME | Predicted | 0.129 |
| --- | --- | --- | --- | --- | --- | --- | --- |
| CT-12 | ADH1C | P00326 | 0.013 | RS-08 | MMP12 | Predicted | 0.165 |
| CT-12 | AGXT | P21549 | 0.019 | RS-08 | MMP9 | Predicted | 0.037 |
| CT-12 | AKR1B10 | O60218 | 0.293 | RS-08 | NME1 | Predicted | 0.188 |
| CT-12 | AKT2 | P31751 | 0.308 | RS-08 | NOS2A | Predicted | 0.038 |
| CT-12 | ASS1 | P00966 | 0.014 | RS-08 | PTGS2 | Predicted | 0.078 |
| CT-12 | AURKA | O14965 | 0.010 | RS-08 | RND3 | Predicted | 0.038 |
| CT-12 | CA1 | P00915 | 0.144 | RS-08 | SGK1 | Predicted | 0.000 |
| CT-12 | CA2 | P00918 | 0.144 | RS-08 | SRC | Predicted | 0.093 |
| CT-12 | CCT3 | P49368 | 0.010 | RS-08 | SULT2A1 | Predicted | 0.035 |
| CT-12 | CES1 | P23141 | 0.013 | RS-08 | TOP2A | Predicted | 0.067 |
| CT-12 | CTSB | P07858 | 0.304 | RS-08 | TRDMT1 | Predicted | 0.008 |
| CT-12 | ESR1 | P03372 | 0.313 | RS-08 | VEGFA | Predicted | 0.081 |
| CT-12 | GLUD1 | P00367 | 0.038 | RS-09 | CASP3 | Validated | 1.000 |
| CT-12 | GNAI1 | P63096 | 0.014 | RS-09 | ABCB1 | Predicted | 0.014 |
| CT-12 | GSTP1 | P09211 | 0.091 | RS-09 | ACSL1 | Predicted | 0.014 |
| CT-12 | HAO1 | Q9UJM8 | 0.000 | RS-09 | ADH1B | Predicted | 0.009 |
| CT-12 | HSP90AA1 | P07900 | 0.010 | RS-09 | ADH1C | Predicted | 0.019 |
| CT-12 | HSP90B1 | P14625 | 0.010 | RS-09 | ADH4 | Predicted | 0.009 |
| CT-12 | HSPA1B | P08107 | 0.010 | RS-09 | AKR1C2 | Predicted | 0.001 |
| CT-12 | HSPA8 | P11142 | 0.010 | RS-09 | AKT2 | Predicted | 0.105 |
| CT-12 | KRAS | P01116 | 0.085 | RS-09 | ALDH1A1 | Predicted | 0.009 |
| CT-12 | MAT1A | Q00266 | 0.010 | RS-09 | ALDH1B1 | Predicted | 0.001 |
| CT-12 | MME | P08473 | 0.199 | RS-09 | ALDH1L1 | Predicted | 0.000 |
| CT-12 | NME1 | P15531 | 0.061 | RS-09 | ALDH2 | Predicted | 0.009 |

| CT-12 | NME2 | P22392 | 0.014 | RS-09 | ALDH3A2 | Predicted | 0.001 |
| --- | --- | --- | --- | --- | --- | --- | --- |
| CT-12 | NOS2A | P35228 | 0.293 | RS-09 | ALDH4A1 | Predicted | 0.001 |
| CT-12 | PGK1 | P00558 | 0.010 | RS-09 | ASS1 | Predicted | 0.014 |
| CT-12 | PTK2 | Q05397 | 0.010 | RS-09 | AURKA | Predicted | 0.006 |
| CT-12 | RHOA | P61586 | 0.014 | RS-09 | CASP1 | Predicted | 0.034 |
| CT-12 | RND3 | P61587 | 0.015 | RS-09 | CCNA2 | Predicted | 0.283 |
| CT-12 | SERPINE1 | P05121 | 0.024 | RS-09 | CCT3 | Predicted | 0.021 |
| CT-12 | SGK1 | O00141 | 0.000 | RS-09 | CDK4 | Predicted | 0.043 |
| CT-12 | TGM2 | P21980 | 0.014 | RS-09 | CES1 | Predicted | 0.003 |
| CT-12 | TOP2A | P11388 | 0.069 | RS-09 | ESR1 | Predicted | 0.136 |
| CT-13 | ACADSB | Predicted | 0.004 | RS-09 | FTCD | Predicted | 0.000 |
| CT-13 | ACAT2 | Predicted | 0.059 | RS-09 | GAPDH | Predicted | 0.029 |
| CT-13 | ADH1C | Predicted | 0.358 | RS-09 | GLUD1 | Predicted | 0.034 |
| CT-13 | AGXT | Predicted | 0.008 | RS-09 | GNAI1 | Predicted | 0.004 |
| CT-13 | ALAS1 | Predicted | 0.008 | RS-09 | GNMT | Predicted | 0.004 |
| CT-13 | ALDH1A1 | Predicted | 0.028 | RS-09 | GSTP1 | Predicted | 0.115 |
| CT-13 | ALDOA | Predicted | 0.052 | RS-09 | HAO1 | Predicted | 0.003 |
| CT-13 | ALDOB | Predicted | 0.147 | RS-09 | HSD17B10 | Predicted | 0.009 |
| CT-13 | ARG1 | Predicted | 0.152 | RS-09 | HSD17B4 | Predicted | 0.009 |
| CT-13 | ASS1 | Predicted | 0.030 | RS-09 | HSP90AA1 | Predicted | 0.006 |
| CT-13 | AXIN1 | Predicted | 0.028 | RS-09 | HSP90B1 | Predicted | 0.006 |
| CT-13 | B2M | Predicted | 0.095 | RS-09 | HSPA1B | Predicted | 0.006 |
| CT-13 | BHMT | Predicted | 0.076 | RS-09 | HSPA8 | Predicted | 0.006 |
| CT-13 | C1R | Predicted | 0.019 | RS-09 | KRAS | Predicted | 0.083 |
| CT-13 | CA1 | Predicted | 0.063 | RS-09 | MAPK1 | Predicted | 0.120 |

| CT-13 | CA2 | Predicted | 0.018 | RS-09 | MAPK3 | Predicted | 0.043 |
| --- | --- | --- | --- | --- | --- | --- | --- |
| CT-13 | CA3 | Predicted | 0.018 | RS-09 | MAT1A | Predicted | 0.006 |
| CT-13 | CBS | Predicted | 0.044 | RS-09 | MET | Predicted | 0.134 |
| CT-13 | CES1 | Predicted | 0.140 | RS-09 | MME | Predicted | 0.209 |
| CT-13 | CTH | Predicted | 0.008 | RS-09 | MMP12 | Predicted | 0.054 |
| CT-13 | FGA | Predicted | 0.019 | RS-09 | MMP9 | Predicted | 0.034 |
| CT-13 | FGF2 | Predicted | 0.486 | RS-09 | MTHFD1 | Predicted | 0.008 |
| CT-13 | FTCD | Predicted | 0.042 | RS-09 | MTR | Predicted | 0.000 |
| CT-13 | GLUD1 | Predicted | 0.005 | RS-09 | NME1 | Predicted | 0.028 |
| CT-13 | GLUL | Predicted | 0.005 | RS-09 | NME2 | Predicted | 0.004 |
| CT-13 | GNMT | Predicted | 0.002 | RS-09 | PGK1 | Predicted | 0.006 |
| CT-13 | GSTP1 | Predicted | 0.122 | RS-09 | PTK2 | Predicted | 0.006 |
| CT-13 | HAO1 | Predicted | 0.324 | RS-09 | QDPR | Predicted | 0.009 |
| CT-13 | IGF2R | Predicted | 0.073 | RS-09 | RHOA | Predicted | 0.004 |
| CT-13 | LCN2 | Predicted | 0.019 | RS-09 | RND3 | Predicted | 0.005 |
| CT-13 | LYZ | Predicted | 0.019 | RS-09 | SERPINE1 | Predicted | 0.009 |
| CT-13 | MAPK1 | Predicted | 0.022 | RS-09 | SGK1 | Predicted | 0.003 |
| CT-13 | MIF | Predicted | 0.066 | RS-09 | SRC | Predicted | 0.065 |
| CT-13 | MMP2 | Predicted | 0.082 | RS-09 | TGM2 | Predicted | 0.004 |
| CT-13 | MMP9 | Predicted | 0.070 | RS-09 | TOP2A | Predicted | 0.201 |
| CT-13 | MTR | Predicted | 0.046 | RS-09 | TRDMT1 | Predicted | 0.004 |
| CT-13 | NOS2A | Predicted | 0.149 | RS-09 | TUBB | Predicted | 0.006 |
| CT-13 | OAT | Predicted | 0.067 | RS-09 | VEGFA | Predicted | 0.065 |
| CT-13 | PGK1 | Predicted | 0.048 | RS-10 | ABCB1 | Predicted | 0.014 |
| CT-13 | PLG | Predicted | 0.378 | RS-10 | ACSL1 | Predicted | 0.014 |

| CT-13 | PPIB | Predicted | 0.028 | RS-10 | ADH1B | Predicted | 0.009 |
| --- | --- | --- | --- | --- | --- | --- | --- |
| CT-13 | PRDX6 | Predicted | 0.035 | RS-10 | ADH1C | Predicted | 0.019 |
| CT-13 | PTGS2 | Predicted | 0.018 | RS-10 | ADH4 | Predicted | 0.009 |
| CT-13 | PYGB | Predicted | 0.008 | RS-10 | AKR1C2 | Predicted | 0.001 |
| CT-13 | QDPR | Predicted | 0.035 | RS-10 | AKT2 | Predicted | 0.105 |
| CT-13 | REG1A | Predicted | 0.027 | RS-10 | ALDH1A1 | Predicted | 0.009 |
| CT-13 | SERPINC1 | Predicted | 0.047 | RS-10 | ALDH1B1 | Predicted | 0.001 |
| CT-13 | SLC7A2 | Predicted | 0.090 | RS-10 | ALDH1L1 | Predicted | 0.000 |
| CT-13 | SOD2 | Predicted | 0.056 | RS-10 | ALDH2 | Predicted | 0.009 |
| CT-13 | SRC | Predicted | 0.002 | RS-10 | ALDH3A2 | Predicted | 0.001 |
| CT-13 | TAOK2 | Predicted | 0.027 | RS-10 | ALDH4A1 | Predicted | 0.001 |
| CT-13 | TST | Predicted | 0.015 | RS-10 | ASS1 | Predicted | 0.014 |
| CT-14 | ACAT2 | Predicted | 0.166 | RS-10 | AURKA | Predicted | 0.006 |
| CT-14 | ASPH | Predicted | 0.087 | RS-10 | CASP1 | Predicted | 0.034 |
| CT-14 | ASS1 | Predicted | 0.039 | RS-10 | CCNA2 | Predicted | 0.283 |
| CT-14 | CBS | Predicted | 0.143 | RS-10 | CCT3 | Predicted | 0.021 |
| CT-14 | CTH | Predicted | 0.030 | RS-10 | CDK4 | Predicted | 0.043 |
| CT-14 | DCN | Predicted | 0.034 | RS-10 | CES1 | Predicted | 0.003 |
| CT-14 | EGF | Predicted | 0.303 | RS-10 | ESR1 | Predicted | 0.136 |
| CT-14 | FTCD | Predicted | 0.023 | RS-10 | FTCD | Predicted | 0.000 |
| CT-14 | GLUD1 | Predicted | 0.023 | RS-10 | GAPDH | Predicted | 0.029 |
| CT-14 | GLUL | Predicted | 0.023 | RS-10 | GLUD1 | Predicted | 0.034 |
| CT-14 | GSTP1 | Predicted | 0.166 | RS-10 | GNAI1 | Predicted | 0.004 |
| CT-14 | HSD17B6 | Predicted | 0.001 | RS-10 | GNMT | Predicted | 0.004 |
| CT-14 | LYZ | Predicted | 0.039 | RS-10 | GSTP1 | Predicted | 0.115 |

| CT-14 | MGMT | Predicted | 0.030 | RS-10 | HAO1 | Predicted | 0.003 |
| --- | --- | --- | --- | --- | --- | --- | --- |
| CT-14 | NME1 | Predicted | 0.013 | RS-10 | HSD17B10 | Predicted | 0.009 |
| CT-14 | PKM2 | Predicted | 0.038 | RS-10 | HSD17B4 | Predicted | 0.009 |
| CT-14 | PPIB | Predicted | 0.000 | RS-10 | HSP90AA1 | Predicted | 0.006 |
| CT-14 | VEGFA | Predicted | 0.034 | RS-10 | HSP90B1 | Predicted | 0.006 |
| CT-15 | ADH1C | predicted | 0.394 | RS-10 | HSPA1B | Predicted | 0.006 |
| CT-15 | SRC | predicted | 0.000 | RS-10 | HSPA8 | Predicted | 0.006 |
| CT-16 | AGXT | predicted | 0.000 | RS-10 | KRAS | Predicted | 0.083 |
| CT-16 | ALAS1 | predicted | 0.000 | RS-10 | MAPK1 | Predicted | 0.120 |
| CT-16 | ARG1 | predicted | 0.085 | RS-10 | MAPK3 | Predicted | 0.043 |
| CT-16 | ASS1 | predicted | 0.038 | RS-10 | MAT1A | Predicted | 0.006 |
| CT-16 | B2M | predicted | 0.056 | RS-10 | MET | Predicted | 0.134 |
| CT-16 | CA1 | predicted | 0.082 | RS-10 | MME | Predicted | 0.209 |
| CT-16 | CA2 | predicted | 0.054 | RS-10 | MMP12 | Predicted | 0.054 |
| CT-16 | CA3 | predicted | 0.054 | RS-10 | MMP9 | Predicted | 0.034 |
| CT-16 | CBS | predicted | 0.000 | RS-10 | MTHFD1 | Predicted | 0.008 |
| CT-16 | CCNA2 | predicted | 0.182 | RS-10 | MTR | Predicted | 0.000 |
| CT-16 | CES1 | predicted | 0.187 | RS-10 | NME1 | Predicted | 0.028 |
| CT-16 | CTH | predicted | 0.000 | RS-10 | NME2 | Predicted | 0.004 |
| CT-16 | DNMT1 | predicted | 0.088 | RS-10 | PGK1 | Predicted | 0.006 |
| CT-16 | FTCD | predicted | 0.000 | RS-10 | PTK2 | Predicted | 0.006 |
| CT-16 | GAPDH | predicted | 0.189 | RS-10 | QDPR | Predicted | 0.009 |
| CT-16 | HAO1 | predicted | 0.383 | RS-10 | RHOA | Predicted | 0.004 |
| CT-16 | LCN2 | predicted | 0.189 | RS-10 | RND3 | Predicted | 0.005 |
| CT-16 | MAPK1 | predicted | 0.042 | RS-10 | SERPINE1 | Predicted | 0.009 |

| CT-16 | NOS2A | predicted | 0.203 | RS-10 | SGK1 | Predicted | 0.003 |
| --- | --- | --- | --- | --- | --- | --- | --- |
| CT-16 | OAT | predicted | 0.000 | RS-10 | SRC | Predicted | 0.065 |
| CT-16 | PC | predicted | 0.072 | RS-10 | TGM2 | Predicted | 0.004 |
| CT-16 | PIN1 | predicted | 0.090 | RS-10 | TOP2A | Predicted | 0.201 |
| CT-16 | PTGS2 | predicted | 1.000 | RS-10 | TRDMT1 | Predicted | 0.004 |
| CT-16 | PYGB | predicted | 0.000 | RS-10 | TUBB | Predicted | 0.006 |
| CT-16 | SERPINC1 | predicted | 0.011 | RS-10 | VEGFA | Predicted | 0.065 |
| CT-16 | SLC7A2 | predicted | 0.035 | RS-11 | ABCB1 | Predicted | 0.003 |
| CT-16 | SRC | predicted | 0.088 | RS-11 | ACSL1 | Predicted | 0.003 |
| CT-16 | TNFSF11 | predicted | 0.057 | RS-11 | AKR1B10 | Predicted | 0.093 |
| CT-16 | TOP2A | predicted | 0.186 | RS-11 | AKT2 | Predicted | 0.154 |
| CT-17 | ACADSB | predicted | 0.881 | RS-11 | ASS1 | Predicted | 0.003 |
| CT-17 | ACAT2 | predicted | 0.895 | RS-11 | CASP1 | Predicted | 0.014 |
| CT-17 | ALDH1A1 | predicted | 0.871 | RS-11 | CCNA2 | Predicted | 1.000 |
| CT-17 | ALDOA | predicted | 0.893 | RS-11 | CCT3 | Predicted | 0.006 |
| CT-17 | ALDOB | predicted | 0.893 | RS-11 | CDK4 | Predicted | 0.036 |
| CT-17 | ARG1 | predicted | 0.891 | RS-11 | CTSB | Predicted | 0.094 |
| CT-17 | ASPH | predicted | 0.922 | RS-11 | ESR1 | Predicted | 0.094 |
| CT-17 | ASS1 | predicted | 0.922 | RS-11 | GABRE | Predicted | 0.000 |
| CT-17 | AXIN1 | predicted | 0.871 | RS-11 | GLUD1 | Predicted | 0.012 |
| CT-17 | BHMT | predicted | 0.866 | RS-11 | GNMT | Predicted | 0.004 |
| CT-17 | CBS | predicted | 0.917 | RS-11 | HAO1 | Predicted | 0.000 |
| CT-17 | CDC25B | predicted | 0.914 | RS-11 | KRAS | Predicted | 0.057 |
| CT-17 | CTH | predicted | 0.917 | RS-11 | MAPK1 | Predicted | 0.100 |
| CT-17 | DCN | predicted | 0.854 | RS-11 | MAPK3 | Predicted | 0.036 |

| CT-17 | EGF | predicted | 0.937 | RS-11 | MME | Predicted | 0.146 |
| --- | --- | --- | --- | --- | --- | --- | --- |
| CT-17 | FTCD | predicted | 0.864 | RS-11 | MMP9 | Predicted | 0.014 |
| CT-17 | GLUD1 | predicted | 0.864 | RS-11 | NME1 | Predicted | 0.093 |
| CT-17 | GLUL | predicted | 0.864 | RS-11 | PTGS2 | Predicted | 0.041 |
| CT-17 | GNMT | predicted | 0.866 | RS-11 | RND3 | Predicted | 0.012 |
| CT-17 | GSTP1 | predicted | 0.895 | RS-11 | SERPINE1 | Predicted | 0.017 |
| CT-17 | HSD17B6 | predicted | 0.915 | RS-11 | SGK1 | Predicted | 0.000 |
| CT-17 | LYZ | predicted | 0.922 | RS-11 | SRC | Predicted | 0.128 |
| CT-17 | MGMT | predicted | 0.917 | RS-11 | TOP2A | Predicted | 0.055 |
| CT-17 | MIF | predicted | 0.866 | RS-11 | TRDMT1 | Predicted | 0.004 |
| CT-17 | NME1 | predicted | 0.938 | RS-11 | VEGFA | Predicted | 0.038 |
| CT-17 | NOS2A | predicted | 0.863 | RS-12 | ABCB1 | Predicted | 0.011 |
| CT-17 | OAT | predicted | 0.891 | RS-12 | ACSL1 | Predicted | 0.011 |
| CT-17 | PCK1 | predicted | 0.898 | RS-12 | AKT2 | Predicted | 0.048 |
| CT-17 | PGK1 | predicted | 0.891 | RS-12 | ALDH1L1 | Predicted | 0.012 |
| CT-17 | PKM2 | predicted | 0.950 | RS-12 | ASS1 | Predicted | 0.011 |
| CT-17 | PPIB | predicted | 0.891 | RS-12 | AURKA | Predicted | 0.129 |
| CT-17 | REG1A | predicted | 0.851 | RS-12 | CCNA2 | Predicted | 0.951 |
| CT-17 | SLC7A2 | predicted | 0.891 | RS-12 | CCT3 | Predicted | 0.008 |
| CT-17 | SRC | predicted | 0.866 | RS-12 | CYP2C8 | Predicted | 0.017 |
| CT-17 | TAOK2 | predicted | 0.851 | RS-12 | DDR1 | Predicted | 0.011 |
| CT-17 | VEGFA | predicted | 0.880 | RS-12 | EGFR | Predicted | 0.130 |
| CT-18 | ADH1C | predicted | 0.442 | RS-12 | ESR1 | Predicted | 0.040 |
| CT-18 | ASPH | predicted | 0.000 | RS-12 | FTCD | Predicted | 0.012 |
| CT-18 | CBS | predicted | 0.131 | RS-12 | GLUD1 | Predicted | 0.014 |

| CT-19 | CDC25B | predicted | 0.038 | RS-12 | GNMT | Predicted | 0.005 |
| --- | --- | --- | --- | --- | --- | --- | --- |
| CT-19 | CTH | predicted | 0.019 | RS-12 | GSTP1 | Predicted | 0.089 |
| CT-19 | DCN | predicted | 0.015 | RS-12 | KRAS | Predicted | 0.030 |
| CT-19 | EGF | predicted | 0.303 | RS-12 | MAPK1 | Predicted | 0.012 |
| CT-19 | FTCD | predicted | 0.014 | RS-12 | MME | Predicted | 0.132 |
| CT-19 | GLUD1 | predicted | 0.014 | RS-12 | MTHFD1 | Predicted | 0.012 |
| CT-19 | GLUL | predicted | 0.014 | RS-12 | MTR | Predicted | 0.012 |
| CT-19 | GSTP1 | predicted | 0.111 | RS-12 | NME1 | Predicted | 0.134 |
| CT-19 | HSD17B6 | predicted | 0.000 | RS-12 | NOS2A | Predicted | 0.130 |
| CT-19 | LYZ | predicted | 0.101 | RS-12 | PDGFRA | Predicted | 0.011 |
| CT-19 | MGMT | predicted | 0.019 | RS-12 | PTGS2 | Predicted | 0.029 |
| CT-19 | NME1 | predicted | 0.005 | RS-12 | RARA | Predicted | 0.018 |
| CT-19 | NOS2A | predicted | 0.297 | RS-12 | RND3 | Predicted | 0.014 |
| CT-19 | PCK1 | predicted | 0.107 | RS-12 | SERPINE1 | Predicted | 0.028 |
| CT-19 | PGK1 | predicted | 0.100 | RS-12 | TRDMT1 | Predicted | 0.005 |
| CT-19 | PKM2 | predicted | 0.076 | RS-12 | VEGFA | Predicted | 0.030 |
| CT-19 | PPIB | predicted | 0.068 | RS-13 | ABCB1 | Predicted | 0.010 |
| CT-19 | REG1A | predicted | 0.010 | RS-13 | ACSL1 | Predicted | 0.010 |
| CT-19 | TAOK2 | predicted | 0.010 | RS-13 | AKT2 | Predicted | 0.075 |
| CT-19 | TST | predicted | 0.040 | RS-13 | ALDH1A1 | Predicted | 0.025 |
| CT-19 | VEGFA | predicted | 0.058 | RS-13 | ALDH1L1 | Predicted | 0.009 |
| CT-20 | AGXT | predicted | 0.007 | RS-13 | ASS1 | Predicted | 0.010 |
| CT-20 | ALAS1 | predicted | 0.007 | RS-13 | CCNA2 | Predicted | 1.000 |
| CT-20 | ALDOA | predicted | 0.098 | RS-13 | CCT3 | Predicted | 0.006 |
| CT-20 | ALDOB | predicted | 0.098 | RS-13 | CDK4 | Predicted | 0.025 |

| CT-20 | ARG1 | predicted | 0.100 | RS-13 | CYP2C8 | Predicted | 0.009 |
| --- | --- | --- | --- | --- | --- | --- | --- |
| CT-20 | ASS1 | predicted | 0.036 | RS-13 | EGFR | Predicted | 0.097 |
| CT-20 | B2M | predicted | 0.066 | RS-13 | ESR1 | Predicted | 0.096 |
| CT-20 | BHMT | predicted | 0.073 | RS-13 | FTCD | Predicted | 0.009 |
| CT-20 | CA1 | predicted | 0.397 | RS-13 | GAPDH | Predicted | 0.014 |
| CT-20 | CA2 | predicted | 0.397 | RS-13 | GLUD1 | Predicted | 0.008 |
| CT-20 | CA3 | predicted | 0.084 | RS-13 | GNMT | Predicted | 0.004 |
| CT-20 | CBS | predicted | 0.007 | RS-13 | GSTP1 | Predicted | 0.029 |
| CT-20 | CES1 | predicted | 0.237 | RS-13 | KRAS | Predicted | 0.053 |
| CT-20 | CTH | predicted | 0.007 | RS-13 | LYZ | Predicted | 0.094 |
| CT-20 | FGF1 | predicted | 0.059 | RS-13 | MAPK1 | Predicted | 0.045 |
| CT-20 | FGF2 | predicted | 0.304 | RS-13 | MAPK3 | Predicted | 0.025 |
| CT-20 | FTCD | predicted | 0.007 | RS-13 | MME | Predicted | 0.149 |
| CT-20 | GAPDH | predicted | 0.232 | RS-13 | MTHFD1 | Predicted | 0.009 |
| CT-20 | GSTP1 | predicted | 0.022 | RS-13 | MTR | Predicted | 0.009 |
| CT-20 | HAO1 | predicted | 0.214 | RS-13 | NME1 | Predicted | 0.093 |
| CT-20 | HGF | predicted | 0.059 | RS-13 | NOS2A | Predicted | 0.099 |
| CT-20 | IGF2R | predicted | 0.049 | RS-13 | PTGS2 | Predicted | 0.024 |
| CT-20 | LCN2 | predicted | 0.217 | RS-13 | RAF1 | Predicted | 0.012 |
| CT-20 | MAPK1 | predicted | 0.037 | RS-13 | RARA | Predicted | 0.055 |
| CT-20 | MIF | predicted | 0.148 | RS-13 | RBP1 | Predicted | 0.008 |
| CT-20 | MMP2 | predicted | 0.041 | RS-13 | RND3 | Predicted | 0.008 |
| CT-20 | MTR | predicted | 0.073 | RS-13 | SERPINE1 | Predicted | 0.031 |
| CT-20 | NOS2A | predicted | 0.314 | RS-13 | SRC | Predicted | 0.093 |
| CT-20 | OAT | predicted | 0.007 | RS-13 | TRDMT1 | Predicted | 0.004 |

| CT-20 | PC | predicted | 0.097 | RS-14 | ABCB1 | Predicted | 0.011 |
| --- | --- | --- | --- | --- | --- | --- | --- |
| CT-20 | PIN1 | predicted | 0.073 | RS-14 | ACSL1 | Predicted | 0.011 |
| CT-20 | PLG | predicted | 0.102 | RS-14 | AGXT | Predicted | 0.025 |
| CT-20 | PRDX6 | predicted | 0.022 | RS-14 | AKT2 | Predicted | 0.382 |
| CT-20 | PTGS2 | predicted | 0.710 | RS-14 | ASS1 | Predicted | 0.011 |
| CT-20 | PYGB | predicted | 0.007 | RS-14 | AURKA | Predicted | 0.017 |
| CT-20 | QDPR | predicted | 0.022 | RS-14 | CA1 | Predicted | 0.154 |
| CT-20 | SERPINC1 | predicted | 0.000 | RS-14 | CA2 | Predicted | 0.154 |
| CT-20 | SLC7A2 | predicted | 0.030 | RS-14 | CCNA2 | Predicted | 0.645 |
| RAB-01 | ACSL1 | Predicted | 0.020 | RS-14 | CCT3 | Predicted | 0.017 |
| RAB-01 | AGXT | Predicted | 0.003 | RS-14 | CDK4 | Predicted | 0.085 |
| RAB-01 | AKR1B10 | Predicted | 0.107 | RS-14 | CTSB | Predicted | 0.324 |
| RAB-01 | AKT2 | Predicted | 0.059 | RS-14 | ESR1 | Predicted | 0.654 |
| RAB-01 | AURKA | Predicted | 0.000 | RS-14 | GLUD1 | Predicted | 0.042 |
| RAB-01 | CA1 | Predicted | 0.077 | RS-14 | GNAI1 | Predicted | 0.015 |
| RAB-01 | CA2 | Predicted | 0.077 | RS-14 | HAO1 | Predicted | 0.000 |
| RAB-01 | CCNA2 | Predicted | 1.000 | RS-14 | HSP90AA1 | Predicted | 0.017 |
| RAB-01 | CCND1 | Validated | 1.000 | RS-14 | HSP90B1 | Predicted | 0.017 |
| RAB-01 | CCT3 | Predicted | 0.000 | RS-14 | HSPA1B | Predicted | 0.017 |
| RAB-01 | CTSB | Predicted | 0.108 | RS-14 | HSPA8 | Predicted | 0.017 |
| RAB-01 | ESR1 | Predicted | 0.104 | RS-14 | KRAS | Predicted | 0.087 |
| RAB-01 | GABRE | Predicted | 0.011 | RS-14 | MAPK1 | Predicted | 0.181 |
| RAB-01 | GLUD1 | Predicted | 0.011 | RS-14 | MAPK3 | Predicted | 0.085 |
| RAB-01 | GNAI1 | Predicted | 0.002 | RS-14 | MAT1A | Predicted | 0.017 |
| RAB-01 | GNMT | Predicted | 0.005 | RS-14 | MME | Predicted | 0.216 |

| RAB-01 | GSTP1 | Predicted | 0.006 | RS-14 | MMP12 | Predicted | 0.105 |
| --- | --- | --- | --- | --- | --- | --- | --- |
| RAB-01 | HSP90AA1 | Predicted | 0.000 | RS-14 | NME1 | Predicted | 0.066 |
| RAB-01 | HSP90B1 | Predicted | 0.000 | RS-14 | NME2 | Predicted | 0.015 |
| RAB-01 | HSPA1B | Predicted | 0.000 | RS-14 | PGK1 | Predicted | 0.017 |
| RAB-01 | HSPA8 | Predicted | 0.000 | RS-14 | PTK2 | Predicted | 0.017 |
| RAB-01 | KRAS | Predicted | 0.042 | RS-14 | RHOA | Predicted | 0.015 |
| RAB-01 | LYZ | Predicted | 0.105 | RS-14 | RND3 | Predicted | 0.016 |
| RAB-01 | MAPK3 | Predicted | 0.038 | RS-14 | SGK1 | Predicted | 0.000 |
| RAB-01 | MAT1A | Predicted | 0.000 | RS-14 | SRC | Predicted | 0.323 |
| RAB-01 | MTAP | Predicted | 0.216 | RS-14 | TGM2 | Predicted | 0.015 |
| RAB-01 | NME1 | Predicted | 0.019 | RS-14 | TOP2A | Predicted | 0.079 |
| RAB-01 | NME2 | Predicted | 0.002 | RS-14 | VEGFA | Predicted | 0.055 |
| RAB-01 | NOS2A | Predicted | 0.111 | RS-15 | ABCB1 | Predicted | 0.009 |
| RAB-01 | PGK1 | Predicted | 0.000 | RS-15 | ACSL1 | Predicted | 0.009 |
| RAB-01 | PTGS2 | Predicted | 0.208 | RS-15 | AKT2 | Predicted | 0.173 |
| RAB-01 | PTK2 | Predicted | 0.000 | RS-15 | ALDH1A1 | Predicted | 0.003 |
| RAB-01 | RHOA | Predicted | 0.002 | RS-15 | ASS1 | Predicted | 0.009 |
| RAB-01 | RND3 | Predicted | 0.003 | RS-15 | CCNA2 | Predicted | 1.000 |
| RAB-01 | SERPINE1 | Predicted | 0.028 | RS-15 | CCT3 | Predicted | 0.009 |
| RAB-01 | SRC | Predicted | 0.105 | RS-15 | CDK4 | Predicted | 0.042 |
| RAB-01 | SULT2A1 | Predicted | 0.004 | RS-15 | CYP2C8 | Predicted | 0.009 |
| RAB-01 | TGM2 | Predicted | 0.002 | RS-15 | ESR1 | Predicted | 0.107 |
| RAB-01 | TOP2A | Predicted | 0.021 | RS-15 | GAPDH | Predicted | 0.016 |
| RAB-01 | TRDMT1 | Predicted | 0.005 | RS-15 | GLUD1 | Predicted | 0.011 |
| RAB-02 | ABCB1 | Predicted | 0.015 | RS-15 | GNMT | Predicted | 0.002 |
| RAB-02 | ACSL1 | Predicted | 0.015 | RS-15 | GSTP1 | Predicted | 0.092 |
| RAB-02 | AKT2 | Predicted | 0.124 | RS-15 | HAO1 | Predicted | 0.003 |
| RAB-02 | ALDH1L1 | Predicted | 0.012 | RS-15 | KRAS | Predicted | 0.052 |
| RAB-02 | ASS1 | Predicted | 0.015 | RS-15 | MAPK1 | Predicted | 0.113 |
| RAB-02 | AURKA | Predicted | 0.132 | RS-15 | MAPK3 | Predicted | 0.042 |
| RAB-02 | CCNA2 | Predicted | 0.408 | RS-15 | MME | Predicted | 0.166 |
| RAB-02 | CCT3 | Predicted | 0.007 | RS-15 | MMP12 | Predicted | 0.042 |
| RAB-02 | CYP2C8 | Predicted | 0.012 | RS-15 | NME1 | Predicted | 0.106 |
| RAB-02 | DDR1 | Predicted | 0.016 | RS-15 | NOS2A | Predicted | 0.106 |
| RAB-02 | EGFR | Predicted | 0.136 | RS-15 | RBP1 | Predicted | 0.003 |
| RAB-02 | ESR1 | Predicted | 0.133 | RS-15 | RND3 | Predicted | 0.011 |
| RAB-02 | FTCD | Predicted | 0.012 | RS-15 | SERPINE1 | Predicted | 0.032 |
| RAB-02 | GAPDH | Predicted | 0.019 | RS-15 | SGK1 | Predicted | 0.003 |
| RAB-02 | GLUD1 | Predicted | 0.015 | RS-15 | SRC | Predicted | 0.110 |
| RAB-02 | GSTP1 | Predicted | 0.023 | RS-15 | TOP2A | Predicted | 0.058 |
| RAB-02 | KRAS | Predicted | 0.055 | RS-15 | TRDMT1 | Predicted | 0.002 |
| RAB-02 | MAPK1 | Predicted | 0.011 | RS-15 | VEGFA | Predicted | 0.022 |
| RAB-02 | MME | Predicted | 0.132 | RS-16 | ABCB1 | Predicted | 0.006 |
| RAB-02 | MTHFD1 | Predicted | 0.012 | RS-16 | ACSL1 | Predicted | 0.032 |
| RAB-02 | MTR | Predicted | 0.012 | RS-16 | AGXT | Predicted | 0.002 |
| RAB-02 | PDGFRA | Predicted | 0.016 | RS-16 | AKT2 | Predicted | 0.178 |
| RAB-02 | PTGS2 | Predicted | 0.031 | RS-16 | ASS1 | Predicted | 0.006 |
| RAB-02 | RAF1 | Predicted | 0.016 | RS-16 | AURKA | Predicted | 0.003 |
| RAB-02 | RARA | Predicted | 0.018 | RS-16 | CCNA2 | Predicted | 0.986 |
| RAB-02 | RND3 | Predicted | 0.015 | RS-16 | CCT3 | Predicted | 0.018 |

| RAB-02 | SERPINE1 | Predicted | 0.026 | RS-16 | CDK4 | Predicted | 0.030 |
| --- | --- | --- | --- | --- | --- | --- | --- |
| RAB-02 | TUBB | Predicted | 0.007 | RS-16 | CES1 | Predicted | 0.039 |
| RAB-03 | ABCB1 | Predicted | 0.015 | RS-16 | CTSB | Predicted | 0.130 |
| RAB-03 | ACSL1 | Predicted | 0.015 | RS-16 | EGFR | Predicted | 0.030 |
| RAB-03 | AKT2 | Predicted | 0.124 | RS-16 | ESR1 | Predicted | 0.554 |
| RAB-03 | ALDH1L1 | Predicted | 0.012 | RS-16 | GLUD1 | Predicted | 0.025 |
| RAB-03 | ASS1 | Predicted | 0.015 | RS-16 | GNMT | Predicted | 0.002 |
| RAB-03 | AURKA | Predicted | 0.132 | RS-16 | HSP90AA1 | Predicted | 0.003 |
| RAB-03 | CCNA2 | Predicted | 0.408 | RS-16 | HSP90B1 | Predicted | 0.003 |
| RAB-03 | CCT3 | Predicted | 0.007 | RS-16 | HSPA1B | Predicted | 0.003 |
| RAB-03 | CYP2C8 | Predicted | 0.012 | RS-16 | HSPA8 | Predicted | 0.003 |
| RAB-03 | DDR1 | Predicted | 0.016 | RS-16 | KRAS | Predicted | 0.058 |
| RAB-03 | EGFR | Predicted | 0.136 | RS-16 | LYZ | Predicted | 0.060 |
| RAB-03 | ESR1 | Predicted | 0.133 | RS-16 | MAPK1 | Predicted | 0.005 |
| RAB-03 | FTCD | Predicted | 0.012 | RS-16 | MAT1A | Predicted | 0.003 |
| RAB-03 | GAPDH | Predicted | 0.019 | RS-16 | MME | Predicted | 0.084 |
| RAB-03 | GLUD1 | Predicted | 0.015 | RS-16 | MMP12 | Predicted | 0.039 |
| RAB-03 | GSTP1 | Predicted | 0.023 | RS-16 | MMP14 | Predicted | 0.009 |
| RAB-03 | KRAS | Predicted | 0.055 | RS-16 | MMP2 | Predicted | 0.009 |
| RAB-03 | MAPK1 | Predicted | 0.011 | RS-16 | MMP3 | Predicted | 0.033 |
| RAB-03 | MME | Predicted | 0.132 | RS-16 | MMP7 | Predicted | 0.009 |
| RAB-03 | MTHFD1 | Predicted | 0.012 | RS-16 | MMP9 | Predicted | 0.009 |
| RAB-03 | MTR | Predicted | 0.012 | RS-16 | NME1 | Predicted | 0.018 |
| RAB-03 | PDGFRA | Predicted | 0.016 | RS-16 | NOS2A | Predicted | 0.033 |
| RAB-03 | PTGS2 | Predicted | 0.031 | RS-16 | PGK1 | Predicted | 0.003 |

| RAB-03 | RAF1 | Predicted | 0.016 | RS-16 | PTGS2 | Predicted | 0.038 |
| --- | --- | --- | --- | --- | --- | --- | --- |
| RAB-03 | RARA | Predicted | 0.018 | RS-16 | PTK2 | Predicted | 0.003 |
| RAB-03 | RND3 | Predicted | 0.015 | RS-16 | RND3 | Predicted | 0.010 |
| RAB-03 | SERPINE1 | Predicted | 0.026 | RS-16 | SERPINE1 | Predicted | 0.040 |
| RAB-03 | TUBB | Predicted | 0.007 | RS-16 | SRC | Predicted | 0.181 |
| RAB-04 | ABCB1 | Predicted | 0.006 | RS-16 | SULT2A1 | Predicted | 0.009 |
| RAB-04 | ACSL1 | Predicted | 0.006 | RS-16 | TOP2A | Predicted | 0.074 |
| RAB-04 | AKT2 | Predicted | 0.046 | RS-16 | TRDMT1 | Predicted | 0.002 |
| RAB-04 | ASS1 | Predicted | 0.006 | RS-17 | ABCB1 | Predicted | 0.015 |
| RAB-04 | CCT3 | Predicted | 0.003 | RS-17 | ACSL1 | Predicted | 0.015 |
| RAB-04 | CES1 | Predicted | 0.045 | RS-17 | ADH1B | Predicted | 0.004 |
| RAB-04 | ESR1 | Predicted | 0.484 | RS-17 | ADH1C | Predicted | 0.004 |
| RAB-04 | FGF1 | Predicted | 0.073 | RS-17 | ADH4 | Predicted | 0.004 |
| RAB-04 | FGF2 | Predicted | 0.073 | RS-17 | AKT2 | Predicted | 0.128 |
| RAB-04 | GAPDH | Predicted | 0.020 | RS-17 | ALDH1A1 | Predicted | 0.004 |
| RAB-04 | GLUD1 | Predicted | 0.000 | RS-17 | ALDH1L1 | Predicted | 0.014 |
| RAB-04 | HAO1 | Predicted | 0.000 | RS-17 | ALDH2 | Predicted | 0.004 |
| RAB-04 | KRAS | Predicted | 0.032 | RS-17 | ASS1 | Predicted | 0.015 |
| RAB-04 | LYZ | Predicted | 0.076 | RS-17 | CCNA2 | Predicted | 0.688 |
| RAB-04 | MME | Predicted | 0.102 | RS-17 | CCT3 | Predicted | 0.009 |
| RAB-04 | MMP12 | Predicted | 0.062 | RS-17 | CDK4 | Predicted | 0.044 |
| RAB-04 | MMP14 | Predicted | 0.015 | RS-17 | CYP2C8 | Predicted | 0.011 |
| RAB-04 | MMP2 | Predicted | 0.015 | RS-17 | EGFR | Predicted | 0.142 |
| RAB-04 | MMP3 | Predicted | 0.015 | RS-17 | ESR1 | Predicted | 0.246 |
| RAB-04 | MMP7 | Predicted | 0.015 | RS-17 | FTCD | Predicted | 0.014 |

| RAB-04 | MMP9 | Predicted | 0.015 | RS-17 | GAPDH | Predicted | 0.019 |
| --- | --- | --- | --- | --- | --- | --- | --- |
| RAB-04 | NME1 | Predicted | 0.161 | RS-17 | GLUD1 | Predicted | 0.022 |
| RAB-04 | NOS2A | Predicted | 0.027 | RS-17 | GNMT | Predicted | 0.005 |
| RAB-04 | PLK1 | Predicted | 0.073 | RS-17 | GSTP1 | Predicted | 0.112 |
| RAB-04 | RND3 | Predicted | 0.000 | RS-17 | HSD17B10 | Predicted | 0.004 |
| RAB-04 | SGK1 | Predicted | 0.000 | RS-17 | HSD17B4 | Predicted | 0.004 |
| RAB-04 | TOP2A | Predicted | 0.074 | RS-17 | KRAS | Predicted | 0.066 |
| RAB-04 | VEGFA | Predicted | 0.026 | RS-17 | MAPK1 | Predicted | 0.121 |
| RAB-05 | ABCB1 | Predicted | 0.010 | RS-17 | MAPK3 | Predicted | 0.044 |
| RAB-05 | ACSL1 | Predicted | 0.035 | RS-17 | MME | Predicted | 0.330 |
| RAB-05 | AGXT | Predicted | 0.005 | RS-17 | MTHFD1 | Predicted | 0.014 |
| RAB-05 | AKT2 | Predicted | 0.045 | RS-17 | MTR | Predicted | 0.014 |
| RAB-05 | AR | Predicted | 0.072 | RS-17 | NME1 | Predicted | 0.134 |
| RAB-05 | ASS1 | Predicted | 0.010 | RS-17 | NOS2A | Predicted | 0.133 |
| RAB-05 | AURKA | Predicted | 0.019 | RS-17 | QDPR | Predicted | 0.004 |
| RAB-05 | CA1 | Predicted | 0.120 | RS-17 | RAF1 | Predicted | 0.016 |
| RAB-05 | CA2 | Predicted | 0.120 | RS-17 | RND3 | Predicted | 0.013 |
| RAB-05 | CCNA2 | Predicted | 0.828 | RS-17 | SERPINE1 | Predicted | 0.041 |
| RAB-05 | CCT3 | Predicted | 0.020 | RS-17 | TRDMT1 | Predicted | 0.005 |
| RAB-05 | CES1 | Predicted | 0.039 | RS-17 | TUBB | Predicted | 0.020 |
| RAB-05 | CTSB | Predicted | 0.130 | RS-17 | VEGFA | Predicted | 0.029 |
| RAB-05 | ESR1 | Predicted | 0.318 | RS-18 | ADH1B | Predicted | 0.010 |
| RAB-05 | FGF1 | Predicted | 0.062 | RS-18 | ADH1C | Predicted | 0.001 |
| RAB-05 | FGF2 | Predicted | 0.062 | RS-18 | ADH4 | Predicted | 0.001 |
| RAB-05 | GABRE | Predicted | 0.037 | RS-18 | AKR1B10 | Predicted | 0.000 |

| RAB-05 | GAPDH | Predicted | 0.008 | RS-18 | AKR1C2 | Predicted | 0.000 |
| --- | --- | --- | --- | --- | --- | --- | --- |
| RAB-05 | GLUD1 | Predicted | 0.019 | RS-18 | ALDH1A1 | Predicted | 0.001 |
| RAB-05 | GNAI1 | Predicted | 0.002 | RS-18 | ALDH1L1 | Predicted | 0.004 |
| RAB-05 | GNMT | Predicted | 0.001 | RS-18 | ALDH2 | Predicted | 0.001 |
| RAB-05 | HSP90AA1 | Predicted | 0.005 | RS-18 | DCXR | Predicted | 0.000 |
| RAB-05 | HSP90B1 | Predicted | 0.005 | RS-18 | DDR1 | Predicted | 0.049 |
| RAB-05 | HSPA1B | Predicted | 0.005 | RS-18 | EGFR | Predicted | 0.246 |
| RAB-05 | HSPA8 | Predicted | 0.005 | RS-18 | ESR1 | Predicted | 0.248 |
| RAB-05 | KRAS | Predicted | 0.078 | RS-18 | FTCD | Predicted | 0.004 |
| RAB-05 | LYZ | Predicted | 0.062 | RS-18 | GAPDH | Predicted | 0.001 |
| RAB-05 | MAPK1 | Predicted | 0.056 | RS-18 | GLUD1 | Predicted | 0.001 |
| RAB-05 | MAT1A | Predicted | 0.005 | RS-18 | HSD17B10 | Predicted | 0.001 |
| RAB-05 | MME | Predicted | 0.085 | RS-18 | HSD17B4 | Predicted | 0.001 |
| RAB-05 | MMP12 | Predicted | 0.039 | RS-18 | MME | Predicted | 0.255 |
| RAB-05 | MMP14 | Predicted | 0.011 | RS-18 | MTHFD1 | Predicted | 0.013 |
| RAB-05 | MMP2 | Predicted | 0.011 | RS-18 | MTR | Predicted | 0.004 |
| RAB-05 | MMP3 | Predicted | 0.034 | RS-18 | PDGFRA | Predicted | 0.049 |
| RAB-05 | MMP7 | Predicted | 0.011 | RS-18 | QDPR | Predicted | 0.001 |
| RAB-05 | MMP9 | Predicted | 0.011 | RS-18 | RAF1 | Predicted | 0.033 |
| RAB-05 | MTAP | Predicted | 0.132 | RS-19 | ABCB1 | Predicted | 0.008 |
| RAB-05 | NME1 | Predicted | 0.030 | RS-19 | ACSL1 | Predicted | 0.008 |
| RAB-05 | NME2 | Predicted | 0.002 | RS-19 | ADH1B | Predicted | 0.000 |
| RAB-05 | PGK1 | Predicted | 0.005 | RS-19 | ADH1C | Predicted | 0.000 |
| RAB-05 | PTGS2 | Predicted | 0.095 | RS-19 | ADH4 | Predicted | 0.000 |
| RAB-05 | PTK2 | Predicted | 0.005 | RS-19 | AGXT | Predicted | 0.005 |

| RAB-05 | RHOA | Predicted | 0.002 | RS-19 | AKT2 | Predicted | 0.113 |
| --- | --- | --- | --- | --- | --- | --- | --- |
| RAB-05 | RND3 | Predicted | 0.004 | RS-19 | ALDH1A1 | Predicted | 0.000 |
| RAB-05 | SERPINE1 | Predicted | 0.037 | RS-19 | ALDH2 | Predicted | 0.000 |
| RAB-05 | SRC | Predicted | 0.175 | RS-19 | ASS1 | Predicted | 0.008 |
| RAB-05 | TGM2 | Predicted | 0.002 | RS-19 | CA1 | Predicted | 0.038 |
| RAB-05 | TOP2A | Predicted | 0.018 | RS-19 | CA2 | Predicted | 0.038 |
| RAB-05 | TRDMT1 | Predicted | 0.001 | RS-19 | CCNA2 | Predicted | 0.708 |
| RAB-06 | ABCB1 | Predicted | 0.006 | RS-19 | CCT3 | Predicted | 0.007 |
| RAB-06 | ACSL1 | Predicted | 0.006 | RS-19 | CDK4 | Predicted | 0.027 |
| RAB-06 | AKT2 | Predicted | 0.084 | RS-19 | ESR1 | Predicted | 0.282 |
| RAB-06 | ALDH1A1 | Predicted | 0.003 | RS-19 | FGF1 | Predicted | 0.061 |
| RAB-06 | ALDH1L1 | Predicted | 0.002 | RS-19 | FGF2 | Predicted | 0.061 |
| RAB-06 | ASS1 | Predicted | 0.006 | RS-19 | GAPDH | Predicted | 0.018 |
| RAB-06 | CA1 | Predicted | 0.016 | RS-19 | GLUD1 | Predicted | 0.009 |
| RAB-06 | CA2 | Predicted | 0.016 | RS-19 | GNAI1 | Predicted | 0.000 |
| RAB-06 | CCNA2 | Predicted | 1.000 | RS-19 | GSTP1 | Predicted | 0.134 |
| RAB-06 | CCT3 | Predicted | 0.006 | RS-19 | HAO1 | Predicted | 0.002 |
| RAB-06 | CYP2C8 | Predicted | 0.014 | RS-19 | HSD17B10 | Predicted | 0.000 |
| RAB-06 | ESR1 | Predicted | 0.098 | RS-19 | HSD17B4 | Predicted | 0.000 |
| RAB-06 | FTCD | Predicted | 0.002 | RS-19 | KRAS | Predicted | 0.091 |
| RAB-06 | GLUD1 | Predicted | 0.014 | RS-19 | LYZ | Predicted | 0.064 |
| RAB-06 | GNMT | Predicted | 0.003 | RS-19 | MAPK1 | Predicted | 0.090 |
| RAB-06 | GSTP1 | Predicted | 0.030 | RS-19 | MAPK3 | Predicted | 0.027 |
| RAB-06 | KRAS | Predicted | 0.025 | RS-19 | MET | Predicted | 0.143 |
| RAB-06 | MAPK1 | Predicted | 0.046 | RS-19 | MME | Predicted | 0.087 |

| RAB-06 | MME | Predicted | 0.146 | RS-19 | MMP12 | Predicted | 0.039 |
| --- | --- | --- | --- | --- | --- | --- | --- |
| RAB-06 | MMP12 | Predicted | 0.039 | RS-19 | NME1 | Predicted | 0.009 |
| RAB-06 | MTHFD1 | Predicted | 0.002 | RS-19 | NME2 | Predicted | 0.000 |
| RAB-06 | MTR | Predicted | 0.002 | RS-19 | QDPR | Predicted | 0.000 |
| RAB-06 | NME1 | Predicted | 0.098 | RS-19 | RHOA | Predicted | 0.000 |
| RAB-06 | PLAU | Predicted | 0.076 | RS-19 | RND3 | Predicted | 0.000 |
| RAB-06 | PTGS2 | Predicted | 0.047 | RS-19 | SERPINE1 | Predicted | 0.020 |
| RAB-06 | RBP1 | Predicted | 0.003 | RS-19 | SGK1 | Predicted | 0.002 |
| RAB-06 | RND3 | Predicted | 0.014 | RS-19 | SRC | Predicted | 0.064 |
| RAB-06 | SERPINE1 | Predicted | 0.023 | RS-19 | TGM2 | Predicted | 0.000 |
| RAB-06 | SRC | Predicted | 0.099 | RS-19 | TOP2A | Predicted | 0.086 |
| RAB-06 | TRDMT1 | Predicted | 0.003 | RS-19 | VEGFA | Predicted | 0.020 |
| RAB-06 | VEGFA | Predicted | 0.024 | RS-20 | EGFR | Predicted | 0.994 |
| RAB-07 | ABCB1 | Predicted | 0.006 | RS-21 | ACSL1 | Predicted | 0.026 |
| RAB-07 | ACSL1 | Predicted | 0.006 | RS-21 | AGXT | Predicted | 0.006 |
| RAB-07 | AKT2 | Predicted | 0.084 | RS-21 | AKR1B10 | Predicted | 0.090 |
| RAB-07 | ALDH1A1 | Predicted | 0.003 | RS-21 | AKT2 | Predicted | 0.066 |
| RAB-07 | ALDH1L1 | Predicted | 0.002 | RS-21 | AURKA | Predicted | 0.004 |
| RAB-07 | ASS1 | Predicted | 0.006 | RS-21 | CA1 | Predicted | 0.066 |
| RAB-07 | CA1 | Predicted | 0.016 | RS-21 | CA2 | Predicted | 0.066 |
| RAB-07 | CA2 | Predicted | 0.016 | RS-21 | CCNA2 | Predicted | 1.000 |
| RAB-07 | CCNA2 | Predicted | 1.000 | RS-21 | CCT3 | Predicted | 0.004 |
| RAB-07 | CCT3 | Predicted | 0.006 | RS-21 | CTSB | Predicted | 0.088 |
| RAB-07 | CYP2C8 | Predicted | 0.014 | RS-21 | ESR1 | Predicted | 0.088 |
| RAB-07 | ESR1 | Predicted | 0.098 | RS-21 | FGF1 | Predicted | 0.041 |

| RAB-07 | FTCD | Predicted | 0.002 | RS-21 | FGF2 | Predicted | 0.041 |
| --- | --- | --- | --- | --- | --- | --- | --- |
| RAB-07 | GLUD1 | Predicted | 0.014 | RS-21 | GABRE | Predicted | 0.006 |
| RAB-07 | GNMT | Predicted | 0.003 | RS-21 | GLUD1 | Predicted | 0.014 |
| RAB-07 | GSTP1 | Predicted | 0.030 | RS-21 | GNAI1 | Predicted | 0.005 |
| RAB-07 | KRAS | Predicted | 0.025 | RS-21 | GNMT | Predicted | 0.003 |
| RAB-07 | MAPK1 | Predicted | 0.046 | RS-21 | GSTP1 | Predicted | 0.007 |
| RAB-07 | MME | Predicted | 0.146 | RS-21 | HAO1 | Predicted | 0.000 |
| RAB-07 | MMP12 | Predicted | 0.039 | RS-21 | HSP90AA1 | Predicted | 0.004 |
| RAB-07 | MTHFD1 | Predicted | 0.002 | RS-21 | HSP90B1 | Predicted | 0.004 |
| RAB-07 | MTR | Predicted | 0.002 | RS-21 | HSPA1B | Predicted | 0.004 |
| RAB-07 | NME1 | Predicted | 0.098 | RS-21 | HSPA8 | Predicted | 0.004 |
| RAB-07 | PLAU | Predicted | 0.076 | RS-21 | KRAS | Predicted | 0.018 |
| RAB-07 | PTGS2 | Predicted | 0.047 | RS-21 | LYZ | Predicted | 0.086 |
| RAB-07 | RBP1 | Predicted | 0.003 | RS-21 | MAPK1 | Predicted | 0.116 |
| RAB-07 | RND3 | Predicted | 0.014 | RS-21 | MAPK3 | Predicted | 0.024 |
| RAB-07 | SERPINE1 | Predicted | 0.023 | RS-21 | MAT1A | Predicted | 0.004 |
| RAB-07 | SRC | Predicted | 0.099 | RS-21 | MME | Predicted | 0.056 |
| RAB-07 | TRDMT1 | Predicted | 0.003 | RS-21 | MTAP | Predicted | 0.176 |
| RAB-07 | VEGFA | Predicted | 0.024 | RS-21 | NME1 | Predicted | 0.021 |
| RAB-08 | ABCB1 | Predicted | 0.009 | RS-21 | NME2 | Predicted | 0.005 |
| RAB-08 | ACSL1 | Predicted | 0.009 | RS-21 | NOS2A | Predicted | 0.087 |
| RAB-08 | ADH1C | Predicted | 0.005 | RS-21 | PGK1 | Predicted | 0.004 |
| RAB-08 | AKT2 | Predicted | 0.052 | RS-21 | PTGS2 | Predicted | 0.104 |
| RAB-08 | ASS1 | Predicted | 0.009 | RS-21 | PTK2 | Predicted | 0.004 |
| RAB-08 | AURKA | Predicted | 0.004 | RS-21 | RHOA | Predicted | 0.005 |

| RAB-08 | CA1 | Predicted | 0.020 | RS-21 | RND3 | Predicted | 0.006 |
| --- | --- | --- | --- | --- | --- | --- | --- |
| RAB-08 | CA2 | Predicted | 0.020 | RS-21 | SERPINE1 | Predicted | 0.025 |
| RAB-08 | CCNA2 | Predicted | 1.000 | RS-21 | SGK1 | Predicted | 0.000 |
| RAB-08 | CCT3 | Predicted | 0.014 | RS-21 | SRC | Predicted | 0.117 |
| RAB-08 | CES1 | Predicted | 0.005 | RS-21 | SULT2A1 | Predicted | 0.007 |
| RAB-08 | ESR1 | Predicted | 0.124 | RS-21 | TGM2 | Predicted | 0.005 |
| RAB-08 | GLUD1 | Predicted | 0.017 | RS-21 | TOP2A | Predicted | 0.051 |
| RAB-08 | GNAI1 | Predicted | 0.005 | RS-21 | TRDMT1 | Predicted | 0.003 |
| RAB-08 | GNMT | Predicted | 0.004 | RS-22 | AURKA | Predicted | 0.472 |
| RAB-08 | GSTP1 | Predicted | 0.104 | RS-22 | DDR1 | Predicted | 0.048 |
| RAB-08 | HAO1 | Predicted | 0.000 | RS-22 | EGFR | Predicted | 0.477 |
| RAB-08 | HSP90AA1 | Predicted | 0.004 | RS-22 | MME | Predicted | 0.505 |
| RAB-08 | HSP90B1 | Predicted | 0.004 | RS-22 | PDGFRA | Predicted | 0.048 |
| RAB-08 | HSPA1B | Predicted | 0.004 | RS-22 | RAF1 | Predicted | 0.044 |
| RAB-08 | HSPA8 | Predicted | 0.004 | RS-22 | SRC | Predicted | 0.047 |
| RAB-08 | KRAS | Predicted | 0.028 | RS-24 | ACSL1 | Predicted | 0.026 |
| RAB-08 | MAPK1 | Predicted | 0.121 | RS-24 | AGXT | Predicted | 0.007 |
| RAB-08 | MAT1A | Predicted | 0.004 | RS-24 | ALDOA | Predicted | 0.118 |
| RAB-08 | MME | Predicted | 0.186 | RS-24 | AURKA | Predicted | 0.013 |
| RAB-08 | MMP12 | Predicted | 0.048 | RS-24 | CA1 | Predicted | 0.130 |
| RAB-08 | NME1 | Predicted | 0.024 | RS-24 | CA2 | Predicted | 0.130 |
| RAB-08 | NME2 | Predicted | 0.005 | RS-24 | CCNA2 | Predicted | 1.000 |
| RAB-08 | PGK1 | Predicted | 0.004 | RS-24 | CCT3 | Predicted | 0.004 |
| RAB-08 | PLAU | Predicted | 0.086 | RS-24 | CES1 | Predicted | 0.036 |
| RAB-08 | PTGS2 | Predicted | 0.026 | RS-24 | CTSB | Predicted | 0.124 |

| RAB-08 | PTK2 | Predicted | 0.004 | RS-24 | FGF1 | Predicted | 0.057 |
| --- | --- | --- | --- | --- | --- | --- | --- |
| RAB-08 | RHOA | Predicted | 0.005 | RS-24 | FGF2 | Predicted | 0.057 |
| RAB-08 | RND3 | Predicted | 0.008 | RS-24 | GABRE | Predicted | 0.033 |
| RAB-08 | SERPINE1 | Predicted | 0.017 | RS-24 | GLUD1 | Predicted | 0.016 |
| RAB-08 | SGK1 | Predicted | 0.000 | RS-24 | GNAI1 | Predicted | 0.005 |
| RAB-08 | SRC | Predicted | 0.119 | RS-24 | GNMT | Predicted | 0.009 |
| RAB-08 | TGM2 | Predicted | 0.005 | RS-24 | GSTP1 | Predicted | 0.004 |
| RAB-08 | TRDMT1 | Predicted | 0.004 | RS-24 | HSP90AA1 | Predicted | 0.004 |
| RAB-08 | VEGFA | Predicted | 0.037 | RS-24 | HSP90B1 | Predicted | 0.004 |
| RAB-09 | ABCB1 | Predicted | 0.015 | RS-24 | HSPA1B | Predicted | 0.004 |
| RAB-09 | ACSL1 | Predicted | 0.015 | RS-24 | HSPA8 | Predicted | 0.004 |
| RAB-09 | AKT2 | Predicted | 0.053 | RS-24 | KRAS | Predicted | 0.027 |
| RAB-09 | ALDH1L1 | Predicted | 0.013 | RS-24 | MAPK1 | Predicted | 0.162 |
| RAB-09 | ASS1 | Predicted | 0.015 | RS-24 | MAPK3 | Predicted | 0.066 |
| RAB-09 | AURKA | Predicted | 0.134 | RS-24 | MAT1A | Predicted | 0.004 |
| RAB-09 | CCNA2 | Predicted | 0.408 | RS-24 | MMP12 | Predicted | 0.119 |
| RAB-09 | CCT3 | Predicted | 0.008 | RS-24 | MMP14 | Predicted | 0.000 |
| RAB-09 | CYP2C8 | Predicted | 0.011 | RS-24 | MMP2 | Predicted | 0.000 |
| RAB-09 | DDR1 | Predicted | 0.016 | RS-24 | MMP3 | Predicted | 0.000 |
| RAB-09 | EGFR | Predicted | 0.138 | RS-24 | MMP7 | Predicted | 0.000 |
| RAB-09 | ESR1 | Predicted | 0.134 | RS-24 | MMP9 | Predicted | 0.000 |
| RAB-09 | FTCD | Predicted | 0.013 | RS-24 | MTAP | Predicted | 0.248 |
| RAB-09 | GLUD1 | Predicted | 0.015 | RS-24 | NME1 | Predicted | 0.024 |
| RAB-09 | GSTP1 | Predicted | 0.023 | RS-24 | NME2 | Predicted | 0.005 |
| RAB-09 | KRAS | Predicted | 0.061 | RS-24 | NOS2A | Predicted | 0.121 |

| RAB-09 | MME | Predicted | 0.139 | RS-24 | PGK1 | Predicted | 0.004 |
| --- | --- | --- | --- | --- | --- | --- | --- |
| RAB-09 | MTHFD1 | Predicted | 0.013 | RS-24 | PTGS2 | Predicted | 0.210 |
| RAB-09 | MTR | Predicted | 0.013 | RS-24 | PTK2 | Predicted | 0.004 |
| RAB-09 | NME1 | Predicted | 0.131 | RS-24 | RHOA | Predicted | 0.005 |
| RAB-09 | PDGFRA | Predicted | 0.016 | RS-24 | RND3 | Predicted | 0.006 |
| RAB-09 | RAF1 | Predicted | 0.016 | RS-24 | SRC | Predicted | 0.119 |
| RAB-09 | RARA | Predicted | 0.018 | RS-24 | SULT2A1 | Predicted | 0.010 |
| RAB-09 | RND3 | Predicted | 0.015 | RS-24 | TGM2 | Predicted | 0.005 |
| RAB-09 | SERPINE1 | Predicted | 0.022 | RS-24 | TOP2A | Predicted | 0.026 |
| RAB-09 | TUBB | Predicted | 0.007 | RS-24 | TRDMT1 | Predicted | 0.009 |
| RAB-09 | VEGFA | Predicted | 0.031 | RS-25 | ABCB1 | Predicted | 0.005 |
| RAB-10 | ABCB1 | Predicted | 0.011 | RS-25 | ACSL1 | Predicted | 0.005 |
| RAB-10 | ACSL1 | Predicted | 0.011 | RS-25 | AGXT | Predicted | 0.011 |
| RAB-10 | AKT2 | Predicted | 0.042 | RS-25 | ALDH2 | Predicted | 0.122 |
| RAB-10 | ALDH1L1 | Predicted | 0.012 | RS-25 | ALDOA | Predicted | 0.116 |
| RAB-10 | ASS1 | Predicted | 0.011 | RS-25 | ASS1 | Predicted | 0.005 |
| RAB-10 | AURKA | Predicted | 0.105 | RS-25 | AURKA | Predicted | 0.002 |
| RAB-10 | CA1 | Predicted | 0.018 | RS-25 | CA1 | Predicted | 0.017 |
| RAB-10 | CA2 | Predicted | 0.018 | RS-25 | CA2 | Predicted | 0.055 |
| RAB-10 | CCNA2 | Predicted | 1.000 | RS-25 | CCT3 | Predicted | 0.011 |
| RAB-10 | CCT3 | Predicted | 0.005 | RS-25 | CDK4 | Predicted | 0.015 |
| RAB-10 | CYP2C8 | Predicted | 0.014 | RS-25 | CES1 | Predicted | 0.012 |
| RAB-10 | EGFR | Predicted | 0.105 | RS-25 | CTSB | Predicted | 0.121 |
| RAB-10 | ESR1 | Predicted | 0.195 | RS-25 | EGFR | Predicted | 0.015 |
| RAB-10 | FTCD | Predicted | 0.012 | RS-25 | ESR1 | Predicted | 0.492 |

| RAB-10 | GLUD1 | Predicted | 0.010 | RS-25 | GABRE | Predicted | 0.146 |
| --- | --- | --- | --- | --- | --- | --- | --- |
| RAB-10 | GNMT | Predicted | 0.004 | RS-25 | GLUD1 | Predicted | 0.010 |
| RAB-10 | GSTP1 | Predicted | 0.092 | RS-25 | GNAI1 | Predicted | 0.000 |
| RAB-10 | KRAS | Predicted | 0.051 | RS-25 | GNMT | Predicted | 0.005 |
| RAB-10 | MAPK1 | Predicted | 0.009 | RS-25 | GSTP1 | Predicted | 0.033 |
| RAB-10 | MME | Predicted | 0.109 | RS-25 | HSP90AA1 | Predicted | 0.002 |
| RAB-10 | MTHFD1 | Predicted | 0.012 | RS-25 | HSP90B1 | Predicted | 0.002 |
| RAB-10 | MTR | Predicted | 0.012 | RS-25 | HSPA1B | Predicted | 0.002 |
| RAB-10 | NME1 | Predicted | 0.109 | RS-25 | HSPA8 | Predicted | 0.002 |
| RAB-10 | NOS2A | Predicted | 0.104 | RS-25 | KRAS | Predicted | 0.043 |
| RAB-10 | PTGS2 | Predicted | 0.045 | RS-25 | LYZ | Predicted | 0.008 |
| RAB-10 | RAF1 | Predicted | 0.013 | RS-25 | MAT1A | Predicted | 0.002 |
| RAB-10 | RARA | Predicted | 0.045 | RS-25 | MMP14 | Predicted | 0.003 |
| RAB-10 | RND3 | Predicted | 0.010 | RS-25 | MMP2 | Predicted | 0.003 |
| RAB-10 | SERPINE1 | Predicted | 0.022 | RS-25 | MMP3 | Predicted | 0.003 |
| RAB-10 | TRDMT1 | Predicted | 0.004 | RS-25 | MMP7 | Predicted | 0.003 |
| RAB-10 | VEGFA | Predicted | 0.027 | RS-25 | MMP9 | Predicted | 0.003 |
| RAB-11 | ABCB1 | Predicted | 0.011 | RS-25 | NME1 | Predicted | 0.019 |
| RAB-11 | ACSL1 | Predicted | 0.011 | RS-25 | NME2 | Predicted | 0.000 |
| RAB-11 | AKT2 | Predicted | 0.042 | RS-25 | NOS2A | Validated | 1.000 |
| RAB-11 | ALDH1L1 | Predicted | 0.012 | RS-25 | PGK1 | Predicted | 0.002 |
| RAB-11 | ASS1 | Predicted | 0.011 | RS-25 | PTGS2 | Predicted | 0.173 |
| RAB-11 | AURKA | Predicted | 0.105 | RS-25 | PTK2 | Predicted | 0.002 |
| RAB-11 | CA1 | Predicted | 0.018 | RS-25 | REG1A | Predicted | 0.012 |
| RAB-11 | CA2 | Predicted | 0.018 | RS-25 | RHOA | Predicted | 0.000 |

| RAB-11 | CCNA2 | Predicted | 1.000 | RS-25 | RND3 | Predicted | 0.000 |
| --- | --- | --- | --- | --- | --- | --- | --- |
| RAB-11 | CCT3 | Predicted | 0.005 | RS-25 | SOD1 | Validated | 1.000 |
| RAB-11 | CYP2C8 | Predicted | 0.014 | RS-25 | TGM2 | Predicted | 0.000 |
| RAB-11 | EGFR | Predicted | 0.105 | RS-25 | TRDMT1 | Predicted | 0.005 |
| RAB-11 | ESR1 | Predicted | 0.195 | RS-26 | ACSL1 | Predicted | 0.017 |
| RAB-11 | FTCD | Predicted | 0.012 | RS-26 | AGXT | Predicted | 0.003 |
| RAB-11 | GLUD1 | Predicted | 0.010 | RS-26 | AKT2 | Predicted | 0.092 |
| RAB-11 | GNMT | Predicted | 0.004 | RS-26 | AURKA | Predicted | 0.011 |
| RAB-11 | GSTP1 | Predicted | 0.092 | RS-26 | CA1 | Predicted | 0.073 |
| RAB-11 | KRAS | Predicted | 0.051 | RS-26 | CA2 | Predicted | 0.073 |
| RAB-11 | MAPK1 | Predicted | 0.009 | RS-26 | CCNA2 | Predicted | 1.000 |
| RAB-11 | MME | Predicted | 0.109 | RS-26 | CCT3 | Predicted | 0.012 |
| RAB-11 | MTHFD1 | Predicted | 0.012 | RS-26 | CES1 | Predicted | 0.027 |
| RAB-11 | MTR | Predicted | 0.012 | RS-26 | CTSB | Predicted | 0.095 |
| RAB-11 | NME1 | Predicted | 0.109 | RS-26 | FGF1 | Predicted | 0.043 |
| RAB-11 | NOS2A | Predicted | 0.104 | RS-26 | FGF2 | Predicted | 0.043 |
| RAB-11 | PTGS2 | Predicted | 0.045 | RS-26 | GABRE | Predicted | 0.021 |
| RAB-11 | RAF1 | Predicted | 0.013 | RS-26 | GLUD1 | Predicted | 0.011 |
| RAB-11 | RARA | Predicted | 0.045 | RS-26 | GNAI1 | Predicted | 0.001 |
| RAB-11 | RND3 | Predicted | 0.010 | RS-26 | GNMT | Predicted | 0.000 |
| RAB-11 | SERPINE1 | Predicted | 0.022 | RS-26 | GSTP1 | Predicted | 0.003 |
| RAB-11 | TRDMT1 | Predicted | 0.004 | RS-26 | HSP90AA1 | Predicted | 0.002 |
| RAB-11 | VEGFA | Predicted | 0.027 | RS-26 | HSP90B1 | Predicted | 0.002 |
| RAB-12 | ABCB1 | Predicted | 0.014 | RS-26 | HSPA1B | Predicted | 0.002 |
| RAB-12 | ACSL1 | Predicted | 0.014 | RS-26 | HSPA8 | Predicted | 0.002 |

| RAB-12 | AKT2 | Predicted | 0.047 | RS-26 | KRAS | Predicted | 0.026 |
| --- | --- | --- | --- | --- | --- | --- | --- |
| RAB-12 | ALDH1L1 | Predicted | 0.013 | RS-26 | MAPK1 | Predicted | 0.128 |
| RAB-12 | ASS1 | Predicted | 0.014 | RS-26 | MAPK3 | Predicted | 0.029 |
| RAB-12 | AURKA | Predicted | 0.137 | RS-26 | MAT1A | Predicted | 0.002 |
| RAB-12 | CCNA2 | Predicted | 0.267 | RS-26 | MME | Predicted | 0.060 |
| RAB-12 | CCT3 | Predicted | 0.006 | RS-26 | MMP12 | Predicted | 0.093 |
| RAB-12 | CYP2C8 | Predicted | 0.009 | RS-26 | MMP14 | Predicted | 0.001 |
| RAB-12 | ESR1 | Predicted | 0.132 | RS-26 | MMP2 | Predicted | 0.001 |
| RAB-12 | FTCD | Predicted | 0.013 | RS-26 | MMP3 | Predicted | 0.001 |
| RAB-12 | GLUD1 | Predicted | 0.009 | RS-26 | MMP7 | Predicted | 0.001 |
| RAB-12 | GNMT | Predicted | 0.000 | RS-26 | MMP9 | Predicted | 0.001 |
| RAB-12 | GSTP1 | Predicted | 0.024 | RS-26 | NME1 | Predicted | 0.019 |
| RAB-12 | KRAS | Predicted | 0.056 | RS-26 | NME2 | Predicted | 0.001 |
| RAB-12 | MME | Predicted | 0.136 | RS-26 | NOS2A | Predicted | 0.047 |
| RAB-12 | MTHFD1 | Predicted | 0.013 | RS-26 | PGK1 | Predicted | 0.002 |
| RAB-12 | MTR | Predicted | 0.013 | RS-26 | PTGS2 | Predicted | 0.057 |
| RAB-12 | NME1 | Predicted | 0.134 | RS-26 | PTK2 | Predicted | 0.002 |
| RAB-12 | NOS2A | Predicted | 0.131 | RS-26 | RHOA | Predicted | 0.001 |
| RAB-12 | RAF1 | Predicted | 0.018 | RS-26 | RND3 | Predicted | 0.002 |
| RAB-12 | RARA | Predicted | 0.019 | RS-26 | SERPINE1 | Predicted | 0.020 |
| RAB-12 | RND3 | Predicted | 0.009 | RS-26 | SRC | Predicted | 0.094 |
| RAB-12 | SERPINE1 | Predicted | 0.024 | RS-26 | SULT2A1 | Predicted | 0.004 |
| RAB-12 | TRDMT1 | Predicted | 0.000 | RS-26 | TGM2 | Predicted | 0.001 |
| RAB-12 | VEGFA | Predicted | 0.027 | RS-26 | TOP2A | Predicted | 0.042 |
| RAB-13 | ABCB1 | Predicted | 0.009 | RS-26 | TRDMT1 | Predicted | 0.000 |

| RAB-13 | ACSL1 | Predicted | 0.009 | RS-27 | ABCB1 | Predicted | 0.003 |
| --- | --- | --- | --- | --- | --- | --- | --- |
| RAB-13 | AKT2 | Predicted | 0.047 | RS-27 | ACSL1 | Predicted | 0.003 |
| RAB-13 | ALDH1L1 | Predicted | 0.015 | RS-27 | AGXT | Predicted | 0.003 |
| RAB-13 | ASS1 | Predicted | 0.009 | RS-27 | AKR1B10 | Predicted | 0.074 |
| RAB-13 | AURKA | Predicted | 0.117 | RS-27 | AKT2 | Predicted | 0.035 |
| RAB-13 | CA1 | Predicted | 0.020 | RS-27 | ALDH1A1 | Predicted | 0.016 |
| RAB-13 | CA2 | Predicted | 0.020 | RS-27 | ASS1 | Predicted | 0.003 |
| RAB-13 | CCNA2 | Predicted | 0.994 | RS-27 | AURKA | Predicted | 0.002 |
| RAB-13 | CCT3 | Predicted | 0.006 | RS-27 | CA1 | Predicted | 0.034 |
| RAB-13 | CYP2C8 | Predicted | 0.009 | RS-27 | CA2 | Predicted | 0.034 |
| RAB-13 | EGFR | Predicted | 0.121 | RS-27 | CCNA2 | Predicted | 1.000 |
| RAB-13 | ESR1 | Predicted | 0.121 | RS-27 | CCT3 | Predicted | 0.009 |
| RAB-13 | FTCD | Predicted | 0.015 | RS-27 | CDK4 | Predicted | 0.028 |
| RAB-13 | GLUD1 | Predicted | 0.010 | RS-27 | CTSB | Predicted | 0.072 |
| RAB-13 | GNMT | Predicted | 0.005 | RS-27 | ESR1 | Predicted | 0.150 |
| RAB-13 | GSTP1 | Predicted | 0.103 | RS-27 | GLUD1 | Predicted | 0.011 |
| RAB-13 | KRAS | Predicted | 0.049 | RS-27 | GNAI1 | Predicted | 0.003 |
| RAB-13 | MAPK1 | Predicted | 0.010 | RS-27 | GNMT | Predicted | 0.003 |
| RAB-13 | MME | Predicted | 0.121 | RS-27 | GSTP1 | Predicted | 0.021 |
| RAB-13 | MTHFD1 | Predicted | 0.015 | RS-27 | HSP90AA1 | Predicted | 0.002 |
| RAB-13 | MTR | Predicted | 0.015 | RS-27 | HSP90B1 | Predicted | 0.002 |
| RAB-13 | NME1 | Predicted | 0.121 | RS-27 | HSPA1B | Predicted | 0.002 |
| RAB-13 | PTGS2 | Predicted | 0.049 | RS-27 | HSPA8 | Predicted | 0.002 |
| RAB-13 | RAF1 | Predicted | 0.014 | RS-27 | KRAS | Predicted | 0.050 |
| RAB-13 | RARA | Predicted | 0.016 | RS-27 | LYZ | Predicted | 0.072 |

| RAB-13 | RND3 | Predicted | 0.010 | RS-27 | MAPK1 | Predicted | 0.075 |
| --- | --- | --- | --- | --- | --- | --- | --- |
| RAB-13 | SERPINE1 | Predicted | 0.025 | RS-27 | MAPK3 | Predicted | 0.028 |
| RAB-13 | TRDMT1 | Predicted | 0.005 | RS-27 | MAT1A | Predicted | 0.002 |
| RAB-13 | TUBB | Predicted | 0.006 | RS-27 | MME | Predicted | 0.112 |
| RAB-13 | VEGFA | Predicted | 0.025 | RS-27 | NME1 | Predicted | 0.017 |
| RAB-14 | ABCB1 | Predicted | 0.014 | RS-27 | NME2 | Predicted | 0.003 |
| RAB-14 | ACSL1 | Predicted | 0.014 | RS-27 | NOS2A | Predicted | 0.073 |
| RAB-14 | AKT2 | Predicted | 0.020 | RS-27 | PGK1 | Predicted | 0.002 |
| RAB-14 | ALDH1L1 | Predicted | 0.015 | RS-27 | PTGS2 | Predicted | 0.052 |
| RAB-14 | ASS1 | Predicted | 0.014 | RS-27 | PTK2 | Predicted | 0.002 |
| RAB-14 | AURKA | Predicted | 0.133 | RS-27 | RARA | Predicted | 0.016 |
| RAB-14 | CCNA2 | Predicted | 0.825 | RS-27 | RHOA | Predicted | 0.003 |
| RAB-14 | CCT3 | Predicted | 0.005 | RS-27 | RND3 | Predicted | 0.004 |
| RAB-14 | CYP2C8 | Predicted | 0.012 | RS-27 | SERPINE1 | Predicted | 0.014 |
| RAB-14 | ESR1 | Predicted | 0.041 | RS-27 | SRC | Predicted | 0.034 |
| RAB-14 | FTCD | Predicted | 0.015 | RS-27 | TGM2 | Predicted | 0.003 |
| RAB-14 | GLUD1 | Predicted | 0.011 | RS-27 | TOP2A | Predicted | 0.016 |
| RAB-14 | GNMT | Predicted | 0.002 | RS-27 | TRDMT1 | Predicted | 0.003 |
| RAB-14 | GSTP1 | Predicted | 0.023 | RS-28 | ABCB1 | Predicted | 0.002 |
| RAB-14 | KRAS | Predicted | 0.064 | RS-28 | ACSL1 | Predicted | 0.002 |
| RAB-14 | MME | Predicted | 0.136 | RS-28 | AGXT | Predicted | 0.003 |
| RAB-14 | MTHFD1 | Predicted | 0.015 | RS-28 | ASS1 | Predicted | 0.002 |
| RAB-14 | MTR | Predicted | 0.015 | RS-28 | AURKA | Predicted | 0.002 |
| RAB-14 | NME1 | Predicted | 0.134 | RS-28 | CA1 | Predicted | 0.084 |
| RAB-14 | NOS2A | Predicted | 0.130 | RS-28 | CA2 | Predicted | 0.084 |

| RAB-14 | PTGS2 | Predicted | 0.030 | RS-28 | CCNA2 | Predicted | 1.000 |
| --- | --- | --- | --- | --- | --- | --- | --- |
| RAB-14 | RAF1 | Predicted | 0.016 | RS-28 | CCT3 | Predicted | 0.002 |
| RAB-14 | RARA | Predicted | 0.018 | RS-28 | CES1 | Predicted | 0.038 |
| RAB-14 | RND3 | Predicted | 0.011 | RS-28 | CTSB | Predicted | 0.136 |
| RAB-14 | SERPINE1 | Predicted | 0.029 | RS-28 | FGF1 | Predicted | 0.060 |
| RAB-14 | TRDMT1 | Predicted | 0.002 | RS-28 | FGF2 | Predicted | 0.060 |
| RAB-14 | VEGFA | Predicted | 0.030 | RS-28 | GABRE | Predicted | 0.033 |
| RAB-15 | ABCB1 | Predicted | 0.014 | RS-28 | GLUD1 | Predicted | 0.016 |
| RAB-15 | ACSL1 | Predicted | 0.014 | RS-28 | GNAI1 | Predicted | 0.001 |
| RAB-15 | AKT2 | Predicted | 0.020 | RS-28 | GNMT | Predicted | 0.003 |
| RAB-15 | ALDH1L1 | Predicted | 0.015 | RS-28 | GSTP1 | Predicted | 0.001 |
| RAB-15 | ASS1 | Predicted | 0.014 | RS-28 | HSP90AA1 | Predicted | 0.002 |
| RAB-15 | AURKA | Predicted | 0.133 | RS-28 | HSP90B1 | Predicted | 0.002 |
| RAB-15 | CCNA2 | Predicted | 0.825 | RS-28 | HSPA1B | Predicted | 0.002 |
| RAB-15 | CCT3 | Predicted | 0.005 | RS-28 | HSPA8 | Predicted | 0.002 |
| RAB-15 | CYP2C8 | Predicted | 0.012 | RS-28 | KRAS | Predicted | 0.032 |
| RAB-15 | ESR1 | Predicted | 0.041 | RS-28 | MAPK1 | Predicted | 0.061 |
| RAB-15 | FTCD | Predicted | 0.015 | RS-28 | MAPK3 | Predicted | 0.066 |
| RAB-15 | GLUD1 | Predicted | 0.011 | RS-28 | MAT1A | Predicted | 0.002 |
| RAB-15 | GNMT | Predicted | 0.002 | RS-28 | MME | Predicted | 0.084 |
| RAB-15 | GSTP1 | Predicted | 0.023 | RS-28 | MMP12 | Predicted | 0.040 |
| RAB-15 | KRAS | Predicted | 0.064 | RS-28 | MMP14 | Predicted | 0.010 |
| RAB-15 | MME | Predicted | 0.136 | RS-28 | MMP2 | Predicted | 0.010 |
| RAB-15 | MTHFD1 | Predicted | 0.015 | RS-28 | MMP3 | Predicted | 0.035 |
| RAB-15 | MTR | Predicted | 0.015 | RS-28 | MMP7 | Predicted | 0.010 |

| RAB-15 | NME1 | Predicted | 0.134 | RS-28 | MMP9 | Predicted | 0.010 |
| --- | --- | --- | --- | --- | --- | --- | --- |
| RAB-15 | NOS2A | Predicted | 0.130 | RS-28 | MTAP | Predicted | 0.132 |
| RAB-15 | PTGS2 | Predicted | 0.030 | RS-28 | NME1 | Predicted | 0.027 |
| RAB-15 | RAF1 | Predicted | 0.016 | RS-28 | NME2 | Predicted | 0.001 |
| RAB-15 | RARA | Predicted | 0.018 | RS-28 | NOS2A | Predicted | 0.090 |
| RAB-15 | RND3 | Predicted | 0.011 | RS-28 | PGK1 | Predicted | 0.002 |
| RAB-15 | SERPINE1 | Predicted | 0.029 | RS-28 | PTGS2 | Predicted | 0.143 |
| RAB-15 | SOD1 | Validated | 1.000 | RS-28 | PTK2 | Predicted | 0.002 |
| RAB-15 | TRDMT1 | Predicted | 0.002 | RS-28 | RHOA | Predicted | 0.001 |
| RAB-15 | VEGFA | Predicted | 0.030 | RS-28 | RND3 | Predicted | 0.002 |
| RAB-16 | ABCB1 | Predicted | 0.015 | RS-28 | SRC | Predicted | 0.133 |
| RAB-16 | ACSL1 | Predicted | 0.015 | RS-28 | SULT2A1 | Predicted | 0.013 |
| RAB-16 | AKT2 | Predicted | 0.047 | RS-28 | TGM2 | Predicted | 0.001 |
| RAB-16 | ALDH1L1 | Predicted | 0.016 | RS-28 | TOP2A | Predicted | 0.086 |
| RAB-16 | ASS1 | Predicted | 0.015 | RS-28 | TRDMT1 | Predicted | 0.003 |
| RAB-16 | AURKA | Predicted | 0.134 | RS-30 | EGFR | Predicted | 0.983 |
| RAB-16 | CCNA2 | Predicted | 0.819 | RS-32 | ADH1B | Predicted | 0.000 |
| RAB-16 | CCT3 | Predicted | 0.006 | RS-32 | ADH1C | Predicted | 0.000 |
| RAB-16 | CYP2C8 | Predicted | 0.014 | RS-32 | ADH4 | Predicted | 0.000 |
| RAB-16 | EGFR | Predicted | 0.132 | RS-32 | ALDH1A1 | Predicted | 0.000 |
| RAB-16 | ESR1 | Predicted | 0.043 | RS-32 | ALDH2 | Predicted | 0.000 |
| RAB-16 | FTCD | Predicted | 0.016 | RS-32 | AURKA | Predicted | 0.963 |
| RAB-16 | GLUD1 | Predicted | 0.013 | RS-32 | DDR1 | Predicted | 0.150 |
| RAB-16 | GNMT | Predicted | 0.000 | RS-32 | EGFR | Predicted | 0.953 |
| RAB-16 | GSTP1 | Predicted | 0.024 | RS-32 | GAPDH | Predicted | 0.000 |

| RAB-16 | KRAS | Predicted | 0.056 | RS-32 | GLUD1 | Predicted | 0.000 |
| --- | --- | --- | --- | --- | --- | --- | --- |
| RAB-16 | MME | Predicted | 0.138 | RS-32 | HSD17B10 | Predicted | 0.000 |
| RAB-16 | MTHFD1 | Predicted | 0.016 | RS-32 | HSD17B4 | Predicted | 0.000 |
| RAB-16 | MTR | Predicted | 0.016 | RS-32 | PDGFRA | Predicted | 0.150 |
| RAB-16 | NME1 | Predicted | 0.133 | RS-32 | PTGS2 | Predicted | 0.243 |
| RAB-16 | NOS2A | Predicted | 0.131 | RS-32 | QDPR | Predicted | 0.000 |
| RAB-16 | PTGS2 | Predicted | 0.031 | RS-32 | SRC | Predicted | 0.149 |
| RAB-16 | RAF1 | Predicted | 0.018 | RS-33 | ACSL1 | Predicted | 0.026 |
| RAB-16 | RARA | Predicted | 0.019 | RS-33 | ALDH2 | Predicted | 0.090 |
| RAB-16 | RND3 | Predicted | 0.013 | RS-33 | ALDOA | Predicted | 0.087 |
| RAB-16 | SERPINE1 | Predicted | 0.031 | RS-33 | AR | Predicted | 0.087 |
| RAB-16 | TRDMT1 | Predicted | 0.000 | RS-33 | CA1 | Predicted | 0.098 |
| RAB-16 | TUBB | Predicted | 0.008 | RS-33 | CA2 | Predicted | 0.115 |
| RAB-17 | ABCB1 | Predicted | 0.009 | RS-33 | CCNA2 | Predicted | 0.087 |
| RAB-17 | ACSL1 | Predicted | 0.009 | RS-33 | CTSB | Predicted | 0.088 |
| RAB-17 | ADH1C | Predicted | 0.008 | RS-33 | ESR1 | Predicted | 0.197 |
| RAB-17 | AGXT | Predicted | 0.009 | RS-33 | FGF1 | Predicted | 0.014 |
| RAB-17 | AKR1B10 | Predicted | 0.141 | RS-33 | FGF2 | Predicted | 0.014 |
| RAB-17 | AKT2 | Predicted | 0.182 | RS-33 | GABRE | Predicted | 0.052 |
| RAB-17 | ASS1 | Predicted | 0.009 | RS-33 | GSTP1 | Predicted | 0.000 |
| RAB-17 | AURKA | Predicted | 0.007 | RS-33 | HGF | Predicted | 0.014 |
| RAB-17 | CA1 | Predicted | 0.105 | RS-33 | KRAS | Predicted | 0.017 |
| RAB-17 | CA2 | Predicted | 0.105 | RS-33 | LYZ | Predicted | 0.002 |
| RAB-17 | CASP1 | Predicted | 0.021 | RS-33 | MMP14 | Predicted | 0.000 |
| RAB-17 | CCNA2 | Predicted | 1.000 | RS-33 | MMP2 | Predicted | 0.000 |

| RAB-17 | CCT3 | Predicted | 0.007 | RS-33 | MMP3 | Predicted | 0.000 |
| --- | --- | --- | --- | --- | --- | --- | --- |
| RAB-17 | CDK4 | Predicted | 0.047 | RS-33 | MMP7 | Predicted | 0.000 |
| RAB-17 | CES1 | Predicted | 0.008 | RS-33 | MMP9 | Predicted | 0.000 |
| RAB-17 | CTSB | Predicted | 0.142 | RS-33 | MTAP | Predicted | 0.181 |
| RAB-17 | ESR1 | Predicted | 0.298 | RS-33 | NOS2A | Predicted | 0.180 |
| RAB-17 | GABRE | Predicted | 0.002 | RS-33 | PTGS2 | Predicted | 1.000 |
| RAB-17 | GLUD1 | Predicted | 0.025 | RS-33 | SULT2A1 | Predicted | 0.047 |
| RAB-17 | GNAI1 | Predicted | 0.009 | RS-33 | TNFSF11 | Predicted | 0.026 |
| RAB-17 | GNMT | Predicted | 0.004 | RS-34 | ABCB1 | Predicted | 0.009 |
| RAB-17 | GSTP1 | Predicted | 0.042 | RS-34 | ACSL1 | Predicted | 0.039 |
| RAB-17 | HAO1 | Predicted | 0.000 | RS-34 | ADH1C | Predicted | 0.006 |
| RAB-17 | HSP90AA1 | Predicted | 0.007 | RS-34 | AGXT | Predicted | 0.003 |
| RAB-17 | HSP90B1 | Predicted | 0.007 | RS-34 | ASS1 | Predicted | 0.009 |
| RAB-17 | HSPA1B | Predicted | 0.007 | RS-34 | AURKA | Predicted | 0.000 |
| RAB-17 | HSPA8 | Predicted | 0.007 | RS-34 | CA1 | Predicted | 0.077 |
| RAB-17 | KRAS | Predicted | 0.043 | RS-34 | CA2 | Predicted | 0.077 |
| RAB-17 | MAPK1 | Predicted | 0.047 | RS-34 | CCNA2 | Predicted | 1.000 |
| RAB-17 | MAPK3 | Predicted | 0.103 | RS-34 | CCT3 | Predicted | 0.000 |
| RAB-17 | MAT1A | Predicted | 0.007 | RS-34 | CES1 | Predicted | 0.028 |
| RAB-17 | MME | Predicted | 0.093 | RS-34 | CTSB | Predicted | 0.137 |
| RAB-17 | MMP12 | Predicted | 0.043 | RS-34 | ESR1 | Predicted | 0.123 |
| RAB-17 | MMP9 | Predicted | 0.021 | RS-34 | GLUD1 | Predicted | 0.000 |
| RAB-17 | NME1 | Predicted | 0.037 | RS-34 | GNAI1 | Predicted | 0.001 |
| RAB-17 | NME2 | Predicted | 0.009 | RS-34 | GNMT | Predicted | 0.005 |
| RAB-17 | NOS2A | Predicted | 0.138 | RS-34 | GSTP1 | Predicted | 0.003 |

| RAB-17 | TRDMT1 | Predicted | 0.004 | RS-34 | HSP90AA1 | Predicted | 0.000 |
| --- | --- | --- | --- | --- | --- | --- | --- |
| RAB-17 | VEGFA | Predicted | 0.021 | RS-34 | HSP90B1 | Predicted | 0.000 |
| RAB-18 | ABCG2 | Validated | 1.000 | RS-34 | HSPA1B | Predicted | 0.000 |
| RAB-18 | AGXT | Predicted | 0.006 | RS-34 | HSPA8 | Predicted | 0.000 |
| RAB-18 | AKR1B10 | Predicted | 0.084 | RS-34 | KRAS | Predicted | 0.052 |
| RAB-18 | AKT2 | Predicted | 0.047 | RS-34 | MAPK3 | Predicted | 0.050 |
| RAB-18 | AURKA | Predicted | 0.004 | RS-34 | MAT1A | Predicted | 0.000 |
| RAB-18 | CA1 | Predicted | 0.039 | RS-34 | MMP14 | Predicted | 0.001 |
| RAB-18 | CA2 | Predicted | 0.039 | RS-34 | MMP2 | Predicted | 0.001 |
| RAB-18 | CASP3 | Validated | 1.000 | RS-34 | MMP3 | Predicted | 0.001 |
| RAB-18 | CCNA2 | Predicted | 1.000 | RS-34 | MMP7 | Predicted | 0.001 |
| RAB-18 | CCT3 | Predicted | 0.004 | RS-34 | MMP9 | Predicted | 0.001 |
| RAB-18 | CDK4 | Validated | 1.000 | RS-34 | MTAP | Predicted | 0.285 |
| RAB-18 | CTSB | Predicted | 0.082 | RS-34 | NME1 | Predicted | 0.013 |
| RAB-18 | CXCL12 | Validated | 1.000 | RS-34 | NME2 | Predicted | 0.001 |
| RAB-18 | ESR1 | Predicted | 0.161 | RS-34 | NOS2A | Predicted | 0.094 |
| RAB-18 | GABRE | Predicted | 0.003 | RS-34 | PGK1 | Predicted | 0.000 |
| RAB-18 | GLUD1 | Predicted | 0.013 | RS-34 | PTGS2 | Predicted | 0.271 |
| RAB-18 | GNAI1 | Predicted | 0.005 | RS-34 | PTK2 | Predicted | 0.000 |
| RAB-18 | GNMT | Predicted | 0.006 | RS-34 | RHOA | Predicted | 0.001 |
| RAB-18 | GSTP1 | Predicted | 0.007 | RS-34 | SRC | Predicted | 0.133 |
| RAB-17 | TRDMT1 | Predicted | 0.004 | RS-34 | SULT2A1 | Predicted | 0.005 |
| RAB-17 | VEGFA | Predicted | 0.021 | RS-34 | TGM2 | Predicted | 0.001 |
| RAB-18 | ABCG2 | Validated | 1.000 | RS-34 | TOP2A | Predicted | 0.023 |
| RAB-18 | AGXT | Predicted | 0.006 | RS-34 | TRDMT1 | Predicted | 0.005 |

| RAB-18 | AKR1B10 | Predicted | 0.084 | RS-36 | ACSL1 | Predicted | 0.046 |
| --- | --- | --- | --- | --- | --- | --- | --- |
| RAB-18 | AKT2 | Predicted | 0.047 | RS-36 | AGXT | Predicted | 0.008 |
| RAB-18 | AURKA | Predicted | 0.004 | RS-36 | AURKA | Predicted | 0.016 |
| RAB-18 | CA1 | Predicted | 0.039 | RS-36 | CA1 | Predicted | 0.164 |
| RAB-18 | CA2 | Predicted | 0.039 | RS-36 | CA2 | Predicted | 0.164 |
| RAB-18 | CASP3 | Validated | 1.000 | RS-36 | CCNA2 | Predicted | 0.953 |
| RAB-18 | CCNA2 | Predicted | 1.000 | RS-36 | CCT3 | Predicted | 0.156 |
| RAB-18 | CCT3 | Predicted | 0.004 | RS-36 | CTSB | Predicted | 0.157 |
| RAB-18 | CDK4 | Validated | 1.000 | RS-36 | ESR1 | Predicted | 0.151 |
| RAB-18 | CTSB | Predicted | 0.082 | RS-36 | GABRE | Predicted | 0.010 |
| RAB-18 | CXCL12 | Validated | 1.000 | RS-36 | GLUD1 | Predicted | 0.004 |
| RAB-18 | ESR1 | Predicted | 0.161 | RS-36 | GNAI1 | Predicted | 0.004 |
| RAB-18 | GABRE | Predicted | 0.003 | RS-36 | GNMT | Predicted | 0.004 |
| RAB-18 | GLUD1 | Predicted | 0.013 | RS-36 | GSTP1 | Predicted | 0.000 |
| RAB-18 | GNAI1 | Predicted | 0.005 | RS-36 | HSP90AA1 | Predicted | 0.004 |
| RAB-18 | GNMT | Predicted | 0.006 | RS-36 | HSP90B1 | Predicted | 0.004 |
| RAB-18 | GSTP1 | Predicted | 0.007 | RS-36 | HSPA1B | Predicted | 0.004 |
| RAB-18 | HAO1 | Predicted | 0.000 | RS-36 | HSPA8 | Predicted | 0.004 |
| RAB-18 | HSP90AA1 | Predicted | 0.004 | RS-36 | KRAS | Predicted | 0.069 |
| RAB-18 | HSP90B1 | Predicted | 0.004 | RS-36 | MAPK1 | Predicted | 0.007 |
| RAB-18 | HSPA1B | Predicted | 0.004 | RS-36 | MAPK3 | Predicted | 0.007 |
| RAB-18 | HSPA8 | Predicted | 0.004 | RS-36 | MAT1A | Predicted | 0.007 |
| RAB-18 | IGF2 | Validated | 1.000 | RS-36 | MMP12 | Predicted | 0.032 |
| RAB-18 | KRAS | Predicted | 0.020 | RS-36 | MMP14 | Predicted | 0.282 |
| RAB-18 | LYZ | Predicted | 0.081 | RS-36 | MMP2 | Predicted | 0.010 |

| RAB-18 | MAPK1 | Predicted | 0.079 | RS-36 | MMP3 | Predicted | 0.031 |
| --- | --- | --- | --- | --- | --- | --- | --- |
| RAB-18 | MAPK1 | Validated | 1.000 | RS-36 | MMP7 | Predicted | 0.010 |
| RAB-18 | MAPK3 | Predicted | 0.025 | RS-36 | MMP9 | Predicted | 0.010 |
| RAB-18 | MAT1A | Predicted | 0.004 | RS-36 | MTAP | Predicted | 0.004 |
| RAB-18 | MME | Predicted | 0.052 | RS-36 | NME1 | Predicted | 0.032 |
| RAB-18 | NME1 | Predicted | 0.018 | RS-36 | NOS2A | Predicted | 0.088 |
| RAB-18 | NME2 | Predicted | 0.005 | RS-36 | PGK1 | Predicted | 0.004 |
| RAB-18 | NOS2A | Validated | 1.000 | RS-36 | PTGS2 | Predicted | 0.071 |
| RAB-18 | PGK1 | Predicted | 0.004 | RS-36 | PTK2 | Predicted | 0.007 |
| RAB-18 | PTGS2 | Predicted | 0.109 | RS-36 | RHOA | Predicted | 0.039 |
| RAB-18 | PTK2 | Predicted | 0.004 | RS-36 | SRC | Predicted | 0.151 |
| RAB-18 | RHOA | Predicted | 0.005 | RS-36 | SULT2A1 | Predicted | 0.057 |
| RAB-18 | RND3 | Predicted | 0.006 | RS-36 | TRDMT1 | Predicted | 0.008 |
| RAB-18 | SERPINE1 | Predicted | 0.028 | RS-37 | ABCB1 | Predicted | 0.009 |
| RAB-18 | SGK1 | Predicted | 0.000 | RS-37 | ACSL1 | Predicted | 0.031 |
| RAB-18 | SRC | Predicted | 0.071 | RS-37 | AGXT | Predicted | 0.004 |
| RAB-18 | TGM2 | Predicted | 0.005 | RS-37 | ASS1 | Predicted | 0.009 |
| RAB-18 | TOP2A | Predicted | 0.047 | RS-37 | AURKA | Predicted | 0.002 |
| RAB-18 | TRDMT1 | Predicted | 0.006 | RS-37 | CA1 | Predicted | 0.082 |
| RAB-18 | VEGFA | Validated | 1.000 | RS-37 | CA2 | Predicted | 0.082 |
| RAB-19 | ACSL1 | Predicted | 0.032 | RS-37 | CCNA2 | Predicted | 1.000 |
| RAB-19 | ADH1C | Predicted | 0.006 | RS-37 | CCT3 | Predicted | 0.002 |
| RAB-19 | AGXT | Predicted | 0.008 | RS-37 | CES1 | Predicted | 0.031 |
| RAB-19 | AKR1B10 | Predicted | 0.112 | RS-37 | CTSB | Predicted | 0.104 |
| RAB-19 | AKT2 | Predicted | 0.081 | RS-37 | ESR1 | Predicted | 0.104 |

| RAB-19 | AURKA | Predicted | 0.006 | RS-37 | GABRE | Predicted | 0.015 |
| --- | --- | --- | --- | --- | --- | --- | --- |
| RAB-19 | CA1 | Predicted | 0.054 | RS-37 | GLUD1 | Predicted | 0.002 |
| RAB-19 | CA2 | Predicted | 0.054 | RS-37 | GNAI1 | Predicted | 0.001 |
| RAB-19 | CCNA2 | Predicted | 1.000 | RS-37 | GNMT | Predicted | 0.004 |
| RAB-19 | CCT3 | Predicted | 0.006 | RS-37 | GSTP1 | Predicted | 0.003 |
| RAB-19 | CES1 | Predicted | 0.006 | RS-37 | HSP90AA1 | Predicted | 0.002 |
| RAB-19 | CTSB | Predicted | 0.112 | RS-37 | HSP90B1 | Predicted | 0.002 |
| RAB-19 | ESR1 | Predicted | 0.217 | RS-37 | HSPA1B | Predicted | 0.002 |
| RAB-19 | GABRE | Predicted | 0.013 | RS-37 | HSPA8 | Predicted | 0.002 |
| RAB-19 | GLUD1 | Predicted | 0.017 | RS-37 | KRAS | Predicted | 0.026 |
| RAB-19 | GNAI1 | Predicted | 0.006 | RS-37 | MAPK1 | Predicted | 0.141 |
| RAB-19 | GNMT | Predicted | 0.007 | RS-37 | MAPK3 | Predicted | 0.034 |
| RAB-19 | GSTP1 | Predicted | 0.009 | RS-37 | MAT1A | Predicted | 0.002 |
| RAB-19 | HAO1 | Predicted | 0.000 | RS-37 | MTAP | Predicted | 0.105 |
| RAB-19 | HSP90AA1 | Predicted | 0.006 | RS-37 | NME1 | Predicted | 0.010 |
| RAB-19 | HSP90B1 | Predicted | 0.006 | RS-37 | NME2 | Predicted | 0.001 |
| RAB-19 | HSPA1B | Predicted | 0.006 | RS-37 | NOS2A | Predicted | 0.032 |
| RAB-19 | HSPA8 | Predicted | 0.006 | RS-37 | PGK1 | Predicted | 0.002 |
| RAB-19 | KRAS | Predicted | 0.022 | RS-37 | PTGS2 | Predicted | 0.104 |
| RAB-19 | LYZ | Predicted | 0.107 | RS-37 | PTK2 | Predicted | 0.002 |
| RAB-19 | MAPK1 | Predicted | 0.051 | RS-37 | RHOA | Predicted | 0.001 |
| RAB-19 | MAPK3 | Predicted | 0.031 | RS-37 | SERPINE1 | Predicted | 0.027 |
| RAB-19 | MAT1A | Predicted | 0.006 | RS-37 | SRC | Predicted | 0.106 |
| RAB-19 | MME | Predicted | 0.071 | RS-37 | SULT2A1 | Predicted | 0.007 |
| RAB-19 | MTAP | Predicted | 0.108 | RS-37 | TGM2 | Predicted | 0.001 |

| RAB-19 | NME1 | Predicted | 0.025 | RS-37 | TOP2A | Predicted | 0.049 |
| --- | --- | --- | --- | --- | --- | --- | --- |
| RAB-19 | NME2 | Predicted | 0.006 | RS-37 | TRDMT1 | Predicted | 0.004 |
| RAB-19 | NOS2A | Predicted | 0.109 | RS-38 | ADH1B | Predicted | 0.005 |
| RAB-19 | PGK1 | Predicted | 0.006 | RS-38 | ADH1C | Predicted | 0.000 |
| RAB-19 | PTGS2 | Predicted | 0.095 | RS-38 | ADH4 | Predicted | 0.000 |
| RAB-19 | PTK2 | Predicted | 0.006 | RS-38 | AKR1B10 | Predicted | 0.001 |
| RAB-19 | RHOA | Predicted | 0.006 | RS-38 | AKR1C2 | Predicted | 0.005 |
| RAB-19 | RND3 | Predicted | 0.007 | RS-38 | ALDH1A1 | Predicted | 0.000 |
| RAB-19 | SERPINE1 | Predicted | 0.019 | RS-38 | ALDH1B1 | Predicted | 0.000 |
| RAB-19 | SGK1 | Predicted | 0.000 | RS-38 | ALDH1L1 | Predicted | 0.001 |
| RAB-19 | SRC | Predicted | 0.144 | RS-38 | ALDH2 | Predicted | 0.000 |
| RAB-19 | SULT2A1 | Predicted | 0.011 | RS-38 | ALDH3A2 | Predicted | 0.000 |
| RAB-19 | TGM2 | Predicted | 0.006 | RS-38 | ALDH4A1 | Predicted | 0.000 |
| RAB-19 | TOP2A | Predicted | 0.057 | RS-38 | DCXR | Predicted | 0.001 |
| RAB-19 | TRDMT1 | Predicted | 0.007 | RS-38 | EGFR | Predicted | 0.279 |
| RAB-20 | ABCB1 | Predicted | 0.007 | RS-38 | ESR1 | Predicted | 0.138 |
| RAB-20 | ACSL1 | Predicted | 0.007 | RS-38 | FTCD | Predicted | 0.001 |
| RAB-20 | ADH1C | Predicted | 0.007 | RS-38 | GAPDH | Predicted | 0.000 |
| RAB-20 | AGXT | Predicted | 0.007 | RS-38 | GLUD1 | Predicted | 0.000 |
| RAB-20 | AKR1B10 | Predicted | 0.109 | RS-38 | GSTP1 | Predicted | 0.025 |
| RAB-20 | AKT2 | Predicted | 0.143 | RS-38 | HSD17B10 | Predicted | 0.000 |
| RAB-20 | ASS1 | Predicted | 0.007 | RS-38 | HSD17B4 | Predicted | 0.000 |
| RAB-20 | AURKA | Predicted | 0.005 | RS-38 | KRAS | Predicted | 0.046 |
| RAB-20 | CA1 | Predicted | 0.081 | RS-38 | MTHFD1 | Predicted | 0.010 |
| RAB-20 | CA2 | Predicted | 0.081 | RS-38 | MTR | Predicted | 0.001 |

| RAB-20 | CASP1 | Predicted | 0.017 | RS-38 | PTGS2 | Predicted | 0.033 |
| --- | --- | --- | --- | --- | --- | --- | --- |
| RAB-20 | CCNA2 | Predicted | 1.000 | RS-38 | QDPR | Predicted | 0.000 |
| RAB-20 | CCT3 | Predicted | 0.005 | RS-38 | RAF1 | Predicted | 0.020 |
| RAB-20 | CDK4 | Predicted | 0.041 | RS-38 | SERPINE1 | Predicted | 0.031 |
| RAB-20 | CES1 | Predicted | 0.007 | RS-38 | TUBB | Predicted | 0.022 |
| RAB-20 | CTSB | Predicted | 0.110 | RS-39 | ABCB1 | Predicted | 0.005 |
| RAB-20 | ESR1 | Predicted | 0.227 | RS-39 | ACSL1 | Predicted | 0.005 |
| RAB-20 | GABRE | Predicted | 0.001 | RS-39 | AGXT | Predicted | 0.003 |
| RAB-20 | GLUD1 | Predicted | 0.019 | RS-39 | AKR1B10 | Predicted | 0.061 |
| RAB-20 | GNAI1 | Predicted | 0.007 | RS-39 | AKT2 | Predicted | 0.023 |
| RAB-20 | GNMT | Predicted | 0.006 | RS-39 | ASS1 | Predicted | 0.005 |
| RAB-20 | HAO1 | Predicted | 0.000 | RS-39 | AURKA | Predicted | 0.003 |
| RAB-20 | HSP90AA1 | Predicted | 0.005 | RS-39 | CA1 | Predicted | 0.029 |
| RAB-20 | HSP90B1 | Predicted | 0.005 | RS-39 | CA2 | Predicted | 0.029 |
| RAB-20 | HSPA1B | Predicted | 0.005 | RS-39 | CCNA2 | Predicted | 1.000 |
| RAB-20 | HSPA8 | Predicted | 0.005 | RS-39 | CCT3 | Predicted | 0.003 |
| RAB-20 | KRAS | Predicted | 0.033 | RS-39 | CTSB | Predicted | 0.057 |
| RAB-20 | MAPK1 | Predicted | 0.041 | RS-39 | CYP2C8 | Predicted | 0.003 |
| RAB-20 | MAPK3 | Predicted | 0.086 | RS-39 | ESR1 | Predicted | 0.060 |
| RAB-20 | MAT1A | Predicted | 0.005 | RS-39 | GABRE | Predicted | 0.000 |
| RAB-20 | MME | Predicted | 0.073 | RS-39 | GLUD1 | Predicted | 0.009 |
| RAB-20 | MMP12 | Predicted | 0.034 | RS-39 | GNAI1 | Predicted | 0.002 |
| RAB-20 | MMP9 | Predicted | 0.017 | RS-39 | GNMT | Predicted | 0.003 |
| RAB-20 | NME1 | Predicted | 0.028 | RS-39 | HAO1 | Predicted | 0.000 |
| RAB-20 | NME2 | Predicted | 0.007 | RS-39 | HSP90AA1 | Predicted | 0.003 |

| RAB-20 | NOS2A | Predicted | 0.107 | RS-39 | HSP90B1 | Predicted | 0.003 |
| --- | --- | --- | --- | --- | --- | --- | --- |
| RAB-20 | PGK1 | Predicted | 0.005 | RS-39 | HSPA1B | Predicted | 0.003 |
| RAB-20 | PTGS2 | Predicted | 0.084 | RS-39 | HSPA8 | Predicted | 0.003 |
| RAB-20 | PTK2 | Predicted | 0.005 | RS-39 | KRAS | Predicted | 0.015 |
| RAB-20 | RHOA | Predicted | 0.007 | RS-39 | LYZ | Predicted | 0.060 |
| RAB-20 | RND3 | Predicted | 0.009 | RS-39 | MAPK1 | Predicted | 0.025 |
| RAB-20 | SERPINE1 | Predicted | 0.017 | RS-39 | MAT1A | Predicted | 0.003 |
| RAB-20 | SGK1 | Predicted | 0.000 | RS-39 | MME | Predicted | 0.058 |
| RAB-20 | SULT2A1 | Predicted | 0.010 | RS-39 | NME1 | Predicted | 0.014 |
| RAB-20 | TGM2 | Predicted | 0.007 | RS-39 | NME2 | Predicted | 0.002 |
| RAB-20 | TOP2A | Predicted | 0.064 | RS-39 | NOS2A | Predicted | 0.058 |
| RAB-20 | TRDMT1 | Predicted | 0.006 | RS-39 | PGK1 | Predicted | 0.003 |
| RAB-20 | VEGFA | Predicted | 0.017 | RS-39 | PTGS2 | Predicted | 0.063 |
| RAB-21 | ABCB1 | Predicted | 0.005 | RS-39 | PTK2 | Predicted | 0.003 |
| RAB-21 | ACSL1 | Predicted | 0.005 | RS-39 | RHOA | Predicted | 0.002 |
| RAB-21 | AKT2 | Predicted | 0.203 | RS-39 | RND3 | Predicted | 0.003 |
| RAB-21 | ASS1 | Predicted | 0.005 | RS-39 | SERPINE1 | Predicted | 0.014 |
| RAB-21 | AURKA | Predicted | 0.007 | RS-39 | SGK1 | Predicted | 0.000 |
| RAB-21 | CCNA2 | Predicted | 1.000 | RS-39 | SRC | Predicted | 0.055 |
| RAB-21 | CCT3 | Predicted | 0.020 | RS-39 | TGM2 | Predicted | 0.002 |
| RAB-21 | CES1 | Predicted | 0.043 | RS-39 | TOP2A | Predicted | 0.037 |
| RAB-21 | CTSB | Predicted | 0.139 | RS-39 | TRDMT1 | Predicted | 0.003 |
| RAB-21 | ESR1 | Predicted | 0.135 | RS-41 | ABCB1 | Predicted | 0.003 |
| RAB-21 | FGF1 | Predicted | 0.064 | RS-41 | ACSL1 | Predicted | 0.003 |
| RAB-21 | FGF2 | Predicted | 0.064 | RS-41 | AGXT | Predicted | 0.002 |

| RAB-21 | GABRE | Predicted | 0.015 | RS-41 | AKR1B10 | Predicted | 0.064 |
| --- | --- | --- | --- | --- | --- | --- | --- |
| RAB-21 | GLUD1 | Predicted | 0.033 | RS-41 | AKT2 | Predicted | 0.025 |
| RAB-21 | GNMT | Predicted | 0.000 | RS-41 | ALDH1A1 | Predicted | 0.009 |
| RAB-21 | HSP90AA1 | Predicted | 0.007 | RS-41 | ASS1 | Predicted | 0.003 |
| RAB-21 | HSP90B1 | Predicted | 0.007 | RS-41 | AURKA | Predicted | 0.001 |
| RAB-21 | HSPA1B | Predicted | 0.007 | RS-41 | CA1 | Predicted | 0.046 |
| RAB-21 | HSPA8 | Predicted | 0.007 | RS-41 | CA2 | Predicted | 0.046 |
| RAB-21 | KRAS | Predicted | 0.063 | RS-41 | CCNA2 | Predicted | 1.000 |
| RAB-21 | LYZ | Predicted | 0.064 | RS-41 | CCT3 | Predicted | 0.006 |
| RAB-21 | MAPK1 | Predicted | 0.059 | RS-41 | CTSB | Predicted | 0.062 |
| RAB-21 | MAT1A | Predicted | 0.007 | RS-41 | CYP2C8 | Predicted | 0.003 |
| RAB-21 | MME | Predicted | 0.090 | RS-41 | ESR1 | Predicted | 0.063 |
| RAB-21 | MMP12 | Predicted | 0.044 | RS-41 | GABRE | Predicted | 0.000 |
| RAB-21 | MMP14 | Predicted | 0.014 | RS-41 | GLUD1 | Predicted | 0.007 |
| RAB-21 | MMP2 | Predicted | 0.014 | RS-41 | GNAI1 | Predicted | 0.001 |
| RAB-21 | MMP3 | Predicted | 0.038 | RS-41 | GNMT | Predicted | 0.002 |
| RAB-21 | MMP7 | Predicted | 0.014 | RS-41 | GSTP1 | Predicted | 0.001 |
| RAB-21 | MMP9 | Predicted | 0.014 | RS-41 | HSP90AA1 | Predicted | 0.001 |
| RAB-21 | NME1 | Predicted | 0.021 | RS-41 | HSP90B1 | Predicted | 0.001 |
| RAB-21 | NOS2A | Predicted | 0.037 | RS-41 | HSPA1B | Predicted | 0.001 |
| RAB-21 | PGK1 | Predicted | 0.007 | RS-41 | HSPA8 | Predicted | 0.001 |
| RAB-21 | PTGS2 | Predicted | 0.083 | RS-41 | KRAS | Predicted | 0.022 |
| RAB-21 | PTK2 | Predicted | 0.007 | RS-41 | LYZ | Predicted | 0.062 |
| RAB-21 | RND3 | Predicted | 0.018 | RS-41 | MAPK1 | Predicted | 0.061 |
| RAB-21 | SRC | Predicted | 0.069 | RS-41 | MAT1A | Predicted | 0.001 |

| RAB-21 | SULT2A1 | Predicted | 0.013 | RS-41 | MTAP | Predicted | 0.060 |
| --- | --- | --- | --- | --- | --- | --- | --- |
| RAB-21 | TOP2A | Predicted | 0.028 | RS-41 | NME1 | Predicted | 0.012 |
| RAB-21 | TRDMT1 | Predicted | 0.000 | RS-41 | NME2 | Predicted | 0.001 |
| RAB-21 | VEGFA | Predicted | 0.031 | RS-41 | NOS2A | Predicted | 0.127 |
| RAB-22 | ABCB1 | Predicted | 0.013 | RS-41 | PGK1 | Predicted | 0.001 |
| RAB-22 | ACSL1 | Predicted | 0.013 | RS-41 | PTGS2 | Predicted | 0.040 |
| RAB-22 | ADH1C | Predicted | 0.012 | RS-41 | PTK2 | Predicted | 0.001 |
| RAB-22 | AGXT | Predicted | 0.013 | RS-41 | RARA | Predicted | 0.009 |
| RAB-22 | AKR1B10 | Predicted | 0.164 | RS-41 | RHOA | Predicted | 0.001 |
| RAB-22 | AKT2 | Predicted | 0.234 | RS-41 | RND3 | Predicted | 0.001 |
| RAB-22 | ASS1 | Predicted | 0.013 | RS-41 | SRC | Predicted | 0.063 |
| RAB-22 | AURKA | Predicted | 0.011 | RS-41 | SULT2A1 | Predicted | 0.003 |
| RAB-22 | CA1 | Predicted | 0.081 | RS-41 | TGM2 | Predicted | 0.001 |
| RAB-22 | CA2 | Predicted | 0.081 | RS-41 | TOP2A | Predicted | 0.038 |
| RAB-22 | CASP1 | Predicted | 0.037 | RS-41 | TRDMT1 | Predicted | 0.002 |
| RAB-22 | CCNA2 | Predicted | 1.000 | RS-42 | ACSL1 | Predicted | 0.042 |
| RAB-22 | CCT3 | Predicted | 0.011 | RS-42 | ALDH2 | Predicted | 0.152 |
| RAB-22 | CDK4 | Predicted | 0.059 | RS-42 | ALDOA | Predicted | 0.154 |
| RAB-22 | CES1 | Predicted | 0.012 | RS-42 | AURKA | Predicted | 0.016 |
| RAB-22 | CTSB | Predicted | 0.167 | RS-42 | CA1 | Predicted | 0.138 |
| RAB-22 | ESR1 | Predicted | 0.176 | RS-42 | CA2 | Predicted | 0.138 |
| RAB-22 | GABRE | Predicted | 0.004 | RS-42 | CCNA2 | Predicted | 0.800 |
| RAB-22 | GLUD1 | Predicted | 0.029 | RS-42 | CCT3 | Predicted | 0.004 |
| RAB-22 | GNAI1 | Predicted | 0.011 | RS-42 | CES1 | Predicted | 0.045 |
| RAB-22 | GNMT | Predicted | 0.007 | RS-42 | CTSB | Predicted | 0.155 |

| RAB-22 | HAO1 | Predicted | 0.000 | RS-42 | ESR1 | Predicted | 0.350 |
| --- | --- | --- | --- | --- | --- | --- | --- |
| RAB-22 | HSP90AA1 | Predicted | 0.011 | RS-42 | GABRE | Predicted | 0.048 |
| RAB-22 | HSP90B1 | Predicted | 0.011 | RS-42 | GLUD1 | Predicted | 0.004 |
| RAB-22 | HSPA1B | Predicted | 0.011 | RS-42 | GNAI1 | Predicted | 0.005 |
| RAB-22 | HSPA8 | Predicted | 0.011 | RS-42 | GSTP1 | Predicted | 0.000 |
| RAB-22 | KRAS | Predicted | 0.052 | RS-42 | HSP90AA1 | Predicted | 0.004 |
| RAB-22 | MAPK1 | Predicted | 0.059 | RS-42 | HSP90B1 | Predicted | 0.004 |
| RAB-22 | MAPK3 | Predicted | 0.120 | RS-42 | HSPA1B | Predicted | 0.004 |
| RAB-22 | MAT1A | Predicted | 0.011 | RS-42 | HSPA8 | Predicted | 0.004 |
| RAB-22 | MME | Predicted | 0.113 | RS-42 | KRAS | Predicted | 0.052 |
| RAB-22 | MMP12 | Predicted | 0.053 | RS-42 | MAPK3 | Predicted | 0.100 |
| RAB-22 | MMP9 | Predicted | 0.037 | RS-42 | MAT1A | Predicted | 0.004 |
| RAB-22 | NME1 | Predicted | 0.043 | RS-42 | MMP12 | Predicted | 0.039 |
| RAB-22 | NME2 | Predicted | 0.011 | RS-42 | MMP14 | Predicted | 0.010 |
| RAB-22 | PGK1 | Predicted | 0.011 | RS-42 | MMP2 | Predicted | 0.010 |
| RAB-22 | PTGS2 | Predicted | 0.055 | RS-42 | MMP3 | Predicted | 0.032 |
| RAB-22 | PTK2 | Predicted | 0.011 | RS-42 | MMP7 | Predicted | 0.010 |
| RAB-22 | RHOA | Predicted | 0.011 | RS-42 | MMP9 | Predicted | 0.010 |
| RAB-22 | RND3 | Predicted | 0.013 | RS-42 | MTAP | Predicted | 0.325 |
| RAB-22 | SERPINE1 | Predicted | 0.028 | RS-42 | NME1 | Predicted | 0.029 |
| RAB-22 | SGK1 | Predicted | 0.000 | RS-42 | NME2 | Predicted | 0.005 |
| RAB-22 | SRC | Predicted | 0.162 | RS-42 | NOS2A | Predicted | 0.318 |
| RAB-22 | SULT2A1 | Predicted | 0.018 | RS-42 | PGK1 | Predicted | 0.004 |
| RAB-22 | TGM2 | Predicted | 0.011 | RS-42 | PTGS2 | Predicted | 0.543 |
| RAB-22 | TOP2A | Predicted | 0.089 | RS-42 | PTK2 | Predicted | 0.004 |

| RAB-22 | TRDMT1 | Predicted | 0.007 | RS-42 | RHOA | Predicted | 0.005 |
| --- | --- | --- | --- | --- | --- | --- | --- |
| RAB-22 | VEGFA | Predicted | 0.088 | RS-42 | SULT2A1 | Predicted | 0.012 |
| RAB-23 | ABCB1 | Predicted | 0.013 | RS-42 | TGM2 | Predicted | 0.005 |
| RAB-23 | ACSL1 | Predicted | 0.013 | RS-43 | ACSL1 | Predicted | 0.017 |
| RAB-23 | ADH1C | Predicted | 0.014 | RS-43 | AGXT | Predicted | 0.003 |
| RAB-23 | AGXT | Predicted | 0.012 | RS-43 | AKR1B10 | Predicted | 0.083 |
| RAB-23 | AKR1B10 | Predicted | 0.162 | RS-43 | ALDH2 | Predicted | 0.084 |
| RAB-23 | AKT2 | Predicted | 0.232 | RS-43 | ALDOA | Predicted | 0.083 |
| RAB-23 | ASS1 | Predicted | 0.013 | RS-43 | AURKA | Predicted | 0.007 |
| RAB-23 | AURKA | Predicted | 0.010 | RS-43 | CA1 | Predicted | 0.137 |
| RAB-23 | CA1 | Predicted | 0.080 | RS-43 | CA2 | Predicted | 0.109 |
| RAB-23 | CA2 | Predicted | 0.080 | RS-43 | CCNA2 | Predicted | 1.000 |
| RAB-23 | CASP1 | Predicted | 0.033 | RS-43 | CCT3 | Predicted | 0.001 |
| RAB-23 | CCNA2 | Predicted | 1.000 | RS-43 | CTSB | Predicted | 0.084 |
| RAB-23 | CCT3 | Predicted | 0.010 | RS-43 | ESR1 | Predicted | 0.121 |
| RAB-23 | CDK4 | Predicted | 0.067 | RS-43 | GABRE | Predicted | 0.006 |
| RAB-23 | CES1 | Predicted | 0.014 | RS-43 | GLUD1 | Predicted | 0.001 |
| RAB-23 | CTSB | Predicted | 0.163 | RS-43 | GNAI1 | Predicted | 0.002 |
| RAB-23 | ESR1 | Predicted | 0.342 | RS-43 | GSTP1 | Predicted | 0.001 |
| RAB-23 | GABRE | Predicted | 0.004 | RS-43 | HSP90AA1 | Predicted | 0.001 |
| RAB-23 | GLUD1 | Predicted | 0.029 | RS-43 | HSP90B1 | Predicted | 0.001 |
| RAB-23 | GNAI1 | Predicted | 0.012 | RS-43 | HSPA1B | Predicted | 0.001 |
| RAB-23 | GNMT | Predicted | 0.007 | RS-43 | HSPA8 | Predicted | 0.001 |
| RAB-23 | GSTP1 | Predicted | 0.052 | RS-43 | KRAS | Predicted | 0.030 |
| RAB-23 | HAO1 | Predicted | 0.000 | RS-43 | MAPK3 | Predicted | 0.042 |

| RAB-23 | HSP90AA1 | Predicted | 0.010 | RS-43 | MAT1A | Predicted | 0.001 |
| --- | --- | --- | --- | --- | --- | --- | --- |
| RAB-23 | HSP90B1 | Predicted | 0.010 | RS-43 | MTAP | Predicted | 0.178 |
| RAB-23 | HSPA1B | Predicted | 0.010 | RS-43 | NME1 | Predicted | 0.009 |
| RAB-23 | HSPA8 | Predicted | 0.010 | RS-43 | NME2 | Predicted | 0.002 |
| RAB-23 | KRAS | Predicted | 0.052 | RS-43 | NOS2A | Predicted | 0.180 |
| RAB-23 | MAPK1 | Predicted | 0.067 | RS-43 | PGK1 | Predicted | 0.001 |
| RAB-23 | MAPK3 | Predicted | 0.067 | RS-43 | PTGS2 | Predicted | 0.249 |
| RAB-23 | MAT1A | Predicted | 0.010 | RS-43 | PTK2 | Predicted | 0.001 |
| RAB-23 | MME | Predicted | 0.111 | RS-43 | RHOA | Predicted | 0.002 |
| RAB-23 | MMP12 | Predicted | 0.053 | RS-43 | SRC | Predicted | 0.089 |
| RAB-23 | MMP9 | Predicted | 0.033 | RS-43 | SULT2A1 | Predicted | 0.004 |
| RAB-23 | NME1 | Predicted | 0.043 | RS-43 | TGM2 | Predicted | 0.002 |
| RAB-23 | NME2 | Predicted | 0.012 | RS-43 | TOP2A | Predicted | 0.058 |
| RAB-23 | PGK1 | Predicted | 0.010 | RS-44 | ABCB1 | Predicted | 0.003 |
| RAB-23 | PTK2 | Predicted | 0.010 | RS-44 | ACSL1 | Predicted | 0.003 |
| RAB-23 | RHOA | Predicted | 0.012 | RS-44 | AGXT | Predicted | 0.008 |
| RAB-23 | RND3 | Predicted | 0.013 | RS-44 | AKT2 | Predicted | 0.102 |
| RAB-23 | SERPINE1 | Predicted | 0.029 | RS-44 | ASS1 | Predicted | 0.003 |
| RAB-23 | SGK1 | Predicted | 0.000 | RS-44 | AURKA | Predicted | 0.009 |
| RAB-23 | SRC | Predicted | 0.162 | RS-44 | CA1 | Predicted | 0.042 |
| RAB-23 | TGM2 | Predicted | 0.012 | RS-44 | CA2 | Predicted | 0.042 |
| RAB-23 | TOP2A | Predicted | 0.089 | RS-44 | CCNA2 | Predicted | 0.849 |
| RAB-23 | TRDMT1 | Predicted | 0.007 | RS-44 | CCT3 | Predicted | 0.024 |
| RAB-23 | VEGFA | Predicted | 0.080 | RS-44 | CES1 | Predicted | 0.043 |
| RAB-24 | ABCB1 | Predicted | 0.009 | RS-44 | CTSB | Predicted | 0.134 |

| RAB-24 | ACSL1 | Predicted | 0.009 | RS-44 | ESR1 | Predicted | 0.549 |
| --- | --- | --- | --- | --- | --- | --- | --- |
| RAB-24 | ADH1C | Predicted | 0.008 | RS-44 | FGF1 | Predicted | 0.063 |
| RAB-24 | AGXT | Predicted | 0.008 | RS-44 | FGF2 | Predicted | 0.063 |
| RAB-24 | AKR1B10 | Predicted | 0.111 | RS-44 | GABRE | Predicted | 0.010 |
| RAB-24 | AKT2 | Predicted | 0.089 | RS-44 | GAPDH | Predicted | 0.014 |
| RAB-24 | ALDH1L1 | Predicted | 0.003 | RS-44 | GLUD1 | Predicted | 0.023 |
| RAB-24 | ASS1 | Predicted | 0.009 | RS-44 | GNAI1 | Predicted | 0.006 |
| RAB-24 | AURKA | Predicted | 0.135 | RS-44 | GNMT | Predicted | 0.008 |
| RAB-24 | CA1 | Predicted | 0.053 | RS-44 | HAO1 | Predicted | 0.005 |
| RAB-24 | CA2 | Predicted | 0.053 | RS-44 | HSP90AA1 | Predicted | 0.009 |
| RAB-24 | CASP1 | Predicted | 0.025 | RS-44 | HSP90B1 | Predicted | 0.009 |
| RAB-24 | CCNA2 | Predicted | 1.000 | RS-44 | HSPA1B | Predicted | 0.009 |
| RAB-24 | CCT3 | Predicted | 0.007 | RS-44 | HSPA8 | Predicted | 0.009 |
| RAB-24 | CDK4 | Predicted | 0.045 | RS-44 | KRAS | Predicted | 0.093 |
| RAB-24 | CES1 | Predicted | 0.008 | RS-44 | LYZ | Predicted | 0.066 |
| RAB-24 | CTSB | Predicted | 0.109 | RS-44 | MAPK1 | Predicted | 0.010 |
| RAB-24 | DDR1 | Predicted | 0.019 | RS-44 | MAT1A | Predicted | 0.009 |
| RAB-24 | ESR1 | Predicted | 0.137 | RS-44 | MME | Predicted | 0.088 |
| RAB-24 | FTCD | Predicted | 0.003 | RS-44 | MMP12 | Predicted | 0.043 |
| RAB-24 | GABRE | Predicted | 0.003 | RS-44 | MMP14 | Predicted | 0.014 |
| RAB-24 | GLUD1 | Predicted | 0.021 | RS-44 | MMP2 | Predicted | 0.014 |
| RAB-24 | GNAI1 | Predicted | 0.008 | RS-44 | MMP3 | Predicted | 0.037 |
| RAB-24 | GNMT | Predicted | 0.005 | RS-44 | MMP7 | Predicted | 0.014 |
| RAB-24 | GSTP1 | Predicted | 0.023 | RS-44 | MMP9 | Predicted | 0.014 |
| RAB-24 | HAO1 | Predicted | 0.000 | RS-44 | NME1 | Predicted | 0.035 |

| RAB-24 | HSP90AA1 | Predicted | 0.007 | RS-44 | NME2 | Predicted | 0.006 |
| --- | --- | --- | --- | --- | --- | --- | --- |
| RAB-24 | HSP90B1 | Predicted | 0.007 | RS-44 | NOS2A | Predicted | 0.028 |
| RAB-24 | HSPA1B | Predicted | 0.007 | RS-44 | PGK1 | Predicted | 0.009 |
| RAB-24 | HSPA8 | Predicted | 0.007 | RS-44 | PTGS2 | Predicted | 0.101 |
| RAB-24 | KRAS | Predicted | 0.034 | RS-44 | PTK2 | Predicted | 0.009 |
| RAB-24 | MAPK1 | Predicted | 0.045 | RS-44 | RHOA | Predicted | 0.006 |
| RAB-24 | MAPK3 | Predicted | 0.091 | RS-44 | RND3 | Predicted | 0.008 |
| RAB-24 | MAT1A | Predicted | 0.007 | RS-44 | SERPINE1 | Predicted | 0.038 |
| RAB-24 | MME | Predicted | 0.074 | RS-44 | SGK1 | Predicted | 0.005 |
| RAB-24 | MMP12 | Predicted | 0.035 | RS-44 | SRC | Predicted | 0.179 |
| RAB-24 | MMP9 | Predicted | 0.025 | RS-44 | SULT2A1 | Predicted | 0.014 |
| RAB-24 | MTHFD1 | Predicted | 0.003 | RS-44 | TGM2 | Predicted | 0.006 |
| RAB-24 | MTR | Predicted | 0.003 | RS-44 | TOP2A | Predicted | 0.119 |
| RAB-24 | NME1 | Predicted | 0.029 | RS-44 | TRDMT1 | Predicted | 0.008 |
| RAB-24 | NME2 | Predicted | 0.008 | RS-45 | ACSL1 | Predicted | 0.017 |
| RAB-24 | NOS2A | Predicted | 0.107 | RS-45 | AURKA | Predicted | 0.000 |
| RAB-24 | PDGFRA | Predicted | 0.019 | RS-45 | CA1 | Predicted | 0.138 |
| RAB-24 | PGK1 | Predicted | 0.007 | RS-45 | CA2 | Predicted | 0.110 |
| RAB-24 | PTGS2 | Predicted | 0.087 | RS-45 | CCNA2 | Predicted | 1.000 |
| RAB-24 | PTK2 | Predicted | 0.007 | RS-45 | CCT3 | Predicted | 0.000 |
| RAB-24 | RAF1 | Predicted | 0.018 | RS-45 | CES1 | Predicted | 0.025 |
| RAB-24 | RARA | Predicted | 0.023 | RS-45 | CTSB | Predicted | 0.086 |
| RAB-24 | RHOA | Predicted | 0.008 | RS-45 | ESR1 | Predicted | 0.080 |
| RAB-24 | RND3 | Predicted | 0.010 | RS-45 | GLUD1 | Predicted | 0.000 |
| RAB-24 | SERPINE1 | Predicted | 0.022 | RS-45 | GNAI1 | Predicted | 0.002 |

| RAB-24 | SGK1 | Predicted | 0.000 | RS-45 | GSTP1 | Predicted | 0.002 |
| --- | --- | --- | --- | --- | --- | --- | --- |
| RAB-24 | SRC | Predicted | 0.018 | RS-45 | HSP90AA1 | Predicted | 0.000 |
| RAB-24 | TGM2 | Predicted | 0.008 | RS-45 | HSP90B1 | Predicted | 0.000 |
| RAB-24 | TOP2A | Predicted | 0.059 | RS-45 | HSPA1B | Predicted | 0.000 |
| RAB-24 | TRDMT1 | Predicted | 0.005 | RS-45 | HSPA8 | Predicted | 0.000 |
| RAB-24 | VEGFA | Predicted | 0.058 | RS-45 | KRAS | Predicted | 0.015 |
| RAB-25 | ABCB1 | Predicted | 0.009 | RS-45 | LYZ | Predicted | 0.086 |
| RAB-25 | ACSL1 | Predicted | 0.009 | RS-45 | MAPK3 | Predicted | 0.054 |
| RAB-25 | ADH1C | Predicted | 0.008 | RS-45 | MAT1A | Predicted | 0.000 |
| RAB-25 | AGXT | Predicted | 0.008 | RS-45 | MTAP | Predicted | 0.179 |
| RAB-25 | AKR1B10 | Predicted | 0.111 | RS-45 | NME1 | Predicted | 0.007 |
| RAB-25 | AKT2 | Predicted | 0.069 | RS-45 | NME2 | Predicted | 0.002 |
| RAB-25 | ASS1 | Predicted | 0.009 | RS-45 | NOS2A | Predicted | 0.180 |
| RAB-25 | AURKA | Predicted | 0.007 | RS-45 | PGK1 | Predicted | 0.000 |
| RAB-25 | CA1 | Predicted | 0.053 | RS-45 | PTGS2 | Predicted | 0.293 |
| RAB-25 | CA2 | Predicted | 0.053 | RS-45 | PTK2 | Predicted | 0.000 |
| RAB-25 | CASP1 | Predicted | 0.025 | RS-45 | RHOA | Predicted | 0.002 |
| RAB-25 | CCNA2 | Predicted | 1.000 | RS-45 | SRC | Predicted | 0.086 |
| RAB-25 | CCT3 | Predicted | 0.007 | RS-45 | SULT2A1 | Predicted | 0.002 |
| RAB-25 | CDK4 | Predicted | 0.045 | RS-45 | TGM2 | Predicted | 0.002 |
| RAB-25 | CES1 | Predicted | 0.008 | RS-45 | TOP2A | Predicted | 0.020 |
| RAB-25 | CTSB | Predicted | 0.109 | RS-47 | ACSL1 | Predicted | 0.029 |
| RAB-25 | ESR1 | Predicted | 0.334 | RS-47 | AGXT | Predicted | 0.008 |
| RAB-25 | GABRE | Predicted | 0.003 | RS-47 | ALDOA | Predicted | 0.136 |
| RAB-25 | GLUD1 | Predicted | 0.021 | RS-47 | AURKA | Predicted | 0.014 |

| RAB-25 | GNAI1 | Predicted | 0.008 | RS-47 | CA1 | Predicted | 0.148 |
| --- | --- | --- | --- | --- | --- | --- | --- |
| RAB-25 | GNMT | Predicted | 0.005 | RS-47 | CA2 | Predicted | 0.148 |
| RAB-25 | GSTP1 | Predicted | 0.035 | RS-47 | CCNA2 | Predicted | 0.853 |
| RAB-25 | HAO1 | Predicted | 0.000 | RS-47 | CCT3 | Predicted | 0.004 |
| RAB-25 | HSP90AA1 | Predicted | 0.007 | RS-47 | CES1 | Predicted | 0.040 |
| RAB-25 | HSP90B1 | Predicted | 0.007 | RS-47 | CTSB | Predicted | 0.135 |
| RAB-25 | HSPA1B | Predicted | 0.007 | RS-47 | ESR1 | Predicted | 0.204 |
| RAB-25 | HSPA8 | Predicted | 0.007 | RS-47 | GABRE | Predicted | 0.035 |
| RAB-25 | KRAS | Predicted | 0.034 | RS-47 | GLUD1 | Predicted | 0.004 |
| RAB-25 | MAPK1 | Predicted | 0.045 | RS-47 | GNAI1 | Predicted | 0.006 |
| RAB-25 | MAPK3 | Predicted | 0.091 | RS-47 | GSTP1 | Predicted | 0.004 |
| RAB-25 | MAT1A | Predicted | 0.007 | RS-47 | HSP90AA1 | Predicted | 0.004 |
| RAB-25 | MME | Predicted | 0.074 | RS-47 | HSP90B1 | Predicted | 0.004 |
| RAB-25 | MMP12 | Predicted | 0.035 | RS-47 | HSPA1B | Predicted | 0.004 |
| RAB-25 | MMP9 | Predicted | 0.025 | RS-47 | HSPA8 | Predicted | 0.004 |
| RAB-25 | NME1 | Predicted | 0.029 | RS-47 | KRAS | Predicted | 0.048 |
| RAB-25 | NME2 | Predicted | 0.008 | RS-47 | MAPK3 | Predicted | 0.085 |
| RAB-25 | NOS2A | Predicted | 0.107 | RS-47 | MAT1A | Predicted | 0.004 |
| RAB-25 | PGK1 | Predicted | 0.007 | RS-47 | MMP14 | Predicted | 0.000 |
| RAB-25 | PTGS2 | Predicted | 0.087 | RS-47 | MMP2 | Predicted | 0.000 |
| RAB-25 | PTK2 | Predicted | 0.007 | RS-47 | MMP3 | Predicted | 0.000 |
| RAB-25 | RHOA | Predicted | 0.008 | RS-47 | MMP7 | Predicted | 0.000 |
| RAB-25 | RND3 | Predicted | 0.010 | RS-47 | MMP9 | Predicted | 0.000 |
| RAB-25 | SERPINE1 | Predicted | 0.018 | RS-47 | MTAP | Predicted | 0.290 |
| RAB-25 | SGK1 | Predicted | 0.000 | RS-47 | NME1 | Predicted | 0.017 |

| RAB-25 | SRC | Predicted | 0.217 | RS-47 | NME2 | Predicted | 0.006 |
| --- | --- | --- | --- | --- | --- | --- | --- |
| RAB-25 | TGM2 | Predicted | 0.008 | RS-47 | NOS2A | Predicted | 0.283 |
| RAB-25 | TOP2A | Predicted | 0.059 | RS-47 | PGK1 | Predicted | 0.004 |
| RAB-25 | TRDMT1 | Predicted | 0.005 | RS-47 | PTGS2 | Predicted | 0.463 |
| RAB-25 | VEGFA | Predicted | 0.058 | RS-47 | PTK2 | Predicted | 0.004 |
| RAB-26 | ABCB1 | Predicted | 0.010 | RS-47 | RHOA | Predicted | 0.006 |
| RAB-26 | ACSL1 | Predicted | 0.010 | RS-47 | SRC | Predicted | 0.136 |
| RAB-26 | ADH1C | Predicted | 0.007 | RS-47 | SULT2A1 | Predicted | 0.011 |
| RAB-26 | AGXT | Predicted | 0.010 | RS-47 | TGM2 | Predicted | 0.006 |
| RAB-26 | AKR1B10 | Predicted | 0.158 | RS-47 | TOP2A | Predicted | 0.053 |
| RAB-26 | AKT2 | Predicted | 0.231 | RS-48 | ACSL1 | Predicted | 0.022 |
| RAB-26 | ALDH1L1 | Predicted | 0.008 | RS-48 | ALDH2 | Predicted | 0.080 |
| RAB-26 | ASS1 | Predicted | 0.010 | RS-48 | ALDOA | Predicted | 0.082 |
| RAB-26 | AURKA | Predicted | 0.009 | RS-48 | AR | Predicted | 0.081 |
| RAB-26 | CA1 | Predicted | 0.077 | RS-48 | B2M | Predicted | 0.017 |
| RAB-26 | CA2 | Predicted | 0.077 | RS-48 | CA1 | Predicted | 0.200 |
| RAB-26 | CASP1 | Predicted | 0.034 | RS-48 | CA2 | Predicted | 0.200 |
| RAB-26 | CCNA2 | Predicted | 1.000 | RS-48 | CCNA2 | Predicted | 0.169 |
| RAB-26 | CCT3 | Predicted | 0.009 | RS-48 | ESR1 | Predicted | 0.114 |
| RAB-26 | CDK4 | Predicted | 0.060 | RS-48 | GABRE | Predicted | 0.015 |
| RAB-26 | CES1 | Predicted | 0.007 | RS-48 | GSTP1 | Predicted | 0.000 |
| RAB-26 | CTSB | Predicted | 0.162 | RS-48 | KRAS | Predicted | 0.007 |
| RAB-26 | DDR1 | Predicted | 0.065 | RS-48 | LCN2 | Predicted | 0.081 |
| RAB-26 | ESR1 | Predicted | 0.504 | RS-48 | MAPK1 | Predicted | 0.033 |
| RAB-26 | FTCD | Predicted | 0.008 | RS-48 | MTAP | Predicted | 0.173 |

| RAB-26 | GABRE | Predicted | 0.005 | RS-48 | NOS2A | Predicted | 0.108 |
| --- | --- | --- | --- | --- | --- | --- | --- |
| RAB-26 | GLUD1 | Predicted | 0.029 | RS-48 | PARP1 | Predicted | 0.082 |
| RAB-26 | GNAI1 | Predicted | 0.009 | RS-48 | PC | Predicted | 0.006 |
| RAB-26 | GNMT | Predicted | 0.005 | RS-48 | PTGS2 | Predicted | 1.000 |
| RAB-26 | GSTP1 | Predicted | 0.049 | RS-48 | SRC | Predicted | 0.022 |
| RAB-26 | HAO1 | Predicted | 0.000 | RS-48 | TNFSF11 | Predicted | 0.024 |
| RAB-26 | HSP90AA1 | Predicted | 0.009 | RS-49 | ACSL1 | Predicted | 0.024 |
| RAB-26 | HSP90B1 | Predicted | 0.009 | RS-49 | AGXT | Predicted | 0.002 |
| RAB-26 | HSPA1B | Predicted | 0.009 | RS-49 | ALDOA | Predicted | 0.121 |
| RAB-26 | HSPA8 | Predicted | 0.009 | RS-49 | AURKA | Predicted | 0.001 |
| RAB-26 | KRAS | Predicted | 0.049 | RS-49 | CA1 | Predicted | 0.250 |
| RAB-26 | MAPK1 | Predicted | 0.129 | RS-49 | CA2 | Predicted | 0.163 |
| RAB-26 | MAPK3 | Predicted | 0.129 | RS-49 | CCNA2 | Predicted | 0.970 |
| RAB-26 | MAT1A | Predicted | 0.009 | RS-49 | CCT3 | Predicted | 0.001 |
| RAB-26 | MME | Predicted | 0.334 | RS-49 | CTSB | Predicted | 0.113 |
| RAB-26 | MMP12 | Predicted | 0.050 | RS-49 | ESR1 | Predicted | 0.049 |
| RAB-26 | MMP9 | Predicted | 0.034 | RS-49 | GLUD1 | Predicted | 0.001 |
| RAB-26 | MTHFD1 | Predicted | 0.008 | RS-49 | GNAI1 | Predicted | 0.000 |
| RAB-26 | MTR | Predicted | 0.008 | RS-49 | GSTP1 | Predicted | 0.001 |
| RAB-26 | NME1 | Predicted | 0.042 | RS-49 | HSP90AA1 | Predicted | 0.001 |
| RAB-26 | NME2 | Predicted | 0.009 | RS-49 | HSP90B1 | Predicted | 0.001 |
| RAB-26 | PDGFRA | Predicted | 0.065 | RS-49 | HSPA1B | Predicted | 0.001 |
| RAB-26 | PGK1 | Predicted | 0.009 | RS-49 | HSPA8 | Predicted | 0.001 |
| RAB-26 | PTGS2 | Predicted | 0.060 | RS-49 | KRAS | Predicted | 0.019 |
| RAB-26 | PTK2 | Predicted | 0.009 | RS-49 | LCN2 | Predicted | 0.113 |

| RAB-26 | RAF1 | Predicted | 0.043 | RS-49 | LYZ | Predicted | 0.111 |
| --- | --- | --- | --- | --- | --- | --- | --- |
| RAB-26 | RHOA | Predicted | 0.009 | RS-49 | MAPK3 | Predicted | 0.073 |
| RAB-26 | RND3 | Predicted | 0.013 | RS-49 | MAT1A | Predicted | 0.001 |
| RAB-26 | SERPINE1 | Predicted | 0.013 | RS-49 | MTAP | Predicted | 0.247 |
| RAB-26 | SGK1 | Predicted | 0.000 | RS-49 | NME1 | Predicted | 0.011 |
| RAB-26 | SRC | Predicted | 0.214 | RS-49 | NME2 | Predicted | 0.000 |
| RAB-26 | SULT2A1 | Predicted | 0.015 | RS-49 | NOS2A | Predicted | 0.247 |
| RAB-26 | TGM2 | Predicted | 0.009 | RS-49 | PGK1 | Predicted | 0.001 |
| RAB-26 | TOP2A | Predicted | 0.087 | RS-49 | PTGS2 | Predicted | 0.497 |
| RAB-26 | TRDMT1 | Predicted | 0.005 | RS-49 | PTK2 | Predicted | 0.001 |
| RAB-26 | VEGFA | Predicted | 0.077 | RS-49 | RHOA | Predicted | 0.000 |
| RAB-27 | ABCB1 | Predicted | 0.011 | RS-49 | SULT2A1 | Predicted | 0.006 |
| RAB-27 | ACSL1 | Predicted | 0.011 | RS-49 | TGM2 | Predicted | 0.000 |
| RAB-27 | ADH1C | Predicted | 0.009 | RS-49 | TOP2A | Predicted | 0.019 |
| RAB-27 | AGXT | Predicted | 0.008 | RS-50 | ACSL1 | Predicted | 0.026 |
| RAB-27 | ASS1 | Predicted | 0.011 | RS-50 | ALDH2 | Predicted | 0.087 |
| RAB-27 | AURKA | Predicted | 0.008 | RS-50 | ALDOA | Predicted | 0.085 |
| RAB-27 | CA1 | Predicted | 0.040 | RS-50 | CA1 | Predicted | 0.153 |
| RAB-27 | CA2 | Predicted | 0.040 | RS-50 | CA2 | Predicted | 0.074 |
| RAB-27 | CCNA2 | Predicted | 0.975 | RS-50 | CCNA2 | Predicted | 0.174 |
| RAB-27 | CCT3 | Predicted | 0.021 | RS-50 | CTSB | Predicted | 0.085 |
| RAB-27 | CES1 | Predicted | 0.028 | RS-50 | ESR1 | Predicted | 0.474 |
| RAB-27 | CTSB | Predicted | 0.134 | RS-50 | GABRE | Predicted | 0.015 |
| RAB-27 | ESR1 | Predicted | 0.419 | RS-50 | GSTP1 | Predicted | 0.000 |
| RAB-27 | FGF1 | Predicted | 0.065 | RS-50 | KRAS | Predicted | 0.007 |

| RAB-27 | FGF2 | Predicted | 0.065 | RS-50 | LCN2 | Predicted | 0.085 |
| --- | --- | --- | --- | --- | --- | --- | --- |
| RAB-27 | GABRE | Predicted | 0.013 | RS-50 | MTAP | Predicted | 0.176 |
| RAB-27 | GAPDH | Predicted | 0.022 | RS-50 | NOS2A | Predicted | 0.179 |
| RAB-27 | GLUD1 | Predicted | 0.021 | RS-50 | PTGS2 | Predicted | 0.885 |
| RAB-27 | GNAI1 | Predicted | 0.007 | RS-50 | SRC | Predicted | 0.084 |
| RAB-27 | GNMT | Predicted | 0.005 | RS-50 | TOP2A | Predicted | 0.055 |
| RAB-27 | HAO1 | Predicted | 0.005 | RS-51 | ACSL1 | Predicted | 0.019 |
| RAB-27 | HSP90AA1 | Predicted | 0.008 | RS-51 | ALDH2 | Predicted | 0.099 |
| RAB-27 | HSP90B1 | Predicted | 0.008 | RS-51 | ALDOA | Predicted | 0.103 |
| RAB-27 | HSPA1B | Predicted | 0.008 | RS-51 | AURKA | Predicted | 0.101 |
| RAB-27 | HSPA8 | Predicted | 0.008 | RS-51 | CA1 | Predicted | 0.163 |
| RAB-27 | KRAS | Predicted | 0.102 | RS-51 | CA2 | Predicted | 0.129 |
| RAB-27 | LYZ | Predicted | 0.067 | RS-51 | CCNA2 | Predicted | 0.973 |
| RAB-27 | MAPK1 | Predicted | 0.010 | RS-51 | CTSB | Predicted | 0.102 |
| RAB-27 | MAT1A | Predicted | 0.008 | RS-51 | ESR1 | Predicted | 0.213 |
| RAB-27 | MET | Predicted | 0.136 | RS-51 | GABRE | Predicted | 0.027 |
| RAB-27 | MME | Predicted | 0.090 | RS-51 | GNAI1 | Predicted | 0.001 |
| RAB-27 | MMP12 | Predicted | 0.055 | RS-51 | GSTP1 | Predicted | 0.000 |
| RAB-27 | MMP14 | Predicted | 0.016 | RS-51 | KRAS | Predicted | 0.016 |
| RAB-27 | MMP2 | Predicted | 0.016 | RS-51 | LCN2 | Predicted | 0.101 |
| RAB-27 | MMP3 | Predicted | 0.016 | RS-51 | MAPK3 | Predicted | 0.063 |
| RAB-27 | MMP7 | Predicted | 0.016 | RS-51 | MTAP | Predicted | 0.218 |
| RAB-27 | MMP9 | Predicted | 0.016 | RS-51 | NME1 | Predicted | 0.001 |
| RAB-27 | NME1 | Predicted | 0.033 | RS-51 | NME2 | Predicted | 0.001 |
| RAB-27 | NME2 | Predicted | 0.007 | RS-51 | NOS2A | Predicted | 0.215 |

| RAB-27 | NOS2A | Predicted | 0.025 | RS-51 | PTGS2 | Predicted | 0.356 |
| --- | --- | --- | --- | --- | --- | --- | --- |
| RAB-27 | PGK1 | Predicted | 0.008 | RS-51 | RHOA | Predicted | 0.001 |
| RAB-27 | PTGS2 | Predicted | 0.061 | RS-51 | SRC | Predicted | 0.103 |
| RAB-27 | PTK2 | Predicted | 0.008 | RS-51 | TGM2 | Predicted | 0.001 |
| RAB-27 | RHOA | Predicted | 0.007 | RS-51 | TOP2A | Predicted | 0.041 |
| RAB-27 | RND3 | Predicted | 0.008 | RS-52 | ACSL1 | Predicted | 0.025 |
| RAB-27 | SERPINE1 | Predicted | 0.018 | RS-52 | ALDH2 | Predicted | 0.087 |
| RAB-27 | SGK1 | Predicted | 0.005 | RS-52 | ALDOA | Predicted | 0.085 |
| RAB-27 | SRC | Predicted | 0.185 | RS-52 | CA1 | Predicted | 0.126 |
| RAB-27 | SULT2A1 | Predicted | 0.014 | RS-52 | CA2 | Predicted | 0.093 |
| RAB-27 | TGM2 | Predicted | 0.007 | RS-52 | CCNA2 | Predicted | 0.355 |
| RAB-27 | TOP2A | Predicted | 0.154 | RS-52 | ESR1 | Predicted | 0.197 |
| RAB-27 | TRDMT1 | Predicted | 0.005 | RS-52 | GABRE | Predicted | 0.009 |
| RAB-27 | VEGFA | Predicted | 0.029 | RS-52 | GSTP1 | Predicted | 0.000 |
| RAB-28 | ABCB1 | Predicted | 0.018 | RS-52 | KRAS | Predicted | 0.016 |
| RAB-28 | ACSL1 | Predicted | 0.018 | RS-52 | MMP12 | Predicted | 0.025 |
| RAB-28 | ADH1C | Predicted | 0.012 | RS-52 | MMP3 | Predicted | 0.025 |
| RAB-28 | AGXT | Predicted | 0.013 | RS-52 | MTAP | Predicted | 0.179 |
| RAB-28 | AKT2 | Predicted | 0.387 | RS-52 | NOS2A | Predicted | 0.088 |
| RAB-28 | ASS1 | Predicted | 0.018 | RS-52 | PTGS2 | Predicted | 0.810 |
| RAB-28 | CA1 | Predicted | 0.058 | RS-53 | ALDH2 | Predicted | 0.067 |
| RAB-28 | CA2 | Predicted | 0.058 | RS-53 | ALDOA | Predicted | 0.067 |
| RAB-28 | CASP1 | Predicted | 0.037 | RS-53 | AR | Predicted | 0.090 |
| RAB-28 | CCNA2 | Predicted | 0.777 | RS-53 | CA1 | Predicted | 0.173 |
| RAB-28 | CDK4 | Predicted | 0.047 | RS-53 | CA2 | Predicted | 0.103 |

| RAB-28 | CES1 | Predicted | 0.036 | RS-53 | CCNA2 | Predicted | 0.068 |
| --- | --- | --- | --- | --- | --- | --- | --- |
| RAB-28 | CTSB | Predicted | 0.194 | RS-53 | ESR1 | Predicted | 0.308 |
| RAB-28 | ESR1 | Predicted | 0.395 | RS-53 | FGF1 | Predicted | 0.011 |
| RAB-28 | FGF1 | Predicted | 0.095 | RS-53 | FGF2 | Predicted | 0.011 |
| RAB-28 | FGF2 | Predicted | 0.095 | RS-53 | GABRE | Predicted | 0.012 |
| RAB-28 | GABRE | Predicted | 0.005 | RS-53 | GSTP1 | Predicted | 0.000 |
| RAB-28 | GAPDH | Predicted | 0.040 | RS-53 | HGF | Predicted | 0.011 |
| RAB-28 | GLUD1 | Predicted | 0.038 | RS-53 | KRAS | Predicted | 0.006 |
| RAB-28 | GNMT | Predicted | 0.008 | RS-53 | LCN2 | Predicted | 0.066 |
| RAB-28 | GSTP1 | Predicted | 0.044 | RS-53 | MAPK1 | Predicted | 0.026 |
| RAB-28 | HAO1 | Predicted | 0.000 | RS-53 | MTAP | Predicted | 0.138 |
| RAB-28 | KRAS | Predicted | 0.126 | RS-53 | NOS2A | Predicted | 0.138 |
| RAB-28 | LYZ | Predicted | 0.090 | RS-53 | PARP1 | Predicted | 0.066 |
| RAB-28 | MAPK1 | Predicted | 0.088 | RS-53 | PC | Predicted | 0.025 |
| RAB-28 | MAPK3 | Predicted | 0.047 | RS-53 | PTGS2 | Predicted | 1.000 |
| RAB-28 | MET | Predicted | 0.188 | RS-53 | SRC | Predicted | 0.020 |
| RAB-28 | MME | Predicted | 0.129 | RS-53 | SULT2A1 | Predicted | 0.014 |
| RAB-28 | MMP12 | Predicted | 0.165 | RS-53 | TNFSF11 | Predicted | 0.020 |
| RAB-28 | MMP9 | Predicted | 0.037 | RS-53 | TOP2A | Predicted | 0.065 |
| RAB-28 | NME1 | Predicted | 0.188 | RS-55 | ACSL1 | Predicted | 0.028 |
| RAB-28 | NOS2A | Predicted | 0.038 | RS-55 | ALDH2 | Predicted | 0.091 |
| RAB-28 | PTGS2 | Predicted | 0.078 | RS-55 | ALDOA | Predicted | 0.093 |
| RAB-28 | RND3 | Predicted | 0.038 | RS-55 | AR | Predicted | 0.055 |
| RAB-28 | SGK1 | Predicted | 0.000 | RS-55 | CA1 | Predicted | 0.104 |
| RAB-28 | SRC | Predicted | 0.093 | RS-55 | CA2 | Predicted | 0.050 |

| RAB-28 | SULT2A1 | Predicted | 0.035 | RS-55 | CCNA2 | Predicted | 0.187 |
| --- | --- | --- | --- | --- | --- | --- | --- |
| RAB-28 | TOP2A | Predicted | 0.067 | RS-55 | ESR1 | Predicted | 0.464 |
| RAB-28 | TRDMT1 | Predicted | 0.008 | RS-55 | FGF1 | Predicted | 0.014 |
| RAB-28 | VEGFA | Predicted | 0.081 | RS-55 | FGF2 | Predicted | 0.014 |
| RAB-29 | ABCB1 | Predicted | 0.004 | RS-55 | GABRE | Predicted | 0.017 |
| RAB-29 | ACSL1 | Predicted | 0.004 | RS-55 | GSTP1 | Predicted | 0.000 |
| RAB-29 | AKT2 | Predicted | 0.033 | RS-55 | HGF | Predicted | 0.014 |
| RAB-29 | ALDH1L1 | Predicted | 0.011 | RS-55 | KRAS | Predicted | 0.008 |
| RAB-29 | ASS1 | Predicted | 0.004 | RS-55 | LCN2 | Predicted | 0.090 |
| RAB-29 | AURKA | Predicted | 0.003 | RS-55 | MMP12 | Predicted | 0.027 |
| RAB-29 | CA1 | Predicted | 0.020 | RS-55 | MMP3 | Predicted | 0.027 |
| RAB-29 | CA2 | Predicted | 0.020 | RS-55 | MTAP | Predicted | 0.186 |
| RAB-29 | CCNA2 | Predicted | 1.000 | RS-55 | NOS2A | Predicted | 0.278 |
| RAB-29 | CCT3 | Predicted | 0.010 | RS-55 | PC | Predicted | 0.034 |
| RAB-29 | CYP2C8 | Predicted | 0.007 | RS-55 | PTGS2 | Predicted | 1.000 |
| RAB-29 | ESR1 | Predicted | 0.124 | RS-56 | ACSL1 | Predicted | 0.026 |
| RAB-29 | FTCD | Predicted | 0.011 | RS-56 | ALDOA | Predicted | 0.131 |
| RAB-29 | GLUD1 | Predicted | 0.016 | RS-56 | AURKA | Predicted | 0.012 |
| RAB-29 | GNMT | Predicted | 0.002 | RS-56 | CA1 | Predicted | 0.212 |
| RAB-29 | GSTP1 | Predicted | 0.102 | RS-56 | CA2 | Predicted | 0.168 |
| RAB-29 | HSP90AA1 | Predicted | 0.003 | RS-56 | CCNA2 | Predicted | 0.846 |
| RAB-29 | HSP90B1 | Predicted | 0.003 | RS-56 | CCT3 | Predicted | 0.001 |
| RAB-29 | HSPA1B | Predicted | 0.003 | RS-56 | CTSB | Predicted | 0.130 |
| RAB-29 | HSPA8 | Predicted | 0.003 | RS-56 | ESR1 | Predicted | 0.272 |
| RAB-29 | KRAS | Predicted | 0.022 | RS-56 | GABRE | Predicted | 0.022 |

| RAB-29 | LYZ | Predicted | 0.118 | RS-56 | GLUD1 | Predicted | 0.001 |
| --- | --- | --- | --- | --- | --- | --- | --- |
| RAB-29 | MAPK1 | Predicted | 0.048 | RS-56 | GNAI1 | Predicted | 0.002 |
| RAB-29 | MAT1A | Predicted | 0.003 | RS-56 | GSTP1 | Predicted | 0.000 |
| RAB-29 | MME | Predicted | 0.120 | RS-56 | HSP90AA1 | Predicted | 0.001 |
| RAB-29 | MTHFD1 | Predicted | 0.011 | RS-56 | HSP90B1 | Predicted | 0.001 |
| RAB-29 | MTR | Predicted | 0.011 | RS-56 | HSPA1B | Predicted | 0.001 |
| RAB-29 | NME1 | Predicted | 0.010 | RS-56 | HSPA8 | Predicted | 0.001 |
| RAB-29 | PGK1 | Predicted | 0.003 | RS-56 | KRAS | Predicted | 0.044 |
| RAB-29 | PTGS2 | Predicted | 0.051 | RS-56 | MAPK3 | Predicted | 0.085 |
| RAB-29 | PTK2 | Predicted | 0.003 | RS-56 | MAT1A | Predicted | 0.001 |
| RAB-29 | RARA | Predicted | 0.051 | RS-56 | MTAP | Predicted | 0.282 |
| RAB-29 | RND3 | Predicted | 0.008 | RS-56 | NME1 | Predicted | 0.014 |
| RAB-29 | SERPINE1 | Predicted | 0.026 | RS-56 | NME2 | Predicted | 0.002 |
| RAB-29 | TRDMT1 | Predicted | 0.002 | RS-56 | NOS2A | Predicted | 0.279 |
| RAB-29 | VEGFA | Predicted | 0.032 | RS-56 | PGK1 | Predicted | 0.001 |
| RAB-30 | ABCB1 | Predicted | 0.018 | RS-56 | PTGS2 | Predicted | 0.474 |
| RAB-30 | ACSL1 | Predicted | 0.018 | RS-56 | PTK2 | Predicted | 0.001 |
| RAB-30 | ADH1C | Predicted | 0.012 | RS-56 | RHOA | Predicted | 0.002 |
| RAB-30 | AGXT | Predicted | 0.013 | RS-56 | SRC | Predicted | 0.132 |
| RAB-30 | AKT2 | Predicted | 0.387 | RS-56 | SULT2A1 | Predicted | 0.007 |
| RAB-30 | ASS1 | Predicted | 0.018 | RS-56 | TGM2 | Predicted | 0.002 |
| RAB-30 | CA1 | Predicted | 0.058 | RS-56 | TOP2A | Predicted | 0.052 |
| RAB-30 | CA2 | Predicted | 0.058 | RS-58 | ACSL1 | Predicted | 0.018 |
| RAB-30 | CASP1 | Predicted | 0.037 | RS-58 | ALDOA | Predicted | 0.083 |
| RAB-30 | CCNA2 | Predicted | 0.777 | RS-58 | CA1 | Predicted | 0.159 |

| RAB-30 | CDK4 | Predicted | 0.047 | RS-58 | CA2 | Predicted | 0.048 |
| --- | --- | --- | --- | --- | --- | --- | --- |
| RAB-30 | CES1 | Predicted | 0.036 | RS-58 | CCNA2 | Predicted | 0.801 |
| RAB-30 | CTSB | Predicted | 0.194 | RS-58 | CDKN1A | Validated | 1.000 |
| RAB-30 | ESR1 | Predicted | 0.395 | RS-58 | ESR1 | Predicted | 0.126 |
| RAB-30 | FGF1 | Predicted | 0.095 | RS-58 | GSTP1 | Predicted | 0.000 |
| RAB-30 | FGF2 | Predicted | 0.095 | RS-58 | KRAS | Predicted | 0.013 |
| RAB-30 | GABRE | Predicted | 0.005 | RS-58 | LCN2 | Predicted | 0.084 |
| RAB-30 | GAPDH | Predicted | 0.040 | RS-58 | LYZ | Predicted | 0.083 |
| RAB-30 | GLUD1 | Predicted | 0.038 | RS-58 | MAPK3 | Predicted | 0.043 |
| RAB-30 | GNMT | Predicted | 0.008 | RS-58 | MTAP | Predicted | 0.174 |
| RAB-30 | GSTP1 | Predicted | 0.044 | RS-58 | NOS2A | Validated | 1.000 |
| RAB-30 | HAO1 | Predicted | 0.000 | RS-58 | NOS2A | Predicted | 0.181 |
| RAB-30 | KRAS | Predicted | 0.126 | RS-58 | PTGS2 | Predicted | 0.318 |
| RAB-30 | LYZ | Predicted | 0.090 | RS-58 | SRC | Predicted | 0.082 |
| RAB-30 | MAPK1 | Predicted | 0.088 | RS-58 | SULT2A1 | Predicted | 0.008 |
| RAB-30 | MAPK3 | Predicted | 0.047 | RS-58 | TOP2A | Predicted | 0.028 |
| RAB-30 | MET | Predicted | 0.188 | RS-59 | ALDH2 | Predicted | 0.085 |
| RAB-30 | MME | Predicted | 0.129 | RS-59 | ALDOA | Predicted | 0.086 |
| RAB-30 | MMP12 | Predicted | 0.165 | RS-59 | AR | Predicted | 0.083 |
| RAB-30 | MMP9 | Predicted | 0.037 | RS-59 | ARG1 | Predicted | 0.041 |
| RAB-30 | NME1 | Predicted | 0.188 | RS-59 | B2M | Predicted | 0.017 |
| RAB-30 | NOS2A | Predicted | 0.038 | RS-59 | CA1 | Predicted | 0.246 |
| RAB-30 | PTGS2 | Predicted | 0.078 | RS-59 | CA2 | Predicted | 0.163 |
| RAB-30 | RND3 | Predicted | 0.038 | RS-59 | CCNA2 | Predicted | 0.257 |
| RAB-30 | SGK1 | Predicted | 0.000 | RS-59 | ESR1 | Predicted | 0.499 |

| RAB-31 | SRC | Predicted | 0.128 | RS-59 | FGF1 | Predicted | 0.013 |
| --- | --- | --- | --- | --- | --- | --- | --- |
| RAB-31 | TOP2A | Predicted | 0.061 | RS-59 | FGF2 | Predicted | 0.013 |
| RAB-31 | TRDMT1 | Predicted | 0.004 | RS-59 | GABRE | Predicted | 0.008 |
| RAB-31 | VEGFA | Predicted | 0.016 | RS-59 | GSTP1 | Predicted | 0.000 |
| RAB-32 | CASP3 | Validated | 1.000 | RS-59 | HGF | Predicted | 0.013 |
| RAB-32 | ABCB1 | Predicted | 0.014 | RS-59 | KRAS | Predicted | 0.007 |
| RAB-32 | ACSL1 | Predicted | 0.014 | RS-59 | LCN2 | Predicted | 0.086 |
| RAB-32 | ADH1B | Predicted | 0.009 | RS-59 | MAPK1 | Predicted | 0.033 |
| RAB-32 | ADH1C | Predicted | 0.019 | RS-59 | MTAP | Predicted | 0.172 |
| RAB-32 | ADH4 | Predicted | 0.009 | RS-59 | NOS2A | Predicted | 0.259 |
| RAB-32 | AKR1C2 | Predicted | 0.001 | RS-59 | PC | Predicted | 0.007 |
| RAB-32 | AKT2 | Predicted | 0.105 | RS-59 | PTGS2 | Predicted | 0.963 |
| RAB-32 | ALDH1A1 | Predicted | 0.009 | RS-59 | SRC | Predicted | 0.023 |
| RAB-32 | ALDH1B1 | Predicted | 0.001 | RS-59 | TNFSF11 | Predicted | 0.025 |
| RAB-32 | ALDH1L1 | Predicted | 0.000 | RS-59 | TOP2A | Predicted | 0.039 |
| RAB-32 | ALDH2 | Predicted | 0.009 | RS-60 | ACSL1 | Predicted | 0.024 |
| RAB-32 | ALDH3A2 | Predicted | 0.001 | RS-60 | ALDH2 | Predicted | 0.085 |
| RAB-32 | ALDH4A1 | Predicted | 0.001 | RS-60 | ALDOA | Predicted | 0.086 |
| RAB-32 | ASS1 | Predicted | 0.014 | RS-60 | AR | Predicted | 0.111 |
| RAB-32 | AURKA | Predicted | 0.006 | RS-60 | CA1 | Predicted | 0.219 |
| RAB-32 | CASP1 | Predicted | 0.034 | RS-60 | CA2 | Predicted | 0.130 |
| RAB-32 | CCNA2 | Predicted | 0.283 | RS-60 | CCNA2 | Predicted | 0.257 |
| RAB-32 | CCT3 | Predicted | 0.021 | RS-60 | ESR1 | Predicted | 0.661 |
| RAB-32 | CDK4 | Predicted | 0.043 | RS-60 | FGF1 | Predicted | 0.013 |
| RAB-32 | CES1 | Predicted | 0.003 | RS-60 | FGF2 | Predicted | 0.013 |

| RAB-32 | ESR1 | Predicted | 0.136 | RS-60 | GABRE | Predicted | 0.022 |
| --- | --- | --- | --- | --- | --- | --- | --- |
| RAB-32 | FTCD | Predicted | 0.000 | RS-60 | GSTP1 | Predicted | 0.000 |
| RAB-32 | GAPDH | Predicted | 0.029 | RS-60 | HGF | Predicted | 0.013 |
| RAB-32 | GLUD1 | Predicted | 0.034 | RS-60 | KRAS | Predicted | 0.007 |
| RAB-32 | GNAI1 | Predicted | 0.004 | RS-60 | LCN2 | Predicted | 0.085 |
| RAB-32 | GNMT | Predicted | 0.004 | RS-60 | MAPK1 | Predicted | 0.033 |
| RAB-32 | GSTP1 | Predicted | 0.115 | RS-60 | MTAP | Predicted | 0.175 |
| RAB-32 | HAO1 | Predicted | 0.003 | RS-60 | NOS2A | Predicted | 0.174 |
| RAB-32 | HSD17B10 | Predicted | 0.009 | RS-60 | PC | Predicted | 0.007 |
| RAB-32 | HSD17B4 | Predicted | 0.009 | RS-60 | PTGS2 | Predicted | 0.820 |
| RAB-32 | HSP90AA1 | Predicted | 0.006 | RS-60 | SRC | Predicted | 0.024 |
| RAB-32 | HSP90B1 | Predicted | 0.006 | RS-60 | TNFSF11 | Predicted | 0.025 |
| RAB-32 | HSPA1B | Predicted | 0.006 | RS-60 | TOP2A | Predicted | 0.055 |
| RAB-32 | HSPA8 | Predicted | 0.006 | RS-61 | ACSL1 | Predicted | 0.033 |
| RAB-32 | KRAS | Predicted | 0.083 | RS-61 | ALDH2 | Predicted | 0.137 |
| RAB-32 | MAPK1 | Predicted | 0.120 | RS-61 | AURKA | Predicted | 0.137 |
| RAB-32 | MAPK3 | Predicted | 0.043 | RS-61 | CA1 | Predicted | 0.297 |
| RAB-32 | MAT1A | Predicted | 0.006 | RS-61 | CA2 | Predicted | 0.196 |
| RAB-32 | MET | Predicted | 0.134 | RS-61 | CCNA2 | Predicted | 0.861 |
| RAB-32 | MME | Predicted | 0.209 | RS-61 | ESR1 | Predicted | 0.278 |
| RAB-32 | MMP12 | Predicted | 0.054 | RS-61 | GSTP1 | Predicted | 0.000 |
| RAB-32 | MMP9 | Predicted | 0.034 | RS-61 | KRAS | Predicted | 0.011 |
| RAB-32 | MTHFD1 | Predicted | 0.008 | RS-61 | MTAP | Predicted | 0.283 |
| RAB-32 | MTR | Predicted | 0.000 | RS-61 | NOS2A | Predicted | 0.284 |
| RAB-32 | NME1 | Predicted | 0.028 | RS-61 | PTGS2 | Predicted | 0.719 |

| RAB-32 | NME2 | Predicted | 0.004 | RS-61 | SRC | Predicted | 0.137 |
| --- | --- | --- | --- | --- | --- | --- | --- |
| RAB-32 | PGK1 | Predicted | 0.006 | RS-61 | TOP2A | Predicted | 0.057 |
| RAB-32 | PTK2 | Predicted | 0.006 | RS-62 | ACSL1 | Predicted | 0.023 |
| RAB-32 | QDPR | Predicted | 0.009 | RS-62 | ALDH2 | Predicted | 0.083 |
| RAB-32 | RHOA | Predicted | 0.004 | RS-62 | ALDOA | Predicted | 0.086 |
| RAB-32 | RND3 | Predicted | 0.005 | RS-62 | CA1 | Predicted | 0.176 |
| RAB-32 | SERPINE1 | Predicted | 0.009 | RS-62 | CA2 | Predicted | 0.086 |
| RAB-32 | SGK1 | Predicted | 0.003 | RS-62 | CCNA2 | Predicted | 0.348 |
| RAB-32 | SRC | Predicted | 0.065 | RS-62 | CTSB | Predicted | 0.082 |
| RAB-32 | TGM2 | Predicted | 0.004 | RS-62 | ESR1 | Predicted | 0.421 |
| RAB-32 | TOP2A | Predicted | 0.201 | RS-62 | GABRE | Predicted | 0.010 |
| RAB-32 | TRDMT1 | Predicted | 0.004 | RS-62 | GSTP1 | Predicted | 0.000 |
| RAB-32 | TUBB | Predicted | 0.006 | RS-62 | KRAS | Predicted | 0.007 |
| RAB-32 | VEGFA | Predicted | 0.065 | RS-62 | LCN2 | Predicted | 0.087 |
| RAB-33 | ABCB1 | Predicted | 0.004 | RS-62 | MTAP | Predicted | 0.177 |
| RAB-33 | ACSL1 | Predicted | 0.004 | RS-62 | NOS2A | Predicted | 0.264 |
| RAB-33 | AKT2 | Predicted | 0.156 | RS-62 | PTGS2 | Predicted | 0.751 |
| RAB-33 | ASS1 | Predicted | 0.004 | RS-62 | TOP2A | Predicted | 0.056 |
| RAB-33 | CCNA2 | Predicted | 1.000 | RS-63 | ACSL1 | Predicted | 0.022 |
| RAB-33 | CCT3 | Predicted | 0.007 | RS-63 | ALDH2 | Predicted | 0.082 |
| RAB-33 | CDK4 | Predicted | 0.036 | RS-63 | ALDOA | Predicted | 0.079 |
| RAB-33 | CTSB | Predicted | 0.095 | RS-63 | AR | Predicted | 0.081 |
| RAB-33 | EGFR | Predicted | 0.093 | RS-63 | B2M | Predicted | 0.019 |
| RAB-33 | ESR1 | Predicted | 0.093 | RS-63 | CA1 | Predicted | 0.191 |
| RAB-33 | GABRE | Predicted | 0.000 | RS-63 | CA2 | Predicted | 0.191 |

| RAB-33 | GAPDH | Predicted | 0.005 | RS-63 | CCNA2 | Predicted | 0.081 |
| --- | --- | --- | --- | --- | --- | --- | --- |
| RAB-33 | GLUD1 | Predicted | 0.010 | RS-63 | ESR1 | Predicted | 0.321 |
| RAB-33 | GNMT | Predicted | 0.004 | RS-63 | GABRE | Predicted | 0.015 |
| RAB-33 | GSTP1 | Predicted | 0.028 | RS-63 | GSTP1 | Predicted | 0.000 |
| RAB-33 | HAO1 | Predicted | 0.000 | RS-63 | KRAS | Predicted | 0.008 |
| RAB-33 | KRAS | Predicted | 0.057 | RS-63 | LCN2 | Predicted | 0.080 |
| RAB-33 | MAPK1 | Predicted | 0.101 | RS-63 | MAPK1 | Predicted | 0.027 |
| RAB-33 | MAPK3 | Predicted | 0.036 | RS-63 | MTAP | Predicted | 0.163 |
| RAB-33 | MME | Predicted | 0.147 | RS-63 | NOS2A | Predicted | 0.205 |
| RAB-33 | MMP12 | Predicted | 0.036 | RS-63 | PARP1 | Predicted | 0.080 |
| RAB-33 | NOS2A | Predicted | 0.096 | RS-63 | PC | Predicted | 0.030 |
| RAB-33 | PTGS2 | Predicted | 0.026 | RS-63 | PTGS2 | Predicted | 1.000 |
| RAB-33 | RND3 | Predicted | 0.010 | RS-63 | SRC | Predicted | 0.024 |
| RAB-33 | SERPINE1 | Predicted | 0.024 | RS-63 | TNFSF11 | Predicted | 0.026 |
| RAB-33 | SGK1 | Predicted | 0.000 | RS-64 | ALDH2 | Predicted | 0.063 |
| RAB-33 | SRC | Predicted | 0.128 | RS-64 | ALDOA | Predicted | 0.063 |
| RAB-33 | TOP2A | Predicted | 0.061 | RS-64 | AR | Predicted | 0.085 |
| RAB-33 | TRDMT1 | Predicted | 0.004 | RS-64 | ARG1 | Predicted | 0.028 |
| RAB-33 | VEGFA | Predicted | 0.016 | RS-64 | B2M | Predicted | 0.013 |
| RAB-34 | ABCB1 | Predicted | 0.014 | RS-64 | CA1 | Predicted | 0.120 |
| RAB-34 | ACSL1 | Predicted | 0.014 | RS-64 | CA2 | Predicted | 0.089 |
| RAB-34 | ADH1B | Predicted | 0.009 | RS-64 | CCNA2 | Predicted | 0.064 |
| RAB-34 | ADH1C | Predicted | 0.019 | RS-64 | ESR1 | Predicted | 0.339 |
| RAB-34 | ADH4 | Predicted | 0.009 | RS-64 | FGF1 | Predicted | 0.010 |
| RAB-34 | AKR1C2 | Predicted | 0.001 | RS-64 | FGF2 | Predicted | 0.010 |

| RAB-34 | AKT2 | Predicted | 0.105 | RS-64 | GABRE | Predicted | 0.005 |
| --- | --- | --- | --- | --- | --- | --- | --- |
| RAB-34 | ALDH1A1 | Predicted | 0.009 | RS-64 | GSTP1 | Predicted | 0.000 |
| RAB-34 | ALDH1B1 | Predicted | 0.001 | RS-64 | HGF | Predicted | 0.010 |
| RAB-34 | ALDH1L1 | Predicted | 0.000 | RS-64 | KRAS | Predicted | 0.005 |
| RAB-34 | ALDH2 | Predicted | 0.009 | RS-64 | LCN2 | Predicted | 0.063 |
| RAB-34 | ALDH3A2 | Predicted | 0.001 | RS-64 | MAPK1 | Predicted | 0.022 |
| RAB-34 | ALDH4A1 | Predicted | 0.001 | RS-64 | MTAP | Predicted | 0.128 |
| RAB-34 | ASS1 | Predicted | 0.014 | RS-64 | NOS2A | Predicted | 0.181 |
| RAB-34 | AURKA | Predicted | 0.006 | RS-64 | PARP1 | Predicted | 0.061 |
| RAB-34 | CASP1 | Predicted | 0.034 | RS-64 | PC | Predicted | 0.023 |
| RAB-34 | CCNA2 | Predicted | 0.283 | RS-64 | PTGS2 | Predicted | 1.000 |
| RAB-34 | CCT3 | Predicted | 0.021 | RS-64 | SRC | Predicted | 0.019 |
| RAB-34 | CDK4 | Predicted | 0.043 | RS-64 | SULT2A1 | Predicted | 0.013 |
| RAB-34 | CES1 | Predicted | 0.003 | RS-64 | TNFSF11 | Predicted | 0.019 |
| RAB-34 | ESR1 | Predicted | 0.136 | RS-64 | TOP2A | Predicted | 0.062 |
| RAB-34 | FTCD | Predicted | 0.000 | RS-65 | ACSL1 | Predicted | 0.024 |
| RAB-34 | GAPDH | Predicted | 0.029 | RS-65 | ALDH2 | Predicted | 0.081 |
| RAB-34 | GLUD1 | Predicted | 0.034 | RS-65 | ALDOA | Predicted | 0.082 |
| RAB-34 | GNAI1 | Predicted | 0.004 | RS-65 | AR | Predicted | 0.081 |
| RAB-34 | GNMT | Predicted | 0.004 | RS-65 | B2M | Predicted | 0.017 |
| RAB-34 | GSTP1 | Predicted | 0.115 | RS-65 | CA1 | Predicted | 0.182 |
| RAB-34 | HAO1 | Predicted | 0.003 | RS-65 | CA2 | Predicted | 0.204 |
| RAB-34 | HSD17B10 | Predicted | 0.009 | RS-65 | CCNA2 | Predicted | 0.170 |
| RAB-34 | HSD17B4 | Predicted | 0.009 | RS-65 | CDKN1A | Validated | 1.000 |
| RAB-34 | HSP90AA1 | Predicted | 0.006 | RS-65 | ESR1 | Predicted | 0.114 |

| RAB-34 | HSP90B1 | Predicted | 0.006 | RS-65 | FN1 | Validated | 1.000 |
| --- | --- | --- | --- | --- | --- | --- | --- |
| RAB-34 | HSPA1B | Predicted | 0.006 | RS-65 | GABRE | Predicted | 0.009 |
| RAB-34 | HSPA8 | Predicted | 0.006 | RS-65 | GSTP1 | Predicted | 0.000 |
| RAB-34 | KRAS | Predicted | 0.083 | RS-65 | KRAS | Predicted | 0.007 |
| RAB-34 | MAPK1 | Predicted | 0.120 | RS-65 | LCN2 | Predicted | 0.081 |
| RAB-34 | MAPK3 | Predicted | 0.043 | RS-65 | MAPK1 | Predicted | 0.027 |
| RAB-34 | MAT1A | Predicted | 0.006 | RS-65 | MCL1 | Validated | 1.000 |
| RAB-34 | MET | Predicted | 0.134 | RS-65 | MMP12 | Predicted | 0.024 |
| RAB-34 | MME | Predicted | 0.209 | RS-65 | MMP3 | Predicted | 0.024 |
| RAB-34 | MMP12 | Predicted | 0.054 | RS-65 | MTAP | Predicted | 0.174 |
| RAB-34 | MMP9 | Predicted | 0.034 | RS-65 | NOS2A | Validated | 1.000 |
| RAB-34 | MTHFD1 | Predicted | 0.008 | RS-65 | PARP1 | Predicted | 0.083 |
| RAB-34 | MTR | Predicted | 0.000 | RS-65 | PC | Predicted | 0.006 |
| RAB-34 | NME1 | Predicted | 0.028 | RS-65 | PTGS2 | Predicted | 1.000 |
| RAB-34 | NME2 | Predicted | 0.004 | RS-65 | SRC | Predicted | 0.022 |
| RAB-34 | PGK1 | Predicted | 0.006 | RS-65 | TNFSF11 | Predicted | 0.024 |
| RAB-34 | PTK2 | Predicted | 0.006 | RS-66 | ACSL1 | Predicted | 0.022 |
| RAB-34 | QDPR | Predicted | 0.009 | RS-66 | AGXT | Predicted | 0.003 |
| RAB-34 | RHOA | Predicted | 0.004 | RS-66 | AKR1B10 | Predicted | 0.101 |
| RAB-34 | RND3 | Predicted | 0.005 | RS-66 | ALDOA | Predicted | 0.103 |
| RAB-34 | SERPINE1 | Predicted | 0.009 | RS-66 | AURKA | Predicted | 0.104 |
| RAB-34 | SGK1 | Predicted | 0.003 | RS-66 | CA1 | Predicted | 0.166 |
| RAB-34 | SRC | Predicted | 0.065 | RS-66 | CA2 | Predicted | 0.131 |
| RAB-34 | TGM2 | Predicted | 0.004 | RS-66 | CCNA2 | Validated | 1.000 |
| RAB-34 | TOP2A | Predicted | 0.201 | RS-66 | CTSB | Predicted | 0.102 |

| RAB-34 | TRDMT1 | Predicted | 0.004 | RS-66 | CYP2C9 | Validated | 1.000 |
| --- | --- | --- | --- | --- | --- | --- | --- |
| RAB-34 | TUBB | Predicted | 0.006 | RS-66 | ESR1 | Predicted | 0.161 |
| RAB-34 | VEGFA | Predicted | 0.065 | RS-66 | GABRE | Predicted | 0.007 |
| RAB-35 | ABCB1 | Predicted | 0.013 | RS-66 | GNAI1 | Predicted | 0.001 |
| RAB-35 | ACSL1 | Predicted | 0.013 | RS-66 | GSTP1 | Predicted | 0.000 |
| RAB-35 | AKT2 | Predicted | 0.047 | RS-66 | KRAS | Predicted | 0.017 |
| RAB-35 | ALDH1L1 | Predicted | 0.016 | RS-66 | LCN2 | Predicted | 0.100 |
| RAB-35 | ASS1 | Predicted | 0.013 | RS-66 | MAPK3 | Predicted | 0.053 |
| RAB-35 | AURKA | Predicted | 0.129 | RS-66 | MTAP | Predicted | 0.221 |
| RAB-35 | CCNA2 | Predicted | 0.944 | RS-66 | NME1 | Predicted | 0.001 |
| RAB-35 | CCT3 | Predicted | 0.007 | RS-66 | NME2 | Predicted | 0.001 |
| RAB-35 | CYP2C8 | Predicted | 0.017 | RS-66 | NOS2A | Predicted | 0.218 |
| RAB-35 | EGFR | Predicted | 0.129 | RS-66 | PTGS2 | Predicted | 0.392 |
| RAB-35 | ESR1 | Predicted | 0.040 | RS-66 | RHOA | Predicted | 0.001 |
| RAB-35 | FTCD | Predicted | 0.016 | RS-66 | SRC | Predicted | 0.102 |
| RAB-35 | GLUD1 | Predicted | 0.014 | RS-66 | TGM2 | Predicted | 0.001 |
| RAB-35 | GNMT | Predicted | 0.005 | RS-66 | TOP2A | Predicted | 0.039 |
| RAB-35 | GSTP1 | Predicted | 0.024 | RS-67 | ACSL1 | Predicted | 0.017 |
| RAB-35 | KRAS | Predicted | 0.030 | RS-67 | ALDH2 | Predicted | 0.080 |
| RAB-35 | MME | Predicted | 0.131 | RS-67 | ALDOA | Predicted | 0.085 |
| RAB-35 | MTHFD1 | Predicted | 0.016 | RS-67 | AURKA | Predicted | 0.080 |
| RAB-35 | MTR | Predicted | 0.016 | RS-67 | CA1 | Predicted | 0.180 |
| RAB-35 | NME1 | Predicted | 0.134 | RS-67 | CA2 | Predicted | 0.106 |
| RAB-35 | NOS2A | Predicted | 0.129 | RS-67 | CCNA2 | Predicted | 0.523 |
| RAB-35 | PTGS2 | Predicted | 0.029 | RS-67 | CTSB | Predicted | 0.081 |

| RAB-35 | RARA | Predicted | 0.017 | RS-67 | ESR1 | Predicted | 0.224 |
| --- | --- | --- | --- | --- | --- | --- | --- |
| RAB-35 | RND3 | Predicted | 0.014 | RS-67 | GABRE | Predicted | 0.008 |
| RAB-35 | SERPINE1 | Predicted | 0.028 | RS-67 | GSTP1 | Predicted | 0.000 |
| RAB-35 | TRDMT1 | Predicted | 0.005 | RS-67 | KRAS | Predicted | 0.005 |
| RAB-35 | VEGFA | Predicted | 0.031 | RS-67 | LCN2 | Predicted | 0.084 |
| RAB-36 | ABCB1 | Predicted | 0.010 | RS-67 | MAPK3 | Predicted | 0.049 |
| RAB-36 | ACSL1 | Predicted | 0.010 | RS-67 | MTAP | Predicted | 0.177 |
| RAB-36 | ADH1B | Predicted | 0.012 | RS-67 | NOS2A | Predicted | 0.179 |
| RAB-36 | ADH1C | Predicted | 0.006 | RS-67 | PTGS2 | Predicted | 0.418 |
| RAB-36 | ADH4 | Predicted | 0.006 | RS-67 | SRC | Predicted | 0.080 |
| RAB-36 | AGXT | Predicted | 0.014 | RS-67 | SULT2A1 | Predicted | 0.012 |
| RAB-36 | AKR1B10 | Predicted | 0.228 | RS-67 | TOP2A | Predicted | 0.035 |
| RAB-36 | AKR1C2 | Predicted | 0.006 | RS-68 | ACSL1 | Predicted | 0.026 |
| RAB-36 | AKT2 | Predicted | 0.334 | RS-68 | ALDH2 | Predicted | 0.087 |
| RAB-36 | ALDH1A1 | Predicted | 0.006 | RS-68 | ALDOA | Predicted | 0.087 |
| RAB-36 | ALDH1B1 | Predicted | 0.000 | RS-68 | CA1 | Predicted | 0.066 |
| RAB-36 | ALDH2 | Predicted | 0.006 | RS-68 | CA2 | Predicted | 0.031 |
| RAB-36 | ALDH3A2 | Predicted | 0.000 | RS-68 | CCNA2 | Predicted | 0.617 |
| RAB-36 | ALDH4A1 | Predicted | 0.000 | RS-68 | ESR1 | Predicted | 0.279 |
| RAB-36 | ASS1 | Predicted | 0.010 | RS-68 | GABRE | Predicted | 0.008 |
| RAB-36 | AURKA | Predicted | 0.011 | RS-68 | GSTP1 | Predicted | 0.000 |
| RAB-36 | CA1 | Predicted | 0.109 | RS-68 | KRAS | Predicted | 0.016 |
| RAB-36 | CA2 | Predicted | 0.109 | RS-68 | LCN2 | Predicted | 0.084 |
| RAB-36 | CASP1 | Predicted | 0.067 | RS-68 | MTAP | Predicted | 0.178 |
| RAB-36 | CCNA2 | Predicted | 0.943 | RS-68 | NOS2A | Predicted | 0.176 |

| RAB-36 | CCT3 | Predicted | 0.034 | RS-68 | PTGS2 | Predicted | 0.812 |
| --- | --- | --- | --- | --- | --- | --- | --- |
| RAB-36 | CDK4 | Predicted | 0.035 | RS-68 | TOP2A | Predicted | 0.036 |
| RAB-36 | CES1 | Predicted | 0.012 | RS-69 | EGFR | Predicted | 0.942 |
| RAB-36 | CTSB | Predicted | 0.236 | RS-69 | PTGS2 | Predicted | 0.033 |
| RAB-36 | DCXR | Predicted | 0.000 | RS-70 | EGFR | Predicted | 0.945 |
| RAB-36 | ESR1 | Predicted | 0.236 | RS-71 | ACSL1 | Validated | 1.000 |
| RAB-36 | GAPDH | Predicted | 0.006 | RS-71 | ALDH2 | Predicted | 0.149 |
| RAB-36 | GLUD1 | Predicted | 0.006 | RS-71 | ALDOA | Predicted | 0.139 |
| RAB-36 | GNAI1 | Predicted | 0.012 | RS-71 | AURKA | Predicted | 0.002 |
| RAB-36 | GNMT | Predicted | 0.007 | RS-71 | CA1 | Predicted | 0.168 |
| RAB-36 | GSTP1 | Predicted | 0.204 | RS-71 | CA2 | Predicted | 0.188 |
| RAB-36 | HAO1 | Predicted | 0.000 | RS-71 | CCNA2 | Predicted | 0.137 |
| RAB-36 | HSD17B10 | Predicted | 0.006 | RS-71 | CCT3 | Predicted | 0.002 |
| RAB-36 | HSD17B4 | Predicted | 0.006 | RS-71 | CES1 | Predicted | 0.018 |
| RAB-36 | HSP90AA1 | Predicted | 0.011 | RS-71 | COL1A1 | Validated | 1.000 |
| RAB-36 | HSP90B1 | Predicted | 0.011 | RS-71 | COL7A1 | Validated | 1.000 |
| RAB-36 | HSPA1B | Predicted | 0.011 | RS-71 | CTSB | Predicted | 0.135 |
| RAB-36 | HSPA8 | Predicted | 0.011 | RS-71 | ESR1 | Predicted | 0.709 |
| RAB-36 | KRAS | Predicted | 0.159 | RS-71 | FGF1 | Predicted | 0.022 |
| RAB-36 | MAPK1 | Predicted | 0.035 | RS-71 | FGF2 | Predicted | 0.022 |
| RAB-36 | MAPK3 | Predicted | 0.035 | RS-71 | FOS | Validated | 1.000 |
| RAB-36 | MAT1A | Predicted | 0.011 | RS-71 | GABRE | Predicted | 0.179 |
| RAB-36 | MME | Predicted | 0.199 | RS-71 | GLUD1 | Predicted | 0.002 |
| RAB-36 | MMP12 | Predicted | 0.048 | RS-71 | GLUL | Validated | 1.000 |
| RAB-36 | MMP9 | Predicted | 0.067 | RS-71 | GNAI1 | Predicted | 0.001 |

| RAB-36 | MTHFD1 | Predicted | 0.006 | RS-71 | GSTP1 | Predicted | 0.003 |
| --- | --- | --- | --- | --- | --- | --- | --- |
| RAB-36 | NME1 | Predicted | 0.056 | RS-71 | HGF | Predicted | 0.022 |
| RAB-36 | NME2 | Predicted | 0.012 | RS-71 | HSP90AA1 | Predicted | 0.002 |
| RAB-36 | PGK1 | Predicted | 0.011 | RS-71 | HSP90B1 | Predicted | 0.002 |
| RAB-36 | PLAU | Predicted | 0.132 | RS-71 | HSPA1B | Predicted | 0.002 |
| RAB-36 | PTK2 | Predicted | 0.011 | RS-71 | HSPA8 | Predicted | 0.002 |
| RAB-36 | QDPR | Predicted | 0.006 | RS-71 | KRAS | Predicted | 0.047 |
| RAB-36 | RHOA | Predicted | 0.012 | RS-71 | LYZ | Predicted | 0.006 |
| RAB-36 | RND3 | Predicted | 0.015 | RS-71 | MAT1A | Predicted | 0.002 |
| RAB-36 | SERPINE1 | Predicted | 0.018 | RS-71 | MMP14 | Predicted | 0.000 |
| RAB-36 | SGK1 | Predicted | 0.000 | RS-71 | MMP2 | Predicted | 0.000 |
| RAB-36 | SRC | Predicted | 0.228 | RS-71 | MMP3 | Predicted | 0.000 |
| RAB-36 | TGM2 | Predicted | 0.012 | RS-71 | MMP7 | Predicted | 0.000 |
| RAB-36 | TOP2A | Predicted | 0.143 | RS-71 | MMP9 | Predicted | 0.000 |
| RAB-36 | TRDMT1 | Predicted | 0.007 | RS-71 | MTAP | Predicted | 0.136 |
| RAB-36 | VEGFA | Predicted | 0.132 | RS-71 | NME1 | Predicted | 0.010 |
| RAB-37 | ABCB1 | Predicted | 0.006 | RS-71 | NME2 | Predicted | 0.001 |
| RAB-37 | ACSL1 | Predicted | 0.006 | RS-71 | PC | Predicted | 0.051 |
| RAB-37 | AKT2 | Predicted | 0.234 | RS-71 | PGK1 | Predicted | 0.002 |
| RAB-37 | ASS1 | Predicted | 0.006 | RS-71 | PTGS2 | Predicted | 0.725 |
| RAB-37 | AURKA | Predicted | 0.008 | RS-71 | PTK2 | Predicted | 0.002 |
| RAB-37 | CA1 | Predicted | 0.046 | RS-71 | REG1A | Predicted | 0.018 |
| RAB-37 | CA2 | Predicted | 0.046 | RS-71 | RHOA | Predicted | 0.001 |
| RAB-37 | CCNA2 | Predicted | 0.993 | RS-71 | SULT2A1 | Predicted | 0.047 |
| RAB-37 | CCT3 | Predicted | 0.024 | RS-71 | TGM2 | Predicted | 0.001 |

| RAB-37 | CES1 | Predicted | 0.051 | RS-71 | TOP2A | Predicted | 0.137 |
| --- | --- | --- | --- | --- | --- | --- | --- |
| RAB-37 | CTSB | Predicted | 0.163 | RS-72 | ACSL1 | Predicted | 0.018 |
| RAB-37 | ESR1 | Predicted | 0.322 | RS-72 | ALDOA | Predicted | 0.087 |
| RAB-37 | FGF1 | Predicted | 0.074 | RS-72 | CA1 | Predicted | 0.178 |
| RAB-37 | FGF2 | Predicted | 0.074 | RS-72 | CA2 | Predicted | 0.105 |
| RAB-37 | GABRE | Predicted | 0.022 | RS-72 | CCNA2 | Predicted | 0.352 |
| RAB-37 | GLUD1 | Predicted | 0.038 | RS-72 | CTSB | Predicted | 0.082 |
| RAB-37 | GNMT | Predicted | 0.000 | RS-72 | ESR1 | Predicted | 0.222 |
| RAB-37 | HSP90AA1 | Predicted | 0.008 | RS-72 | GABRE | Predicted | 0.011 |
| RAB-37 | HSP90B1 | Predicted | 0.008 | RS-72 | GNAI1 | Predicted | 0.000 |
| RAB-37 | HSPA1B | Predicted | 0.008 | RS-72 | GSTP1 | Predicted | 0.000 |
| RAB-37 | HSPA8 | Predicted | 0.008 | RS-72 | KRAS | Predicted | 0.005 |
| RAB-37 | KRAS | Predicted | 0.073 | RS-72 | LCN2 | Predicted | 0.083 |
| RAB-37 | LYZ | Predicted | 0.075 | RS-72 | MAPK3 | Predicted | 0.057 |
| RAB-37 | MAPK1 | Predicted | 0.067 | RS-72 | MMP12 | Predicted | 0.081 |
| RAB-37 | MAT1A | Predicted | 0.008 | RS-72 | MMP3 | Predicted | 0.037 |
| RAB-37 | MME | Predicted | 0.105 | RS-72 | MTAP | Predicted | 0.183 |
| RAB-37 | MMP12 | Predicted | 0.052 | RS-72 | NME1 | Predicted | 0.000 |
| RAB-37 | MMP14 | Predicted | 0.016 | RS-72 | NME2 | Predicted | 0.000 |
| RAB-37 | MMP2 | Predicted | 0.016 | RS-72 | NOS2A | Predicted | 0.177 |
| RAB-37 | MMP3 | Predicted | 0.045 | RS-72 | PTGS2 | Predicted | 0.453 |
| RAB-37 | MMP7 | Predicted | 0.016 | RS-72 | RHOA | Predicted | 0.000 |
| RAB-37 | MMP9 | Predicted | 0.016 | RS-72 | SULT2A1 | Predicted | 0.013 |
| RAB-37 | NME1 | Predicted | 0.024 | RS-72 | TGM2 | Predicted | 0.000 |
| RAB-37 | NOS2A | Predicted | 0.045 | RS-72 | TOP2A | Predicted | 0.034 |

| RAB-37 | PGK1 | Predicted | 0.008 | RS-74 | ACSL1 | Predicted | 0.031 |
| --- | --- | --- | --- | --- | --- | --- | --- |
| RAB-37 | PTGS2 | Predicted | 0.085 | RS-74 | AGXT | Predicted | 0.006 |
| RAB-37 | PTK2 | Predicted | 0.008 | RS-74 | ALDOA | Predicted | 0.120 |
| RAB-37 | RND3 | Predicted | 0.021 | RS-74 | AURKA | Predicted | 0.010 |
| RAB-37 | SRC | Predicted | 0.080 | RS-74 | CA1 | Predicted | 0.126 |
| RAB-37 | SULT2A1 | Predicted | 0.015 | RS-74 | CA2 | Predicted | 0.126 |
| RAB-37 | TOP2A | Predicted | 0.106 | RS-74 | CCNA2 | Predicted | 0.740 |
| RAB-37 | TRDMT1 | Predicted | 0.000 | RS-74 | CCT3 | Predicted | 0.002 |
| RAB-37 | VEGFA | Predicted | 0.036 | RS-74 | CES1 | Predicted | 0.035 |
| RAB-38 | ABCB1 | Predicted | 0.011 | RS-74 | CTSB | Predicted | 0.116 |
| RAB-38 | ACSL1 | Predicted | 0.011 | RS-74 | ESR1 | Predicted | 0.109 |
| RAB-38 | ADH1B | Predicted | 0.002 | RS-74 | GABRE | Predicted | 0.027 |
| RAB-38 | ADH1C | Predicted | 0.002 | RS-74 | GLUD1 | Predicted | 0.002 |
| RAB-38 | ADH4 | Predicted | 0.002 | RS-74 | GNAI1 | Predicted | 0.005 |
| RAB-38 | AKT2 | Predicted | 0.046 | RS-74 | GNMT | Predicted | 0.004 |
| RAB-38 | ALDH1A1 | Predicted | 0.002 | RS-74 | GSTP1 | Predicted | 0.000 |
| RAB-38 | ALDH1L1 | Predicted | 0.010 | RS-74 | HSP90AA1 | Predicted | 0.002 |
| RAB-38 | ALDH2 | Predicted | 0.002 | RS-74 | HSP90B1 | Predicted | 0.002 |
| RAB-38 | ASS1 | Predicted | 0.011 | RS-74 | HSPA1B | Predicted | 0.002 |
| RAB-38 | AURKA | Predicted | 0.319 | RS-74 | HSPA8 | Predicted | 0.002 |
| RAB-38 | CCNA2 | Predicted | 1.000 | RS-74 | KRAS | Predicted | 0.022 |
| RAB-38 | CCT3 | Predicted | 0.006 | RS-74 | MAPK3 | Predicted | 0.081 |
| RAB-38 | DDR1 | Predicted | 0.043 | RS-74 | MAT1A | Predicted | 0.002 |
| RAB-38 | EGFR | Predicted | 0.125 | RS-74 | MMP12 | Predicted | 0.004 |
| RAB-38 | ESR1 | Predicted | 0.221 | RS-74 | MMP14 | Predicted | 0.004 |

| RAB-38 | FTCD | Predicted | 0.010 | RS-74 | MMP2 | Predicted | 0.004 |
| --- | --- | --- | --- | --- | --- | --- | --- |
| RAB-38 | GAPDH | Predicted | 0.047 | RS-74 | MMP3 | Predicted | 0.014 |
| RAB-38 | GLUD1 | Predicted | 0.015 | RS-74 | MMP7 | Predicted | 0.004 |
| RAB-38 | GNMT | Predicted | 0.000 | RS-74 | MMP9 | Predicted | 0.004 |
| RAB-38 | GSTP1 | Predicted | 0.103 | RS-74 | MTAP | Predicted | 0.249 |
| RAB-38 | HSD17B10 | Predicted | 0.002 | RS-74 | NME1 | Predicted | 0.013 |
| RAB-38 | HSD17B4 | Predicted | 0.002 | RS-74 | NME2 | Predicted | 0.005 |
| RAB-38 | KRAS | Predicted | 0.047 | RS-74 | NOS2A | Predicted | 0.243 |
| RAB-38 | MAPK1 | Predicted | 0.009 | RS-74 | PGK1 | Predicted | 0.002 |
| RAB-38 | MME | Predicted | 0.301 | RS-74 | PTGS2 | Predicted | 0.623 |
| RAB-38 | MTHFD1 | Predicted | 0.010 | RS-74 | PTK2 | Predicted | 0.002 |
| RAB-38 | MTR | Predicted | 0.010 | RS-74 | RHOA | Predicted | 0.005 |
| RAB-38 | NME1 | Predicted | 0.123 | RS-74 | SRC | Predicted | 0.116 |
| RAB-38 | PDGFRA | Predicted | 0.043 | RS-74 | TGM2 | Predicted | 0.005 |
| RAB-38 | PTGS2 | Predicted | 0.052 | RS-74 | TRDMT1 | Predicted | 0.004 |
| RAB-38 | QDPR | Predicted | 0.002 | RS-75 | ABCB1 | Predicted | 0.006 |
| RAB-38 | RAF1 | Predicted | 0.041 | RS-75 | ACSL1 | Predicted | 0.036 |
| RAB-38 | RARA | Predicted | 0.054 | RS-75 | AR | Predicted | 0.056 |
| RAB-38 | RND3 | Predicted | 0.006 | RS-75 | ASS1 | Predicted | 0.006 |
| RAB-38 | SERPINE1 | Predicted | 0.018 | RS-75 | AURKA | Predicted | 0.002 |
| RAB-38 | SRC | Predicted | 0.043 | RS-75 | CA1 | Predicted | 0.035 |
| RAB-38 | TRDMT1 | Predicted | 0.000 | RS-75 | CA2 | Predicted | 0.035 |
| RAB-38 | TUBB | Predicted | 0.005 | RS-75 | CCNA2 | Predicted | 0.127 |
| RAB-38 | VEGFA | Predicted | 0.025 | RS-75 | CCT3 | Predicted | 0.015 |
| RAB-39 | ADH1B | Predicted | 0.043 | RS-75 | CES1 | Predicted | 0.037 |

| RAB-39 | ADH1C | Predicted | 0.025 | RS-75 | CTSB | Predicted | 0.130 |
| --- | --- | --- | --- | --- | --- | --- | --- |
| RAB-39 | ADH4 | Predicted | 0.025 | RS-75 | ESR1 | Predicted | 0.268 |
| RAB-39 | AKR1B10 | Predicted | 0.000 | RS-75 | GABRE | Predicted | 0.014 |
| RAB-39 | AKR1C2 | Predicted | 0.019 | RS-75 | GLUD1 | Predicted | 0.015 |
| RAB-39 | ALDH1A1 | Predicted | 0.025 | RS-75 | GNAI1 | Predicted | 0.000 |
| RAB-39 | ALDH1B1 | Predicted | 0.002 | RS-75 | GSTP1 | Predicted | 0.005 |
| RAB-39 | ALDH2 | Predicted | 0.025 | RS-75 | HSP90AA1 | Predicted | 0.002 |
| RAB-39 | ALDH3A2 | Predicted | 0.002 | RS-75 | HSP90B1 | Predicted | 0.002 |
| RAB-39 | ALDH4A1 | Predicted | 0.002 | RS-75 | HSPA1B | Predicted | 0.002 |
| RAB-39 | CCNA2 | Predicted | 0.488 | RS-75 | HSPA8 | Predicted | 0.002 |
| RAB-39 | DCXR | Predicted | 0.000 | RS-75 | KRAS | Predicted | 0.014 |
| RAB-39 | GAPDH | Predicted | 0.070 | RS-75 | LYZ | Predicted | 0.007 |
| RAB-39 | GLUD1 | Predicted | 0.025 | RS-75 | MAT1A | Predicted | 0.002 |
| RAB-39 | GSTP1 | Predicted | 0.486 | RS-75 | MME | Predicted | 0.081 |
| RAB-39 | HSD17B10 | Predicted | 0.025 | RS-75 | MMP12 | Predicted | 0.039 |
| RAB-39 | HSD17B4 | Predicted | 0.025 | RS-75 | MMP14 | Predicted | 0.010 |
| RAB-39 | MME | Predicted | 0.490 | RS-75 | MMP2 | Predicted | 0.010 |
| RAB-39 | MTHFD1 | Predicted | 0.019 | RS-75 | MMP3 | Predicted | 0.034 |
| RAB-39 | QDPR | Predicted | 0.025 | RS-75 | MMP7 | Predicted | 0.010 |
| RAB-39 | TOP2A | Predicted | 0.588 | RS-75 | MMP9 | Predicted | 0.010 |
| RAB-39 | VEGFA | Predicted | 0.057 | RS-75 | MTAP | Predicted | 0.126 |
| RAB-40 | ADH1B | Predicted | 0.017 | RS-75 | NME1 | Predicted | 0.024 |
| RAB-40 | ADH1C | Predicted | 0.009 | RS-75 | NME2 | Predicted | 0.000 |
| RAB-40 | ADH4 | Predicted | 0.009 | RS-75 | NOS2A | Predicted | 0.093 |
| RAB-40 | AKR1B10 | Predicted | 0.001 | RS-75 | PGK1 | Predicted | 0.002 |

| RAB-40 | AKR1C2 | Predicted | 0.009 | RS-75 | PTGS2 | Predicted | 0.074 |
| --- | --- | --- | --- | --- | --- | --- | --- |
| RAB-40 | ALDH1A1 | Predicted | 0.009 | RS-75 | PTK2 | Predicted | 0.002 |
| RAB-40 | ALDH1B1 | Predicted | 0.000 | RS-75 | RHOA | Predicted | 0.000 |
| RAB-40 | ALDH2 | Predicted | 0.009 | RS-75 | RND3 | Predicted | 0.001 |
| RAB-40 | ALDH3A2 | Predicted | 0.000 | RS-75 | SULT2A1 | Predicted | 0.013 |
| RAB-40 | ALDH4A1 | Predicted | 0.000 | RS-75 | TGM2 | Predicted | 0.000 |
| RAB-40 | DCXR | Predicted | 0.001 | RS-76 | ALDH2 | Predicted | 0.085 |
| RAB-40 | GAPDH | Predicted | 0.009 | RS-76 | ALDOA | Predicted | 0.087 |
| RAB-40 | GLUD1 | Predicted | 0.009 | RS-76 | AR | Predicted | 0.083 |
| RAB-40 | GSTP1 | Predicted | 0.494 | RS-76 | ARG1 | Predicted | 0.038 |
| RAB-40 | HSD17B10 | Predicted | 0.009 | RS-76 | B2M | Predicted | 0.018 |
| RAB-40 | HSD17B4 | Predicted | 0.009 | RS-76 | CA1 | Predicted | 0.219 |
| RAB-40 | MMP12 | Predicted | 0.118 | RS-76 | CA2 | Predicted | 0.130 |
| RAB-40 | MTHFD1 | Predicted | 0.009 | RS-76 | CCNA2 | Predicted | 0.170 |
| RAB-40 | QDPR | Predicted | 0.009 | RS-76 | ESR1 | Predicted | 0.658 |
| RAB-41 | ADH1B | Predicted | 0.013 | RS-76 | FGF1 | Predicted | 0.014 |
| RAB-41 | ADH1C | Predicted | 0.000 | RS-76 | FGF2 | Predicted | 0.014 |
| RAB-41 | ADH4 | Predicted | 0.000 | RS-76 | GABRE | Predicted | 0.007 |
| RAB-41 | AKR1B10 | Predicted | 0.000 | RS-76 | GSTP1 | Predicted | 0.000 |
| RAB-41 | AKR1C2 | Predicted | 0.013 | RS-76 | HGF | Predicted | 0.014 |
| RAB-41 | ALDH1A1 | Predicted | 0.000 | RS-76 | KRAS | Predicted | 0.007 |
| RAB-41 | ALDH1B1 | Predicted | 0.000 | RS-76 | LCN2 | Predicted | 0.086 |
| RAB-41 | ALDH2 | Predicted | 0.000 | RS-76 | MAPK1 | Predicted | 0.034 |
| RAB-41 | ALDH3A2 | Predicted | 0.000 | RS-76 | MTAP | Predicted | 0.171 |
| RAB-41 | ALDH4A1 | Predicted | 0.000 | RS-76 | NOS2A | Predicted | 0.244 |

| RAB-41 | CASP3 | Validated | 1.000 | RS-77 | ACSL1 | Predicted | 0.024 |
| --- | --- | --- | --- | --- | --- | --- | --- |
| RAB-41 | CAT | Validated | 1.000 | RS-77 | ALDH2 | Predicted | 0.085 |
| RAB-41 | CCNA2 | Predicted | 0.320 | RS-77 | ALDOA | Predicted | 0.085 |
| RAB-41 | CCT3 | Predicted | 0.017 | RS-77 | AR | Predicted | 0.085 |
| RAB-41 | DCXR | Predicted | 0.000 | RS-77 | B2M | Predicted | 0.018 |
| RAB-41 | ESR1 | Predicted | 0.321 | RS-77 | CA1 | Predicted | 0.177 |
| RAB-41 | GAPDH | Predicted | 0.046 | RS-77 | CA2 | Predicted | 0.177 |
| RAB-41 | GLUD1 | Predicted | 0.000 | RS-77 | CCNA2 | Predicted | 0.089 |
| RAB-41 | GSTP1 | Predicted | 1.000 | RS-77 | ESR1 | Predicted | 0.137 |
| RAB-41 | HAO1 | Predicted | 0.001 | RS-77 | GABRE | Predicted | 0.015 |
| RAB-41 | HAS2 | Validated | 1.000 | RS-77 | GSTP1 | Predicted | 0.000 |
| RAB-41 | HSD17B10 | Predicted | 0.000 | RS-77 | KRAS | Predicted | 0.007 |
| RAB-41 | HSD17B4 | Predicted | 0.000 | RS-77 | LCN2 | Predicted | 0.086 |
| RAB-41 | KRAS | Predicted | 0.128 | RS-77 | MAPK1 | Predicted | 0.038 |
| RAB-41 | MMP12 | Predicted | 0.103 | RS-77 | MTAP | Predicted | 0.179 |
| RAB-41 | MTHFD1 | Predicted | 0.013 | RS-77 | NOS2A | Predicted | 0.113 |
| RAB-41 | NOS2A | Validated | 1.000 | RS-77 | PARP1 | Predicted | 0.086 |
| RAB-41 | QDPR | Predicted | 0.000 | RS-77 | PC | Predicted | 0.007 |
| RAB-41 | SGK1 | Predicted | 0.001 | RS-77 | PTGS2 | Predicted | 0.980 |
| RAB-41 | SOD1 | Validated | 1.000 | RS-77 | SRC | Predicted | 0.023 |
| RAB-41 | VEGFA | Predicted | 0.030 | RS-78 | ALDH2 | Predicted | 0.131 |
| RAB-42 | ABCB1 | Predicted | 0.012 | RS-78 | APC | validated | 1.000 |
| RAB-42 | ACSL1 | Predicted | 0.012 | RS-78 | AR | validated | 1.000 |
| RAB-42 | ADH1B | Predicted | 0.017 | RS-78 | BAD | validated | 1.000 |
| RAB-42 | ADH1C | Predicted | 0.019 | RS-78 | CA1 | Predicted | 0.125 |

| RAB-42 | ADH4 | Predicted | 0.010 | RS-78 | CA2 | Predicted | 0.058 |
| --- | --- | --- | --- | --- | --- | --- | --- |
| RAB-42 | AKR1B10 | Predicted | 0.000 | RS-78 | CASP3 | validated | 1.000 |
| RAB-42 | AKR1C2 | Predicted | 0.008 | RS-78 | CCNA2 | validated | 1.000 |
| RAB-42 | AKT2 | Predicted | 0.053 | RS-78 | CDK4 | validated | 1.000 |
| RAB-42 | ALDH1A1 | Predicted | 0.010 | RS-78 | CDKN1A | validated | 1.000 |
| RAB-42 | ALDH1B1 | Predicted | 0.001 | RS-78 | CDKN2A | validated | 1.000 |
| RAB-42 | ALDH1L1 | Predicted | 0.000 | RS-78 | ESR1 | validated | 1.000 |
| RAB-42 | ALDH2 | Predicted | 0.010 | RS-78 | FOS | validated | 1.000 |
| RAB-42 | ALDH3A2 | Predicted | 0.001 | RS-78 | ICAM1 | validated | 1.000 |
| RAB-42 | ALDH4A1 | Predicted | 0.001 | RS-78 | IL2 | validated | 1.000 |
| RAB-42 | ASS1 | Predicted | 0.012 | RS-78 | KRAS | Predicted | 0.007 |
| RAB-42 | CCNA2 | Predicted | 0.142 | RS-78 | MCL1 | validated | 1.000 |
| RAB-42 | CCT3 | Predicted | 0.009 | RS-78 | MMP9 | validated | 1.000 |
| RAB-42 | CES1 | Predicted | 0.003 | RS-78 | MTAP | Predicted | 0.264 |
| RAB-42 | DCXR | Predicted | 0.000 | RS-78 | NFKBIA | validated | 1.000 |
| RAB-42 | DDR1 | Predicted | 0.014 | RS-78 | NOS2A | validated | 1.000 |
| RAB-42 | EGFR | Predicted | 0.143 | RS-78 | PLAU | validated | 1.000 |
| RAB-42 | ESR1 | Predicted | 0.284 | RS-78 | PTGS2 | validated | 1.000 |
| RAB-42 | FTCD | Predicted | 0.000 | RS-78 | RB1 | validated | 1.000 |
| RAB-42 | GAPDH | Predicted | 0.024 | RS-78 | SERPINE1 | validated | 1.000 |
| RAB-42 | GLUD1 | Predicted | 0.021 | RS-78 | SRC | Predicted | 0.124 |
| RAB-42 | GSTP1 | Predicted | 0.091 | RS-78 | TOP2A | Predicted | 0.079 |
| RAB-42 | HSD17B10 | Predicted | 0.010 | RS-78 | TP53 | validated | 1.000 |
| RAB-42 | HSD17B4 | Predicted | 0.010 | RS-78 | VEGFA | validated | 1.000 |
| RAB-42 | KRAS | Predicted | 0.023 | RS-79 | ACSL1 | Predicted | 0.026 |

| RAB-42 | MAPK1 | Predicted | 0.012 | RS-79 | ALDH2 | Predicted | 0.098 |
| --- | --- | --- | --- | --- | --- | --- | --- |
| RAB-42 | MME | Predicted | 0.323 | RS-79 | ALDOA | Predicted | 0.096 |
| RAB-42 | MMP12 | Predicted | 0.030 | RS-79 | CA1 | Predicted | 0.095 |
| RAB-42 | MTHFD1 | Predicted | 0.015 | RS-79 | CA2 | Predicted | 0.046 |
| RAB-42 | MTR | Predicted | 0.000 | RS-79 | CCNA2 | Predicted | 0.389 |
| RAB-42 | NME1 | Predicted | 0.135 | RS-79 | ESR1 | Predicted | 0.532 |
| RAB-42 | PDGFRA | Predicted | 0.014 | RS-79 | FABP5 | Validated | 1.000 |
| RAB-42 | QDPR | Predicted | 0.010 | RS-79 | FOS | Validated | 1.000 |
| RAB-42 | RND3 | Predicted | 0.004 | RS-79 | GABRE | Predicted | 0.008 |
| RAB-42 | SERPINE1 | Predicted | 0.009 | RS-79 | GSTP1 | Predicted | 0.000 |
| RAB-42 | TUBB | Predicted | 0.020 | RS-79 | IGF2 | Validated | 1.000 |
| RAB-42 | VEGFA | Predicted | 0.021 | RS-79 | KRAS | Predicted | 0.010 |
| RAB-43 | ABCB1 | Predicted | 0.005 | RS-79 | LCN2 | Predicted | 0.094 |
| RAB-43 | ACSL1 | Predicted | 0.005 | RS-79 | MMP9 | Validated | 1.000 |
| RAB-43 | ADH1C | Predicted | 0.005 | RS-79 | MTAP | Predicted | 0.198 |
| RAB-43 | AGXT | Predicted | 0.006 | RS-79 | NOS2A | Predicted | 0.098 |
| RAB-43 | AKR1B10 | Predicted | 0.089 | RS-79 | PTGS2 | Predicted | 0.887 |
| RAB-43 | AKT2 | Predicted | 0.045 | RS-79 | SRC | Validated | 1.000 |
| RAB-43 | ALDH1A1 | Predicted | 0.017 | RS-79 | TOP2A | Predicted | 0.064 |
| RAB-43 | ASS1 | Predicted | 0.005 | RS-79 | VEGFA | Validated | 1.000 |
| RAB-43 | AURKA | Predicted | 0.004 | RS-80 | ACSL1 | Predicted | 0.018 |
| RAB-43 | CA1 | Predicted | 0.043 | RS-80 | ALDH2 | Predicted | 0.090 |
| RAB-43 | CA2 | Predicted | 0.043 | RS-80 | AURKA | Predicted | 0.087 |
| RAB-43 | CCNA2 | Predicted | 1.000 | RS-80 | CA1 | Predicted | 0.133 |
| RAB-43 | CCT3 | Predicted | 0.012 | RS-80 | CA2 | Predicted | 0.097 |

| RAB-43 | CDK4 | Predicted | 0.043 | RS-80 | CCNA2 | Predicted | 0.380 |
| --- | --- | --- | --- | --- | --- | --- | --- |
| RAB-43 | CES1 | Predicted | 0.005 | RS-80 | ESR1 | Predicted | 0.383 |
| RAB-43 | CTSB | Predicted | 0.088 | RS-80 | GABRE | Predicted | 0.005 |
| RAB-43 | ESR1 | Predicted | 0.178 | RS-80 | KRAS | Predicted | 0.002 |
| RAB-43 | GLUD1 | Predicted | 0.014 | RS-80 | LCN2 | Predicted | 0.087 |
| RAB-43 | GNAI1 | Predicted | 0.005 | RS-80 | MTAP | Predicted | 0.190 |
| RAB-43 | GNMT | Predicted | 0.004 | RS-80 | NOS2A | Predicted | 0.188 |
| RAB-43 | GSTP1 | Predicted | 0.027 | RS-80 | PTGS2 | Predicted | 0.690 |
| RAB-43 | HAO1 | Predicted | 0.000 | RS-80 | TOP2A | Predicted | 0.054 |
| RAB-43 | HSP90AA1 | Predicted | 0.004 | RS-81 | ACSL1 | Predicted | 0.024 |
| RAB-43 | HSP90B1 | Predicted | 0.004 | RS-81 | ALDH2 | Predicted | 0.083 |
| RAB-43 | HSPA1B | Predicted | 0.004 | RS-81 | ALDOA | Predicted | 0.087 |
| RAB-43 | HSPA8 | Predicted | 0.004 | RS-81 | AR | Predicted | 0.039 |
| RAB-43 | KRAS | Predicted | 0.052 | RS-81 | CA1 | Predicted | 0.173 |
| RAB-43 | LYZ | Predicted | 0.087 | RS-81 | CA2 | Predicted | 0.084 |
| RAB-43 | MAPK1 | Predicted | 0.089 | RS-81 | CCNA2 | Predicted | 0.262 |
| RAB-43 | MAPK3 | Predicted | 0.043 | RS-81 | ESR1 | Predicted | 0.228 |
| RAB-43 | MAT1A | Predicted | 0.004 | RS-81 | GABRE | Predicted | 0.010 |
| RAB-43 | MME | Predicted | 0.135 | RS-81 | GSTP1 | Predicted | 0.000 |
| RAB-43 | NME1 | Predicted | 0.020 | RS-81 | KRAS | Predicted | 0.007 |
| RAB-43 | NME2 | Predicted | 0.005 | RS-81 | LCN2 | Predicted | 0.084 |
| RAB-43 | PGK1 | Predicted | 0.004 | RS-81 | MTAP | Predicted | 0.179 |
| RAB-43 | PTGS2 | Predicted | 0.022 | RS-81 | NOS2A | Predicted | 0.265 |
| RAB-43 | PTK2 | Predicted | 0.004 | RS-81 | PC | Predicted | 0.031 |
| RAB-43 | RHOA | Predicted | 0.005 | RS-81 | PTGS2 | Predicted | 0.702 |

| RAB-43 | RND3 | Predicted | 0.007 | RS-81 | TOP2A | Predicted | 0.050 |
| --- | --- | --- | --- | --- | --- | --- | --- |
| RAB-43 | SERPINE1 | Predicted | 0.016 | RS-82 | ACSL1 | Predicted | 0.026 |
| RAB-43 | SGK1 | Predicted | 0.000 | RS-82 | ALDOA | Predicted | 0.096 |
| RAB-43 | SRC | Predicted | 0.119 | RS-82 | AURKA | Predicted | 0.005 |
| RAB-43 | TGM2 | Predicted | 0.005 | RS-82 | CA1 | Predicted | 0.102 |
| RAB-43 | TOP2A | Predicted | 0.023 | RS-82 | CA2 | Predicted | 0.102 |
| RAB-43 | TRDMT1 | Predicted | 0.004 | RS-82 | CCT3 | Predicted | 0.000 |
| RAB-43 | VEGFA | Predicted | 0.026 | RS-82 | CTSB | Predicted | 0.095 |
| RAB-44 | AURKA | Predicted | 0.486 | RS-82 | ESR1 | Predicted | 0.089 |
| RAB-44 | DDR1 | Predicted | 0.047 | RS-82 | GABRE | Predicted | 0.014 |
| RAB-44 | PDGFRA | Predicted | 0.047 | RS-82 | GLUD1 | Predicted | 0.000 |
| RAB-44 | PTGS2 | Predicted | 0.061 | RS-82 | GNAI1 | Predicted | 0.003 |
| RAB-44 | RAF1 | Predicted | 0.044 | RS-82 | GSTP1 | Predicted | 0.002 |
| RAB-44 | SRC | Predicted | 0.046 | RS-82 | HSP90AA1 | Predicted | 0.000 |
| RAB-45 | ADH1B | Predicted | 0.024 | RS-82 | HSP90B1 | Predicted | 0.000 |
| RAB-45 | ADH1C | Predicted | 0.014 | RS-82 | HSPA1B | Predicted | 0.000 |
| RAB-45 | ADH4 | Predicted | 0.014 | RS-82 | HSPA8 | Predicted | 0.000 |
| RAB-45 | AKR1B10 | Predicted | 0.000 | RS-82 | KRAS | Predicted | 0.017 |
| RAB-45 | AKR1C2 | Predicted | 0.011 | RS-82 | LYZ | Predicted | 0.094 |
| RAB-45 | AKT2 | Predicted | 0.075 | RS-82 | MAPK3 | Predicted | 0.065 |
| RAB-45 | ALDH1A1 | Predicted | 0.014 | RS-82 | MAT1A | Predicted | 0.000 |
| RAB-45 | ALDH1B1 | Predicted | 0.001 | RS-82 | MMP12 | Predicted | 0.003 |
| RAB-45 | ALDH2 | Predicted | 0.014 | RS-82 | MMP14 | Predicted | 0.002 |
| RAB-45 | ALDH3A2 | Predicted | 0.001 | RS-82 | MMP2 | Predicted | 0.002 |
| RAB-45 | ALDH4A1 | Predicted | 0.001 | RS-82 | MMP3 | Predicted | 0.011 |

| RAB-45 | CCNA2 | Predicted | 0.250 | RS-82 | MMP7 | Predicted | 0.002 |
| --- | --- | --- | --- | --- | --- | --- | --- |
| RAB-45 | DCXR | Predicted | 0.000 | RS-82 | MMP9 | Validated | 1.000 |
| RAB-45 | GAPDH | Predicted | 0.038 | RS-82 | MTAP | Predicted | 0.196 |
| RAB-45 | GLUD1 | Predicted | 0.027 | RS-82 | NME1 | Predicted | 0.009 |
| RAB-45 | GSTP1 | Predicted | 0.249 | RS-82 | NME2 | Predicted | 0.003 |
| RAB-45 | HSD17B10 | Predicted | 0.014 | RS-82 | NOS2A | Predicted | 0.201 |
| RAB-45 | HSD17B4 | Predicted | 0.014 | RS-82 | PGK1 | Predicted | 0.000 |
| RAB-45 | MMP12 | Predicted | 0.054 | RS-82 | PTGS2 | Predicted | 0.509 |
| RAB-45 | MTHFD1 | Predicted | 0.011 | RS-82 | PTK2 | Predicted | 0.000 |
| RAB-45 | QDPR | Predicted | 0.014 | RS-82 | RHOA | Predicted | 0.003 |
| RAB-45 | RND3 | Predicted | 0.003 | RS-82 | SRC | Predicted | 0.096 |
| RAB-45 | VEGFA | Predicted | 0.034 | RS-82 | TGM2 | Predicted | 0.003 |
| RAB-46 | ALB | Predicted | 0.995 | RS-83 | EGFR | Predicted | 0.925 |
| RAB-46 | GPC3 | Predicted | 1.000 | RS-84 | AGXT | Predicted | 0.004 |
| RAB-47 | AGXT | Predicted | 0.301 | RS-84 | ALAS1 | Predicted | 0.004 |
| RAB-47 | AKR1B10 | Predicted | 0.299 | RS-84 | ALDOA | Predicted | 0.102 |
| RAB-47 | ALB | Predicted | 0.504 | RS-84 | ARG1 | Predicted | 0.046 |
| RAB-47 | ALDOB | Predicted | 0.392 | RS-84 | B2M | Predicted | 0.033 |
| RAB-47 | APOA1 | Predicted | 0.299 | RS-84 | CA1 | Predicted | 0.319 |
| RAB-47 | ARG1 | Predicted | 0.299 | RS-84 | CA2 | Predicted | 0.289 |
| RAB-47 | CCT3 | Predicted | 0.269 | RS-84 | CA3 | Predicted | 0.025 |
| RAB-47 | CLIC1 | Predicted | 0.255 | RS-84 | CBS | Predicted | 0.004 |
| RAB-47 | CPS1 | Predicted | 0.327 | RS-84 | CCNA2 | Predicted | 0.104 |
| RAB-47 | CYP2E1 | Predicted | 0.494 | RS-84 | CTH | Predicted | 0.004 |
| RAB-47 | GLUL | Predicted | 0.255 | RS-84 | DNMT1 | Predicted | 0.050 |

| RAB-47 | GPC3 | Predicted | 0.510 | RS-84 | ESR1 | Predicted | 0.020 |
| --- | --- | --- | --- | --- | --- | --- | --- |
| RAB-47 | HMMR | Predicted | 0.255 | RS-84 | FTCD | Predicted | 0.004 |
| RAB-47 | HSP90B1 | Predicted | 0.252 | RS-84 | GABRE | Predicted | 0.051 |
| RAB-47 | HSPA5 | Predicted | 0.321 | RS-84 | GSTP1 | Predicted | 0.000 |
| RAB-47 | HSPB1 | Predicted | 0.260 | RS-84 | HAO1 | Predicted | 0.106 |
| RAB-47 | IGF2 | Predicted | 0.260 | RS-84 | KRAS | Predicted | 0.010 |
| RAB-47 | IGFBP3 | Predicted | 0.302 | RS-84 | LCN2 | Predicted | 0.103 |
| RAB-47 | PLG | Predicted | 0.500 | RS-84 | MAPK1 | Predicted | 0.029 |
| RAB-47 | SPARC | Predicted | 0.481 | RS-84 | MTAP | Predicted | 0.101 |
| RAB-47 | STMN1 | Predicted | 0.252 | RS-84 | NOS2A | Predicted | 0.241 |
| RAB-47 | SULT2A1 | Predicted | 0.258 | RS-84 | OAT | Predicted | 0.004 |
| RAB-47 | UBD | Predicted | 0.256 | RS-84 | PARP1 | Predicted | 0.109 |
| RAB-48 | AGXT | Predicted | 0.479 | RS-84 | PC | Predicted | 0.040 |
| RAB-48 | AKR1B10 | Predicted | 0.478 | RS-84 | PIN1 | Predicted | 0.054 |
| RAB-48 | ALB | Predicted | 0.641 | RS-84 | PTGS2 | Predicted | 0.733 |
| RAB-48 | ALDOB | Predicted | 0.490 | RS-84 | PYGB | Predicted | 0.004 |
| RAB-48 | APOA1 | Predicted | 0.477 | RS-85 | ALDH2 | Predicted | 0.083 |
| RAB-48 | ARG1 | Predicted | 0.476 | RS-85 | AR | Predicted | 0.082 |
| RAB-48 | CCT3 | Predicted | 0.473 | RS-85 | CA1 | Predicted | 0.098 |
| RAB-48 | CPS1 | Predicted | 0.489 | RS-85 | CA2 | Predicted | 0.045 |
| RAB-48 | CYP2E1 | Predicted | 0.492 | RS-85 | CCNA2 | Predicted | 0.086 |
| RAB-48 | GPC3 | Predicted | 0.642 | RS-85 | CDKN1A | Validated | 1.000 |
| RAB-48 | HSPA5 | Predicted | 0.486 | RS-85 | ESR1 | Predicted | 0.134 |
| RAB-48 | HSPB1 | Predicted | 0.473 | RS-85 | KRAS | Predicted | 0.004 |
| RAB-48 | IGF2 | Predicted | 0.472 | RS-85 | MAPK1 | Predicted | 0.032 |

| RAB-48 | IGFBP3 | Predicted | 0.480 | RS-85 | MTAP | Predicted | 0.177 |
| --- | --- | --- | --- | --- | --- | --- | --- |
| RAB-48 | PLG | Predicted | 0.500 | RS-85 | NOS2A | Predicted | 0.054 |
| RAB-48 | SPARC | Predicted | 0.491 | RS-85 | PARP1 | Predicted | 0.084 |
| RAB-48 | SULT2A1 | Predicted | 0.472 | RS-85 | PTGS2 | Predicted | 1.000 |
| RAB-49 | A2M | Predicted | 0.186 | RS-85 | SRC | Predicted | 0.020 |
| RAB-49 | AGXT | Predicted | 0.289 | RS-85 | TNFSF11 | Predicted | 0.022 |
| RAB-49 | AKR1B10 | Predicted | 0.285 | RB-01 | AURKA | Predicted | 0.601 |
| RAB-49 | ALB | Predicted | 0.441 | RB-01 | DDR1 | Predicted | 0.079 |
| RAB-49 | ALDOB | Predicted | 0.348 | RB-01 | PDGFRA | Predicted | 0.079 |
| RAB-49 | APOA1 | Predicted | 0.283 | RB-01 | PTGS2 | Predicted | 0.113 |
| RAB-49 | ARG1 | Predicted | 0.251 | RB-01 | SRC | Predicted | 0.078 |
| RAB-49 | ASS1 | Predicted | 0.186 | RB-03 | ABCB1 | Validated | 1.000 |
| RAB-49 | CA2 | Predicted | 0.170 | RB-03 | AR | Validated | 1.000 |
| RAB-49 | CAP2 | Predicted | 0.158 | RB-03 | CASP3 | Validated | 1.000 |
| RAB-49 | CCT3 | Predicted | 0.251 | RB-03 | FOS | Validated | 1.000 |
| RAB-49 | CDKN1A | Predicted | 0.157 | RB-03 | GLUL | Validated | 1.000 |
| RAB-49 | CLIC1 | Predicted | 0.219 | RB-03 | JUNB | Validated | 1.000 |
| RAB-49 | CPS1 | Predicted | 0.323 | RB-03 | NFKBIA | Validated | 1.000 |
| RAB-49 | CTGF | Predicted | 0.155 | RB-03 | PTGS2 | Validated | 1.000 |
| RAB-49 | CYP2E1 | Predicted | 0.407 | RB-03 | TGFA | Validated | 1.000 |
| RAB-49 | ETS2 | Predicted | 0.154 | RB-03 | VEGFA | Validated | 1.000 |
| RAB-49 | FABP5 | Predicted | 0.153 | RB-03 | ACSL1 | Predicted | 0.012 |
| RAB-49 | GLUL | Predicted | 0.218 | RB-03 | AKT2 | Predicted | 0.056 |
| RAB-49 | GNMT | Predicted | 0.148 | RB-03 | ALDH1L1 | Predicted | 0.013 |
| RAB-49 | GPC3 | Predicted | 0.453 | RB-03 | ASS1 | Predicted | 0.012 |

| RAB-49 | HDGF | Predicted | 0.147 | RB-03 | AURKA | Predicted | 0.140 |
| --- | --- | --- | --- | --- | --- | --- | --- |
| RAB-49 | HMMR | Predicted | 0.209 | RB-03 | CA1 | Predicted | 0.022 |
| RAB-49 | HSP90B1 | Predicted | 0.209 | RB-03 | CA2 | Predicted | 0.022 |
| RAB-49 | HSPA5 | Predicted | 0.295 | RB-03 | CCNA2 | Predicted | 0.863 |
| RAB-49 | HSPA8 | Predicted | 0.147 | RB-03 | CCT3 | Predicted | 0.005 |
| RAB-49 | HSPB1 | Predicted | 0.249 | RB-03 | CYP2C8 | Predicted | 0.011 |
| RAB-49 | IGF2 | Predicted | 0.249 | RB-03 | DDR1 | Predicted | 0.015 |
| RAB-49 | IGFBP3 | Predicted | 0.292 | RB-03 | ESR1 | Predicted | 0.041 |
| RAB-49 | MAT1A | Predicted | 0.147 | RB-03 | FTCD | Predicted | 0.013 |
| RAB-49 | MMP9 | Predicted | 0.147 | RB-03 | GLUD1 | Predicted | 0.014 |
| RAB-49 | PCNA | Predicted | 0.146 | RB-03 | GNMT | Predicted | 0.003 |
| RAB-49 | PLG | Predicted | 0.407 | RB-03 | GSTP1 | Predicted | 0.023 |
| RAB-49 | SPARC | Predicted | 0.373 | RB-03 | KRAS | Predicted | 0.067 |
| RAB-49 | STMN1 | Predicted | 0.209 | RB-03 | LYZ | Predicted | 0.142 |
| RAB-49 | SULT2A1 | Predicted | 0.248 | RB-03 | MTHFD1 | Predicted | 0.013 |
| RAB-49 | TOP2A | Predicted | 0.192 | RB-03 | MTR | Predicted | 0.013 |
| RAB-49 | UBD | Predicted | 0.230 | RB-03 | NME1 | Predicted | 0.143 |
| RAB-49 | VTN | Predicted | 0.146 | RB-03 | NOS2A | Predicted | 0.142 |
| RAB-50 | AGXT | Predicted | 0.332 | RB-03 | PDGFRA | Predicted | 0.015 |
| RAB-50 | AKR1B10 | Predicted | 0.330 | RB-03 | RAF1 | Predicted | 0.015 |
| RAB-50 | ALB | Predicted | 0.572 | RB-03 | RARA | Predicted | 0.061 |
| RAB-50 | ALDOB | Predicted | 0.382 | RB-03 | RND3 | Predicted | 0.014 |
| RAB-50 | APOA1 | Predicted | 0.330 | RB-03 | SERPINE1 | Predicted | 0.028 |
| RAB-50 | ARG1 | Predicted | 0.329 | RB-03 | TRDMT1 | Predicted | 0.003 |
| RAB-50 | CCT3 | Predicted | 0.328 | RB-05 | ABCB1 | Predicted | 0.004 |

| RAB-50 | CLIC1 | Predicted | 0.326 | RB-05 | ACSL1 | Predicted | 0.004 |
| --- | --- | --- | --- | --- | --- | --- | --- |
| RAB-50 | CPS1 | Predicted | 0.377 | RB-05 | AKT2 | Predicted | 0.156 |
| RAB-50 | CYP2E1 | Predicted | 0.566 | RB-05 | ASS1 | Predicted | 0.004 |
| RAB-50 | GLUL | Predicted | 0.324 | RB-05 | CCNA2 | Predicted | 1.000 |
| RAB-50 | GPC3 | Predicted | 0.644 | RB-05 | CCT3 | Predicted | 0.007 |
| RAB-50 | HMMR | Predicted | 0.323 | RB-05 | CDK4 | Predicted | 0.036 |
| RAB-50 | HSP90B1 | Predicted | 0.322 | RB-05 | CTSB | Predicted | 0.095 |
| RAB-50 | HSPA5 | Predicted | 0.342 | RB-05 | EGFR | Predicted | 0.093 |
| RAB-50 | HSPB1 | Predicted | 0.328 | RB-05 | ESR1 | Predicted | 0.093 |
| RAB-50 | IGF2 | Predicted | 0.327 | RB-05 | GABRE | Predicted | 0.000 |
| RAB-50 | IGFBP3 | Predicted | 0.339 | RB-05 | GAPDH | Predicted | 0.005 |
| RAB-50 | PLG | Predicted | 0.566 | RB-05 | GLUD1 | Predicted | 0.010 |
| RAB-50 | SPARC | Predicted | 0.418 | RB-05 | GNMT | Predicted | 0.004 |
| RAB-50 | STMN1 | Predicted | 0.322 | RB-05 | GSTP1 | Predicted | 0.028 |
| RAB-50 | SULT2A1 | Predicted | 0.327 | RB-05 | HAO1 | Predicted | 0.000 |
| RAB-50 | TOP2A | Predicted | 0.322 | RB-05 | KRAS | Predicted | 0.057 |
| RAB-50 | UBD | Predicted | 0.327 | RB-05 | MAPK1 | Predicted | 0.101 |
| RAB-51 | AGXT | Predicted | 0.501 | RB-05 | MAPK3 | Predicted | 0.036 |
| RAB-51 | AKR1B10 | Predicted | 0.499 | RB-05 | MME | Predicted | 0.147 |
| RAB-51 | ALB | Predicted | 0.985 | RB-05 | MMP12 | Predicted | 0.036 |
| RAB-51 | ALDOB | Predicted | 0.508 | RB-05 | NOS2A | Predicted | 0.096 |
| RAB-51 | APOA1 | Predicted | 0.497 | RB-05 | PTGS2 | Predicted | 0.026 |
| RAB-51 | ARG1 | Predicted | 0.495 | RB-05 | RND3 | Predicted | 0.010 |
| RAB-51 | CCT3 | Predicted | 0.495 | RB-05 | SERPINE1 | Predicted | 0.024 |
| RAB-51 | CLIC1 | Predicted | 0.494 | RB-05 | SGK1 | Predicted | 0.000 |

| RAB-51 | CPS1 | Predicted | 0.507 | RB-05 | SRC | Predicted | 0.128 |
| --- | --- | --- | --- | --- | --- | --- | --- |
| RAB-51 | CYP2E1 | Predicted | 0.513 | RB-05 | TOP2A | Predicted | 0.061 |
| RAB-51 | GLUL | Predicted | 0.492 | RB-05 | TRDMT1 | Predicted | 0.004 |
| RAB-51 | GPC3 | Predicted | 0.993 | RB-05 | VEGFA | Predicted | 0.016 |
| RAB-51 | HMMR | Predicted | 0.491 | RB-06 | ACSL1 | Predicted | 0.017 |
| RAB-51 | HSPA5 | Predicted | 0.502 | RB-06 | AURKA | Predicted | 0.000 |
| RAB-51 | HSPB1 | Predicted | 0.495 | RB-06 | CA1 | Predicted | 0.138 |
| RAB-51 | IGF2 | Predicted | 0.495 | RB-06 | CA2 | Predicted | 0.110 |
| RAB-51 | IGFBP3 | Predicted | 0.501 | RB-06 | CCNA2 | Predicted | 1.000 |
| RAB-51 | PLG | Predicted | 0.515 | RB-06 | CCT3 | Predicted | 0.000 |
| RAB-51 | SPARC | Predicted | 0.509 | RB-06 | CES1 | Predicted | 0.025 |
| RAB-51 | SULT2A1 | Predicted | 0.495 | RB-06 | CTSB | Predicted | 0.086 |
| RAB-51 | UBD | Predicted | 0.494 | RB-06 | ESR1 | Predicted | 0.080 |
| RAB-52 | ALB | Predicted | 0.319 | RB-06 | GLUD1 | Predicted | 0.000 |
| RAB-52 | CYP2E1 | Predicted | 0.317 | RB-06 | GNAI1 | Predicted | 0.002 |
| RAB-52 | GPC3 | Predicted | 1.000 | RB-06 | GSTP1 | Predicted | 0.002 |
| RAB-52 | PLG | Predicted | 0.318 | RB-06 | HSP90AA1 | Predicted | 0.000 |
| RAB-52 | SPARC | Predicted | 0.314 | RB-06 | HSP90B1 | Predicted | 0.000 |
| RAB-53 | A2M | Predicted | 0.193 | RB-06 | HSPA1B | Predicted | 0.000 |
| RAB-53 | AGXT | Predicted | 0.272 | RB-06 | HSPA8 | Predicted | 0.000 |
| RAB-53 | AKR1B10 | Predicted | 0.268 | RB-06 | KRAS | Predicted | 0.015 |
| RAB-53 | ALB | Predicted | 0.420 | RB-06 | LYZ | Predicted | 0.086 |
| RAB-53 | ALDOB | Predicted | 0.378 | RB-06 | MAPK3 | Predicted | 0.054 |
| RAB-53 | APOA1 | Predicted | 0.253 | RB-06 | MAT1A | Predicted | 0.000 |
| RAB-53 | ARG1 | Predicted | 0.247 | RB-06 | MTAP | Predicted | 0.179 |

| RAB-53 | ASS1 | Predicted | 0.191 | RB-06 | NME1 | Predicted | 0.007 |
| --- | --- | --- | --- | --- | --- | --- | --- |
| RAB-53 | CA2 | Predicted | 0.191 | RB-06 | NME2 | Predicted | 0.002 |
| RAB-53 | CAP2 | Predicted | 0.191 | RB-06 | NOS2A | Predicted | 0.180 |
| RAB-53 | CCT3 | Predicted | 0.247 | RB-06 | PGK1 | Predicted | 0.000 |
| RAB-53 | CLIC1 | Predicted | 0.203 | RB-06 | PTGS2 | Predicted | 0.293 |
| RAB-53 | CPS1 | Predicted | 0.375 | RB-06 | PTK2 | Predicted | 0.000 |
| RAB-53 | CYP2E1 | Predicted | 0.382 | RB-06 | RHOA | Predicted | 0.002 |
| RAB-53 | GLUL | Predicted | 0.201 | RB-06 | SRC | Predicted | 0.086 |
| RAB-53 | GPC3 | Predicted | 0.558 | RB-06 | SULT2A1 | Predicted | 0.002 |
| RAB-53 | HMMR | Predicted | 0.195 | RB-06 | TGM2 | Predicted | 0.002 |
| RAB-53 | HSP90B1 | Predicted | 0.193 | RB-06 | TOP2A | Predicted | 0.020 |
| RAB-53 | HSPA5 | Predicted | 0.375 | RB-07 | ABCB1 | Predicted | 0.018 |
| RAB-53 | HSPB1 | Predicted | 0.237 | RB-07 | ACSL1 | Predicted | 0.018 |
| RAB-53 | IGF2 | Predicted | 0.237 | RB-07 | ADH1C | Predicted | 0.012 |
| RAB-53 | IGFBP3 | Predicted | 0.308 | RB-07 | AGXT | Predicted | 0.013 |
| RAB-53 | PLG | Predicted | 0.392 | RB-07 | AKT2 | Predicted | 0.387 |
| RAB-53 | SPARC | Predicted | 0.380 | RB-07 | ASS1 | Predicted | 0.018 |
| RAB-53 | STMN1 | Predicted | 0.193 | RB-07 | CA1 | Predicted | 0.058 |
| RAB-53 | SULT2A1 | Predicted | 0.211 | RB-07 | CA2 | Predicted | 0.058 |
| RAB-53 | TOP2A | Predicted | 0.193 | RB-07 | CASP1 | Predicted | 0.037 |
| RAB-53 | UBD | Predicted | 0.211 | RB-07 | CCNA2 | Predicted | 0.777 |
| RAB-54 | ALDH2 | Predicted | 0.118 | RB-07 | CDK4 | Predicted | 0.047 |
| RAB-54 | ALDOA | Predicted | 0.114 | RB-07 | CES1 | Predicted | 0.036 |
| RAB-54 | AR | Predicted | 0.124 | RB-07 | CTSB | Predicted | 0.194 |
| RAB-54 | AURKA | Predicted | 0.002 | RB-07 | ESR1 | Predicted | 0.395 |

| RAB-54 | CA1 | Predicted | 0.108 | RB-07 | FGF1 | Predicted | 0.095 |
| --- | --- | --- | --- | --- | --- | --- | --- |
| RAB-54 | CA2 | Predicted | 0.093 | RB-07 | FGF2 | Predicted | 0.095 |
| RAB-54 | CCT3 | Predicted | 0.002 | RB-07 | GABRE | Predicted | 0.005 |
| RAB-54 | CTSB | Predicted | 0.111 | RB-07 | GAPDH | Predicted | 0.040 |
| RAB-54 | ESR1 | Predicted | 0.448 | RB-07 | GLUD1 | Predicted | 0.038 |
| RAB-54 | FGF1 | Predicted | 0.018 | RB-07 | GNMT | Predicted | 0.008 |
| RAB-54 | FGF2 | Predicted | 0.018 | RB-07 | GSTP1 | Predicted | 0.044 |
| RAB-54 | GABRE | Predicted | 0.057 | RB-07 | HAO1 | Predicted | 0.000 |
| RAB-54 | GLUD1 | Predicted | 0.002 | RB-07 | KRAS | Predicted | 0.126 |
| RAB-54 | GNAI1 | Predicted | 0.001 | RB-07 | LYZ | Predicted | 0.090 |
| RAB-54 | GSTP1 | Predicted | 0.000 | RB-07 | MAPK1 | Predicted | 0.088 |
| RAB-54 | HGF | Predicted | 0.018 | RB-07 | MAPK3 | Predicted | 0.047 |
| RAB-54 | HSP90AA1 | Predicted | 0.002 | RB-07 | MET | Predicted | 0.188 |
| RAB-54 | HSP90B1 | Predicted | 0.002 | RB-07 | MME | Predicted | 0.129 |
| RAB-54 | HSPA1B | Predicted | 0.002 | RB-07 | MMP12 | Predicted | 0.165 |
| RAB-54 | HSPA8 | Predicted | 0.002 | RB-07 | MMP9 | Predicted | 0.037 |
| RAB-54 | KRAS | Predicted | 0.037 | RB-07 | NME1 | Predicted | 0.188 |
| RAB-54 | LYZ | Predicted | 0.005 | RB-07 | NOS2A | Predicted | 0.038 |
| RAB-54 | MAT1A | Predicted | 0.002 | RB-07 | PTGS2 | Predicted | 0.078 |
| RAB-54 | MMP12 | Predicted | 0.008 | RB-07 | RND3 | Predicted | 0.038 |
| RAB-54 | MMP14 | Predicted | 0.008 | RB-07 | SGK1 | Predicted | 0.000 |
| RAB-54 | MMP2 | Predicted | 0.008 | RB-07 | SRC | Predicted | 0.093 |
| RAB-54 | MMP3 | Predicted | 0.021 | RB-07 | SULT2A1 | Predicted | 0.035 |
| RAB-54 | MMP7 | Predicted | 0.008 | RB-07 | TOP2A | Predicted | 0.067 |
| RAB-54 | MMP9 | Predicted | 0.008 | RB-07 | TRDMT1 | Predicted | 0.008 |

| RAB-54 | MTAP | Predicted | 0.109 | RB-07 | VEGFA | Predicted | 0.081 |
| --- | --- | --- | --- | --- | --- | --- | --- |
| RAB-54 | NME1 | Predicted | 0.009 | RB-08 | ABCB1 | Predicted | 0.006 |
| RAB-54 | NME2 | Predicted | 0.001 | RB-08 | ACSL1 | Predicted | 0.006 |
| RAB-54 | NOS2A | Predicted | 0.109 | RB-08 | ADH1C | Predicted | 0.006 |
| RAB-54 | PC | Predicted | 0.041 | RB-08 | AGXT | Predicted | 0.006 |
| RAB-54 | PGK1 | Predicted | 0.002 | RB-08 | AKR1B10 | Predicted | 0.096 |
| RAB-54 | PTGS2 | Predicted | 1.000 | RB-08 | AKT2 | Predicted | 0.128 |
| RAB-54 | PTK2 | Predicted | 0.002 | RB-08 | ASS1 | Predicted | 0.006 |
| RAB-54 | RHOA | Predicted | 0.001 | RB-08 | AURKA | Predicted | 0.005 |
| RAB-54 | SULT2A1 | Predicted | 0.093 | RB-08 | CA1 | Predicted | 0.047 |
| RAB-54 | TGM2 | Predicted | 0.001 | RB-08 | CA2 | Predicted | 0.047 |
| RAB-54 | TNFSF11 | Predicted | 0.033 | RB-08 | CASP1 | Predicted | 0.028 |
| RAB-54 | TOP2A | Predicted | 0.112 | RB-08 | CCNA2 | Predicted | 1.000 |
| RAB-55 | ABCB1 | Predicted | 0.003 | RB-08 | CCT3 | Predicted | 0.005 |
| RAB-55 | ACSL1 | Predicted | 0.003 | RB-08 | CDK4 | Predicted | 0.042 |
| RAB-55 | AKT2 | Predicted | 0.241 | RB-08 | CES1 | Predicted | 0.006 |
| RAB-55 | ASS1 | Predicted | 0.003 | RB-08 | CTSB | Predicted | 0.097 |
| RAB-55 | AURKA | Predicted | 0.003 | RB-08 | ESR1 | Predicted | 0.182 |
| RAB-55 | CCNA2 | Predicted | 0.962 | RB-08 | GABRE | Predicted | 0.001 |
| RAB-55 | CCT3 | Predicted | 0.017 | RB-08 | GLUD1 | Predicted | 0.017 |
| RAB-55 | CES1 | Predicted | 0.045 | RB-08 | GNAI1 | Predicted | 0.005 |
| RAB-55 | CTSB | Predicted | 0.156 | RB-08 | GNMT | Predicted | 0.003 |
| RAB-55 | CYP2C8 | Predicted | 0.005 | RB-08 | GSTP1 | Predicted | 0.030 |
| RAB-55 | ESR1 | Predicted | 0.150 | RB-08 | HAO1 | Predicted | 0.000 |
| RAB-55 | GAPDH | Predicted | 0.015 | RB-08 | HSP90AA1 | Predicted | 0.005 |

| RAB-55 | GLUD1 | Predicted | 0.027 | RB-08 | HSP90B1 | Predicted | 0.005 |
| --- | --- | --- | --- | --- | --- | --- | --- |
| RAB-55 | GNMT | Predicted | 0.005 | RB-08 | HSPA1B | Predicted | 0.005 |
| RAB-55 | HSP90AA1 | Predicted | 0.003 | RB-08 | HSPA8 | Predicted | 0.005 |
| RAB-55 | HSP90B1 | Predicted | 0.003 | RB-08 | KRAS | Predicted | 0.030 |
| RAB-55 | HSPA1B | Predicted | 0.003 | RB-08 | MAPK1 | Predicted | 0.089 |
| RAB-55 | HSPA8 | Predicted | 0.003 | RB-08 | MAPK3 | Predicted | 0.042 |
| RAB-55 | KRAS | Predicted | 0.081 | RB-08 | MAT1A | Predicted | 0.005 |
| RAB-55 | MAPK1 | Predicted | 0.061 | RB-08 | MME | Predicted | 0.064 |
| RAB-55 | MAT1A | Predicted | 0.003 | RB-08 | MMP12 | Predicted | 0.030 |
| RAB-55 | MME | Predicted | 0.096 | RB-08 | MMP9 | Predicted | 0.028 |
| RAB-55 | MMP12 | Predicted | 0.048 | RB-08 | NME1 | Predicted | 0.025 |
| RAB-55 | MMP14 | Predicted | 0.013 | RB-08 | NME2 | Predicted | 0.005 |
| RAB-55 | MMP2 | Predicted | 0.013 | RB-08 | PGK1 | Predicted | 0.005 |
| RAB-55 | MMP3 | Predicted | 0.041 | RB-08 | PLAU | Predicted | 0.029 |
| RAB-55 | MMP7 | Predicted | 0.013 | RB-08 | PTK2 | Predicted | 0.005 |
| RAB-55 | MMP9 | Predicted | 0.013 | RB-08 | RHOA | Predicted | 0.005 |
| RAB-55 | NME1 | Predicted | 0.003 | RB-08 | RND3 | Predicted | 0.008 |
| RAB-55 | NOS2A | Predicted | 0.048 | RB-08 | SERPINE1 | Predicted | 0.015 |
| RAB-55 | PGK1 | Predicted | 0.003 | RB-08 | SGK1 | Predicted | 0.000 |
| RAB-55 | PTGS2 | Predicted | 0.090 | RB-08 | SRC | Predicted | 0.094 |
| RAB-55 | PTK2 | Predicted | 0.003 | RB-08 | TGM2 | Predicted | 0.005 |
| RAB-55 | RND3 | Predicted | 0.012 | RB-08 | TOP2A | Predicted | 0.052 |
| RAB-55 | SRC | Predicted | 0.072 | RB-08 | TRDMT1 | Predicted | 0.003 |
| RAB-55 | SULT2A1 | Predicted | 0.011 | RB-08 | VEGFA | Predicted | 0.060 |
| RAB-55 | TOP2A | Predicted | 0.028 | RB-09 | ABCB1 | Predicted | 0.002 |

| RAB-55 | TRDMT1 | Predicted | 0.005 | RB-09 | ACSL1 | Predicted | 0.002 |
| --- | --- | --- | --- | --- | --- | --- | --- |
| RAB-56 | A2M | Predicted | 0.165 | RB-09 | AKT2 | Predicted | 0.205 |
| RAB-56 | AGXT | Predicted | 0.238 | RB-09 | ASS1 | Predicted | 0.002 |
| RAB-56 | AKR1B10 | Predicted | 0.228 | RB-09 | AURKA | Predicted | 0.004 |
| RAB-56 | ALB | Predicted | 0.335 | RB-09 | CCNA2 | Predicted | 0.853 |
| RAB-56 | ALDOB | Predicted | 0.257 | RB-09 | CCT3 | Predicted | 0.019 |
| RAB-56 | APOA1 | Predicted | 0.219 | RB-09 | CES1 | Predicted | 0.040 |
| RAB-56 | ARG1 | Predicted | 0.217 | RB-09 | CTSB | Predicted | 0.136 |
| RAB-56 | ASS1 | Predicted | 0.164 | RB-09 | ESR1 | Predicted | 0.272 |
| RAB-56 | CA2 | Predicted | 0.164 | RB-09 | FGF1 | Predicted | 0.060 |
| RAB-56 | CAP2 | Predicted | 0.163 | RB-09 | FGF2 | Predicted | 0.060 |
| RAB-56 | CCT3 | Predicted | 0.210 | RB-09 | GAPDH | Predicted | 0.021 |
| RAB-56 | CDKN1A | Predicted | 0.163 | RB-09 | GLUD1 | Predicted | 0.030 |
| RAB-56 | CLIC1 | Predicted | 0.167 | RB-09 | GNMT | Predicted | 0.004 |
| RAB-56 | CPS1 | Predicted | 0.257 | RB-09 | GSTP1 | Predicted | 0.030 |
| RAB-56 | CTGF | Predicted | 0.163 | RB-09 | HAO1 | Predicted | 0.000 |
| RAB-56 | CYP2E1 | Predicted | 0.332 | RB-09 | HSP90AA1 | Predicted | 0.004 |
| RAB-56 | ETS2 | Predicted | 0.163 | RB-09 | HSP90B1 | Predicted | 0.004 |
| RAB-56 | GLUL | Predicted | 0.167 | RB-09 | HSPA1B | Predicted | 0.004 |
| RAB-56 | GPC3 | Predicted | 0.338 | RB-09 | HSPA8 | Predicted | 0.004 |
| RAB-56 | HMMR | Predicted | 0.167 | RB-09 | KRAS | Predicted | 0.071 |
| RAB-56 | HSP90B1 | Predicted | 0.166 | RB-09 | LYZ | Predicted | 0.062 |
| RAB-56 | HSPA5 | Predicted | 0.255 | RB-09 | MAPK1 | Predicted | 0.055 |
| RAB-56 | HSPB1 | Predicted | 0.210 | RB-09 | MAT1A | Predicted | 0.004 |
| RAB-56 | IGF2 | Predicted | 0.194 | RB-09 | MME | Predicted | 0.087 |

| RAB-56 | IGFBP3 | Predicted | 0.250 | RB-09 | MMP12 | Predicted | 0.041 |
| --- | --- | --- | --- | --- | --- | --- | --- |
| RAB-56 | PLG | Predicted | 0.334 | RB-09 | MMP14 | Predicted | 0.011 |
| RAB-56 | SPARC | Predicted | 0.273 | RB-09 | MMP2 | Predicted | 0.011 |
| RAB-56 | STMN1 | Predicted | 0.165 | RB-09 | MMP3 | Predicted | 0.035 |
| RAB-56 | SULT2A1 | Predicted | 0.180 | RB-09 | MMP7 | Predicted | 0.011 |
| RAB-56 | TOP2A | Predicted | 0.165 | RB-09 | MMP9 | Predicted | 0.011 |
| RAB-56 | UBD | Predicted | 0.178 | RB-09 | NME1 | Predicted | 0.019 |
| RAB-57 | ADH1B | Predicted | 0.012 | RB-09 | NOS2A | Predicted | 0.035 |
| RAB-57 | ADH1C | Predicted | 0.006 | RB-09 | PGK1 | Predicted | 0.004 |
| RAB-57 | ADH4 | Predicted | 0.006 | RB-09 | PTGS2 | Predicted | 0.063 |
| RAB-57 | AKR1B10 | Predicted | 0.000 | RB-09 | PTK2 | Predicted | 0.004 |
| RAB-57 | AKR1C2 | Predicted | 0.006 | RB-09 | RND3 | Predicted | 0.016 |
| RAB-57 | ALDH1A1 | Predicted | 0.006 | RB-09 | SERPINE1 | Predicted | 0.024 |
| RAB-57 | ALDH1B1 | Predicted | 0.000 | RB-09 | SGK1 | Predicted | 0.000 |
| RAB-57 | ALDH2 | Predicted | 0.006 | RB-09 | SRC | Predicted | 0.066 |
| RAB-57 | ALDH3A2 | Predicted | 0.000 | RB-09 | SULT2A1 | Predicted | 0.010 |
| RAB-57 | ALDH4A1 | Predicted | 0.000 | RB-09 | TOP2A | Predicted | 0.082 |
| RAB-57 | CDK4 | Predicted | 0.035 | RB-09 | TRDMT1 | Predicted | 0.004 |
| RAB-57 | DCXR | Predicted | 0.000 | RB-09 | VEGFA | Predicted | 0.027 |
| RAB-57 | GAPDH | Predicted | 0.006 | RB-10 | NOS2A | Validated | 1.000 |
| RAB-57 | GLUD1 | Predicted | 0.006 | RB-10 | ACSL1 | Predicted | 0.025 |
| RAB-57 | GSTP1 | Predicted | 0.204 | RB-10 | ALDH2 | Predicted | 0.085 |
| RAB-57 | HSD17B10 | Predicted | 0.006 | RB-10 | ALDOA | Predicted | 0.086 |
| RAB-57 | HSD17B4 | Predicted | 0.006 | RB-10 | AURKA | Predicted | 0.083 |
| RAB-57 | MAPK1 | Predicted | 0.035 | RB-10 | CA1 | Predicted | 0.096 |

| RAB-57 | MAPK3 | Predicted | 0.035 | RB-10 | CA2 | Predicted | 0.063 |
| --- | --- | --- | --- | --- | --- | --- | --- |
| RAB-57 | MME | Predicted | 0.199 | RB-10 | CCNA2 | Predicted | 0.351 |
| RAB-57 | MMP12 | Predicted | 0.048 | RB-10 | CTSB | Predicted | 0.084 |
| RAB-57 | MTHFD1 | Predicted | 0.006 | RB-10 | ESR1 | Predicted | 0.458 |
| RAB-57 | QDPR | Predicted | 0.006 | RB-10 | GABRE | Predicted | 0.015 |
| RAB-58 | ABCB1 | Predicted | 0.008 | RB-10 | GSTP1 | Predicted | 0.000 |
| RAB-58 | ACSL1 | Predicted | 0.008 | RB-10 | KRAS | Predicted | 0.007 |
| RAB-58 | AKT2 | Predicted | 0.018 | RB-10 | LCN2 | Predicted | 0.083 |
| RAB-58 | ALDH1L1 | Predicted | 0.010 | RB-10 | MTAP | Predicted | 0.176 |
| RAB-58 | ASS1 | Predicted | 0.008 | RB-10 | PTGS2 | Predicted | 0.941 |
| RAB-58 | AURKA | Predicted | 0.119 | RB-10 | TOP2A | Predicted | 0.055 |
| RAB-58 | CA1 | Predicted | 0.021 | RB-11 | ABCB1 | Predicted | 0.019 |
| RAB-58 | CA2 | Predicted | 0.021 | RB-11 | ACSL1 | Predicted | 0.019 |
| RAB-58 | CCNA2 | Predicted | 0.904 | RB-11 | ADH1B | Predicted | 0.027 |
| RAB-58 | CCT3 | Predicted | 0.005 | RB-11 | ADH1C | Predicted | 0.015 |
| RAB-58 | CYP2C8 | Predicted | 0.009 | RB-11 | ADH4 | Predicted | 0.015 |
| RAB-58 | ESR1 | Predicted | 0.037 | RB-11 | AKR1B10 | Predicted | 0.000 |
| RAB-58 | FTCD | Predicted | 0.010 | RB-11 | AKR1C2 | Predicted | 0.012 |
| RAB-58 | GLUD1 | Predicted | 0.011 | RB-11 | AKT2 | Predicted | 0.147 |
| RAB-58 | GNMT | Predicted | 0.002 | RB-11 | ALDH1A1 | Predicted | 0.015 |
| RAB-58 | GSTP1 | Predicted | 0.020 | RB-11 | ALDH1B1 | Predicted | 0.001 |
| RAB-58 | KRAS | Predicted | 0.025 | RB-11 | ALDH2 | Predicted | 0.015 |
| RAB-58 | LYZ | Predicted | 0.128 | RB-11 | ALDH3A2 | Predicted | 0.001 |
| RAB-58 | MAPK1 | Predicted | 0.010 | RB-11 | ALDH4A1 | Predicted | 0.001 |
| RAB-58 | MTHFD1 | Predicted | 0.010 | RB-11 | ASS1 | Predicted | 0.019 |

| RAB-58 | MTR | Predicted | 0.010 | RB-11 | CCNA2 | Predicted | 0.252 |
| --- | --- | --- | --- | --- | --- | --- | --- |
| RAB-58 | NME1 | Predicted | 0.127 | RB-11 | CCT3 | Predicted | 0.008 |
| RAB-58 | NOS2A | Predicted | 0.123 | RB-11 | CDK4 | Predicted | 0.098 |
| RAB-58 | PTGS2 | Predicted | 0.067 | RB-11 | DCXR | Predicted | 0.000 |
| RAB-58 | RAF1 | Predicted | 0.015 | RB-11 | GAPDH | Predicted | 0.040 |
| RAB-58 | RARA | Predicted | 0.055 | RB-11 | GLUD1 | Predicted | 0.015 |
| RAB-58 | RND3 | Predicted | 0.011 | RB-11 | GSTP1 | Predicted | 0.263 |
| RAB-58 | SERPINE1 | Predicted | 0.037 | RB-11 | HSD17B10 | Predicted | 0.015 |
| RAB-58 | TRDMT1 | Predicted | 0.002 | RB-11 | HSD17B4 | Predicted | 0.015 |
| RAB-58 | VEGFA | Predicted | 0.028 | RB-11 | MAPK1 | Predicted | 0.098 |
| RAB-59 | ADH1B | Predicted | 0.019 | RB-11 | MAPK3 | Predicted | 0.098 |
| RAB-59 | ADH1C | Predicted | 0.010 | RB-11 | MME | Predicted | 0.249 |
| RAB-59 | ADH4 | Predicted | 0.010 | RB-11 | MTHFD1 | Predicted | 0.012 |
| RAB-59 | AKR1B10 | Predicted | 0.000 | RB-11 | QDPR | Predicted | 0.015 |
| RAB-59 | AKR1C2 | Predicted | 0.009 | RB-11 | TOP2A | Predicted | 0.334 |
| RAB-59 | ALDH1A1 | Predicted | 0.010 | RB-11 | VEGFA | Predicted | 0.037 |
| RAB-59 | ALDH1B1 | Predicted | 0.000 | RB-12 | ACSL1 | Predicted | 0.024 |
| RAB-59 | ALDH2 | Predicted | 0.010 | RB-12 | ALDH2 | Predicted | 0.086 |
| RAB-59 | ALDH3A2 | Predicted | 0.000 | RB-12 | ALDOA | Predicted | 0.085 |
| RAB-59 | ALDH4A1 | Predicted | 0.000 | RB-12 | AR | Predicted | 0.083 |
| RAB-59 | DCXR | Predicted | 0.000 | RB-12 | CA1 | Predicted | 0.219 |
| RAB-59 | ESR1 | Predicted | 0.327 | RB-12 | CA2 | Predicted | 0.130 |
| RAB-59 | GAPDH | Predicted | 0.010 | RB-12 | CASP3 | Validated | 1.000 |
| RAB-59 | GLUD1 | Predicted | 0.010 | RB-12 | CCNA2 | Predicted | 0.261 |
| RAB-59 | GSTP1 | Predicted | 0.324 | RB-12 | CYP1A2 | Validated | 1.000 |

| RAB-59 | HSD17B10 | Predicted | 0.010 | RB-12 | CYP3A4 | Validated | 1.000 |
| --- | --- | --- | --- | --- | --- | --- | --- |
| RAB-59 | HSD17B4 | Predicted | 0.010 | RB-12 | ESR1 | Validated | 1.000 |
| RAB-59 | MME | Predicted | 0.339 | RB-12 | GABRE | Predicted | 0.008 |
| RAB-59 | MMP12 | Predicted | 0.077 | RB-12 | GSTP1 | Validated | 1.000 |
| RAB-59 | MTHFD1 | Predicted | 0.009 | RB-12 | HAS2 | Validated | 1.000 |
| RAB-59 | QDPR | Predicted | 0.010 | RB-12 | ICAM1 | Validated | 1.000 |
| RAB-60 | ALDH1L1 | Predicted | 0.000 | RB-12 | KRAS | Predicted | 0.007 |
| RAB-60 | AURKA | Predicted | 0.318 | RB-12 | LCN2 | Predicted | 0.085 |
| RAB-60 | FTCD | Predicted | 0.000 | RB-12 | MAPK1 | Predicted | 0.035 |
| RAB-60 | GAPDH | Predicted | 0.041 | RB-12 | MTAP | Predicted | 0.174 |
| RAB-60 | MTHFD1 | Predicted | 0.000 | RB-12 | NOS2A | Predicted | 0.174 |
| RAB-60 | MTR | Predicted | 0.000 | RB-12 | PC | Predicted | 0.007 |
| RAB-60 | RARA | Predicted | 0.050 | RB-12 | PTGS2 | Predicted | 0.876 |
| RAB-60 | SRC | Predicted | 0.051 | RB-12 | STAT1 | Validated | 1.000 |
| RAB-61 | ALDH1L1 | Predicted | 0.014 | RB-12 | TOP2A | Predicted | 0.041 |
| RAB-61 | AURKA | Predicted | 0.242 | RB-12 | TP53 | Validated | 1.000 |
| RAB-61 | DDR1 | Predicted | 0.022 | RB-12 | VEGFA | Validated | 1.000 |
| RAB-61 | FTCD | Predicted | 0.014 | RB-13 | ABCB1 | Predicted | 0.015 |
| RAB-61 | MTHFD1 | Predicted | 0.014 | RB-13 | ACSL1 | Predicted | 0.015 |
| RAB-61 | MTR | Predicted | 0.014 | RB-13 | ADH1C | Predicted | 0.012 |
| RAB-61 | PDGFRA | Predicted | 0.022 | RB-13 | AGXT | Predicted | 0.014 |
| RAB-61 | RAF1 | Predicted | 0.026 | RB-13 | AKR1B10 | Predicted | 0.193 |
| RAB-61 | RARA | Predicted | 0.041 | RB-13 | AKT2 | Predicted | 0.277 |
| RAB-61 | SRC | Predicted | 0.023 | RB-13 | ASS1 | Predicted | 0.015 |
| RAB-62 | ABCG2 | Validated | 1.000 | RB-13 | AURKA | Predicted | 0.013 |

| RAB-62 | AR | Validated | 1.000 | RB-13 | CA1 | Predicted | 0.093 |
| --- | --- | --- | --- | --- | --- | --- | --- |
| RAB-62 | CASP3 | Validated | 1.000 | RB-13 | CA2 | Predicted | 0.093 |
| RAB-62 | CAT | Validated | 1.000 | RB-13 | CCNA2 | Predicted | 0.780 |
| RAB-62 | CDKN1A | Validated | 1.000 | RB-13 | CCT3 | Predicted | 0.013 |
| RAB-62 | CDKN2A | Validated | 1.000 | RB-13 | CDK4 | Predicted | 0.057 |
| RAB-62 | CLDN4 | Validated | 1.000 | RB-13 | CES1 | Predicted | 0.012 |
| RAB-62 | COL1A1 | Validated | 1.000 | RB-13 | CTSB | Predicted | 0.200 |
| RAB-62 | CXCL2 | Validated | 1.000 | RB-13 | ESR1 | Predicted | 0.593 |
| RAB-62 | CYP3A4 | Validated | 1.000 | RB-13 | FGF1 | Predicted | 0.095 |
| RAB-62 | E2F1 | Validated | 1.000 | RB-13 | FGF2 | Predicted | 0.095 |
| RAB-62 | EGF | Validated | 1.000 | RB-13 | GABRE | Predicted | 0.027 |
| RAB-62 | ESR1 | Validated | 1.000 | RB-13 | GLUD1 | Predicted | 0.037 |
| RAB-62 | FOS | Validated | 1.000 | RB-13 | GNAI1 | Predicted | 0.013 |
| RAB-62 | GSTP1 | Validated | 1.000 | RB-13 | GNMT | Predicted | 0.009 |
| RAB-62 | HAS2 | Validated | 1.000 | RB-13 | GSTP1 | Predicted | 0.057 |
| RAB-62 | HSPA5 | Validated | 1.000 | RB-13 | HAO1 | Predicted | 0.000 |
| RAB-62 | HSPB1 | Validated | 1.000 | RB-13 | HSP90AA1 | Predicted | 0.013 |
| RAB-62 | ICAM1 | Validated | 1.000 | RB-13 | HSP90B1 | Predicted | 0.013 |
| RAB-62 | IGF2 | Validated | 1.000 | RB-13 | HSPA1B | Predicted | 0.013 |
| RAB-62 | IGFBP3 | Validated | 1.000 | RB-13 | HSPA8 | Predicted | 0.013 |
| RAB-62 | IL2 | Validated | 1.000 | RB-13 | KRAS | Predicted | 0.061 |
| RAB-62 | IRF1 | Validated | 1.000 | RB-13 | MAPK1 | Predicted | 0.122 |
| RAB-62 | MMP9 | Validated | 1.000 | RB-13 | MAPK3 | Predicted | 0.116 |
| RAB-62 | MYC | Validated | 1.000 | RB-13 | MAT1A | Predicted | 0.013 |
| RAB-62 | NFKBIA | Validated | 1.000 | RB-13 | MET | Predicted | 0.189 |

| RAB-62 | NOS2A | Validated | 1.000 | RB-13 | MME | Predicted | 0.134 |
| --- | --- | --- | --- | --- | --- | --- | --- |
| RAB-62 | PARP1 | Validated | 1.000 | RB-13 | MMP12 | Predicted | 0.063 |
| RAB-62 | PLAU | Validated | 1.000 | RB-13 | NME1 | Predicted | 0.051 |
| RAB-62 | PTENP1 | Validated | 1.000 | RB-13 | NME2 | Predicted | 0.013 |
| RAB-62 | PTGS2 | Validated | 1.000 | RB-13 | PGK1 | Predicted | 0.013 |
| RAB-62 | RAF1 | Validated | 1.000 | RB-13 | PTGS2 | Predicted | 0.109 |
| RAB-62 | SERPINE1 | Validated | 1.000 | RB-13 | PTK2 | Predicted | 0.013 |
| RAB-62 | SPP1 | Validated | 1.000 | RB-13 | RHOA | Predicted | 0.013 |
| RAB-62 | STAT1 | Validated | 1.000 | RB-13 | RND3 | Predicted | 0.017 |
| RAB-62 | TOP2A | Validated | 1.000 | RB-13 | SERPINE1 | Predicted | 0.030 |
| RAB-62 | VEGFA | Validated | 1.000 | RB-13 | SGK1 | Predicted | 0.000 |
| RAB-62 | ACSL1 | Predicted | 0.026 | RB-13 | SRC | Predicted | 0.256 |
| RAB-62 | ALDH2 | Predicted | 0.085 | RB-13 | SULT2A1 | Predicted | 0.021 |
| RAB-62 | ALDOA | Predicted | 0.085 | RB-13 | TGM2 | Predicted | 0.013 |
| RAB-62 | CA1 | Predicted | 0.176 | RB-13 | TOP2A | Predicted | 0.119 |
| RAB-62 | CA2 | Predicted | 0.086 | RB-13 | TRDMT1 | Predicted | 0.009 |
| RAB-62 | CCNA2 | Predicted | 0.262 | RB-13 | VEGFA | Predicted | 0.049 |
| RAB-62 | CTSB | Predicted | 0.082 | RB-14 | ABCB1 | Predicted | 0.012 |
| RAB-62 | GABRE | Predicted | 0.010 | RB-14 | ACSL1 | Predicted | 0.012 |
| RAB-62 | KRAS | Predicted | 0.007 | RB-14 | ADH1B | Predicted | 0.003 |
| RAB-62 | LCN2 | Predicted | 0.087 | RB-14 | ADH1C | Predicted | 0.022 |
| RAB-62 | MTAP | Predicted | 0.175 | RB-14 | ADH4 | Predicted | 0.003 |
| RAB-63 | ALDH1L1 | Predicted | 0.008 | RB-14 | AGXT | Predicted | 0.011 |
| RAB-63 | DDR1 | Predicted | 0.065 | RB-14 | AKR1B10 | Predicted | 0.191 |
| RAB-63 | FTCD | Predicted | 0.008 | RB-14 | AKT2 | Predicted | 0.226 |

| RAB-63 | MME | Predicted | 0.334 | RB-14 | ALDH1A1 | Predicted | 0.003 |
| --- | --- | --- | --- | --- | --- | --- | --- |
| RAB-63 | MTHFD1 | Predicted | 0.008 | RB-14 | ALDH2 | Predicted | 0.003 |
| RAB-63 | MTR | Predicted | 0.008 | RB-14 | ASS1 | Predicted | 0.012 |
| RAB-63 | PDGFRA | Predicted | 0.065 | RB-14 | AURKA | Predicted | 0.010 |
| RAB-63 | RAF1 | Predicted | 0.043 | RB-14 | CASP1 | Predicted | 0.038 |
| RAB-64 | ACSL1 | Predicted | 0.024 | RB-14 | CCNA2 | Predicted | 0.994 |
| RAB-64 | ALDH2 | Predicted | 0.086 | RB-14 | CCT3 | Predicted | 0.030 |
| RAB-64 | ALDOA | Predicted | 0.085 | RB-14 | CDK4 | Predicted | 0.060 |
| RAB-64 | AR | Predicted | 0.083 | RB-14 | CES1 | Predicted | 0.010 |
| RAB-64 | CA1 | Predicted | 0.219 | RB-14 | CTSB | Predicted | 0.189 |
| RAB-64 | CA2 | Predicted | 0.130 | RB-14 | ESR1 | Predicted | 0.395 |
| RAB-64 | CASP3 | Validated | 1.000 | RB-14 | GABRE | Predicted | 0.002 |
| RAB-64 | CCNA2 | Predicted | 0.261 | RB-14 | GAPDH | Predicted | 0.029 |
| RAB-64 | CYP1A2 | Validated | 1.000 | RB-14 | GLUD1 | Predicted | 0.043 |
| RAB-64 | CYP3A4 | Validated | 1.000 | RB-14 | GNAI1 | Predicted | 0.010 |
| RAB-64 | ESR1 | Validated | 1.000 | RB-14 | GNMT | Predicted | 0.006 |
| RAB-64 | GABRE | Predicted | 0.008 | RB-14 | GSTP1 | Predicted | 0.052 |
| RAB-64 | GSTP1 | Validated | 1.000 | RB-14 | HAO1 | Predicted | 0.000 |
| RAB-64 | HAS2 | Validated | 1.000 | RB-14 | HSD17B10 | Predicted | 0.003 |
| RAB-64 | ICAM1 | Validated | 1.000 | RB-14 | HSD17B4 | Predicted | 0.003 |
| RAB-64 | KRAS | Predicted | 0.007 | RB-14 | HSP90AA1 | Predicted | 0.010 |
| RAB-64 | LCN2 | Predicted | 0.085 | RB-14 | HSP90B1 | Predicted | 0.010 |
| RAB-64 | MAPK1 | Predicted | 0.035 | RB-14 | HSPA1B | Predicted | 0.010 |
| RAB-64 | MTAP | Predicted | 0.174 | RB-14 | HSPA8 | Predicted | 0.010 |
| RAB-64 | NOS2A | Predicted | 0.174 | RB-14 | KRAS | Predicted | 0.050 |

| RAB-64 | PC | Predicted | 0.007 | RB-14 | MAPK1 | Predicted | 0.168 |
| --- | --- | --- | --- | --- | --- | --- | --- |
| RAB-64 | PTGS2 | Predicted | 0.876 | RB-14 | MAPK3 | Predicted | 0.060 |
| RAB-64 | STAT1 | Validated | 1.000 | RB-14 | MAT1A | Predicted | 0.010 |
| RAB-64 | TOP2A | Predicted | 0.041 | RB-14 | MME | Predicted | 0.298 |
| RAB-64 | TP53 | Validated | 1.000 | RB-14 | MMP12 | Predicted | 0.077 |
| RAB-64 | VEGFA | Validated | 1.000 | RB-14 | MMP9 | Predicted | 0.038 |
| RAB-65 | ACSL1 | Predicted | 0.024 | RB-14 | NME1 | Predicted | 0.047 |
| RAB-65 | ALDH2 | Predicted | 0.081 | RB-14 | NME2 | Predicted | 0.010 |
| RAB-65 | ALDOA | Predicted | 0.082 | RB-14 | PGK1 | Predicted | 0.010 |
| RAB-65 | AR | Predicted | 0.081 | RB-14 | PTGS2 | Predicted | 0.043 |
| RAB-65 | B2M | Predicted | 0.017 | RB-14 | PTK2 | Predicted | 0.010 |
| RAB-65 | CA1 | Predicted | 0.182 | RB-14 | QDPR | Predicted | 0.003 |
| RAB-65 | CA2 | Predicted | 0.204 | RB-14 | RHOA | Predicted | 0.010 |
| RAB-65 | CCNA2 | Predicted | 0.170 | RB-14 | RND3 | Predicted | 0.012 |
| RAB-65 | CDKN1A | Validated | 1.000 | RB-14 | SERPINE1 | Predicted | 0.026 |
| RAB-65 | ESR1 | Predicted | 0.114 | RB-14 | SGK1 | Predicted | 0.000 |
| RAB-65 | FN1 | Validated | 1.000 | RB-14 | SRC | Predicted | 0.179 |
| RAB-65 | GABRE | Predicted | 0.009 | RB-14 | TGM2 | Predicted | 0.010 |
| RAB-65 | GSTP1 | Predicted | 0.000 | RB-14 | TOP2A | Predicted | 0.053 |
| RAB-65 | KRAS | Predicted | 0.007 | RB-14 | TRDMT1 | Predicted | 0.006 |
| RAB-65 | LCN2 | Predicted | 0.081 | RB-14 | VEGFA | Predicted | 0.084 |
| RAB-65 | MAPK1 | Predicted | 0.027 | RB-15 | ABCB1 | Predicted | 0.010 |
| RAB-65 | MCL1 | Validated | 1.000 | RB-15 | ACSL1 | Predicted | 0.010 |
| RAB-65 | MMP12 | Predicted | 0.024 | RB-15 | ADH1B | Predicted | 0.009 |
| RAB-65 | MMP3 | Predicted | 0.024 | RB-15 | ADH1C | Predicted | 0.009 |

| RAB-65 | MTAP | Predicted | 0.174 | RB-15 | ADH4 | Predicted | 0.009 |
| --- | --- | --- | --- | --- | --- | --- | --- |
| RAB-65 | NOS2A | Validated | 1.000 | RB-15 | AGXT | Predicted | 0.006 |
| RAB-65 | PARP1 | Predicted | 0.083 | RB-15 | AKR1B10 | Predicted | 0.132 |
| RAB-65 | PC | Predicted | 0.006 | RB-15 | AKR1C2 | Predicted | 0.000 |
| RAB-65 | PTGS2 | Predicted | 1.000 | RB-15 | AKT2 | Predicted | 0.104 |
| RAB-65 | SRC | Predicted | 0.022 | RB-15 | ALDH1A1 | Predicted | 0.009 |
| RAB-65 | TNFSF11 | Predicted | 0.024 | RB-15 | ALDH1B1 | Predicted | 0.000 |
| RAB-66 | AGXT | Predicted | 0.003 | RB-15 | ALDH2 | Predicted | 0.009 |
| RAB-66 | ALAS1 | Predicted | 0.003 | RB-15 | ALDH3A2 | Predicted | 0.000 |
| RAB-66 | ALDOA | Predicted | 0.086 | RB-15 | ALDH4A1 | Predicted | 0.000 |
| RAB-66 | AR | Predicted | 0.086 | RB-15 | ASS1 | Predicted | 0.010 |
| RAB-66 | ARG1 | Predicted | 0.039 | RB-15 | AURKA | Predicted | 0.006 |
| RAB-66 | B2M | Predicted | 0.028 | RB-15 | CA1 | Predicted | 0.020 |
| RAB-66 | CA1 | Predicted | 0.194 | RB-15 | CA2 | Predicted | 0.020 |
| RAB-66 | CA2 | Predicted | 0.214 | RB-15 | CCNA2 | Predicted | 0.567 |
| RAB-66 | CBS | Predicted | 0.003 | RB-15 | CCT3 | Predicted | 0.020 |
| RAB-66 | CCNA2 | Predicted | 0.087 | RB-15 | CDK4 | Predicted | 0.042 |
| RAB-66 | CTH | Predicted | 0.003 | RB-15 | ESR1 | Predicted | 0.139 |
| RAB-66 | DNMT1 | Predicted | 0.042 | RB-15 | GABRE | Predicted | 0.000 |
| RAB-66 | ESR1 | Predicted | 0.038 | RB-15 | GAPDH | Predicted | 0.028 |
| RAB-66 | FGF1 | Predicted | 0.015 | RB-15 | GLUD1 | Predicted | 0.031 |
| RAB-66 | FGF2 | Predicted | 0.015 | RB-15 | GNAI1 | Predicted | 0.001 |
| RAB-66 | FTCD | Predicted | 0.003 | RB-15 | GNMT | Predicted | 0.005 |
| RAB-66 | GABRE | Predicted | 0.019 | RB-15 | GSTP1 | Predicted | 0.134 |
| RAB-66 | GSTP1 | Predicted | 0.000 | RB-15 | HAO1 | Predicted | 0.003 |

| RAB-66 | HAO1 | Predicted | 0.086 | RB-15 | HSD17B10 | Predicted | 0.009 |
| --- | --- | --- | --- | --- | --- | --- | --- |
| RAB-66 | HGF | Predicted | 0.015 | RB-15 | HSD17B4 | Predicted | 0.009 |
| RAB-66 | KRAS | Predicted | 0.009 | RB-15 | HSP90AA1 | Predicted | 0.006 |
| RAB-66 | LCN2 | Predicted | 0.089 | RB-15 | HSP90B1 | Predicted | 0.006 |
| RAB-66 | MAPK1 | Predicted | 0.027 | RB-15 | HSPA1B | Predicted | 0.006 |
| RAB-66 | MTAP | Predicted | 0.172 | RB-15 | HSPA8 | Predicted | 0.006 |
| RAB-66 | NOS2A | Predicted | 0.202 | RB-15 | KRAS | Predicted | 0.075 |
| RAB-66 | OAT | Predicted | 0.003 | RB-15 | LYZ | Predicted | 0.064 |
| RAB-66 | PARP1 | Predicted | 0.090 | RB-15 | MAPK1 | Predicted | 0.118 |
| RAB-66 | PC | Predicted | 0.034 | RB-15 | MAPK3 | Predicted | 0.042 |
| RAB-66 | PIN1 | Predicted | 0.045 | RB-15 | MAT1A | Predicted | 0.006 |
| RAB-66 | PTGS2 | Predicted | 1.000 | RB-15 | MET | Predicted | 0.135 |
| RAB-66 | PYGB | Predicted | 0.003 | RB-15 | MME | Predicted | 0.209 |
| RAB-66 | SRC | Predicted | 0.041 | RB-15 | MMP12 | Predicted | 0.052 |
| RAB-66 | TNFSF11 | Predicted | 0.027 | RB-15 | MTHFD1 | Predicted | 0.000 |
| RAB-67 | ACSL1 | Predicted | 0.026 | RB-15 | NME1 | Predicted | 0.025 |
| RAB-67 | ALDH2 | Predicted | 0.098 | RB-15 | NME2 | Predicted | 0.001 |
| RAB-67 | ALDOA | Predicted | 0.096 | RB-15 | PGK1 | Predicted | 0.006 |
| RAB-67 | CA1 | Predicted | 0.095 | RB-15 | PTGS2 | Predicted | 0.030 |
| RAB-67 | CA2 | Predicted | 0.046 | RB-15 | PTK2 | Predicted | 0.006 |
| RAB-67 | CCNA2 | Predicted | 0.389 | RB-15 | QDPR | Predicted | 0.009 |
| RAB-67 | ESR1 | Predicted | 0.532 | RB-15 | RHOA | Predicted | 0.001 |
| RAB-67 | FABP5 | Validated | 1.000 | RB-15 | RND3 | Predicted | 0.002 |
| RAB-67 | FOS | Validated | 1.000 | RB-15 | SERPINE1 | Predicted | 0.027 |
| RAB-67 | GABRE | Predicted | 0.008 | RB-15 | SGK1 | Predicted | 0.003 |

| RAB-67 | GSTP1 | Predicted | 0.000 | RB-15 | SRC | Predicted | 0.062 |
| --- | --- | --- | --- | --- | --- | --- | --- |
| RAB-67 | IGF2 | Validated | 1.000 | RB-15 | TGM2 | Predicted | 0.001 |
| RAB-67 | KRAS | Predicted | 0.010 | RB-15 | TOP2A | Predicted | 0.120 |
| RAB-67 | LCN2 | Predicted | 0.094 | RB-15 | TRDMT1 | Predicted | 0.005 |
| RAB-67 | MMP9 | Validated | 1.000 | RB-15 | TUBB | Predicted | 0.011 |
| RAB-67 | MTAP | Predicted | 0.198 | RB-15 | VEGFA | Predicted | 0.024 |
| RAB-67 | NOS2A | Predicted | 0.098 | RB-16 | ABCB1 | Predicted | 0.002 |
| RAB-67 | PTGS2 | Predicted | 0.887 | RB-16 | ACSL1 | Predicted | 0.002 |
| RAB-67 | SRC | Validated | 1.000 | RB-16 | AGXT | Predicted | 0.005 |
| RAB-67 | TOP2A | Predicted | 0.064 | RB-16 | AKR1B10 | Predicted | 0.079 |
| RAB-67 | VEGFA | Validated | 1.000 | RB-16 | AKT2 | Predicted | 0.111 |
| RAB-68 | AGXT | Predicted | 0.009 | RB-16 | ASS1 | Predicted | 0.002 |
| RAB-68 | AKR1B10 | Predicted | 0.019 | RB-16 | AURKA | Predicted | 0.004 |
| RAB-68 | ALB | Predicted | 0.090 | RB-16 | CA1 | Predicted | 0.013 |
| RAB-68 | ALDOB | Predicted | 0.088 | RB-16 | CA2 | Predicted | 0.013 |
| RAB-68 | APOA1 | Predicted | 0.089 | RB-16 | CCNA2 | Predicted | 1.000 |
| RAB-68 | ARG1 | Predicted | 0.152 | RB-16 | CCT3 | Predicted | 0.011 |
| RAB-68 | CCT3 | Predicted | 0.087 | RB-16 | CDK4 | Predicted | 0.029 |
| RAB-68 | CLIC1 | Predicted | 0.187 | RB-16 | CTSB | Predicted | 0.081 |
| RAB-68 | CPS1 | Predicted | 0.042 | RB-16 | ESR1 | Predicted | 0.165 |
| RAB-68 | CYP2E1 | Predicted | 0.045 | RB-16 | GLUD1 | Predicted | 0.012 |
| RAB-68 | GPC3 | Predicted | 0.170 | RB-16 | GNAI1 | Predicted | 0.003 |
| RAB-68 | HSPA5 | Predicted | 0.090 | RB-16 | GNMT | Predicted | 0.005 |
| RAB-68 | HSPB1 | Predicted | 0.021 | RB-16 | GSTP1 | Predicted | 0.025 |
| RAB-68 | IGF2 | Predicted | 0.038 | RB-16 | HAO1 | Predicted | 0.002 |

| RAB-68 | IGFBP3 | Predicted | 0.179 | RB-16 | HSP90AA1 | Predicted | 0.004 |
| --- | --- | --- | --- | --- | --- | --- | --- |
| RAB-68 | PLG | Predicted | 0.028 | RB-16 | HSP90B1 | Predicted | 0.004 |
| RAB-68 | SPARC | Predicted | 1.000 | RB-16 | HSPA1B | Predicted | 0.004 |
| RAB-68 | SULT2A1 | Predicted | 0.000 | RB-16 | HSPA8 | Predicted | 0.004 |
| SL-01 | ABCB1 | Predicted | 0.000 | RB-16 | KRAS | Predicted | 0.025 |
| SL-01 | ACSL1 | Predicted | 0.000 | RB-16 | MAPK1 | Predicted | 0.077 |
| SL-01 | ADH1C | Predicted | 0.001 | RB-16 | MAPK3 | Predicted | 0.029 |
| SL-01 | AKT2 | Predicted | 0.374 | RB-16 | MAT1A | Predicted | 0.004 |
| SL-01 | ASS1 | Predicted | 0.000 | RB-16 | MME | Predicted | 0.126 |
| SL-01 | AURKA | Predicted | 0.003 | RB-16 | MMP12 | Predicted | 0.032 |
| SL-01 | CA1 | Predicted | 0.046 | RB-16 | NME1 | Predicted | 0.017 |
| SL-01 | CA2 | Predicted | 0.046 | RB-16 | NME2 | Predicted | 0.003 |
| SL-01 | CCNA2 | Predicted | 0.907 | RB-16 | PGK1 | Predicted | 0.004 |
| SL-01 | CCT3 | Predicted | 0.003 | RB-16 | PTK2 | Predicted | 0.004 |
| SL-01 | CES1 | Predicted | 0.024 | RB-16 | RHOA | Predicted | 0.003 |
| SL-01 | CTSB | Predicted | 0.178 | RB-16 | RND3 | Predicted | 0.004 |
| SL-01 | ESR1 | Predicted | 0.441 | RB-16 | SERPINE1 | Predicted | 0.024 |
| SL-01 | FGF1 | Predicted | 0.077 | RB-16 | SGK1 | Predicted | 0.002 |
| SL-01 | FGF2 | Predicted | 0.077 | RB-16 | SRC | Predicted | 0.163 |
| SL-01 | GABRE | Predicted | 0.013 | RB-16 | TGM2 | Predicted | 0.003 |
| SL-01 | GAPDH | Predicted | 0.013 | RB-16 | TOP2A | Predicted | 0.047 |
| SL-01 | GLUD1 | Predicted | 0.030 | RB-16 | TRDMT1 | Predicted | 0.005 |
| SL-01 | GNMT | Predicted | 0.002 | RB-16 | VEGFA | Predicted | 0.020 |
| SL-01 | HSP90AA1 | Predicted | 0.003 | RB-17 | ACSL1 | Predicted | 0.022 |
| SL-01 | HSP90B1 | Predicted | 0.003 | RB-17 | AGXT | Predicted | 0.006 |

| SL-01 | HSPA1B | Predicted | 0.003 | RB-17 | ALDOA | Predicted | 0.107 |
| --- | --- | --- | --- | --- | --- | --- | --- |
| SL-01 | HSPA8 | Predicted | 0.003 | RB-17 | AURKA | Predicted | 0.005 |
| SL-01 | KRAS | Predicted | 0.114 | RB-17 | CA1 | Predicted | 0.170 |
| SL-01 | MAPK1 | Predicted | 0.005 | RB-17 | CA2 | Predicted | 0.135 |
| SL-01 | MAT1A | Predicted | 0.003 | RB-17 | CCNA2 | Predicted | 1.000 |
| SL-01 | MME | Predicted | 0.109 | RB-17 | CCT3 | Predicted | 0.005 |
| SL-01 | MMP12 | Predicted | 0.172 | RB-17 | CES1 | Predicted | 0.032 |
| SL-01 | NME1 | Predicted | 0.021 | RB-17 | CTSB | Predicted | 0.110 |
| SL-01 | NOS2A | Predicted | 0.056 | RB-17 | ESR1 | Predicted | 0.106 |
| SL-01 | PGK1 | Predicted | 0.003 | RB-17 | GLUD1 | Predicted | 0.005 |
| SL-01 | PTGS2 | Predicted | 0.089 | RB-17 | GNAI1 | Predicted | 0.005 |
| SL-01 | PTK2 | Predicted | 0.003 | RB-17 | GSTP1 | Predicted | 0.004 |
| SL-01 | RND3 | Predicted | 0.011 | RB-17 | HSP90AA1 | Predicted | 0.005 |
| SL-01 | SRC | Predicted | 0.229 | RB-17 | HSP90B1 | Predicted | 0.005 |
| SL-01 | SULT2A1 | Predicted | 0.010 | RB-17 | HSPA1B | Predicted | 0.005 |
| SL-01 | TOP2A | Predicted | 0.028 | RB-17 | HSPA8 | Predicted | 0.005 |
| SL-01 | TRDMT1 | Predicted | 0.002 | RB-17 | KRAS | Predicted | 0.032 |
| SL-02 | ABCB1 | Predicted | 0.018 | RB-17 | MAPK3 | Predicted | 0.062 |
| SL-02 | ACSL1 | Predicted | 0.052 | RB-17 | MAT1A | Predicted | 0.005 |
| SL-02 | ADH1C | Predicted | 0.010 | RB-17 | MMP14 | Predicted | 0.000 |
| SL-02 | AGXT | Predicted | 0.011 | RB-17 | MMP2 | Predicted | 0.000 |
| SL-02 | AKT2 | Predicted | 0.125 | RB-17 | MMP3 | Predicted | 0.000 |
| SL-02 | ASS1 | Predicted | 0.018 | RB-17 | MMP7 | Predicted | 0.000 |
| SL-02 | AURKA | Predicted | 0.008 | RB-17 | MMP9 | Predicted | 0.000 |
| SL-02 | CA1 | Predicted | 0.052 | RB-17 | MTAP | Predicted | 0.225 |

| SL-02 | CA2 | Predicted | 0.052 | RB-17 | NME1 | Predicted | 0.025 |
| --- | --- | --- | --- | --- | --- | --- | --- |
| SL-02 | CASP1 | Predicted | 0.038 | RB-17 | NME2 | Predicted | 0.005 |
| SL-02 | CCNA2 | Predicted | 1.000 | RB-17 | NOS2A | Predicted | 0.223 |
| SL-02 | CCT3 | Predicted | 0.023 | RB-17 | PGK1 | Predicted | 0.005 |
| SL-02 | CES1 | Predicted | 0.031 | RB-17 | PTGS2 | Predicted | 0.337 |
| SL-02 | CTSB | Predicted | 0.173 | RB-17 | PTK2 | Predicted | 0.005 |
| SL-02 | ESR1 | Predicted | 0.426 | RB-17 | RHOA | Predicted | 0.005 |
| SL-02 | FGF1 | Predicted | 0.080 | RB-17 | SRC | Predicted | 0.105 |
| SL-02 | FGF2 | Predicted | 0.080 | RB-17 | SULT2A1 | Predicted | 0.010 |
| SL-02 | GABRE | Predicted | 0.026 | RB-17 | TGM2 | Predicted | 0.005 |
| SL-02 | GAPDH | Predicted | 0.016 | RB-17 | TOP2A | Predicted | 0.057 |
| SL-02 | GLUD1 | Predicted | 0.027 | RB-18 | ACSL1 | Predicted | 0.019 |
| SL-02 | GNAI1 | Predicted | 0.010 | RB-18 | AGXT | Predicted | 0.004 |
| SL-02 | GNMT | Predicted | 0.006 | RB-18 | AKR1B10 | Predicted | 0.077 |
| SL-02 | GSTP1 | Predicted | 0.015 | RB-18 | AKT2 | Predicted | 0.055 |
| SL-02 | HAO1 | Predicted | 0.000 | RB-18 | AURKA | Predicted | 0.003 |
| SL-02 | HSP90AA1 | Predicted | 0.008 | RB-18 | CA1 | Predicted | 0.055 |
| SL-02 | HSP90B1 | Predicted | 0.008 | RB-18 | CA2 | Predicted | 0.055 |
| SL-02 | HSPA1B | Predicted | 0.008 | RB-18 | CASP1 | Predicted | 0.005 |
| SL-02 | HSPA8 | Predicted | 0.008 | RB-18 | CCNA2 | Predicted | 1.000 |
| SL-02 | KRAS | Predicted | 0.038 | RB-18 | CCT3 | Predicted | 0.003 |
| SL-02 | MAPK1 | Predicted | 0.012 | RB-18 | CTSB | Predicted | 0.073 |
| SL-02 | MAPK3 | Predicted | 0.044 | RB-18 | ESR1 | Predicted | 0.074 |
| SL-02 | MAT1A | Predicted | 0.008 | RB-18 | FGF1 | Predicted | 0.034 |
| SL-02 | MME | Predicted | 0.109 | RB-18 | FGF2 | Predicted | 0.034 |

| SL-02 | MMP12 | Predicted | 0.161 | RB-18 | GABRE | Predicted | 0.001 |
| --- | --- | --- | --- | --- | --- | --- | --- |
| SL-02 | MMP9 | Predicted | 0.038 | RB-18 | GLUD1 | Predicted | 0.011 |
| SL-02 | MTAP | Predicted | 0.162 | RB-18 | GNAI1 | Predicted | 0.004 |
| SL-02 | NME1 | Predicted | 0.039 | RB-18 | GNMT | Predicted | 0.002 |
| SL-02 | NME2 | Predicted | 0.010 | RB-18 | GSTP1 | Predicted | 0.000 |
| SL-02 | NOS2A | Predicted | 0.050 | RB-18 | HAO1 | Predicted | 0.000 |
| SL-02 | PGK1 | Predicted | 0.008 | RB-18 | HSP90AA1 | Predicted | 0.003 |
| SL-02 | PTGS2 | Predicted | 0.084 | RB-18 | HSP90B1 | Predicted | 0.003 |
| SL-02 | PTK2 | Predicted | 0.008 | RB-18 | HSPA1B | Predicted | 0.003 |
| SL-02 | RHOA | Predicted | 0.010 | RB-18 | HSPA8 | Predicted | 0.003 |
| SL-02 | RND3 | Predicted | 0.013 | RB-18 | KRAS | Predicted | 0.018 |
| SL-02 | SGK1 | Predicted | 0.000 | RB-18 | LYZ | Predicted | 0.071 |
| SL-02 | SRC | Predicted | 0.077 | RB-18 | MAPK1 | Predicted | 0.098 |
| SL-02 | SULT2A1 | Predicted | 0.017 | RB-18 | MAPK3 | Predicted | 0.015 |
| SL-02 | TGM2 | Predicted | 0.010 | RB-18 | MAT1A | Predicted | 0.003 |
| SL-02 | TOP2A | Predicted | 0.031 | RB-18 | MME | Predicted | 0.047 |
| SL-02 | TRDMT1 | Predicted | 0.006 | RB-18 | MMP9 | Predicted | 0.005 |
| SL-02 | VEGFA | Predicted | 0.038 | RB-18 | MTAP | Predicted | 0.072 |
| SL-03 | ABCB1 | Predicted | 0.005 | RB-18 | NME1 | Predicted | 0.017 |
| SL-03 | ACSL1 | Predicted | 0.005 | RB-18 | NME2 | Predicted | 0.004 |
| SL-03 | AKT2 | Predicted | 0.069 | RB-18 | NOS2A | Predicted | 0.074 |
| SL-03 | ALDH1A1 | Predicted | 0.014 | RB-18 | PGK1 | Predicted | 0.003 |
| SL-03 | ALDH1L1 | Predicted | 0.002 | RB-18 | PTGS2 | Predicted | 0.051 |
| SL-03 | ASS1 | Predicted | 0.005 | RB-18 | PTK2 | Predicted | 0.003 |
| SL-03 | CA1 | Predicted | 0.012 | RB-18 | RHOA | Predicted | 0.004 |

| SL-03 | CA2 | Predicted | 0.012 | RB-18 | RND3 | Predicted | 0.004 |
| --- | --- | --- | --- | --- | --- | --- | --- |
| SL-03 | CCNA2 | Predicted | 1.000 | RB-18 | SERPINE1 | Predicted | 0.015 |
| SL-03 | CCT3 | Predicted | 0.005 | RB-18 | SGK1 | Predicted | 0.000 |
| SL-03 | CDK4 | Predicted | 0.024 | RB-18 | SRC | Predicted | 0.068 |
| SL-03 | CYP2C8 | Predicted | 0.007 | RB-18 | TGM2 | Predicted | 0.004 |
| SL-03 | EGFR | Predicted | 0.072 | RB-18 | TOP2A | Predicted | 0.039 |
| SL-03 | ESR1 | Predicted | 0.074 | RB-18 | TRDMT1 | Predicted | 0.002 |
| SL-03 | FTCD | Predicted | 0.002 | RB-18 | VEGFA | Predicted | 0.005 |
| SL-03 | GLUD1 | Predicted | 0.008 | RB-19 | ABCB1 | Predicted | 0.004 |
| SL-03 | GNMT | Predicted | 0.001 | RB-19 | ACSL1 | Predicted | 0.004 |
| SL-03 | GSTP1 | Predicted | 0.023 | RB-19 | AKT2 | Predicted | 0.166 |
| SL-03 | KRAS | Predicted | 0.045 | RB-19 | ASS1 | Predicted | 0.004 |
| SL-03 | LYZ | Predicted | 0.072 | RB-19 | CA1 | Predicted | 0.029 |
| SL-03 | MAPK1 | Predicted | 0.067 | RB-19 | CA2 | Predicted | 0.029 |
| SL-03 | MAPK3 | Predicted | 0.024 | RB-19 | CCNA2 | Predicted | 1.000 |
| SL-03 | MME | Predicted | 0.115 | RB-19 | CCT3 | Predicted | 0.008 |
| SL-03 | MTHFD1 | Predicted | 0.002 | RB-19 | CDK4 | Predicted | 0.031 |
| SL-03 | MTR | Predicted | 0.002 | RB-19 | CES1 | Predicted | 0.029 |
| SL-03 | NME1 | Predicted | 0.074 | RB-19 | CTSB | Predicted | 0.098 |
| SL-03 | NOS2A | Predicted | 0.073 | RB-19 | ESR1 | Predicted | 0.195 |
| SL-03 | PLAU | Predicted | 0.062 | RB-19 | GABRE | Predicted | 0.001 |
| SL-03 | PTGS2 | Predicted | 0.040 | RB-19 | GAPDH | Predicted | 0.011 |
| SL-03 | RARA | Predicted | 0.007 | RB-19 | GLUD1 | Predicted | 0.015 |
| SL-03 | RBP1 | Predicted | 0.005 | RB-19 | GSTP1 | Predicted | 0.029 |
| SL-03 | RND3 | Predicted | 0.008 | RB-19 | HAO1 | Predicted | 0.000 |

| SL-03 | SERPINE1 | Predicted | 0.019 | RB-19 | KRAS | Predicted | 0.068 |
| --- | --- | --- | --- | --- | --- | --- | --- |
| SL-03 | SRC | Predicted | 0.073 | RB-19 | MAPK1 | Predicted | 0.089 |
| SL-03 | TRDMT1 | Predicted | 0.001 | RB-19 | MAPK3 | Predicted | 0.031 |
| SL-03 | VEGFA | Predicted | 0.019 | RB-19 | MME | Predicted | 0.148 |
| SL-04 | ABCB1 | Predicted | 0.014 | RB-19 | MMP12 | Predicted | 0.094 |
| SL-04 | ACSL1 | Predicted | 0.014 | RB-19 | NME1 | Predicted | 0.095 |
| SL-04 | ADH1B | Predicted | 0.009 | RB-19 | NOS2A | Predicted | 0.023 |
| SL-04 | ADH1C | Predicted | 0.019 | RB-19 | PTGS2 | Predicted | 0.028 |
| SL-04 | ADH4 | Predicted | 0.009 | RB-19 | RND3 | Predicted | 0.015 |
| SL-04 | AKR1C2 | Predicted | 0.001 | RB-19 | SGK1 | Predicted | 0.000 |
| SL-04 | AKT2 | Predicted | 0.105 | RB-19 | SRC | Predicted | 0.046 |
| SL-04 | ALDH1A1 | Predicted | 0.009 | RB-19 | SULT2A1 | Predicted | 0.016 |
| SL-04 | ALDH1B1 | Predicted | 0.001 | RB-19 | TOP2A | Predicted | 0.019 |
| SL-04 | ALDH1L1 | Predicted | 0.000 | RB-20 | ABCB1 | Predicted | 0.016 |
| SL-04 | ALDH2 | Predicted | 0.009 | RB-20 | ACSL1 | Predicted | 0.016 |
| SL-04 | ALDH3A2 | Predicted | 0.001 | RB-20 | ADH1B | Predicted | 0.008 |
| SL-04 | ALDH4A1 | Predicted | 0.001 | RB-20 | AKR1B10 | Predicted | 0.008 |
| SL-04 | ASS1 | Predicted | 0.014 | RB-20 | AKR1C2 | Predicted | 0.008 |
| SL-04 | AURKA | Predicted | 0.006 | RB-20 | AKT2 | Predicted | 0.122 |
| SL-04 | CASP1 | Predicted | 0.034 | RB-20 | ALDH1L1 | Predicted | 0.007 |
| SL-04 | CCNA2 | Predicted | 0.283 | RB-20 | ASS1 | Predicted | 0.016 |
| SL-04 | CCT3 | Predicted | 0.021 | RB-20 | CCNA2 | Predicted | 0.830 |
| SL-04 | CDK4 | Predicted | 0.043 | RB-20 | CCT3 | Predicted | 0.008 |
| SL-04 | CES1 | Predicted | 0.003 | RB-20 | DCXR | Predicted | 0.008 |
| SL-04 | ESR1 | Predicted | 0.136 | RB-20 | EGFR | Predicted | 0.141 |

| SL-04 | FTCD | Predicted | 0.000 | RB-20 | ESR1 | Predicted | 0.260 |
| --- | --- | --- | --- | --- | --- | --- | --- |
| SL-04 | GAPDH | Predicted | 0.029 | RB-20 | FTCD | Predicted | 0.007 |
| SL-04 | GLUD1 | Predicted | 0.034 | RB-20 | GAPDH | Predicted | 0.020 |
| SL-04 | GNAI1 | Predicted | 0.004 | RB-20 | GLUD1 | Predicted | 0.009 |
| SL-04 | GNMT | Predicted | 0.004 | RB-20 | GSTP1 | Predicted | 0.091 |
| SL-04 | GSTP1 | Predicted | 0.115 | RB-20 | KRAS | Predicted | 0.066 |
| SL-04 | HAO1 | Predicted | 0.003 | RB-20 | MAPK1 | Predicted | 0.012 |
| SL-04 | HSD17B10 | Predicted | 0.009 | RB-20 | MME | Predicted | 0.331 |
| SL-04 | HSD17B4 | Predicted | 0.009 | RB-20 | MTHFD1 | Predicted | 0.021 |
| SL-04 | HSP90AA1 | Predicted | 0.006 | RB-20 | MTR | Predicted | 0.007 |
| SL-04 | HSP90B1 | Predicted | 0.006 | RB-20 | NME1 | Predicted | 0.131 |
| SL-04 | HSPA1B | Predicted | 0.006 | RB-20 | NOS2A | Predicted | 0.133 |
| SL-04 | HSPA8 | Predicted | 0.006 | RB-20 | RAF1 | Predicted | 0.016 |
| SL-04 | KRAS | Predicted | 0.083 | RB-20 | RARA | Predicted | 0.018 |
| SL-04 | MAPK1 | Predicted | 0.120 | RB-20 | RND3 | Predicted | 0.009 |
| SL-04 | MAPK3 | Predicted | 0.043 | RB-20 | SERPINE1 | Predicted | 0.042 |
| SL-04 | MAT1A | Predicted | 0.006 | RB-20 | TUBB | Predicted | 0.008 |
| SL-04 | MET | Predicted | 0.134 | RB-20 | VEGFA | Predicted | 0.022 |
| SL-04 | MME | Predicted | 0.209 | RB-21 | ABCB1 | Predicted | 0.005 |
| SL-04 | MMP12 | Predicted | 0.054 | RB-21 | ACSL1 | Predicted | 0.005 |
| SL-04 | MMP9 | Predicted | 0.034 | RB-21 | AGXT | Predicted | 0.004 |
| SL-04 | MTHFD1 | Predicted | 0.008 | RB-21 | AKT2 | Predicted | 0.043 |
| SL-04 | MTR | Predicted | 0.000 | RB-21 | ALDH1A1 | Predicted | 0.020 |
| SL-04 | NME1 | Predicted | 0.028 | RB-21 | ASS1 | Predicted | 0.005 |
| SL-04 | NME2 | Predicted | 0.004 | RB-21 | AURKA | Predicted | 0.003 |

| SL-04 | PGK1 | Predicted | 0.006 | RB-21 | CA1 | Predicted | 0.012 |
| --- | --- | --- | --- | --- | --- | --- | --- |
| SL-04 | PTK2 | Predicted | 0.006 | RB-21 | CA2 | Predicted | 0.012 |
| SL-04 | QDPR | Predicted | 0.009 | RB-21 | CCNA2 | Predicted | 1.000 |
| SL-04 | RHOA | Predicted | 0.004 | RB-21 | CCT3 | Predicted | 0.010 |
| SL-04 | RND3 | Predicted | 0.005 | RB-21 | CYP2C8 | Predicted | 0.006 |
| SL-04 | SERPINE1 | Predicted | 0.009 | RB-21 | ESR1 | Predicted | 0.074 |
| SL-04 | SGK1 | Predicted | 0.003 | RB-21 | GLUD1 | Predicted | 0.012 |
| SL-04 | SRC | Predicted | 0.065 | RB-21 | GNAI1 | Predicted | 0.003 |
| SL-04 | TGM2 | Predicted | 0.004 | RB-21 | GNMT | Predicted | 0.002 |
| SL-04 | TOP2A | Predicted | 0.201 | RB-21 | GSTP1 | Predicted | 0.023 |
| SL-04 | TRDMT1 | Predicted | 0.004 | RB-21 | HSP90AA1 | Predicted | 0.003 |
| SL-04 | TUBB | Predicted | 0.006 | RB-21 | HSP90B1 | Predicted | 0.003 |
| SL-04 | VEGFA | Predicted | 0.065 | RB-21 | HSPA1B | Predicted | 0.003 |
| SL-05 | ABCB1 | Predicted | 0.010 | RB-21 | HSPA8 | Predicted | 0.003 |
| SL-05 | ACSL1 | Predicted | 0.010 | RB-21 | KRAS | Predicted | 0.046 |
| SL-05 | ADH1B | Predicted | 0.004 | RB-21 | LYZ | Predicted | 0.075 |
| SL-05 | ADH1C | Predicted | 0.004 | RB-21 | MAPK1 | Predicted | 0.031 |
| SL-05 | ADH4 | Predicted | 0.004 | RB-21 | MAT1A | Predicted | 0.003 |
| SL-05 | AKT2 | Predicted | 0.131 | RB-21 | MME | Predicted | 0.112 |
| SL-05 | ALDH1A1 | Predicted | 0.004 | RB-21 | NME1 | Predicted | 0.017 |
| SL-05 | ALDH1L1 | Predicted | 0.011 | RB-21 | NME2 | Predicted | 0.003 |
| SL-05 | ALDH2 | Predicted | 0.004 | RB-21 | PGK1 | Predicted | 0.003 |
| SL-05 | ASS1 | Predicted | 0.010 | RB-21 | PLAU | Predicted | 0.050 |
| SL-05 | CCNA2 | Predicted | 0.712 | RB-21 | PTGS2 | Predicted | 0.043 |
| SL-05 | CCT3 | Predicted | 0.010 | RB-21 | PTK2 | Predicted | 0.003 |

| SL-05 | CDK4 | Predicted | 0.044 | RB-21 | RARA | Predicted | 0.030 |
| --- | --- | --- | --- | --- | --- | --- | --- |
| SL-05 | EGFR | Predicted | 0.134 | RB-21 | RBP1 | Predicted | 0.006 |
| SL-05 | ESR1 | Predicted | 0.138 | RB-21 | RHOA | Predicted | 0.003 |
| SL-05 | FTCD | Predicted | 0.011 | RB-21 | RND3 | Predicted | 0.005 |
| SL-05 | GAPDH | Predicted | 0.019 | RB-21 | SERPINE1 | Predicted | 0.022 |
| SL-05 | GLUD1 | Predicted | 0.020 | RB-21 | TGM2 | Predicted | 0.003 |
| SL-05 | GNMT | Predicted | 0.002 | RB-21 | TRDMT1 | Predicted | 0.002 |
| SL-05 | GSTP1 | Predicted | 0.118 | RB-21 | VEGFA | Predicted | 0.022 |
| SL-05 | HSD17B10 | Predicted | 0.004 | RB-22 | ALDH2 | Predicted | 0.322 |
| SL-05 | HSD17B4 | Predicted | 0.004 | RB-22 | ALDOA | Predicted | 0.315 |
| SL-05 | KRAS | Predicted | 0.066 | RB-22 | CES1 | Predicted | 0.025 |
| SL-05 | MAPK1 | Predicted | 0.123 | RB-22 | ESR1 | Predicted | 1.000 |
| SL-05 | MAPK3 | Predicted | 0.044 | RB-22 | LYZ | Predicted | 0.006 |
| SL-05 | MME | Predicted | 0.340 | RB-22 | MMP14 | Predicted | 0.000 |
| SL-05 | MMP12 | Predicted | 0.055 | RB-22 | MMP2 | Predicted | 0.000 |
| SL-05 | MTHFD1 | Predicted | 0.011 | RB-22 | MMP3 | Predicted | 0.000 |
| SL-05 | MTR | Predicted | 0.011 | RB-22 | MMP7 | Predicted | 0.000 |
| SL-05 | NME1 | Predicted | 0.137 | RB-22 | MMP9 | Predicted | 0.000 |
| SL-05 | NOS2A | Predicted | 0.136 | RB-22 | REG1A | Predicted | 0.025 |
| SL-05 | QDPR | Predicted | 0.004 | RB-23 | ALDH1L1 | Predicted | 0.006 |
| SL-05 | RND3 | Predicted | 0.010 | RB-23 | DDR1 | Predicted | 0.060 |
| SL-05 | SRC | Predicted | 0.136 | RB-23 | EGFR | Predicted | 0.317 |
| SL-05 | TRDMT1 | Predicted | 0.002 | RB-23 | ESR1 | Predicted | 0.315 |
| SL-05 | VEGFA | Predicted | 0.029 | RB-23 | FTCD | Predicted | 0.006 |
| SL-06 | CASP3 | Validated | 1.000 | RB-23 | GSTP1 | Predicted | 0.052 |

| SL-06 | ABCB1 | P08183 | 0.014 | RB-23 | MME | Predicted | 0.330 |
| --- | --- | --- | --- | --- | --- | --- | --- |
| SL-06 | ACSL1 | P33121 | 0.014 | RB-23 | MTHFD1 | Predicted | 0.006 |
| SL-06 | ADH1B | P00325 | 0.009 | RB-23 | MTR | Predicted | 0.006 |
| SL-06 | ADH1C | P00326 | 0.019 | RB-23 | PDGFRA | Predicted | 0.060 |
| SL-06 | ADH4 | P08319 | 0.009 | RB-24 | ABCB1 | Predicted | 0.012 |
| SL-06 | AKR1C2 | P52895 | 0.001 | RB-24 | ACSL1 | Predicted | 0.012 |
| SL-06 | AKT2 | P31751 | 0.105 | RB-24 | ADH1C | Predicted | 0.014 |
| SL-06 | ALDH1A1 | P00352 | 0.009 | RB-24 | AGXT | Predicted | 0.013 |
| SL-06 | ALDH1B1 | P30837 | 0.001 | RB-24 | AKR1B10 | Predicted | 0.163 |
| SL-06 | ALDH1L1 | O75891 | 0.000 | RB-24 | AKT2 | Predicted | 0.224 |
| SL-06 | ALDH2 | P05091 | 0.009 | RB-24 | ASS1 | Predicted | 0.012 |
| SL-06 | ALDH3A2 | P51648 | 0.001 | RB-24 | AURKA | Predicted | 0.010 |
| SL-06 | ALDH4A1 | P30038 | 0.001 | RB-24 | CA1 | Predicted | 0.080 |
| SL-06 | ASS1 | P00966 | 0.014 | RB-24 | CA2 | Predicted | 0.080 |
| SL-06 | AURKA | O14965 | 0.006 | RB-24 | CASP1 | Predicted | 0.036 |
| SL-06 | CASP1 | P29466 | 0.034 | RB-24 | CCNA2 | Predicted | 1.000 |
| SL-06 | CCNA2 | P20248 | 0.283 | RB-24 | CCT3 | Predicted | 0.026 |
| SL-06 | CCT3 | P49368 | 0.021 | RB-24 | CDK4 | Predicted | 0.059 |
| SL-06 | CDK4 | P11802 | 0.043 | RB-24 | CES1 | Predicted | 0.014 |
| SL-06 | CES1 | P23141 | 0.003 | RB-24 | CTSB | Predicted | 0.165 |
| SL-06 | ESR1 | P03372 | 0.136 | RB-24 | ESR1 | Predicted | 0.175 |
| SL-06 | FTCD | O95954 | 0.000 | RB-24 | GABRE | Predicted | 0.004 |
| SL-06 | GAPDH | P04406 | 0.029 | RB-24 | GLUD1 | Predicted | 0.031 |
| SL-06 | GLUD1 | P00367 | 0.034 | RB-24 | GNAI1 | Predicted | 0.012 |
| SL-06 | GNAI1 | P63096 | 0.004 | RB-24 | GNMT | Predicted | 0.007 |

| SL-06 | GNMT | Q14749 | 0.004 | RB-24 | GSTP1 | Predicted | 0.052 |
| --- | --- | --- | --- | --- | --- | --- | --- |
| SL-06 | GSTP1 | P09211 | 0.115 | RB-24 | HAO1 | Predicted | 0.000 |
| SL-06 | HAO1 | Q9UJM8 | 0.003 | RB-24 | HSP90AA1 | Predicted | 0.010 |
| SL-06 | HSD17B10 | Q99714 | 0.009 | RB-24 | HSP90B1 | Predicted | 0.010 |
| SL-06 | HSD17B4 | P51659 | 0.009 | RB-24 | HSPA1B | Predicted | 0.010 |
| SL-06 | HSP90AA1 | P07900 | 0.006 | RB-24 | HSPA8 | Predicted | 0.010 |
| SL-06 | HSP90B1 | P14625 | 0.006 | RB-24 | KRAS | Predicted | 0.052 |
| SL-06 | HSPA1B | P08107 | 0.006 | RB-24 | MAPK1 | Predicted | 0.059 |
| SL-06 | HSPA8 | P11142 | 0.006 | RB-24 | MAPK3 | Predicted | 0.118 |
| SL-06 | KRAS | P01116 | 0.083 | RB-24 | MAT1A | Predicted | 0.010 |
| SL-06 | MAPK1 | P28482 | 0.120 | RB-24 | MME | Predicted | 0.112 |
| SL-06 | MAPK3 | P27361 | 0.043 | RB-24 | MMP12 | Predicted | 0.053 |
| SL-06 | MAT1A | Q00266 | 0.006 | RB-24 | MMP9 | Predicted | 0.036 |
| SL-06 | MET | P08581 | 0.134 | RB-24 | NME1 | Predicted | 0.043 |
| SL-06 | MME | P08473 | 0.209 | RB-24 | NME2 | Predicted | 0.012 |
| SL-06 | MMP12 | P39900 | 0.054 | RB-24 | PGK1 | Predicted | 0.010 |
| SL-06 | MMP9 | P14780 | 0.034 | RB-24 | PTGS2 | Predicted | 0.054 |
| SL-06 | MTHFD1 | P11586 | 0.008 | RB-24 | PTK2 | Predicted | 0.010 |
| SL-06 | MTR | Q99707 | 0.000 | RB-24 | RHOA | Predicted | 0.012 |
| SL-06 | NME1 | P15531 | 0.028 | RB-24 | RND3 | Predicted | 0.015 |
| SL-06 | NME2 | P22392 | 0.004 | RB-24 | SERPINE1 | Predicted | 0.029 |
| SL-06 | PGK1 | P00558 | 0.006 | RB-24 | SGK1 | Predicted | 0.000 |
| SL-06 | PTK2 | Q05397 | 0.006 | RB-24 | SRC | Predicted | 0.162 |
| SL-06 | QDPR | P09417 | 0.009 | RB-24 | TGM2 | Predicted | 0.012 |
| SL-06 | RHOA | P61586 | 0.004 | RB-24 | TOP2A | Predicted | 0.089 |

| SL-06 | RND3 | P61587 | 0.005 | RB-24 | TRDMT1 | Predicted | 0.007 |
| --- | --- | --- | --- | --- | --- | --- | --- |
| SL-06 | SERPINE1 | P05121 | 0.009 | RB-24 | VEGFA | Predicted | 0.081 |
| SL-06 | SGK1 | O00141 | 0.003 | RB-25 | ALDH2 | Predicted | 0.094 |
| SL-06 | SRC | P12931 | 0.065 | RB-25 | ALDOA | Predicted | 0.095 |
| SL-06 | TGM2 | P21980 | 0.004 | RB-25 | ARG1 | Predicted | 0.042 |
| SL-06 | TOP2A | P11388 | 0.201 | RB-25 | B2M | Predicted | 0.019 |
| SL-06 | TRDMT1 | O14717 | 0.004 | RB-25 | CA1 | Predicted | 0.242 |
| SL-06 | TUBB | P07437 | 0.006 | RB-25 | CA2 | Predicted | 0.172 |
| SL-06 | VEGFA | P15692 | 0.065 | RB-25 | CCNA2 | Predicted | 0.091 |
| SL-07 | ABCB1 | Predicted | 0.010 | RB-25 | CES1 | Predicted | 0.014 |
| SL-07 | ACSL1 | Predicted | 0.043 | RB-25 | DNMT1 | Predicted | 0.043 |
| SL-07 | ADH1C | Predicted | 0.004 | RB-25 | ESR1 | Predicted | 0.044 |
| SL-07 | AGXT | Predicted | 0.005 | RB-25 | FGF1 | Predicted | 0.015 |
| SL-07 | AKT2 | Predicted | 0.140 | RB-25 | FGF2 | Predicted | 0.015 |
| SL-07 | ASS1 | Predicted | 0.010 | RB-25 | GABRE | Predicted | 0.054 |
| SL-07 | AURKA | Predicted | 0.004 | RB-25 | GSTP1 | Predicted | 0.000 |
| SL-07 | CA1 | Predicted | 0.095 | RB-25 | HGF | Predicted | 0.015 |
| SL-07 | CA2 | Predicted | 0.095 | RB-25 | KRAS | Predicted | 0.008 |
| SL-07 | CCNA2 | Predicted | 0.633 | RB-25 | LCN2 | Predicted | 0.091 |
| SL-07 | CCT3 | Predicted | 0.019 | RB-25 | MAPK1 | Predicted | 0.032 |
| SL-07 | CES1 | Predicted | 0.024 | RB-25 | MMP12 | Predicted | 0.027 |
| SL-07 | CTSB | Predicted | 0.161 | RB-25 | MMP3 | Predicted | 0.027 |
| SL-07 | ESR1 | Predicted | 0.281 | RB-25 | MTAP | Predicted | 0.090 |
| SL-07 | FGF1 | Predicted | 0.072 | RB-25 | NOS2A | Predicted | 0.179 |
| SL-07 | FGF2 | Predicted | 0.072 | RB-25 | PARP1 | Predicted | 0.094 |

| SL-07 | GABRE | Predicted | 0.014 | RB-25 | PC | Predicted | 0.035 |
| --- | --- | --- | --- | --- | --- | --- | --- |
| SL-07 | GLUD1 | Predicted | 0.004 | RB-25 | PTGS2 | Predicted | 0.432 |
| SL-07 | GNAI1 | Predicted | 0.000 | RB-25 | REG1A | Predicted | 0.014 |
| SL-07 | GNMT | Predicted | 0.001 | RB-25 | TOP2A | Predicted | 0.093 |
| SL-07 | GSTP1 | Predicted | 0.007 | RB-26 | BAD | Validated | 1.000 |
| SL-07 | HSP90AA1 | Predicted | 0.004 | RB-26 | CASP3 | Validated | 1.000 |
| SL-07 | HSP90B1 | Predicted | 0.004 | RB-26 | CDKN1B | Validated | 1.000 |
| SL-07 | HSPA1B | Predicted | 0.004 | RB-26 | FAS | Validated | 1.000 |
| SL-07 | HSPA8 | Predicted | 0.004 | RB-26 | FOS | Validated | 1.000 |
| SL-07 | KRAS | Predicted | 0.043 | RB-26 | GSTP1 | Validated | 1.000 |
| SL-07 | MAPK1 | Predicted | 0.006 | RB-26 | LEPR | Validated | 1.000 |
| SL-07 | MAPK3 | Predicted | 0.043 | RB-26 | MMP9 | Validated | 1.000 |
| SL-07 | MAT1A | Predicted | 0.004 | RB-26 | NFKBIA | Validated | 1.000 |
| SL-07 | MME | Predicted | 0.098 | RB-26 | NOS2A | Validated | 1.000 |
| SL-07 | MMP12 | Predicted | 0.156 | RB-26 | SRC | Validated | 1.000 |
| SL-07 | MTAP | Predicted | 0.310 | RB-26 | VEGFA | Validated | 1.000 |
| SL-07 | NME1 | Predicted | 0.015 | RB-26 | ABCB1 | Predicted | 0.004 |
| SL-07 | NME2 | Predicted | 0.000 | RB-26 | ACSL1 | Predicted | 0.004 |
| SL-07 | NOS2A | Predicted | 0.050 | RB-26 | AGXT | Predicted | 0.003 |
| SL-07 | PGK1 | Predicted | 0.004 | RB-26 | AKR1B10 | Predicted | 0.084 |
| SL-07 | PTGS2 | Predicted | 0.132 | RB-26 | AKT2 | Predicted | 0.130 |
| SL-07 | PTK2 | Predicted | 0.004 | RB-26 | ALDH1A1 | Predicted | 0.022 |
| SL-07 | RHOA | Predicted | 0.000 | RB-26 | ASS1 | Predicted | 0.004 |
| SL-07 | SRC | Predicted | 0.148 | RB-26 | CA1 | Predicted | 0.012 |
| SL-07 | SULT2A1 | Predicted | 0.011 | RB-26 | CA2 | Predicted | 0.012 |

| SL-07 | TGM2 | Predicted | 0.000 | RB-26 | CCNA2 | Predicted | 1.000 |
| --- | --- | --- | --- | --- | --- | --- | --- |
| SL-07 | TOP2A | Predicted | 0.028 | RB-26 | CCT3 | Predicted | 0.004 |
| SL-07 | TRDMT1 | Predicted | 0.001 | RB-26 | CDK4 | Predicted | 0.029 |
| SL-08 | ABCB1 | Predicted | 0.013 | RB-26 | CTSB | Predicted | 0.086 |
| SL-08 | ACSL1 | Predicted | 0.013 | RB-26 | CYP2C8 | Predicted | 0.008 |
| SL-08 | ADH1B | Predicted | 0.003 | RB-26 | ESR1 | Predicted | 0.176 |
| SL-08 | ADH1C | Predicted | 0.015 | RB-26 | GLUD1 | Predicted | 0.007 |
| SL-08 | ADH4 | Predicted | 0.003 | RB-26 | GNAI1 | Predicted | 0.004 |
| SL-08 | AKT2 | Predicted | 0.111 | RB-26 | GNMT | Predicted | 0.003 |
| SL-08 | ALDH1A1 | Predicted | 0.003 | RB-26 | HAO1 | Predicted | 0.000 |
| SL-08 | ALDH1L1 | Predicted | 0.000 | RB-26 | KRAS | Predicted | 0.045 |
| SL-08 | ALDH2 | Predicted | 0.003 | RB-26 | MAPK1 | Predicted | 0.084 |
| SL-08 | ASS1 | Predicted | 0.013 | RB-26 | MAPK3 | Predicted | 0.029 |
| SL-08 | CCNA2 | Predicted | 0.416 | RB-26 | MME | Predicted | 0.132 |
| SL-08 | CCT3 | Predicted | 0.007 | RB-26 | NME1 | Predicted | 0.013 |
| SL-08 | CDK4 | Predicted | 0.034 | RB-26 | NME2 | Predicted | 0.004 |
| SL-08 | CES1 | Predicted | 0.004 | RB-26 | PTGS2 | Predicted | 0.047 |
| SL-08 | EGFR | Predicted | 0.139 | RB-26 | RARA | Predicted | 0.015 |
| SL-08 | ESR1 | Predicted | 0.248 | RB-26 | RBP1 | Predicted | 0.005 |
| SL-08 | FTCD | Predicted | 0.000 | RB-26 | RHOA | Predicted | 0.004 |
| SL-08 | GAPDH | Predicted | 0.019 | RB-26 | RND3 | Predicted | 0.007 |
| SL-08 | GLUD1 | Predicted | 0.021 | RB-26 | SERPINE1 | Predicted | 0.020 |
| SL-08 | GSTP1 | Predicted | 0.113 | RB-26 | SGK1 | Predicted | 0.000 |
| SL-08 | HAO1 | Predicted | 0.002 | RB-26 | TGM2 | Predicted | 0.004 |
| SL-08 | HSD17B10 | Predicted | 0.003 | RB-26 | TOP2A | Predicted | 0.022 |

| SL-08 | KRAS | Predicted | 0.052 | RB-26 | TRDMT1 | Predicted | 0.003 |
| --- | --- | --- | --- | --- | --- | --- | --- |
| SL-08 | MAPK1 | Predicted | 0.064 | RB-27 | ACSL1 | Predicted | 0.022 |
| SL-08 | MAPK3 | Predicted | 0.034 | RB-27 | ALDH2 | Predicted | 0.080 |
| SL-08 | MME | Predicted | 0.336 | RB-27 | ALDOA | Predicted | 0.087 |
| SL-08 | MMP12 | Predicted | 0.039 | RB-27 | CA1 | Predicted | 0.175 |
| SL-08 | MTHFD1 | Predicted | 0.000 | RB-27 | CA2 | Predicted | 0.085 |
| SL-08 | MTR | Predicted | 0.000 | RB-27 | CCNA2 | Predicted | 0.257 |
| SL-08 | QDPR | Predicted | 0.003 | RB-27 | ESR1 | Predicted | 0.283 |
| SL-08 | RND3 | Predicted | 0.010 | RB-27 | GSTP1 | Predicted | 0.000 |
| SL-08 | SERPINE1 | Predicted | 0.019 | RB-27 | KRAS | Predicted | 0.007 |
| SL-08 | SGK1 | Predicted | 0.002 | RB-27 | LCN2 | Predicted | 0.087 |
| SL-08 | SRC | Predicted | 0.131 | RB-27 | MTAP | Predicted | 0.175 |
| SL-08 | TUBB | Predicted | 0.018 | RB-27 | NOS2A | Predicted | 0.267 |
| SL-08 | VEGFA | Predicted | 0.024 | RB-27 | PC | Predicted | 0.031 |
| SL-09 | ABCB1 | Predicted | 0.012 | RB-27 | PTGS2 | Predicted | 0.740 |
| SL-09 | ACSL1 | Predicted | 0.012 | RB-27 | TOP2A | Predicted | 0.038 |
| SL-09 | ADH1B | Predicted | 0.013 | RB-28 | ABCB1 | Predicted | 0.016 |
| SL-09 | ADH1C | Predicted | 0.003 | RB-28 | ACSL1 | Predicted | 0.016 |
| SL-09 | ADH4 | Predicted | 0.003 | RB-28 | ADH1C | Predicted | 0.018 |
| SL-09 | AKR1B10 | Predicted | 0.003 | RB-28 | AGXT | Predicted | 0.014 |
| SL-09 | AKR1C2 | Predicted | 0.003 | RB-28 | AKR1B10 | Predicted | 0.192 |
| SL-09 | AKT2 | Predicted | 0.054 | RB-28 | AKT2 | Predicted | 0.267 |
| SL-09 | ALDH1A1 | Predicted | 0.003 | RB-28 | ASS1 | Predicted | 0.016 |
| SL-09 | ALDH1L1 | Predicted | 0.001 | RB-28 | AURKA | Predicted | 0.013 |
| SL-09 | ALDH2 | Predicted | 0.003 | RB-28 | CA1 | Predicted | 0.061 |

| SL-09 | ASS1 | Predicted | 0.012 | RB-28 | CA2 | Predicted | 0.061 |
| --- | --- | --- | --- | --- | --- | --- | --- |
| SL-09 | CCNA2 | Predicted | 0.694 | RB-28 | CASP1 | Predicted | 0.034 |
| SL-09 | CCT3 | Predicted | 0.007 | RB-28 | CCNA2 | Predicted | 0.981 |
| SL-09 | DCXR | Predicted | 0.003 | RB-28 | CCT3 | Predicted | 0.032 |
| SL-09 | EGFR | Predicted | 0.145 | RB-28 | CDK4 | Predicted | 0.064 |
| SL-09 | ESR1 | Predicted | 0.249 | RB-28 | CES1 | Predicted | 0.046 |
| SL-09 | FTCD | Predicted | 0.001 | RB-28 | CTSB | Predicted | 0.196 |
| SL-09 | GAPDH | Predicted | 0.019 | RB-28 | ESR1 | Predicted | 0.601 |
| SL-09 | GLUD1 | Predicted | 0.017 | RB-28 | FGF1 | Predicted | 0.094 |
| SL-09 | GNMT | Predicted | 0.000 | RB-28 | FGF2 | Predicted | 0.094 |
| SL-09 | GSTP1 | Predicted | 0.116 | RB-28 | GABRE | Predicted | 0.009 |
| SL-09 | HSD17B10 | Predicted | 0.003 | RB-28 | GAPDH | Predicted | 0.032 |
| SL-09 | HSD17B4 | Predicted | 0.003 | RB-28 | GLUD1 | Predicted | 0.040 |
| SL-09 | KRAS | Predicted | 0.022 | RB-28 | GNAI1 | Predicted | 0.016 |
| SL-09 | MAPK1 | Predicted | 0.061 | RB-28 | GNMT | Predicted | 0.009 |
| SL-09 | MME | Predicted | 0.282 | RB-28 | GSTP1 | Predicted | 0.062 |
| SL-09 | MMP12 | Predicted | 0.029 | RB-28 | HAO1 | Predicted | 0.000 |
| SL-09 | MTHFD1 | Predicted | 0.012 | RB-28 | HSP90AA1 | Predicted | 0.013 |
| SL-09 | MTR | Predicted | 0.001 | RB-28 | HSP90B1 | Predicted | 0.013 |
| SL-09 | NME1 | Predicted | 0.134 | RB-28 | HSPA1B | Predicted | 0.013 |
| SL-09 | QDPR | Predicted | 0.003 | RB-28 | HSPA8 | Predicted | 0.013 |
| SL-09 | RARA | Predicted | 0.016 | RB-28 | KRAS | Predicted | 0.058 |
| SL-09 | RND3 | Predicted | 0.007 | RB-28 | MAPK1 | Predicted | 0.064 |
| SL-09 | SERPINE1 | Predicted | 0.021 | RB-28 | MAPK3 | Predicted | 0.132 |
| SL-09 | SRC | Predicted | 0.134 | RB-28 | MAT1A | Predicted | 0.013 |

| SL-09 | TRDMT1 | Predicted | 0.000 | RB-28 | MME | Predicted | 0.132 |
| --- | --- | --- | --- | --- | --- | --- | --- |
| SL-09 | TUBB | Predicted | 0.006 | RB-28 | MMP12 | Predicted | 0.063 |
| SL-09 | VEGFA | Predicted | 0.024 | RB-28 | MMP9 | Predicted | 0.034 |
| SL-10 | AURKA | Predicted | 0.621 | RB-28 | NME1 | Predicted | 0.054 |
| SL-10 | DDR1 | Predicted | 0.083 | RB-28 | NME2 | Predicted | 0.016 |
| SL-10 | GAPDH | Predicted | 0.089 | RB-28 | NOS2A | Predicted | 0.051 |
| SL-10 | PDGFRA | Predicted | 0.083 | RB-28 | PGK1 | Predicted | 0.013 |
| SL-10 | PTGS2 | Predicted | 0.098 | RB-28 | PTGS2 | Predicted | 0.062 |
| SL-10 | RAF1 | Predicted | 0.078 | RB-28 | PTK2 | Predicted | 0.013 |
| SL-10 | SRC | Predicted | 0.081 | RB-28 | RHOA | Predicted | 0.016 |
| SL-11 | ACSL1 | Predicted | 0.025 | RB-28 | RND3 | Predicted | 0.021 |
| SL-11 | ALDH2 | Predicted | 0.085 | RB-28 | SERPINE1 | Predicted | 0.035 |
| SL-11 | ALDOA | Predicted | 0.086 | RB-28 | SGK1 | Predicted | 0.000 |
| SL-11 | AURKA | Predicted | 0.083 | RB-28 | SRC | Predicted | 0.258 |
| SL-11 | CA1 | Predicted | 0.096 | RB-28 | SULT2A1 | Predicted | 0.021 |
| SL-11 | CA2 | Predicted | 0.063 | RB-28 | TGM2 | Predicted | 0.016 |
| SL-11 | CCNA2 | Predicted | 0.351 | RB-28 | TOP2A | Predicted | 0.117 |
| SL-11 | CTSB | Predicted | 0.084 | RB-28 | TRDMT1 | Predicted | 0.009 |
| SL-11 | ESR1 | Predicted | 0.458 | RB-28 | VEGFA | Predicted | 0.082 |
| SL-11 | GABRE | Predicted | 0.015 | RB-29 | ABCB1 | Predicted | 0.010 |
| SL-11 | GSTP1 | Predicted | 0.000 | RB-29 | ACSL1 | Predicted | 0.010 |
| SL-11 | KRAS | Predicted | 0.007 | RB-29 | ADH1C | Predicted | 0.004 |
| SL-11 | LCN2 | Predicted | 0.083 | RB-29 | AGXT | Predicted | 0.006 |
| SL-11 | MTAP | Predicted | 0.176 | RB-29 | AKT2 | Predicted | 0.199 |
| SL-11 | NOS2A | Validated | 1.000 | RB-29 | ASS1 | Predicted | 0.010 |

| SL-11 | PTGS2 | Predicted | 0.941 | RB-29 | CASP1 | Predicted | 0.019 |
| --- | --- | --- | --- | --- | --- | --- | --- |
| SL-11 | TOP2A | Predicted | 0.055 | RB-29 | CCNA2 | Predicted | 0.850 |
| SL-12 | EGFR | Predicted | 0.972 | RB-29 | CCT3 | Predicted | 0.012 |
| SL-13 | ABCG2 | Validated | 1.000 | RB-29 | CDK4 | Predicted | 0.043 |
| SL-13 | AR | Validated | 1.000 | RB-29 | CES1 | Predicted | 0.004 |
| SL-13 | CASP3 | Validated | 1.000 | RB-29 | CTSB | Predicted | 0.135 |
| SL-13 | CAT | Validated | 1.000 | RB-29 | ESR1 | Predicted | 0.277 |
| SL-13 | CDKN1A | Validated | 1.000 | RB-29 | FGF1 | Predicted | 0.065 |
| SL-13 | CDKN2A | Validated | 1.000 | RB-29 | FGF2 | Predicted | 0.065 |
| SL-13 | CLDN4 | Validated | 1.000 | RB-29 | GABRE | Predicted | 0.000 |
| SL-13 | COL1A1 | Validated | 1.000 | RB-29 | GAPDH | Predicted | 0.019 |
| SL-13 | CXCL2 | Validated | 1.000 | RB-29 | GLUD1 | Predicted | 0.005 |
| SL-13 | CYP3A4 | Validated | 1.000 | RB-29 | GNAI1 | Predicted | 0.005 |
| SL-13 | E2F1 | Validated | 1.000 | RB-29 | GNMT | Predicted | 0.003 |
| SL-13 | EGF | Validated | 1.000 | RB-29 | GSTP1 | Predicted | 0.039 |
| SL-13 | ESR1 | Validated | 1.000 | RB-29 | HAO1 | Predicted | 0.001 |
| SL-13 | FOS | Validated | 1.000 | RB-29 | KRAS | Predicted | 0.081 |
| SL-13 | GSTP1 | Validated | 1.000 | RB-29 | MAPK1 | Predicted | 0.124 |
| SL-13 | HAS2 | Validated | 1.000 | RB-29 | MAPK3 | Predicted | 0.043 |
| SL-13 | HSPA5 | Validated | 1.000 | RB-29 | MET | Predicted | 0.139 |
| SL-13 | HSPB1 | Validated | 1.000 | RB-29 | MME | Predicted | 0.089 |
| SL-13 | ICAM1 | Validated | 1.000 | RB-29 | MMP12 | Predicted | 0.117 |
| SL-13 | IGF2 | Validated | 1.000 | RB-29 | MMP9 | Predicted | 0.019 |
| SL-13 | IGFBP3 | Validated | 1.000 | RB-29 | NME1 | Predicted | 0.018 |
| SL-13 | IL2 | Validated | 1.000 | RB-29 | NME2 | Predicted | 0.005 |

| SL-13 | IRF1 | Validated | 1.000 | RB-29 | NOS2A | Predicted | 0.021 |
| --- | --- | --- | --- | --- | --- | --- | --- |
| SL-13 | MMP9 | Validated | 1.000 | RB-29 | RHOA | Predicted | 0.005 |
| SL-13 | MYC | Validated | 1.000 | RB-29 | RND3 | Predicted | 0.005 |
| SL-13 | NFKBIA | Validated | 1.000 | RB-29 | SERPINE1 | Predicted | 0.008 |
| SL-13 | NOS2A | Validated | 1.000 | RB-29 | SGK1 | Predicted | 0.001 |
| SL-13 | PARP1 | Validated | 1.000 | RB-29 | SRC | Predicted | 0.126 |
| SL-13 | PLAU | Validated | 1.000 | RB-29 | TGM2 | Predicted | 0.005 |
| SL-13 | PTENP1 | Validated | 1.000 | RB-29 | TOP2A | Predicted | 0.040 |
| SL-13 | PTGS2 | Validated | 1.000 | RB-29 | TRDMT1 | Predicted | 0.003 |
| SL-13 | RAF1 | Validated | 1.000 | RB-29 | VEGFA | Predicted | 0.050 |
| SL-13 | SERPINE1 | Validated | 1.000 | RB-30 | ABCB1 | Predicted | 0.011 |
| SL-13 | SPP1 | Validated | 1.000 | RB-30 | ACSL1 | Predicted | 0.011 |
| SL-13 | STAT1 | Validated | 1.000 | RB-30 | ADH1C | Predicted | 0.012 |
| SL-13 | TOP2A | Validated | 1.000 | RB-30 | AGXT | Predicted | 0.011 |
| SL-13 | VEGFA | Validated | 1.000 | RB-30 | AKR1B10 | Predicted | 0.139 |
| SL-13 | ACSL1 | Predicted | 0.026 | RB-30 | AKT2 | Predicted | 0.200 |
| SL-13 | ALDH2 | Predicted | 0.085 | RB-30 | ASS1 | Predicted | 0.011 |
| SL-13 | ALDOA | Predicted | 0.085 | RB-30 | AURKA | Predicted | 0.009 |
| SL-13 | CA1 | Predicted | 0.176 | RB-30 | CA1 | Predicted | 0.069 |
| SL-13 | CA2 | Predicted | 0.086 | RB-30 | CA2 | Predicted | 0.069 |
| SL-13 | CCNA2 | Predicted | 0.262 | RB-30 | CASP1 | Predicted | 0.028 |
| SL-13 | CTSB | Predicted | 0.082 | RB-30 | CCNA2 | Predicted | 1.000 |
| SL-13 | GABRE | Predicted | 0.010 | RB-30 | CCT3 | Predicted | 0.009 |
| SL-13 | KRAS | Predicted | 0.007 | RB-30 | CDK4 | Predicted | 0.050 |
| SL-13 | LCN2 | Predicted | 0.087 | RB-30 | CES1 | Predicted | 0.012 |

| SL-13 | MTAP | Predicted | 0.175 | RB-30 | CTSB | Predicted | 0.142 |
| --- | --- | --- | --- | --- | --- | --- | --- |
| SL-14 | EGFR | Predicted | 0.946 | RB-30 | ESR1 | Predicted | 0.289 |
| SL-15 | ACSL1 | Predicted | 0.024 | RB-30 | GABRE | Predicted | 0.004 |
| SL-15 | ALDH2 | Predicted | 0.086 | RB-30 | GLUD1 | Predicted | 0.026 |
| SL-15 | ALDOA | Predicted | 0.085 | RB-30 | GNAI1 | Predicted | 0.010 |
| SL-15 | AR | Predicted | 0.083 | RB-30 | GNMT | Predicted | 0.006 |
| SL-15 | CA1 | Predicted | 0.219 | RB-30 | GSTP1 | Predicted | 0.044 |
| SL-15 | CA2 | Predicted | 0.130 | RB-30 | HAO1 | Predicted | 0.000 |
| SL-15 | CASP3 | Validated | 1.000 | RB-30 | HSP90AA1 | Predicted | 0.009 |
| SL-15 | CCNA2 | Predicted | 0.261 | RB-30 | HSP90B1 | Predicted | 0.009 |
| SL-15 | CYP1A2 | Validated | 1.000 | RB-30 | HSPA1B | Predicted | 0.009 |
| SL-15 | CYP3A4 | Validated | 1.000 | RB-30 | HSPA8 | Predicted | 0.009 |
| SL-15 | ESR1 | Validated | 1.000 | RB-30 | KRAS | Predicted | 0.044 |
| SL-15 | GABRE | Predicted | 0.008 | RB-30 | MAPK1 | Predicted | 0.050 |
| SL-15 | GSTP1 | Validated | 1.000 | RB-30 | MAPK3 | Predicted | 0.101 |
| SL-15 | HAS2 | Validated | 1.000 | RB-30 | MAT1A | Predicted | 0.009 |
| SL-15 | ICAM1 | Validated | 1.000 | RB-30 | MME | Predicted | 0.097 |
| SL-15 | KRAS | Predicted | 0.007 | RB-30 | MMP12 | Predicted | 0.046 |
| SL-15 | LCN2 | Predicted | 0.085 | RB-30 | MMP9 | Predicted | 0.028 |
| SL-15 | MAPK1 | Predicted | 0.035 | RB-30 | NME1 | Predicted | 0.037 |
| SL-15 | MTAP | Predicted | 0.174 | RB-30 | NME2 | Predicted | 0.010 |
| SL-15 | NOS2A | Predicted | 0.174 | RB-30 | PGK1 | Predicted | 0.009 |
| SL-15 | PC | Predicted | 0.007 | RB-30 | PTGS2 | Predicted | 0.047 |
| SL-15 | PTGS2 | Predicted | 0.876 | RB-30 | PTK2 | Predicted | 0.009 |
| SL-15 | STAT1 | Validated | 1.000 | RB-30 | RHOA | Predicted | 0.010 |

| SL-15 | TOP2A | Predicted | 0.041 | RB-30 | RND3 | Predicted | 0.013 |
| --- | --- | --- | --- | --- | --- | --- | --- |
| SL-15 | TP53 | Validated | 1.000 | RB-30 | SERPINE1 | Predicted | 0.025 |
| SL-15 | VEGFA | Validated | 1.000 | RB-30 | SGK1 | Predicted | 0.000 |
| SL-16 | ADH1B | Predicted | 0.000 | RB-30 | SRC | Predicted | 0.138 |
| SL-16 | ADH1C | Predicted | 0.000 | RB-30 | TGM2 | Predicted | 0.010 |
| SL-16 | ADH4 | Predicted | 0.000 | RB-30 | TOP2A | Predicted | 0.077 |
| SL-16 | ALDH1A1 | Predicted | 0.000 | RB-30 | TRDMT1 | Predicted | 0.006 |
| SL-16 | ALDH2 | Predicted | 0.000 | RB-30 | VEGFA | Predicted | 0.066 |
| SL-16 | DDR1 | Predicted | 0.158 | RB-31 | ABCB1 | Predicted | 0.018 |
| SL-16 | EGFR | Predicted | 0.960 | RB-31 | ACSL1 | Predicted | 0.018 |
| SL-16 | GAPDH | Predicted | 0.000 | RB-31 | ADH1B | Predicted | 0.005 |
| SL-16 | GLUD1 | Predicted | 0.000 | RB-31 | ADH1C | Predicted | 0.026 |
| SL-16 | HSD17B10 | Predicted | 0.000 | RB-31 | ADH4 | Predicted | 0.005 |
| SL-16 | HSD17B4 | Predicted | 0.000 | RB-31 | AGXT | Predicted | 0.012 |
| SL-16 | PDGFRA | Predicted | 0.158 | RB-31 | AKT2 | Predicted | 0.312 |
| SL-16 | PTGS2 | Predicted | 0.250 | RB-31 | ALDH1A1 | Predicted | 0.005 |
| SL-16 | QDPR | Predicted | 0.000 | RB-31 | ALDH2 | Predicted | 0.005 |
| SL-16 | SRC | Predicted | 0.156 | RB-31 | ASS1 | Predicted | 0.018 |
| SL-17 | EGFR | Predicted | 0.942 | RB-31 | AURKA | Predicted | 0.012 |
| SL-17 | PTGS2 | Predicted | 0.033 | RB-31 | CASP1 | Predicted | 0.060 |
| SL-18 | ADH1B | Predicted | 0.027 | RB-31 | CCNA2 | Predicted | 0.656 |
| SL-18 | ADH1C | Predicted | 0.000 | RB-31 | CCT3 | Predicted | 0.035 |
| SL-18 | ADH4 | Predicted | 0.000 | RB-31 | CDK4 | Predicted | 0.052 |
| SL-18 | AKR1B10 | Predicted | 0.003 | RB-31 | CES1 | Predicted | 0.011 |
| SL-18 | AKR1C2 | Predicted | 0.003 | RB-31 | CTSB | Predicted | 0.211 |

| SL-18 | ALDH1A1 | Predicted | 0.000 | RB-31 | ESR1 | Predicted | 0.438 |
| --- | --- | --- | --- | --- | --- | --- | --- |
| SL-18 | ALDH2 | Predicted | 0.000 | RB-31 | FGF1 | Predicted | 0.101 |
| SL-18 | DCXR | Predicted | 0.003 | RB-31 | FGF2 | Predicted | 0.101 |
| SL-18 | GAPDH | Predicted | 0.000 | RB-31 | GABRE | Predicted | 0.002 |
| SL-18 | GLUD1 | Predicted | 0.000 | RB-31 | GAPDH | Predicted | 0.038 |
| SL-18 | HSD17B10 | Predicted | 0.000 | RB-31 | GLUD1 | Predicted | 0.052 |
| SL-18 | HSD17B4 | Predicted | 0.000 | RB-31 | GNAI1 | Predicted | 0.013 |
| SL-18 | MTHFD1 | Predicted | 0.003 | RB-31 | GNMT | Predicted | 0.006 |
| SL-18 | QDPR | Predicted | 0.000 | RB-31 | GSTP1 | Predicted | 0.057 |
| SL-19 | DDR1 | Predicted | 0.875 | RB-31 | HAO1 | Predicted | 0.000 |
| SL-19 | EGFR | Predicted | 0.874 | RB-31 | HSD17B10 | Predicted | 0.005 |
| SL-19 | PDGFRA | Predicted | 0.875 | RB-31 | HSD17B4 | Predicted | 0.005 |
| SL-20 | EGFR | Predicted | 0.947 | RB-31 | HSP90AA1 | Predicted | 0.012 |
| SL-21 | AURKA | Predicted | 0.313 | RB-31 | HSP90B1 | Predicted | 0.012 |
| SL-21 | DDR1 | Predicted | 0.025 | RB-31 | HSPA1B | Predicted | 0.012 |
| SL-21 | PDGFRA | Predicted | 0.025 | RB-31 | HSPA8 | Predicted | 0.012 |
| SL-21 | PTGS2 | Predicted | 0.062 | RB-31 | KRAS | Predicted | 0.121 |
| SL-21 | SRC | Predicted | 0.025 | RB-31 | MAPK1 | Predicted | 0.151 |
| SL-22 | AURKA | Predicted | 0.318 | RB-31 | MAPK3 | Predicted | 0.052 |
| SL-22 | DDR1 | Predicted | 0.025 | RB-31 | MAT1A | Predicted | 0.012 |
| SL-22 | EGFR | Predicted | 0.310 | RB-31 | MET | Predicted | 0.212 |
| SL-22 | PDGFRA | Predicted | 0.025 | RB-31 | MME | Predicted | 0.329 |
| SL-22 | PTGS2 | Predicted | 0.060 | RB-31 | MMP12 | Predicted | 0.068 |
| SL-22 | SRC | Predicted | 0.025 | RB-31 | MMP9 | Predicted | 0.060 |
| SL-23 | ACSL1 | Predicted | 0.033 | RB-31 | NME1 | Predicted | 0.057 |

| SL-23 | ALDH2 | Predicted | 0.152 | RB-31 | NME2 | Predicted | 0.013 |
| --- | --- | --- | --- | --- | --- | --- | --- |
| SL-23 | ALDOA | Predicted | 0.149 | RB-31 | PGK1 | Predicted | 0.012 |
| SL-23 | AR | Predicted | 0.108 | RB-31 | PTGS2 | Predicted | 0.047 |
| SL-23 | AURKA | Predicted | 0.002 | RB-31 | PTK2 | Predicted | 0.012 |
| SL-23 | CA1 | Predicted | 0.120 | RB-31 | QDPR | Predicted | 0.005 |
| SL-23 | CA2 | Predicted | 0.191 | RB-31 | RHOA | Predicted | 0.013 |
| SL-23 | CCT3 | Predicted | 0.002 | RB-31 | RND3 | Predicted | 0.015 |
| SL-23 | CDK4 | Predicted | 0.031 | RB-31 | SERPINE1 | Predicted | 0.029 |
| SL-23 | CES1 | Predicted | 0.009 | RB-31 | SGK1 | Predicted | 0.000 |
| SL-23 | CTSB | Predicted | 0.144 | RB-31 | SRC | Predicted | 0.102 |
| SL-23 | EGFR | Predicted | 0.031 | RB-31 | TGM2 | Predicted | 0.013 |
| SL-23 | ESR1 | Predicted | 0.781 | RB-31 | TOP2A | Predicted | 0.081 |
| SL-23 | FGF1 | Predicted | 0.020 | RB-31 | TRDMT1 | Predicted | 0.006 |
| SL-23 | FGF2 | Predicted | 0.020 | RB-31 | VEGFA | Predicted | 0.115 |
| SL-23 | GABRE | Predicted | 0.118 | RB-32 | ACSL1 | Predicted | 0.021 |
| SL-23 | GLUD1 | Predicted | 0.002 | RB-32 | AGXT | Predicted | 0.004 |
| SL-23 | GNAI1 | Predicted | 0.000 | RB-32 | AKR1B10 | Predicted | 0.105 |
| SL-23 | GSTP1 | Predicted | 0.040 | RB-32 | AURKA | Predicted | 0.000 |
| SL-23 | HGF | Predicted | 0.020 | RB-32 | CA1 | Predicted | 0.169 |
| SL-23 | HSP90AA1 | Predicted | 0.002 | RB-32 | CA2 | Predicted | 0.134 |
| SL-23 | HSP90B1 | Predicted | 0.002 | RB-32 | CCNA2 | Predicted | 1.000 |
| SL-23 | HSPA1B | Predicted | 0.002 | RB-32 | CCT3 | Predicted | 0.000 |
| SL-23 | HSPA8 | Predicted | 0.002 | RB-32 | CTSB | Predicted | 0.107 |
| SL-23 | KRAS | Predicted | 0.050 | RB-32 | ESR1 | Predicted | 0.106 |
| SL-23 | LYZ | Predicted | 0.003 | RB-32 | GABRE | Predicted | 0.004 |

| SL-23 | MAT1A | Predicted | 0.002 | RB-32 | GLUD1 | Predicted | 0.010 |
| --- | --- | --- | --- | --- | --- | --- | --- |
| SL-23 | MMP12 | Predicted | 0.007 | RB-32 | GNAI1 | Predicted | 0.003 |
| SL-23 | MMP14 | Predicted | 0.007 | RB-32 | GNMT | Predicted | 0.005 |
| SL-23 | MMP2 | Predicted | 0.007 | RB-32 | GSTP1 | Predicted | 0.005 |
| SL-23 | MMP3 | Predicted | 0.026 | RB-32 | HSP90AA1 | Predicted | 0.000 |
| SL-23 | MMP7 | Predicted | 0.007 | RB-32 | HSP90B1 | Predicted | 0.000 |
| SL-23 | MMP9 | Predicted | 0.007 | RB-32 | HSPA1B | Predicted | 0.000 |
| SL-23 | MTAP | Predicted | 0.151 | RB-32 | HSPA8 | Predicted | 0.000 |
| SL-23 | NME1 | Predicted | 0.025 | RB-32 | KRAS | Predicted | 0.038 |
| SL-23 | NME2 | Predicted | 0.000 | RB-32 | LYZ | Predicted | 0.107 |
| SL-23 | PGK1 | Predicted | 0.002 | RB-32 | MAPK3 | Predicted | 0.041 |
| SL-23 | PTGS2 | Predicted | 0.353 | RB-32 | MAT1A | Predicted | 0.000 |
| SL-23 | PTK2 | Predicted | 0.002 | RB-32 | MTAP | Predicted | 0.218 |
| SL-23 | REG1A | Predicted | 0.009 | RB-32 | NME1 | Predicted | 0.017 |
| SL-23 | RHOA | Predicted | 0.000 | RB-32 | NME2 | Predicted | 0.003 |
| SL-23 | SULT2A1 | Predicted | 0.102 | RB-32 | NOS2A | Predicted | 0.221 |
| SL-23 | TGM2 | Predicted | 0.000 | RB-32 | PGK1 | Predicted | 0.000 |
| SL-23 | TOP2A | Predicted | 0.144 | RB-32 | PTGS2 | Predicted | 0.221 |
| SL-24 | EGFR | Predicted | 0.898 | RB-32 | PTK2 | Predicted | 0.000 |
| SL-25 | ACSL1 | Predicted | 0.028 | RB-32 | RHOA | Predicted | 0.003 |
| SL-25 | AGXT | Predicted | 0.011 | RB-32 | RND3 | Predicted | 0.003 |
| SL-25 | ALDH2 | Predicted | 0.114 | RB-32 | SRC | Predicted | 0.106 |
| SL-25 | ALDOA | Predicted | 0.117 | RB-32 | SULT2A1 | Predicted | 0.003 |
| SL-25 | AURKA | Predicted | 0.002 | RB-32 | TGM2 | Predicted | 0.003 |
| SL-25 | CA1 | Predicted | 0.066 | RB-32 | TOP2A | Predicted | 0.024 |

| SL-25 | CA2 | Predicted | 0.124 | RB-32 | TRDMT1 | Predicted | 0.005 |
| --- | --- | --- | --- | --- | --- | --- | --- |
| SL-25 | CCT3 | Predicted | 0.002 | RB-33 | ABCB1 | Predicted | 0.008 |
| SL-25 | CTSB | Predicted | 0.115 | RB-33 | ACSL1 | Predicted | 0.027 |
| SL-25 | ESR1 | Predicted | 0.239 | RB-33 | AGXT | Predicted | 0.003 |
| SL-25 | GABRE | Predicted | 0.096 | RB-33 | AKT2 | Predicted | 0.084 |
| SL-25 | GLUD1 | Predicted | 0.002 | RB-33 | ASS1 | Predicted | 0.008 |
| SL-25 | GNAI1 | Predicted | 0.000 | RB-33 | AURKA | Predicted | 0.009 |
| SL-25 | GNMT | Predicted | 0.005 | RB-33 | CA1 | Predicted | 0.064 |
| SL-25 | GSTP1 | Predicted | 0.002 | RB-33 | CA2 | Predicted | 0.064 |
| SL-25 | HSP90AA1 | Predicted | 0.002 | RB-33 | CCNA2 | Predicted | 1.000 |
| SL-25 | HSP90B1 | Predicted | 0.002 | RB-33 | CCT3 | Predicted | 0.002 |
| SL-25 | HSPA1B | Predicted | 0.002 | RB-33 | CES1 | Predicted | 0.025 |
| SL-25 | HSPA8 | Predicted | 0.002 | RB-33 | CTSB | Predicted | 0.086 |
| SL-25 | KRAS | Predicted | 0.037 | RB-33 | GABRE | Predicted | 0.003 |
| SL-25 | LYZ | Predicted | 0.009 | RB-33 | GLUD1 | Predicted | 0.010 |
| SL-25 | MAT1A | Predicted | 0.002 | RB-33 | GNAI1 | Predicted | 0.001 |
| SL-25 | MMP12 | Predicted | 0.007 | RB-33 | GNMT | Predicted | 0.005 |
| SL-25 | MMP14 | Predicted | 0.007 | RB-33 | GSTP1 | Predicted | 0.001 |
| SL-25 | MMP2 | Predicted | 0.007 | RB-33 | HSP90AA1 | Predicted | 0.002 |
| SL-25 | MMP3 | Predicted | 0.021 | RB-33 | HSP90B1 | Predicted | 0.002 |
| SL-25 | MMP7 | Predicted | 0.007 | RB-33 | HSPA1B | Predicted | 0.002 |
| SL-25 | MMP9 | Predicted | 0.007 | RB-33 | HSPA8 | Predicted | 0.002 |
| SL-25 | MTAP | Predicted | 0.235 | RB-33 | KRAS | Predicted | 0.052 |
| SL-25 | NME1 | Predicted | 0.018 | RB-33 | MAPK1 | Predicted | 0.115 |
| SL-25 | NME2 | Predicted | 0.000 | RB-33 | MAPK3 | Predicted | 0.047 |

| SL-25 | NOS2A | Predicted | 0.117 | RB-33 | MAT1A | Predicted | 0.002 |
| --- | --- | --- | --- | --- | --- | --- | --- |
| SL-25 | PGK1 | Predicted | 0.002 | RB-33 | MME | Predicted | 0.054 |
| SL-25 | PTGS2 | Predicted | 0.582 | RB-33 | MMP14 | Predicted | 0.002 |
| SL-25 | PTK2 | Predicted | 0.002 | RB-33 | MMP2 | Predicted | 0.002 |
| SL-25 | RHOA | Predicted | 0.000 | RB-33 | MMP3 | Predicted | 0.002 |
| SL-25 | SRC | Predicted | 0.115 | RB-33 | MMP7 | Predicted | 0.002 |
| SL-25 | TGM2 | Predicted | 0.000 | RB-33 | MMP9 | Predicted | 0.002 |
| SL-25 | TRDMT1 | Predicted | 0.005 | RB-33 | MTAP | Predicted | 0.175 |
| RAM-01 | ABCB1 | Predicted | 0.004 | RB-33 | NME1 | Predicted | 0.009 |
| RAM-01 | ACSL1 | Predicted | 0.004 | RB-33 | NME2 | Predicted | 0.001 |
| RAM-01 | ASS1 | Predicted | 0.004 | RB-33 | NOS2A | Predicted | 0.084 |
| RAM-01 | CCNA2 | Predicted | 0.153 | RB-33 | PGK1 | Predicted | 0.002 |
| RAM-01 | CCT3 | Predicted | 0.006 | RB-33 | PTGS2 | Predicted | 0.099 |
| RAM-01 | CES1 | Predicted | 0.044 | RB-33 | PTK2 | Predicted | 0.002 |
| RAM-01 | CTSB | Predicted | 0.153 | RB-33 | RHOA | Predicted | 0.001 |
| RAM-01 | ESR1 | Predicted | 0.156 | RB-33 | RND3 | Predicted | 0.002 |
| RAM-01 | FGF1 | Predicted | 0.074 | RB-33 | SERPINE1 | Predicted | 0.024 |
| RAM-01 | FGF2 | Predicted | 0.074 | RB-33 | SRC | Predicted | 0.088 |
| RAM-01 | GAPDH | Predicted | 0.019 | RB-33 | SULT2A1 | Predicted | 0.006 |
| RAM-01 | GLUD1 | Predicted | 0.000 | RB-33 | TGM2 | Predicted | 0.001 |
| RAM-01 | KRAS | Predicted | 0.033 | RB-33 | TOP2A | Predicted | 0.046 |
| RAM-01 | LYZ | Predicted | 0.075 | RB-33 | TRDMT1 | Predicted | 0.005 |
| RAM-01 | MME | Predicted | 0.101 | RB-34 | ABCB1 | Predicted | 0.014 |
| RAM-01 | MMP12 | Predicted | 0.062 | RB-34 | ACSL1 | Predicted | 0.014 |
| RAM-01 | MMP14 | Predicted | 0.014 | RB-34 | AKT2 | Predicted | 0.058 |

| RAM-01 | MMP2 | Predicted | 0.014 | RB-34 | ALDH1L1 | Predicted | 0.013 |
| --- | --- | --- | --- | --- | --- | --- | --- |
| RAM-01 | MMP3 | Predicted | 0.014 | RB-34 | ASS1 | Predicted | 0.014 |
| RAM-01 | MMP7 | Predicted | 0.014 | RB-34 | AURKA | Predicted | 0.140 |
| RAM-01 | MMP9 | Predicted | 0.014 | RB-34 | CCNA2 | Predicted | 0.959 |
| RAM-01 | NME1 | Predicted | 0.157 | RB-34 | CCT3 | Predicted | 0.005 |
| RAM-01 | RND3 | Predicted | 0.000 | RB-34 | CYP2C8 | Predicted | 0.011 |
| RAM-01 | TOP2A | Predicted | 0.016 | RB-34 | DDR1 | Predicted | 0.015 |
| RAM-01 | TUBB | Predicted | 0.014 | RB-34 | ESR1 | Predicted | 0.041 |
| RAM-01 | VEGFA | Predicted | 0.041 | RB-34 | FTCD | Predicted | 0.013 |
| RAM-02 | ABCB1 | Predicted | 0.004 | RB-34 | GLUD1 | Predicted | 0.014 |
| RAM-02 | ACSL1 | Predicted | 0.004 | RB-34 | GNMT | Predicted | 0.002 |
| RAM-02 | ASS1 | Predicted | 0.004 | RB-34 | GSTP1 | Predicted | 0.022 |
| RAM-02 | CCNA2 | Predicted | 0.153 | RB-34 | KRAS | Predicted | 0.065 |
| RAM-02 | CCT3 | Predicted | 0.006 | RB-34 | LYZ | Predicted | 0.134 |
| RAM-02 | CES1 | Predicted | 0.044 | RB-34 | MTHFD1 | Predicted | 0.013 |
| RAM-02 | CTSB | Predicted | 0.153 | RB-34 | MTR | Predicted | 0.013 |
| RAM-02 | ESR1 | Predicted | 0.156 | RB-34 | NME1 | Predicted | 0.134 |
| RAM-02 | FGF1 | Predicted | 0.074 | RB-34 | NOS2A | Predicted | 0.136 |
| RAM-02 | FGF2 | Predicted | 0.074 | RB-34 | PDGFRA | Predicted | 0.015 |
| RAM-02 | GAPDH | Predicted | 0.019 | RB-34 | PTGS2 | Predicted | 0.095 |
| RAM-02 | GLUD1 | Predicted | 0.000 | RB-34 | RAF1 | Predicted | 0.015 |
| RAM-02 | KRAS | Predicted | 0.033 | RB-34 | RARA | Predicted | 0.086 |
| RAM-02 | LYZ | Predicted | 0.075 | RB-34 | RND3 | Predicted | 0.014 |
| RAM-02 | MME | Predicted | 0.101 | RB-34 | SERPINE1 | Predicted | 0.028 |
| RAM-02 | MMP12 | Predicted | 0.062 | RB-34 | TRDMT1 | Predicted | 0.002 |

| RAM-02 | MMP14 | Predicted | 0.014 | RB-34 | VEGFA | Predicted | 0.035 |
| --- | --- | --- | --- | --- | --- | --- | --- |
| RAM-02 | MMP2 | Predicted | 0.014 | RB-35 | ADH1B | Predicted | 0.024 |
| RAM-02 | MMP3 | Predicted | 0.014 | RB-35 | ADH1C | Predicted | 0.014 |
| RAM-02 | MMP7 | Predicted | 0.014 | RB-35 | ADH4 | Predicted | 0.014 |
| RAM-02 | MMP9 | Predicted | 0.014 | RB-35 | AKR1B10 | Predicted | 0.000 |
| RAM-02 | NME1 | Predicted | 0.157 | RB-35 | AKR1C2 | Predicted | 0.011 |
| RAM-02 | RND3 | Predicted | 0.000 | RB-35 | AKT2 | Predicted | 0.075 |
| RAM-02 | TOP2A | Predicted | 0.016 | RB-35 | ALDH1A1 | Predicted | 0.014 |
| RAM-02 | TUBB | Predicted | 0.014 | RB-35 | ALDH1B1 | Predicted | 0.001 |
| RAM-02 | VEGFA | Predicted | 0.041 | RB-35 | ALDH2 | Predicted | 0.014 |
| RAM-03 | GAPDH | Predicted | 0.087 | RB-35 | ALDH3A2 | Predicted | 0.001 |
| RAM-03 | MMP12 | Predicted | 0.204 | RB-35 | ALDH4A1 | Predicted | 0.001 |
| RAM-03 | TUBB | Predicted | 0.015 | RB-35 | CCNA2 | Predicted | 0.250 |
| RAM-04 | ABCB1 | Predicted | 0.016 | RB-35 | DCXR | Predicted | 0.000 |
| RAM-04 | ACSL1 | Predicted | 0.016 | RB-35 | GAPDH | Predicted | 0.038 |
| RAM-04 | ADH1B | Predicted | 0.017 | RB-35 | GLUD1 | Predicted | 0.027 |
| RAM-04 | ADH1C | Predicted | 0.010 | RB-35 | GSTP1 | Predicted | 0.249 |
| RAM-04 | ADH4 | Predicted | 0.010 | RB-35 | HSD17B10 | Predicted | 0.014 |
| RAM-04 | AKR1B10 | Predicted | 0.002 | RB-35 | HSD17B4 | Predicted | 0.014 |
| RAM-04 | AKR1C2 | Predicted | 0.009 | RB-35 | MMP12 | Predicted | 0.054 |
| RAM-04 | AKT2 | Predicted | 0.217 | RB-35 | MTHFD1 | Predicted | 0.011 |
| RAM-04 | ALDH1A1 | Predicted | 0.010 | RB-35 | QDPR | Predicted | 0.014 |
| RAM-04 | ALDH1B1 | Predicted | 0.002 | RB-35 | RND3 | Predicted | 0.003 |
| RAM-04 | ALDH1L1 | Predicted | 0.001 | RB-35 | VEGFA | Predicted | 0.034 |
| RAM-04 | ALDH2 | Predicted | 0.010 | RB-36 | ABCB1 | Predicted | 0.009 |

| RAM-04 | ALDH3A2 | Predicted | 0.002 | RB-36 | ACSL1 | Predicted | 0.009 |
| --- | --- | --- | --- | --- | --- | --- | --- |
| RAM-04 | ALDH4A1 | Predicted | 0.002 | RB-36 | ADH1C | Predicted | 0.008 |
| RAM-04 | ASS1 | Predicted | 0.016 | RB-36 | AGXT | Predicted | 0.009 |
| RAM-04 | CCNA2 | Predicted | 0.412 | RB-36 | AKT2 | Predicted | 0.142 |
| RAM-04 | CCT3 | Predicted | 0.010 | RB-36 | ASS1 | Predicted | 0.009 |
| RAM-04 | CDK4 | Predicted | 0.064 | RB-36 | AURKA | Predicted | 0.009 |
| RAM-04 | DCXR | Predicted | 0.002 | RB-36 | CA1 | Predicted | 0.070 |
| RAM-04 | EGFR | Predicted | 0.131 | RB-36 | CA2 | Predicted | 0.070 |
| RAM-04 | FTCD | Predicted | 0.001 | RB-36 | CCNA2 | Predicted | 0.983 |
| RAM-04 | GAPDH | Predicted | 0.024 | RB-36 | CCT3 | Predicted | 0.025 |
| RAM-04 | GLUD1 | Predicted | 0.025 | RB-36 | CDK4 | Predicted | 0.040 |
| RAM-04 | GNMT | Predicted | 0.007 | RB-36 | CES1 | Predicted | 0.008 |
| RAM-04 | GSTP1 | Predicted | 0.113 | RB-36 | CTSB | Predicted | 0.133 |
| RAM-04 | HAO1 | Predicted | 0.002 | RB-36 | ESR1 | Predicted | 0.284 |
| RAM-04 | HSD17B10 | Predicted | 0.010 | RB-36 | FGF1 | Predicted | 0.068 |
| RAM-04 | HSD17B4 | Predicted | 0.010 | RB-36 | FGF2 | Predicted | 0.068 |
| RAM-04 | KRAS | Predicted | 0.065 | RB-36 | GABRE | Predicted | 0.014 |
| RAM-04 | MAPK1 | Predicted | 0.133 | RB-36 | GAPDH | Predicted | 0.020 |
| RAM-04 | MAPK3 | Predicted | 0.064 | RB-36 | GLUD1 | Predicted | 0.023 |
| RAM-04 | MME | Predicted | 0.202 | RB-36 | GNAI1 | Predicted | 0.007 |
| RAM-04 | MMP12 | Predicted | 0.029 | RB-36 | GNMT | Predicted | 0.005 |
| RAM-04 | MTHFD1 | Predicted | 0.016 | RB-36 | HAO1 | Predicted | 0.002 |
| RAM-04 | MTR | Predicted | 0.001 | RB-36 | HSP90AA1 | Predicted | 0.009 |
| RAM-04 | NME1 | Predicted | 0.138 | RB-36 | HSP90B1 | Predicted | 0.009 |
| RAM-04 | NOS2A | Predicted | 0.135 | RB-36 | HSPA1B | Predicted | 0.009 |

| RAM-04 | QDPR | Predicted | 0.010 | RB-36 | HSPA8 | Predicted | 0.009 |
| --- | --- | --- | --- | --- | --- | --- | --- |
| RAM-04 | RND3 | Predicted | 0.009 | RB-36 | KRAS | Predicted | 0.102 |
| RAM-04 | SERPINE1 | Predicted | 0.022 | RB-36 | LYZ | Predicted | 0.067 |
| RAM-04 | SGK1 | Predicted | 0.002 | RB-36 | MAPK1 | Predicted | 0.111 |
| RAM-04 | TOP2A | Predicted | 0.163 | RB-36 | MAPK3 | Predicted | 0.087 |
| RAM-04 | TRDMT1 | Predicted | 0.007 | RB-36 | MAT1A | Predicted | 0.009 |
| RAM-04 | VEGFA | Predicted | 0.020 | RB-36 | MET | Predicted | 0.139 |
| RAM-05 | ABCB1 | Predicted | 0.005 | RB-36 | MME | Predicted | 0.091 |
| RAM-05 | ACSL1 | Predicted | 0.005 | RB-36 | MMP12 | Predicted | 0.042 |
| RAM-05 | AGXT | Predicted | 0.005 | RB-36 | NME1 | Predicted | 0.035 |
| RAM-05 | AKT2 | Predicted | 0.045 | RB-36 | NME2 | Predicted | 0.007 |
| RAM-05 | AR | Predicted | 0.065 | RB-36 | NOS2A | Predicted | 0.026 |
| RAM-05 | ASS1 | Predicted | 0.005 | RB-36 | PGK1 | Predicted | 0.009 |
| RAM-05 | AURKA | Predicted | 0.006 | RB-36 | PTGS2 | Predicted | 0.042 |
| RAM-05 | CA1 | Predicted | 0.038 | RB-36 | PTK2 | Predicted | 0.009 |
| RAM-05 | CA2 | Predicted | 0.038 | RB-36 | RHOA | Predicted | 0.007 |
| RAM-05 | CCNA2 | Predicted | 0.701 | RB-36 | RND3 | Predicted | 0.007 |
| RAM-05 | CCT3 | Predicted | 0.020 | RB-36 | SERPINE1 | Predicted | 0.021 |
| RAM-05 | CES1 | Predicted | 0.039 | RB-36 | SGK1 | Predicted | 0.002 |
| RAM-05 | CTSB | Predicted | 0.134 | RB-36 | SRC | Predicted | 0.184 |
| RAM-05 | ESR1 | Predicted | 0.290 | RB-36 | TGM2 | Predicted | 0.007 |
| RAM-05 | FGF1 | Predicted | 0.062 | RB-36 | TOP2A | Predicted | 0.077 |
| RAM-05 | FGF2 | Predicted | 0.062 | RB-36 | TRDMT1 | Predicted | 0.005 |
| RAM-05 | GABRE | Predicted | 0.002 | RB-36 | VEGFA | Predicted | 0.027 |
| RAM-05 | GAPDH | Predicted | 0.023 | RB-37 | ABCB1 | Predicted | 0.005 |

| RAM-05 | GLUD1 | Predicted | 0.026 | RB-37 | ACSL1 | Predicted | 0.005 |
| --- | --- | --- | --- | --- | --- | --- | --- |
| RAM-05 | GNMT | Predicted | 0.002 | RB-37 | AKT2 | Predicted | 0.203 |
| RAM-05 | HAO1 | Predicted | 0.002 | RB-37 | ASS1 | Predicted | 0.005 |
| RAM-05 | HSP90AA1 | Predicted | 0.006 | RB-37 | AURKA | Predicted | 0.007 |
| RAM-05 | HSP90B1 | Predicted | 0.006 | RB-37 | CCNA2 | Predicted | 1.000 |
| RAM-05 | HSPA1B | Predicted | 0.006 | RB-37 | CCT3 | Predicted | 0.020 |
| RAM-05 | HSPA8 | Predicted | 0.006 | RB-37 | CES1 | Predicted | 0.043 |
| RAM-05 | KRAS | Predicted | 0.093 | RB-37 | CTSB | Predicted | 0.139 |
| RAM-05 | LYZ | Predicted | 0.064 | RB-37 | ESR1 | Predicted | 0.135 |
| RAM-05 | MAT1A | Predicted | 0.006 | RB-37 | FGF1 | Predicted | 0.064 |
| RAM-05 | MET | Predicted | 0.133 | RB-37 | FGF2 | Predicted | 0.064 |
| RAM-05 | MME | Predicted | 0.089 | RB-37 | GABRE | Predicted | 0.015 |
| RAM-05 | MMP12 | Predicted | 0.132 | RB-37 | GLUD1 | Predicted | 0.033 |
| RAM-05 | MMP14 | Predicted | 0.004 | RB-37 | GNMT | Predicted | 0.000 |
| RAM-05 | MMP2 | Predicted | 0.004 | RB-37 | HSP90AA1 | Predicted | 0.007 |
| RAM-05 | MMP3 | Predicted | 0.004 | RB-37 | HSP90B1 | Predicted | 0.007 |
| RAM-05 | MMP7 | Predicted | 0.004 | RB-37 | HSPA1B | Predicted | 0.007 |
| RAM-05 | MMP9 | Predicted | 0.004 | RB-37 | HSPA8 | Predicted | 0.007 |
| RAM-05 | NME1 | Predicted | 0.019 | RB-37 | KRAS | Predicted | 0.063 |
| RAM-05 | NOS2A | Predicted | 0.021 | RB-37 | LYZ | Predicted | 0.064 |
| RAM-05 | PGK1 | Predicted | 0.006 | RB-37 | MAPK1 | Predicted | 0.059 |
| RAM-05 | PTGS2 | Predicted | 0.083 | RB-37 | MAT1A | Predicted | 0.007 |
| RAM-05 | PTK2 | Predicted | 0.006 | RB-37 | MME | Predicted | 0.090 |
| RAM-05 | RND3 | Predicted | 0.012 | RB-37 | MMP12 | Predicted | 0.044 |
| RAM-05 | SERPINE1 | Predicted | 0.031 | RB-37 | MMP14 | Predicted | 0.014 |

| RAM-05 | SGK1 | Predicted | 0.002 | RB-37 | MMP2 | Predicted | 0.014 |
| --- | --- | --- | --- | --- | --- | --- | --- |
| RAM-05 | SRC | Predicted | 0.183 | RB-37 | MMP3 | Predicted | 0.038 |
| RAM-05 | SULT2A1 | Predicted | 0.011 | RB-37 | MMP7 | Predicted | 0.014 |
| RAM-05 | TOP2A | Predicted | 0.055 | RB-37 | MMP9 | Predicted | 0.014 |
| RAM-05 | TRDMT1 | Predicted | 0.002 | RB-37 | NME1 | Predicted | 0.021 |
| RAM-05 | TUBB | Predicted | 0.014 | RB-37 | NOS2A | Predicted | 0.037 |
| RAM-05 | VEGFA | Predicted | 0.025 | RB-37 | PGK1 | Predicted | 0.007 |
| RAM-06 | ABCB1 | Predicted | 0.005 | RB-37 | PTGS2 | Predicted | 0.083 |
| RAM-06 | ACSL1 | Predicted | 0.005 | RB-37 | PTK2 | Predicted | 0.007 |
| RAM-06 | AGXT | Predicted | 0.005 | RB-37 | RND3 | Predicted | 0.018 |
| RAM-06 | AKT2 | Predicted | 0.045 | RB-37 | SRC | Predicted | 0.069 |
| RAM-06 | AR | Predicted | 0.065 | RB-37 | SULT2A1 | Predicted | 0.013 |
| RAM-06 | ASS1 | Predicted | 0.005 | RB-37 | TOP2A | Predicted | 0.028 |
| RAM-06 | AURKA | Predicted | 0.006 | RB-37 | TRDMT1 | Predicted | 0.000 |
| RAM-06 | CA1 | Predicted | 0.038 | RB-37 | VEGFA | Predicted | 0.031 |
| RAM-06 | CA2 | Predicted | 0.038 | RB-38 | ABCB1 | Predicted | 0.013 |
| RAM-06 | CCNA2 | Predicted | 0.701 | RB-38 | ACSL1 | Predicted | 0.013 |
| RAM-06 | CCT3 | Predicted | 0.020 | RB-38 | ADH1B | Predicted | 0.018 |
| RAM-06 | CES1 | Predicted | 0.039 | RB-38 | ADH1C | Predicted | 0.009 |
| RAM-06 | CTSB | Predicted | 0.134 | RB-38 | ADH4 | Predicted | 0.009 |
| RAM-06 | ESR1 | Predicted | 0.290 | RB-38 | AKR1B10 | Predicted | 0.000 |
| RAM-06 | FGF1 | Predicted | 0.062 | RB-38 | AKR1C2 | Predicted | 0.009 |
| RAM-06 | FGF2 | Predicted | 0.062 | RB-38 | AKT2 | Predicted | 0.134 |
| RAM-06 | GABRE | Predicted | 0.002 | RB-38 | ALDH1A1 | Predicted | 0.009 |
| RAM-06 | GAPDH | Predicted | 0.023 | RB-38 | ALDH1B1 | Predicted | 0.000 |

| RAM-06 | GLUD1 | Predicted | 0.026 | RB-38 | ALDH1L1 | Predicted | 0.001 |
| --- | --- | --- | --- | --- | --- | --- | --- |
| RAM-06 | GNMT | Predicted | 0.002 | RB-38 | ALDH2 | Predicted | 0.009 |
| RAM-06 | HAO1 | Predicted | 0.002 | RB-38 | ALDH3A2 | Predicted | 0.000 |
| RAM-06 | HSP90AA1 | Predicted | 0.006 | RB-38 | ALDH4A1 | Predicted | 0.000 |
| RAM-06 | HSP90B1 | Predicted | 0.006 | RB-38 | ASS1 | Predicted | 0.013 |
| RAM-06 | HSPA1B | Predicted | 0.006 | RB-38 | CCNA2 | Predicted | 0.560 |
| RAM-06 | HSPA8 | Predicted | 0.006 | RB-38 | CCT3 | Predicted | 0.008 |
| RAM-06 | KRAS | Predicted | 0.093 | RB-38 | CDK4 | Predicted | 0.041 |
| RAM-06 | LYZ | Predicted | 0.064 | RB-38 | DCXR | Predicted | 0.000 |
| RAM-06 | MAT1A | Predicted | 0.006 | RB-38 | EGFR | Predicted | 0.135 |
| RAM-06 | MET | Predicted | 0.133 | RB-38 | ESR1 | Predicted | 0.134 |
| RAM-06 | MME | Predicted | 0.089 | RB-38 | FTCD | Predicted | 0.001 |
| RAM-06 | MMP12 | Predicted | 0.132 | RB-38 | GAPDH | Predicted | 0.024 |
| RAM-06 | MMP14 | Predicted | 0.004 | RB-38 | GLUD1 | Predicted | 0.027 |
| RAM-06 | MMP2 | Predicted | 0.004 | RB-38 | GSTP1 | Predicted | 0.115 |
| RAM-06 | MMP3 | Predicted | 0.004 | RB-38 | HSD17B10 | Predicted | 0.009 |
| RAM-06 | MMP7 | Predicted | 0.004 | RB-38 | HSD17B4 | Predicted | 0.009 |
| RAM-06 | MMP9 | Predicted | 0.004 | RB-38 | KRAS | Predicted | 0.044 |
| RAM-06 | NME1 | Predicted | 0.019 | RB-38 | MAPK1 | Predicted | 0.121 |
| RAM-06 | NOS2A | Predicted | 0.021 | RB-38 | MAPK3 | Predicted | 0.041 |
| RAM-06 | PGK1 | Predicted | 0.006 | RB-38 | MME | Predicted | 0.207 |
| RAM-06 | PTGS2 | Predicted | 0.083 | RB-38 | MMP12 | Predicted | 0.039 |
| RAM-06 | PTK2 | Predicted | 0.006 | RB-38 | MTHFD1 | Predicted | 0.019 |
| RAM-06 | RND3 | Predicted | 0.012 | RB-38 | MTR | Predicted | 0.001 |
| RAM-06 | SERPINE1 | Predicted | 0.031 | RB-38 | NME1 | Predicted | 0.132 |

| RAM-06 | SGK1 | Predicted | 0.002 | RB-38 | NOS2A | Predicted | 0.132 |
| --- | --- | --- | --- | --- | --- | --- | --- |
| RAM-06 | SRC | Predicted | 0.183 | RB-38 | QDPR | Predicted | 0.009 |
| RAM-06 | SULT2A1 | Predicted | 0.011 | RB-38 | RND3 | Predicted | 0.009 |
| RAM-06 | TOP2A | Predicted | 0.055 | RB-38 | SERPINE1 | Predicted | 0.020 |
| RAM-06 | TRDMT1 | Predicted | 0.002 | RB-38 | SRC | Predicted | 0.138 |
| RAM-06 | TUBB | Predicted | 0.014 | RB-38 | TOP2A | Predicted | 0.171 |
| RAM-06 | VEGFA | Predicted | 0.025 | RB-38 | VEGFA | Predicted | 0.018 |
| RAM-10 | ACSL1 | Predicted | 0.028 | RB-39 | ADH1B | Predicted | 0.017 |
| RAM-10 | ALDH2 | Predicted | 0.101 | RB-39 | ADH1C | Predicted | 0.009 |
| RAM-10 | ALDOA | Predicted | 0.102 | RB-39 | ADH4 | Predicted | 0.009 |
| RAM-10 | AR | Predicted | 0.135 | RB-39 | AKR1B10 | Predicted | 0.000 |
| RAM-10 | CA1 | Predicted | 0.150 | RB-39 | AKR1C2 | Predicted | 0.008 |
| RAM-10 | CA2 | Predicted | 0.098 | RB-39 | AKT2 | Predicted | 0.047 |
| RAM-10 | CCNA2 | Predicted | 0.098 | RB-39 | ALDH1A1 | Predicted | 0.009 |
| RAM-10 | ESR1 | Predicted | 0.398 | RB-39 | ALDH1B1 | Predicted | 0.000 |
| RAM-10 | FGF1 | Predicted | 0.016 | RB-39 | ALDH1L1 | Predicted | 0.003 |
| RAM-10 | FGF2 | Predicted | 0.016 | RB-39 | ALDH2 | Predicted | 0.009 |
| RAM-10 | GABRE | Predicted | 0.032 | RB-39 | ALDH3A2 | Predicted | 0.000 |
| RAM-10 | GNAI1 | Predicted | 0.001 | RB-39 | ALDH4A1 | Predicted | 0.000 |
| RAM-10 | GSTP1 | Predicted | 0.000 | RB-39 | AURKA | Predicted | 0.161 |
| RAM-10 | HGF | Predicted | 0.016 | RB-39 | CCNA2 | Predicted | 0.326 |
| RAM-10 | KRAS | Predicted | 0.008 | RB-39 | CCT3 | Predicted | 0.008 |
| RAM-10 | LCN2 | Predicted | 0.097 | RB-39 | DCXR | Predicted | 0.000 |
| RAM-10 | LYZ | Predicted | 0.002 | RB-39 | DDR1 | Predicted | 0.025 |
| RAM-10 | MMP12 | Predicted | 0.004 | RB-39 | EGFR | Predicted | 0.162 |

| RAM-10 | MMP14 | Predicted | 0.003 | RB-39 | ESR1 | Predicted | 0.343 |
| --- | --- | --- | --- | --- | --- | --- | --- |
| RAM-10 | MMP2 | Predicted | 0.003 | RB-39 | FTCD | Predicted | 0.003 |
| RAM-10 | MMP3 | Predicted | 0.012 | RB-39 | GAPDH | Predicted | 0.009 |
| RAM-10 | MMP7 | Predicted | 0.003 | RB-39 | GLUD1 | Predicted | 0.009 |
| RAM-10 | MMP9 | Predicted | 0.003 | RB-39 | GSTP1 | Predicted | 0.106 |
| RAM-10 | MTAP | Predicted | 0.205 | RB-39 | HSD17B10 | Predicted | 0.009 |
| RAM-10 | NME1 | Predicted | 0.001 | RB-39 | HSD17B4 | Predicted | 0.009 |
| RAM-10 | NME2 | Predicted | 0.001 | RB-39 | MAPK1 | Predicted | 0.075 |
| RAM-10 | NOS2A | Predicted | 0.204 | RB-39 | MME | Predicted | 0.245 |
| RAM-10 | PC | Predicted | 0.036 | RB-39 | MTHFD1 | Predicted | 0.019 |
| RAM-10 | PTGS2 | Predicted | 1.000 | RB-39 | MTR | Predicted | 0.003 |
| RAM-10 | RHOA | Predicted | 0.001 | RB-39 | PDGFRA | Predicted | 0.025 |
| RAM-10 | SRC | Predicted | 0.063 | RB-39 | QDPR | Predicted | 0.009 |
| RAM-10 | SULT2A1 | Predicted | 0.087 | RB-39 | RAF1 | Predicted | 0.022 |
| RAM-10 | TGM2 | Predicted | 0.001 | RB-39 | TUBB | Predicted | 0.008 |
|  |  |  |  | RB-39 | VEGFA | Predicted | 0.028 |
|  |  |  |  | RB-40 | ABCB1 | Predicted | 0.003 |
|  |  |  |  | RB-40 | ACSL1 | Predicted | 0.003 |
|  |  |  |  | RB-40 | AGXT | Predicted | 0.004 |
|  |  |  |  | RB-40 | AKR1B10 | Predicted | 0.079 |
|  |  |  |  | RB-40 | AKT2 | Predicted | 0.117 |
|  |  |  |  | RB-40 | ASS1 | Predicted | 0.003 |
|  |  |  |  | RB-40 | AURKA | Predicted | 0.003 |
|  |  |  |  | RB-40 | CA1 | Predicted | 0.012 |
|  |  |  |  | RB-40 | CA2 | Predicted | 0.012 |

|  |  |  |  | RB-40 | CCNA2 | Predicted | 1.000 |
| --- | --- | --- | --- | --- | --- | --- | --- |
|  |  |  |  | RB-40 | CCT3 | Predicted | 0.011 |
|  |  |  |  | RB-40 | CDK4 | Predicted | 0.028 |
|  |  |  |  | RB-40 | CTSB | Predicted | 0.079 |
|  |  |  |  | RB-40 | GABRE | Predicted | 0.000 |
|  |  |  |  | RB-40 | GLUD1 | Predicted | 0.012 |
|  |  |  |  | RB-40 | GNAI1 | Predicted | 0.003 |
|  |  |  |  | RB-40 | GNMT | Predicted | 0.002 |
|  |  |  |  | RB-40 | HAO1 | Predicted | 0.000 |
|  |  |  |  | RB-40 | HSP90AA1 | Predicted | 0.003 |
|  |  |  |  | RB-40 | HSP90B1 | Predicted | 0.003 |
|  |  |  |  | RB-40 | HSPA1B | Predicted | 0.003 |
|  |  |  |  | RB-40 | HSPA8 | Predicted | 0.003 |
|  |  |  |  | RB-40 | KRAS | Predicted | 0.047 |
|  |  |  |  | RB-40 | MAPK1 | Predicted | 0.076 |
|  |  |  |  | RB-40 | MAPK3 | Predicted | 0.028 |
|  |  |  |  | RB-40 | MAT1A | Predicted | 0.003 |
|  |  |  |  | RB-40 | MME | Predicted | 0.052 |
|  |  |  |  | RB-40 | NME1 | Predicted | 0.018 |
|  |  |  |  | RB-40 | NME2 | Predicted | 0.003 |
|  |  |  |  | RB-40 | PGK1 | Predicted | 0.003 |
|  |  |  |  | RB-40 | PTGS2 | Predicted | 0.053 |
|  |  |  |  | RB-40 | PTK2 | Predicted | 0.003 |
|  |  |  |  | RB-40 | RHOA | Predicted | 0.003 |
|  |  |  |  | RB-40 | RND3 | Predicted | 0.004 |

|  |  |  |  | RB-40 | SERPINE1 | Predicted | 0.014 |
| --- | --- | --- | --- | --- | --- | --- | --- |
|  |  |  |  | RB-40 | SGK1 | Predicted | 0.000 |
|  |  |  |  | RB-40 | SRC | Predicted | 0.074 |
|  |  |  |  | RB-40 | TGM2 | Predicted | 0.003 |
|  |  |  |  | RB-40 | TOP2A | Predicted | 0.047 |
|  |  |  |  | RB-40 | TRDMT1 | Predicted | 0.002 |
|  |  |  |  | RB-40 | VEGFA | Predicted | 0.024 |
|  |  |  |  | RB-41 | ABCB1 | Predicted | 0.007 |
|  |  |  |  | RB-41 | ACSL1 | Predicted | 0.007 |
|  |  |  |  | RB-41 | AKT2 | Predicted | 0.070 |
|  |  |  |  | RB-41 | ALDH1A1 | Predicted | 0.002 |
|  |  |  |  | RB-41 | ALDH1L1 | Predicted | 0.009 |
|  |  |  |  | RB-41 | ASS1 | Predicted | 0.007 |
|  |  |  |  | RB-41 | CA1 | Predicted | 0.015 |
|  |  |  |  | RB-41 | CA2 | Predicted | 0.015 |
|  |  |  |  | RB-41 | CCNA2 | Predicted | 1.000 |
|  |  |  |  | RB-41 | CCT3 | Predicted | 0.005 |
|  |  |  |  | RB-41 | CYP2C8 | Predicted | 0.007 |
|  |  |  |  | RB-41 | EGFR | Predicted | 0.088 |
|  |  |  |  | RB-41 | ESR1 | Predicted | 0.160 |
|  |  |  |  | RB-41 | FTCD | Predicted | 0.009 |
|  |  |  |  | RB-41 | GLUD1 | Predicted | 0.008 |
|  |  |  |  | RB-41 | GNMT | Predicted | 0.003 |
|  |  |  |  | RB-41 | GSTP1 | Predicted | 0.026 |
|  |  |  |  | RB-41 | KRAS | Predicted | 0.047 |

|  |  |  |  | RB-41 | LYZ | Predicted | 0.087 |
| --- | --- | --- | --- | --- | --- | --- | --- |
|  |  |  |  | RB-41 | MAPK1 | Predicted | 0.040 |
|  |  |  |  | RB-41 | MME | Predicted | 0.134 |
|  |  |  |  | RB-41 | MTHFD1 | Predicted | 0.009 |
|  |  |  |  | RB-41 | MTR | Predicted | 0.009 |
|  |  |  |  | RB-41 | NME1 | Predicted | 0.089 |
|  |  |  |  | RB-41 | NOS2A | Predicted | 0.090 |
|  |  |  |  | RB-41 | PTGS2 | Predicted | 0.051 |
|  |  |  |  | RB-41 | RAF1 | Predicted | 0.011 |
|  |  |  |  | RB-41 | RARA | Predicted | 0.012 |
|  |  |  |  | RB-41 | RBP1 | Predicted | 0.002 |
|  |  |  |  | RB-41 | RND3 | Predicted | 0.008 |
|  |  |  |  | RB-41 | SERPINE1 | Predicted | 0.028 |
|  |  |  |  | RB-41 | TRDMT1 | Predicted | 0.003 |
|  |  |  |  | RB-41 | VEGFA | Predicted | 0.020 |
|  |  |  |  | RB-42 | ACSL1 | Predicted | 0.030 |
|  |  |  |  | RB-42 | AGXT | Predicted | 0.007 |
|  |  |  |  | RB-42 | ALDOA | Predicted | 0.132 |
|  |  |  |  | RB-42 | AR | Predicted | 0.220 |
|  |  |  |  | RB-42 | AURKA | Predicted | 0.005 |
|  |  |  |  | RB-42 | CA1 | Predicted | 0.120 |
|  |  |  |  | RB-42 | CA2 | Predicted | 0.120 |
|  |  |  |  | RB-42 | CCNA2 | Predicted | 0.137 |
|  |  |  |  | RB-42 | CCT3 | Predicted | 0.005 |
|  |  |  |  | RB-42 | CES1 | Predicted | 0.039 |

|  |  |  |  | RB-42 | CTSB | Predicted | 0.136 |
| --- | --- | --- | --- | --- | --- | --- | --- |
|  |  |  |  | RB-42 | ESR1 | Predicted | 0.679 |
|  |  |  |  | RB-42 | GABRE | Predicted | 0.021 |
|  |  |  |  | RB-42 | GLUD1 | Predicted | 0.005 |
|  |  |  |  | RB-42 | GNAI1 | Predicted | 0.004 |
|  |  |  |  | RB-42 | GSTP1 | Predicted | 0.000 |
|  |  |  |  | RB-42 | HSP90AA1 | Predicted | 0.005 |
|  |  |  |  | RB-42 | HSP90B1 | Predicted | 0.005 |
|  |  |  |  | RB-42 | HSPA1B | Predicted | 0.005 |
|  |  |  |  | RB-42 | HSPA8 | Predicted | 0.005 |
|  |  |  |  | RB-42 | KRAS | Predicted | 0.045 |
|  |  |  |  | RB-42 | MAPK3 | Predicted | 0.083 |
|  |  |  |  | RB-42 | MAT1A | Predicted | 0.005 |
|  |  |  |  | RB-42 | MMP12 | Predicted | 0.035 |
|  |  |  |  | RB-42 | MMP14 | Predicted | 0.009 |
|  |  |  |  | RB-42 | MMP2 | Predicted | 0.009 |
|  |  |  |  | RB-42 | MMP3 | Predicted | 0.028 |
|  |  |  |  | RB-42 | MMP7 | Predicted | 0.009 |
|  |  |  |  | RB-42 | MMP9 | Predicted | 0.009 |
|  |  |  |  | RB-42 | MTAP | Predicted | 0.280 |
|  |  |  |  | RB-42 | NME1 | Predicted | 0.016 |
|  |  |  |  | RB-42 | NME2 | Predicted | 0.004 |
|  |  |  |  | RB-42 | NOS2A | Predicted | 0.135 |
|  |  |  |  | RB-42 | PGK1 | Predicted | 0.005 |
|  |  |  |  | RB-42 | PTGS2 | Predicted | 0.453 |

|  |  |  |  | RB-42 | PTK2 | Predicted | 0.005 |
| --- | --- | --- | --- | --- | --- | --- | --- |
|  |  |  |  | RB-42 | RHOA | Predicted | 0.004 |
|  |  |  |  | RB-42 | SULT2A1 | Predicted | 0.011 |
|  |  |  |  | RB-42 | TGM2 | Predicted | 0.004 |
|  |  |  |  | RB-43 | ADH1B | Predicted | 0.013 |
|  |  |  |  | RB-43 | ADH1C | Predicted | 0.000 |
|  |  |  |  | RB-43 | ADH4 | Predicted | 0.000 |
|  |  |  |  | RB-43 | AKR1B10 | Predicted | 0.000 |
|  |  |  |  | RB-43 | AKR1C2 | Predicted | 0.013 |
|  |  |  |  | RB-43 | ALDH1A1 | Predicted | 0.000 |
|  |  |  |  | RB-43 | ALDH1B1 | Predicted | 0.000 |
|  |  |  |  | RB-43 | ALDH2 | Predicted | 0.000 |
|  |  |  |  | RB-43 | ALDH3A2 | Predicted | 0.000 |
|  |  |  |  | RB-43 | ALDH4A1 | Predicted | 0.000 |
|  |  |  |  | RB-43 | CASP3 | Validated | 1.000 |
|  |  |  |  | RB-43 | CAT | Validated | 1.000 |
|  |  |  |  | RB-43 | CCNA2 | Predicted | 0.320 |
|  |  |  |  | RB-43 | CCT3 | Predicted | 0.017 |
|  |  |  |  | RB-43 | DCXR | Predicted | 0.000 |
|  |  |  |  | RB-43 | ESR1 | Predicted | 0.321 |
|  |  |  |  | RB-43 | GAPDH | Predicted | 0.046 |
|  |  |  |  | RB-43 | GLUD1 | Predicted | 0.000 |
|  |  |  |  | RB-43 | GSTP1 | Predicted | 1.000 |
|  |  |  |  | RB-43 | HAO1 | Predicted | 0.001 |
|  |  |  |  | RB-43 | HAS2 | Validated | 1.000 |

|  |  |  |  | RB-43 | HSD17B10 | Predicted | 0.000 |
| --- | --- | --- | --- | --- | --- | --- | --- |
|  |  |  |  | RB-43 | HSD17B4 | Predicted | 0.000 |
|  |  |  |  | RB-43 | KRAS | Predicted | 0.128 |
|  |  |  |  | RB-43 | MMP12 | Predicted | 0.103 |
|  |  |  |  | RB-43 | MTHFD1 | Predicted | 0.013 |
|  |  |  |  | RB-43 | NOS2A | Validated | 1.000 |
|  |  |  |  | RB-43 | QDPR | Predicted | 0.000 |
|  |  |  |  | RB-43 | SGK1 | Predicted | 0.001 |
|  |  |  |  | RB-43 | SOD1 | Validated | 1.000 |
|  |  |  |  | RB-43 | VEGFA | Predicted | 0.030 |
|  |  |  |  | RB-44 | ABCB1 | Predicted | 0.008 |
|  |  |  |  | RB-44 | ACSL1 | Predicted | 0.008 |
|  |  |  |  | RB-44 | ADH1C | Predicted | 0.007 |
|  |  |  |  | RB-44 | AGXT | Predicted | 0.011 |
|  |  |  |  | RB-44 | AKR1B10 | Predicted | 0.189 |
|  |  |  |  | RB-44 | AKT2 | Predicted | 0.265 |
|  |  |  |  | RB-44 | ASS1 | Predicted | 0.008 |
|  |  |  |  | RB-44 | AURKA | Predicted | 0.010 |
|  |  |  |  | RB-44 | CA1 | Predicted | 0.057 |
|  |  |  |  | RB-44 | CA2 | Predicted | 0.057 |
|  |  |  |  | RB-44 | CASP1 | Predicted | 0.054 |
|  |  |  |  | RB-44 | CCNA2 | Predicted | 1.000 |
|  |  |  |  | RB-44 | CCT3 | Predicted | 0.029 |
|  |  |  |  | RB-44 | CDK4 | Predicted | 0.052 |
|  |  |  |  | RB-44 | CES1 | Predicted | 0.007 |

|  |  |  |  | RB-44 | CTSB | Predicted | 0.192 |
| --- | --- | --- | --- | --- | --- | --- | --- |
|  |  |  |  | RB-44 | ESR1 | Predicted | 0.194 |
|  |  |  |  | RB-44 | GLUD1 | Predicted | 0.034 |
|  |  |  |  | RB-44 | GNAI1 | Predicted | 0.010 |
|  |  |  |  | RB-44 | GNMT | Predicted | 0.006 |
|  |  |  |  | RB-44 | GSTP1 | Predicted | 0.166 |
|  |  |  |  | RB-44 | HAO1 | Predicted | 0.002 |
|  |  |  |  | RB-44 | HSP90AA1 | Predicted | 0.010 |
|  |  |  |  | RB-44 | HSP90B1 | Predicted | 0.010 |
|  |  |  |  | RB-44 | HSPA1B | Predicted | 0.010 |
|  |  |  |  | RB-44 | HSPA8 | Predicted | 0.010 |
|  |  |  |  | RB-44 | KRAS | Predicted | 0.058 |
|  |  |  |  | RB-44 | MAPK1 | Predicted | 0.115 |
|  |  |  |  | RB-44 | MAPK3 | Predicted | 0.052 |
|  |  |  |  | RB-44 | MAT1A | Predicted | 0.010 |
|  |  |  |  | RB-44 | MME | Predicted | 0.301 |
|  |  |  |  | RB-44 | MMP12 | Predicted | 0.078 |
|  |  |  |  | RB-44 | MMP9 | Predicted | 0.054 |
|  |  |  |  | RB-44 | NME1 | Predicted | 0.048 |
|  |  |  |  | RB-44 | NME2 | Predicted | 0.010 |
|  |  |  |  | RB-44 | PGK1 | Predicted | 0.010 |
|  |  |  |  | RB-44 | PLAU | Predicted | 0.146 |
|  |  |  |  | RB-44 | PTK2 | Predicted | 0.010 |
|  |  |  |  | RB-44 | RHOA | Predicted | 0.010 |
|  |  |  |  | RB-44 | RND3 | Predicted | 0.015 |

|  |  |  |  | RB-44 | SERPINE1 | Predicted | 0.015 |
| --- | --- | --- | --- | --- | --- | --- | --- |
|  |  |  |  | RB-44 | SGK1 | Predicted | 0.002 |
|  |  |  |  | RB-44 | SRC | Predicted | 0.192 |
|  |  |  |  | RB-44 | TGM2 | Predicted | 0.010 |
|  |  |  |  | RB-44 | TOP2A | Predicted | 0.120 |
|  |  |  |  | RB-44 | TRDMT1 | Predicted | 0.006 |
|  |  |  |  | RB-44 | VEGFA | Predicted | 0.104 |
|  |  |  |  | RB-45 | ABCB1 | Predicted | 0.012 |
|  |  |  |  | RB-45 | ACSL1 | Predicted | 0.012 |
|  |  |  |  | RB-45 | ADH1C | Predicted | 0.010 |
|  |  |  |  | RB-45 | AGXT | Predicted | 0.011 |
|  |  |  |  | RB-45 | AKR1B10 | Predicted | 0.187 |
|  |  |  |  | RB-45 | AKT2 | Predicted | 0.293 |
|  |  |  |  | RB-45 | ASS1 | Predicted | 0.012 |
|  |  |  |  | RB-45 | AURKA | Predicted | 0.010 |
|  |  |  |  | RB-45 | CA1 | Predicted | 0.057 |
|  |  |  |  | RB-45 | CA2 | Predicted | 0.057 |
|  |  |  |  | RB-45 | CASP1 | Predicted | 0.053 |
|  |  |  |  | RB-45 | CCNA2 | Predicted | 1.000 |
|  |  |  |  | RB-45 | CCT3 | Predicted | 0.029 |
|  |  |  |  | RB-45 | CDK4 | Predicted | 0.065 |
|  |  |  |  | RB-45 | CES1 | Predicted | 0.010 |
|  |  |  |  | RB-45 | CTSB | Predicted | 0.193 |
|  |  |  |  | RB-45 | ESR1 | Predicted | 0.392 |
|  |  |  |  | RB-45 | GLUD1 | Predicted | 0.033 |

|  |  |  |  | RB-45 | GNAI1 | Predicted | 0.009 |
| --- | --- | --- | --- | --- | --- | --- | --- |
|  |  |  |  | RB-45 | GNMT | Predicted | 0.006 |
|  |  |  |  | RB-45 | GSTP1 | Predicted | 0.164 |
|  |  |  |  | RB-45 | HAO1 | Predicted | 0.002 |
|  |  |  |  | RB-45 | HSP90AA1 | Predicted | 0.010 |
|  |  |  |  | RB-45 | HSP90B1 | Predicted | 0.010 |
|  |  |  |  | RB-45 | HSPA1B | Predicted | 0.010 |
|  |  |  |  | RB-45 | HSPA8 | Predicted | 0.010 |
|  |  |  |  | RB-45 | KRAS | Predicted | 0.044 |
|  |  |  |  | RB-45 | MAPK1 | Predicted | 0.139 |
|  |  |  |  | RB-45 | MAPK3 | Predicted | 0.065 |
|  |  |  |  | RB-45 | MAT1A | Predicted | 0.010 |
|  |  |  |  | RB-45 | MME | Predicted | 0.297 |
|  |  |  |  | RB-45 | MMP12 | Predicted | 0.077 |
|  |  |  |  | RB-45 | MMP9 | Predicted | 0.053 |
|  |  |  |  | RB-45 | NME1 | Predicted | 0.047 |
|  |  |  |  | RB-45 | NME2 | Predicted | 0.009 |
|  |  |  |  | RB-45 | PGK1 | Predicted | 0.010 |
|  |  |  |  | RB-45 | PTK2 | Predicted | 0.010 |
|  |  |  |  | RB-45 | RHOA | Predicted | 0.009 |
|  |  |  |  | RB-45 | RND3 | Predicted | 0.014 |
|  |  |  |  | RB-45 | SERPINE1 | Predicted | 0.016 |
|  |  |  |  | RB-45 | SGK1 | Predicted | 0.002 |
|  |  |  |  | RB-45 | SRC | Predicted | 0.257 |
|  |  |  |  | RB-45 | TGM2 | Predicted | 0.009 |

|  |  |  |  | RB-45 | TOP2A | Predicted | 0.118 |
| --- | --- | --- | --- | --- | --- | --- | --- |
|  |  |  |  | RB-45 | TRDMT1 | Predicted | 0.006 |
|  |  |  |  | RB-45 | VEGFA | Predicted | 0.099 |
[truncated: 24,134 more chars]
