# Supplementary material for: Molecular targets of Chinese herbs: a clinical study of hepatoma based on network pharmacology
Source: Sci Rep. 2016 May 4;6:24944. doi: 10.1038/srep24944 (PMC4855233; doi:10.1038/srep24944)
Supplement: Supplementary Information [file srep24944-s3.doc]

Table S4 TCM syndromes based effective formulae.

| Syndrome | Effective formula | Correlation coefficient |
| --- | --- | --- |
| Liver qi and blood stasis | *Carapacis Trionycis Bolus*  *Xiaochaihu Decoction* | 0.795  0.436 |
| Dampness-heat in Liver channe | *Cang Niu Fangji Decoction* | 0.620 |
| Deficiency of Spleen and Kidney | *Ganlu Xiaodu Pill* | 0.575 |
